# Low-Basicity 5-HT_6_ Receptor Ligands from the Group of Cyclic Arylguanidine Derivatives and Their Antiproliferative Activity Evaluation

**DOI:** 10.3390/ijms251910287

**Published:** 2024-09-24

**Authors:** Przemysław Zaręba, Anna K. Drabczyk, Artur Wnorowski, Maciej Maj, Katarzyna Malarz, Patryk Rurka, Gniewomir Latacz, Beata Duszyńska, Krzesimir Ciura, Katarzyna Ewa Greber, Anna Boguszewska-Czubara, Paweł Śliwa, Julia Kuliś

**Affiliations:** 1 Department of Chemical Technology and Environmental Analytics, Faculty of Chemical Engineering and Technology, Cracow University of Technology, 24 Warszawska Street, 31-155 Cracow, Poland; julia.kulis@student.pk.edu.pl; 2 Department of Organic Chemistry and Technology, Faculty of Chemical Engineering and Technology, Cracow University of Technology, 24 Warszawska Street, 31-155 Cracow, Poland; anna.drabczyk@pk.edu.pl (A.K.D.); pawel.sliwa@pk.edu.pl (P.Ś.); 3 Department of Biopharmacy, Faculty of Pharmacy, Medical University, 4a Chodźki Street, 20-093 Lublin, Poland; artur.wnorowski@umlub.pl (A.W.); maciejmaj@umlub.pl (M.M.); 4 Department of Systems Biology and Engineering, Silesian University of Technology, 11 Akademicka Street, 44-100 Gliwice, Poland; katarzyna.malarz@polsl.pl; 5 Institute of Physics, University of Silesia in Katowice, 1A 75 Pułku Piechoty Street, 41-500 Chorzow, Poland; patryk.rurka@us.edu.pl; 6 Department of Technology and Biotechnology of Drugs, Jagiellonian University Medical College, 9 Medyczna Street, 30-688 Cracow, Poland; gniewomir.latacz@uj.edu.pl; 7 Department of Medicinal Chemistry, Maj Institute of Pharmacology—Polish Academy of Sciences, 12 Smętna Street, 31-343 Cracow, Poland; duszyn@if-pan.krakow.pl; 8 Department of Physical Chemistry, Faculty of Pharmacy, Medical University of Gdansk, 107 Al. Gen. J. Hallera Street, 80-416 Gdansk, Poland; krzesimir.ciura@gumed.edu.pl (K.C.); katarzyna.greber@gumed.edu.pl (K.E.G.); 9 Laboratory of Environmental Chemoinformatics, Faculty of Chemistry, University of Gdansk, 63 Wita Stwosza Street, 80-308 Gdansk, Poland; 10 Department of Medical Chemistry, Medical University of Lublin, 4a Chodźki Street, 20-093 Lublin, Poland; anna.boguszewska-czubara@umlub.pl

**Keywords:** 5-HT_6_, anticancer, glioma, sonochemistry, microwave-assisted, ADMET, arylguanidine

## Abstract

The serotonin 5-HT_6_ receptor (5-HT_6_R), expressed almost exclusively in the brain, affects the Cdk5 signaling as well as the mTOR pathway. Due to the association of 5-HT_6_R signaling with pathways involved in cancer progression, we decided to check the usefulness of 5-HT_6_R ligands in the treatment of CNS tumors. For this purpose, a new group of low-base 5-HT_6_R ligands was developed, belonging to arylsulfonamide derivatives of cyclic arylguanidines. The selected group of molecules was also tested for their antiproliferative activity on astrocytoma (1321N1) and glioblastoma (U87MG, LN-229, U-251) cell lines. Some of the molecules were subjected to ADMET tests in vitro, including lipophilicity, drug binding to plasma proteins, affinity for phospholipids, drug–drug interaction (DDI), the penetration of the membrane (PAMPA), metabolic stability, and hepatotoxicity as well as in vivo cardiotoxicity in the *Danio rerio* model. Two antagonists with an affinity constant *K*_i_ < 50 nM (**PR 68** *K*_i_ = 37 nM) were selected. These compounds were characterized by very high selectivity. An analysis of pharmacokinetic parameters for the lead compound **PR 68** confirmed favorable properties for administration, including passive diffusion and acceptable metabolic stability (metabolized in 49%, MLMs). The compound did not exhibit the potential for drug–drug interactions.

## 1. Introduction 

The serotonin 5-HT_6_ receptor (5-HT_6_R) belongs to G_s_-coupled receptors, expressed almost exclusively in the brain [1]. This protein plays a key role in several neurodevelopmental processes, which makes it an attractive target in the search for drugs that improve cognitive functions [2]. 5-HT_6_R affects the cyclin-dependent kinase (Cdk) 5 signaling pathway, activating it and promoting the migration of cortical pyramidal neurons [3]. The 5-HT_6_R/Cdk5 association occurs in the absence of an agonist but can be disturbed by antagonists, indicating that it is a dynamic process dependent on the conformational state of the receptor. 5-HT_6_R has been found to induce Cdk5 activation in an agonist-independent manner in neuroblastoma and glioma cell lines [1]. The gain of 5-HT_6_R function in neurogliomas significantly increased the phosphorylation status of Cdk5 substrates required for neuronal migration, including focal adhesion kinase and microtubule-associated doublecortin [4]. Moreover, CDK5 expression has been linked to glioma aggressiveness. Higher CDK5 activity correlates with increased glioma cell growth and migration, which is further supported by evidence that the downregulation of CDK5 leads to reduced proliferation and induced apoptosis in glioma cells [5]. The 5-HT_6_R recruits Cdk5, which leads to the phosphorylation of the receptor itself, promoting the growth of neural processes via a Cdc42-dependent pathway [2]. This mechanism may be relevant in the context of gliomas, as the ability of tumor cells to migrate and invade is a key factor in tumor aggressiveness. In addition to its role in neuronal development, Cdk5 is implicated in the regulation of various signaling pathways that are critical for tumor cell proliferation and survival. Cdk5 activity can be modulated by its post-translational modifications, which may influence its role in glioma cell biology [6]. The phosphorylation of substrates by Cdk5 is essential for various cellular processes, including apoptosis and cell cycle regulation, which are key in the context of cancer [7]. 

This receptor is also associated with the mechanistic target of the rapamycin (mTOR) pathway. Abnormal mTOR signaling induced by 5-HT_6_R leads to cognitive deficits caused by neurodevelopmental changes. Although the mTOR pathway has been identified as a key regulator of neurodevelopmental processes, the role of mTOR signaling in 5-HT_6_R-dependent neuronal migration and neurite outgrowth remains to be determined [8]. The mTOR has been shown to control cell growth and survival, which is crucial in the PI3K/PTEN/Akt signaling pathway. This signaling network with mTOR works by integrating a series of environmental stimuli to regulate cell growth, proliferation, cell metabolism, protein synthesis, and cell death by autophagy [9]. Increased levels of mTOR are associated with various pathological conditions. This happens in the case of high-grade gliomas, characterized by a high tendency for proliferation, migration, and metastasis [10]. The mTOR/5-HT_6_R signaling is also associated with neurofibromatosis type 1 (NF1), an autosomal dominant disease characterized by “café au lait” macules and tumors of the central and peripheral nervous system [11]. Recent research has shown that neurofibromin physically interacts with and promotes constitutive activity of 5-HT_6_R [12].

Epidemiological studies indicate that patients with schizophrenia often show a reduced incidence of cancer after long-term pharmacological treatment [13]. The second-generation antipsychotic drug sertindole has been shown to have broad antiproliferative effects, in particular on breast cancer cell lines. Sertindole has been proven to cause cell death through apoptosis associated with autophagy. Moreover, the inhibition of 5-HT_6_R is involved in this process [14]. The 5-HT_6_R is one of the receptors coupled to the Gs protein, increasing the production of cAMP [1]. The inhibition of Gs—cAMP signaling may induce autophagy [15]. Sertindole and 5-HT_6_R antagonists may be effective in the treatment of breast-to-brain metastases due to their ability to penetrate the blood–brain barrier (BBB).

5-HT_6_R antagonists improve cognitive performance. The administration of selective 5-HT_6_R antagonists increases brain glutamate levels, leading to synaptic plasticity and improved cognitive function. Cognitive deficits are common in patients with brain tumors, and increasing neurotransmitter levels could potentially alleviate these deficits [16]. Moreover, the neuroprotective properties of 5-HT_6_R antagonists have been highlighted in studies in which these compounds have shown glioprotective effects against cytotoxicity induced by various agents, including doxorubicin [17]. 5-HT_6_R antagonists may not only improve cognitive function but also provide protective effects on neuronal cells. The ability of antagonists to enhance the efficacy of other therapeutic agents, such as gabapentinoids, further underscores their potential utility in treating cognitive deficits and pain associated with glioma [18]. An additional effect on neuroinflammatory processes may also play a role in their therapeutic potential, as inflammation is a significant factor in glioma progression and patient outcomes [19].

Despite ongoing clinical trials and promising results from preclinical studies, the effectiveness of 5-HT_6_R antagonists as adjunctive therapy in the treatment of Alzheimer’s disease (AD) has not yet been confirmed [16]. They are effective in the treatment of schizophrenia by alleviating positive symptoms and having a pro-cognitive effect [16]. Most 5-HT_6_R antagonists belong to basic diarylsulfones, which may lead to insufficient permeability of the BBB or low metabolic stability [20,21]. An interesting approach to solving this problem is reducing the ligands’ basicity. In the case of G protein-coupled receptors, it is postulated that the key interaction for high affinity is the presence of a protonable, strongly basic nitrogen atom in the structure of the ligand, which can form salt bridge-type interactions with the D3.32 residue [22]. Interestingly, in recent years, structures of ligands with a high binding potential, without a basic nitrogen atom, have been published [23,24,25], which seem to question this rule. For the 5-HT_6_R, libraries of reduced basicity ligands have also been published [21,26,27]. Particularly noteworthy is the group of non-basic *N*-phenylsulfonylindoles, which verifies the structural minimum for binding to the 5-HT_6_R. These are two phenyl rings and a connector stabilizing them in a specific, optimal position [21]. An interesting chemotype for serotonin receptor ligands is 4-methyl-3,4-dihydroquinazolin-2-amine derivatives. These compounds have high basicity due to the presence of a guanidine moiety. However, despite the simple structure deviating from the classic chemotype of serotonin receptor ligands, these compounds have a high affinity for 5-HT_5A_R and 5-HT_7_R and a moderate affinity for 5-HT_1A_R [28,29]. The affinity for the 5-HT_6_R in the case of compounds from this group was relatively low.

**Figure 1:**
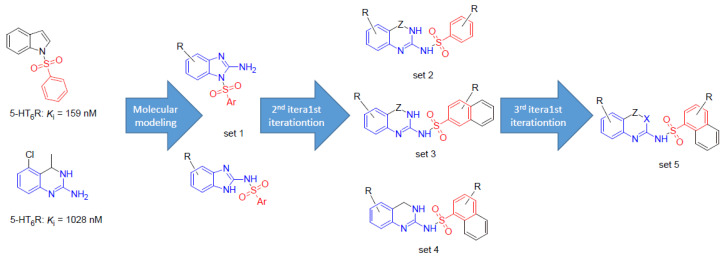
Reference ligands and the chemotype structure diagram in this publication.

As part of this publication, it was decided to check the usefulness of 5-HT_6_R ligands in the treatment of CNS tumors. For this purpose, a new group of low-base 5-HT_6_R ligands was developed, and their anticancer effectiveness was evaluated (Figure 1). The ligand chemotype was based on a hybrid of cyclic arylguanidines, described as 5-HT_5A_/5-HT_7_ receptor ligands, and *N*-phenylsulfonylindoles, as non-basic 5-HT_6_R ligands.

## 2. Results and Discussion

This research aimed to develop a new group of low-base 5-HT_6_R ligands and assess their potential in the treatment of brain tumors. The new chemotype was selected based on a virtual screening of the arylguanidine set. The parameter determining the selection of compounds or groups for synthesis was the GlideScore (empirical scoring function that approximates the ligand binding free energy), but the results were analyzed for coherence with known receptor ligands. Virtual screening allowed for the selection of five groups of ligands, all of which constitute the library of arylsulfonamide derivatives of cyclic arylguanidines. In the next step, the designed compounds were obtained and their affinity for the 5-HT6 receptor was determined in vitro. For most potent compounds, an extended receptor profile was assessed (5-HT_1A_R, 5-HT_5A_, 5-HT_7_R), and dopamine (D_2_R), as well as functions for these receptors. The selected group of molecules was also assessed for their antiproliferative activity on astrocytoma (1321N1) and glioblastoma (U87MG, LN-229, U-251) cell lines.

Their expected antiproliferative effect is related to the promotion of apoptosis and indirect modulation of CDK-5 and mTOR kinase signaling. For the study of anticancer activity, a set of compounds with low, medium, and high affinity for the 5-HT_6_R, showing similar structural features, was selected. The antiproliferative profiling has also been extended to breast (MCF7) and pancreatic (AsPC-1) cancer cells. The selectivity studies for the most promising compound were performed on human normal astrocyte cells (NHAs). Some of the molecules were subjected to ADMET tests in vitro, including lipophilicity, drug binding to plasma proteins, affinity for phospholipids, drug–drug interaction (DDI), the penetration of the membrane (PAMPA), metabolic stability, and hepatotoxicity as well as in vivo cardiotoxicity in the *Danio rerio* model. Structure–Activity Relationship (SAR) was discussed using molecular modeling methods.

### 2.1. Chemistry

**Scheme 1:**
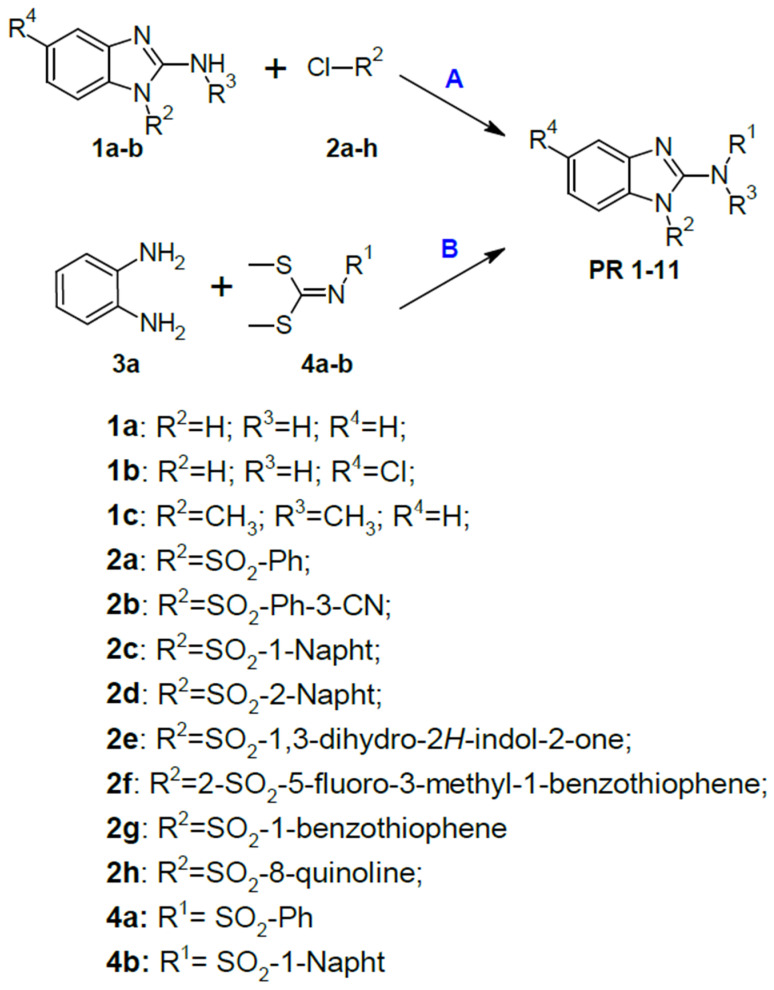
Method of synthesis of compounds from first group ^a^. ^a^ Reaction and conditions—A: acetone, MW 30 s; B: K_2_CO_3_, EtOH, reflux of 2 h or acetone, MW 30 s.

As part of this research, five sets of ligands were obtained. The first core consisted of arylsulfone derivatives of 1*H*-1,3-benzimidazol-2-amine (set 1, Figure 1). The set contains nine derivatives, **PR 1–9**, in which the arylsulfone group is attached to the nitrogen atom in the benzimidazole ring. The compounds were obtained by the reaction between 1*H*-1,3-benzimidazol-2-amine **1a** or its chlorinated analog **1b** and the corresponding arylsulfonyl chloride **2a–h**. The reactions were carried out according to the previously developed, microwave-assisted synthesis method (**A**) in acetone [30]. The next three ligands contain an arylsulfone group attached to the amine in the second position of benzimidazole **PR 10–12**, including the **PR 12** derivative with methylated unsubstituted nitrogen atoms. **PR 10–11** were obtained by the reaction between *o*-phenylenediamine **3a** and dimethyl (arylsulfonyl)carbonodithioimidate **4a–b**, carried out in the presence of K_2_CO_3_ and ethanol (EtOH) in reflux (**B**). **PR 12** was obtained by methylating 2-aminobenzimidazole **1a** with methyl iodide in KOH and acetone, then reacting the resulting product **1c** with arylsulfonyl chloride **2d**. The reactions were carried out in a microwave radiation field in acetone (**A**) (Scheme 1).

**Scheme 2:**
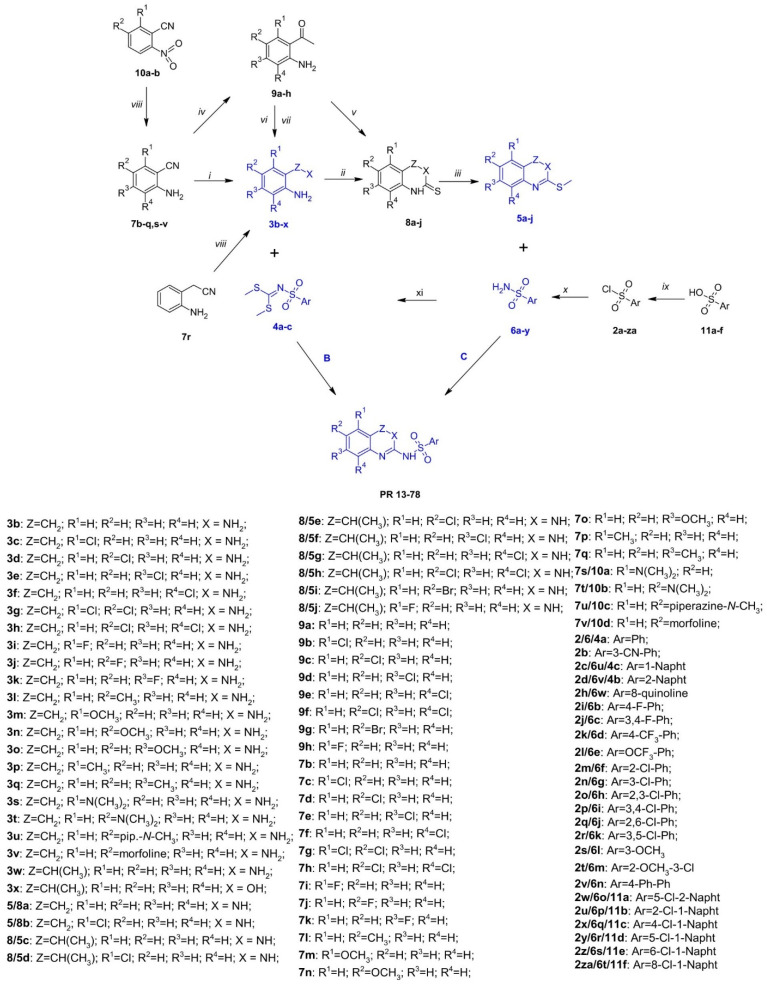
Method of synthesis of compounds belongs to group 2–5 ^b^. ^b^ Reaction and conditions—**B**: K_2_CO_3_, EtOH, reflux of 2h in **B^1^** or))) 30 min in **B^2^**; **C**: TEA, 180 °C, 3 h in **C^1^** or K_2_CO_3_, TBAB, MW 30 s in **C^2^**, (i) NaBH_4_/CF_3_COOH, THF, RT of 3–8 h or BH_3_/THF, THF; (ii) CS_2_, EtOH, reflux, 6 h; (iii) CH_3_I, acetone, RT, 24 h; (iv) CH_3_MgBr, Et_2_O; (v) NaBH_4_, EtOH, KSCN, H_2_O, HCl; (vi) CH_3_NH, EtOH; (vii) NaBH_4_, EtOH; (viii) Sn/HCl, reflux of 1 h; (ix) Na_2_CO_3_/H_2_O at 100 °C, POCl_3_, reflux of 3 h; (x) NH_3_/H_2_O, acetone, RT; (xi) CS_2_/DMF, KOH at 0–5 °C, CH_3_I at 5–10 °C.

The remaining ligands (set 2–5, Figure 1) were obtained using two previously developed complementary methods [30,31]. In path **B**, reactions between the appropriate aryldiamine **3a–x** and dimethyl (arylsulfonyl)carbonodithioimidate **4a–c** were carried out in ethanol with K_2_CO_3_ under reflux according to variant **B^1^** or in ultrasound, according to method **B^2^**. In **C**, the derivatives of 2-(methylsulfanyl)-3,4-dihydroquinazoline **5a–j** reacted with arylsulfonamide **6a–y**. The reactions were carried out in the presence of triethylamine (TEA) at 180 °C for 3 h in **C^1^** or K_2_CO_3_ and tetra-n-butylammonium bromide (TBAB), microwave (MW) 30 s in **C^2^** (Scheme 2).

The reactants were obtained according to known procedures. Derivatives of 2-(methylsulfanyl)-3,4-dihydroquinazoline **5a–b** were synthesized in three steps, starting from the corresponding 2-aminobenzonitrile **7a-p**, which in the first step were reduced with BH_3_/THF [31,32] or NaBH_4_/CF_3_COOH in THF [33]. The obtained amines **3b–p** were used directly for the synthesis of final products, according to path **B**, or converted into 3,4-dihydroquinazoline-2(1H)-thione derivatives **8a–b** by a reaction with CS_2_ in EtOH [34]. In the final step, **8a–b** were methylated with methyl iodide in acetone [28]. 2-[1-(methylamino)ethyl]aniline **3w** and 1-(2-aminophenyl)ethan-1-ol **3x** were obtained from 1-(2-aminophenyl)ethan-1-one **9a**, by a reaction with methylamine in ethanol followed by the reduction of the resulting imine with NaBH_4_ for **3w** [35] or direct reduction with NaBH_4_ for **3x**. Substituted 1-(2-aminophenyl)ethan-1-one **9b–h** were obtained from the corresponding nitriles **7b–p** by a reaction with methylmagnesium bromide in Et_2_O. The resulting products **9a–h** were converted into 4-methyl-3,4-dihydroquinazoline-2(1H)-thione derivatives **8c–j** in a two-step reaction consisting of NaBH_4_/EtOH reduction followed by cyclization with KSCN in the presence of HCl [28,29]. The resulting products were methylated, analogously to derivatives **5a–b**.

N1 disubstituted derivatives of 2-(aminomethyl)benzene-1,4-diamine or 2-(aminomethyl)benzene-1,3-diamine **3s–v** were obtained by a three-step synthesis from 2-fluoro-6-nitrobenzonitrile **10a** or 2-fluoro-5-nitrobenzonitrile **10b [36]**. The substrate was substituted with the appropriate amine in dimethylformamide (DMF), using TEA. In the next step, the nitro group was reduced with Sn in HCl [37]. The obtained 2-aminobenzonitriles **7s–v** were reduced to diamine **3s–v** according to the procedure described for **3b–p**.

Chloro-substituted derivatives of naphthalenesulfonyl chlorides **2v-za** were obtained as a result of a three-step reaction with aminonaphthalene sulfonic acids, which in the first step were subjected to the Sandmayer reaction, by diazotizing with NaNO_2_ in HCl and then heating the resulting aryldiamonium salt with CuCl in HCl [38]. After converting the obtained products **11a–f** into their sodium salts with Na_2_CO_3_ in water, they were converted to **2v–za** with POCl_3_ [39]. The obtained derivatives were converted into sulfonamide equivalents **6r–v [40]** by adding an aqueous solution of ammonia in acetone. A similar procedure was used for commercial arylsulfochlorides **2i–u**, obtaining arylsulfonamides **6a–p** from them. The dimethyl (arylsulfonyl)carbonodithioimidate **4a–c** were obtained by reacting the corresponding arylsulfonamide **6a,p,w** with CS_2_ in the DMF/KOH system, and then the resulting intermediate products were methylated with methyl iodide, without isolating the intermediate product.

### 2.2. Radioligand Binding and SAR Study

Next, the ligands’ affinity for the 5-HT_6_ receptors was evaluated in the [^3^H]-LSD radioligand binding assay. Experiments were performed in a stable HEK293 cell line expressing the human 5-HT_6_R.

The research began with the analysis of a group of arylsulfone derivatives of 2-aminobenzimidazole. In the first series of compounds, a trend of a significant increase in affinity was observed when using simple bicyclic aryl groups on the sulfone part (Table 1).

However, the binding constants were not particularly high (*K*_i_ = 0.133 µM for **PR 9**). Changing the arylsulfone substitution site from the ring nitrogen atom to the free amino group (**PR 10–12**) did not result in a significant increase in affinity. Importantly, the substitution of unsubstituted nitrogen atoms with methyls in **PR 12** resulted in a complete loss of affinity, indicating the key importance of the hydrogen bond donor in this region. The obtained SAR results clearly indicate an almost complete loss of affinity in the case of decorating the aryl group at the sulfone with additional hydrogen bond donors or acceptors (**PR 2** and **PR 5**). The loss of activity of **PR 7** compared to **PR 8** also seems interesting. In this case, it can be explained by too large aryl substituents. The greatest increase in affinity was observed when a bicyclic substituent was used. However, when comparing the compound **PR 1** and **PR 10**, substitution with an aryl sulphonic group at the nitrogen atom in the benzimidazole ring appears less favorable than at the free amino group in the second position. Based on this observation, we decided to investigate a group of derivatives having an additional methyl bridge between the aryl group and the guanidine fragment (transition from 2-aminobenzimidazole derivatives to 2-amino-3,4-dihydroquinazoline analogs).

The second group consisted of phenylsulfone derivatives of 2-amino-3,4-dihydroquinazoline. The influence of substitution in the ring at the sulfone group and in the 3,4-dihydroquinazoline region on the activity was checked (Table 2).

Importantly, only chlorine substitution in positions *2-* and *3-* of the phenylsulfone group **PR 20** caused the affinity constant to drop below 0.300 µM. Comparing the structure with **PR 19** and **PR 23**, it can be assumed that the *3*-position in the phenylsulfone ring is particularly sensitive to modifications. However, the expansion of the arylsulfone group at the second, third, and fourth positions in most cases resulted in an increase in affinity compared to the unsubstituted compound, which prompted us to synthesize two further sets of compounds with extended arylsulfone groups. To overcome this limitation, a group of 2-naphthalenesulfonamide analogs for the second group were synthesized, to obtain a similar effect as in the first set. Surprisingly, the use of this core also did not achieve the desired results. The unsubstituted derivative of **PR 29** showed very low affinity, and its methyl decoration at the *4*-position raised it only to 0.702 µM in the case of **PR 34** (Table 3).

Taking into account the results presented for previous sets, indicating that the use of a bicyclic arylsulfone and the expansion of the phenylsulfone group in the second and third positions lead to an increase in affinity, we examined the group of 1-naphthyl derivatives. Even the basic ligand **PR 40** showed higher affinity than all molecules from the previous group (*K*_i_ = 0.651 µM) (Table 4). 

Decorations in the 1-naphthalenesulfonamide ring with chlorine atoms resulted in a further increase in affinity, reaching *K*_i_ values of 0.282–0.210 µM for **PR 41–44** derivatives. Only the substitution at position *8-* **PR 45** resulted in a loss of affinity. Even more beneficial results were obtained by introducing modifications in the aryl region of 3,4-dihydroquinazoline. The most favorable substitution turned out to be in position *5-* **PR 65,58,63** or *8-* **PR 49**. In the last case, an affinity of 0.077 µM was obtained. Substitution with a chlorine atom turned out to be the most advantageous. By simultaneously replacing the 3,4-dihydroquinazoline fragment in position *5-* and the naphthalenesulfone fragment in position *2-* with chlorine atoms, no significant improvement in affinity was achieved in **PR 52**. The presence of sterically extended substituents resulted in an almost complete loss of activity regarding **PR 65–66.**

In the third set, it was observed that the substitution of 3,4-dihydroquinazoline at the fourth position with a methyl group is beneficial for 5-HT_6_R affinity. Therefore, observations from the third and fourth sets were combined to obtain the next set. The compounds belonging to the 4-methyl analogs of the previous group performed the most favorably (Table 5). 

Substituting the 3,4-dihydroquinazoline ring with chlorine or fluorine atoms in positions *5-* and *8-* resulted in the lowest values of the affinity *K*_i_ constant, reaching 0.037 µM for **PR 68**. Interestingly, substituting the nitrogen atom in the quinazoline ring with a methyl group **PR 76** resulted in an increase in affinity compared to the unsubstituted analog **PR 67**. Moreover, changing the core of 4-methyl-3,4-dihydroquinazoline to 4,5-dihydro-3*H*-1,3-benzodiazepine did not result in a loss of affinity, which opens up further possibilities for the synthesis of a library of 4,5-dihydro-3*H*-1,3-benzodiazepine derivatives. Importantly, as in the case of 3,4-dihydroquinazolin-2-amine derivatives tested for affinity to 5-HT_5A_R [28,29], substitution in the fourth position with a methyl group in *N*-(3,4-dihydroquinazolin-2-yl)naphthalene-1-sulfonamide seems to be crucial for the high affinity towards 5-HT_6_R, and the additional presence of the substituent at the fifth position is the most favored. Unlike arylsulfone derivatives of indole, in the compounds developed by us, the use of phenylsulfone did not lead to high affinity towards 5-HT_6_R. However, in the case of indole derivatives, the sulfone group was attached within the nitrogen atom built into the indole ring [21].

The four most active compounds and two structures deviating from the basic core were tested for selectivity in comparison to 5-HT_1A_, 5-HT_5A_, and 5-HT_7_ serotonin and dopamine D_2_ receptors, as well as their function being evaluated, based on the ability of a ligand to inhibit cAMP production induced by an agonist in HEK293 cells (Table 6). Importantly, all tested compounds showed unexpectedly high selectivity towards 5-HT_6_. All compounds, except **PR 76**, exhibited antagonist functions. The inversion towards the agonist of the mentioned compound seems to be extremely interesting.

### 2.3. Antiproliferative Activity

The antiproliferative activity of selected compounds was determined on several cancer cell lines. The assays were carried out on CNS tumors, such as an astrocytoma cell line—1321N1—and three glioblastoma cell lines, U87MG, LN-229, and U-251. In addition, the extended evaluation was performed on MCF7 breast and AsPC-1 pancreatic cancer cells. These cell lines represent the most commonly diagnosed female cancer and some of the most aggressive next to GBM. In our study, the results for these cancers were used comparatively. For one of the most active compounds on cancer cells, the selectivity against human normal astrocytes (NHAs) and normal brain cat (*Feline catus*) astrocyte cell line PG-4 was assessed. The results as IC_50_ values are depicted in Table 7. In order to determine the influence of affinity for the 5-HT_6_R on antiproliferative activity, we used a group of compounds with the highest activity (4 molecules with *K*_i_ < 0.1 µM); 17 compounds with medium affinity, including 4 with *K*_i_ (0.1, 0.2) µM, 6 with *K*_i_ (0.2, 0.3) µM, 5 with *K*_i_ (0.3, 0.4), and 2 with *K*_i_ (0.4, 0.5) nM; and 5 compounds with low affinity, *K*_i_ > 0.5 µM, and 3 with very low affinity below *K*_i_ > 9 µM.

Most of the tested compounds did not inhibit the viability of cancer cells. These compounds were classified as non-active, and their IC_50_ value was above 25 µM. Interestingly, in the case of all CNS tumors, only two compounds, **PR 64** and **PR 74**, significantly reduced cell viability. The **PR 74** showed moderate antiproliferative activity against 1321N1 and U87MG. The calculated IC_50_ values were 13.4 and 17 µM, respectively. In the case of **PR 64**, the high antiproliferative activity on U-251 cells was registered (IC_50_ = 7.7 µM). Moreover, this compound appeared to be active on MCF7 cells, where it showed slightly lower activity than on GBM cells. The studies on normal astrocytes revealed that **PR 64** was slightly less toxic. Despite this, its selectivity index (comparing the activity on NHA to U-251 cells) is very high, reaching 1.96. It is worth noting that for most cytostatic drugs, the therapeutic index (identical to SI, but based on clinical studies) does not exceed 0.3. 

Nevertheless, no correlation was found between affinity for the 5-HT_6_ receptor and biological activity in the tested astrocytoma and glioblastoma lines unlike the previously described research [14]. Thus, due to the high selectivity of the tested compounds towards the 5-HT_6_R and their low antiproliferative activity, they may find utility in the treatment of cognitive defects or other targets associated with this protein. Our hopes for the potential role of 5-HT_6_R antagonists in the treatment of glioma resulted from reports on the involvement of this protein in processes such as lethal autophagy [14] and mediating changes in the signaling of Cdk5 and mTOR kinases. The lack of the expected correlation between affinity for the 5-HT_6_R and antiproliferative activity may be related to the lack of receptor expression in the glioma cells used, as indicated by the data included in Proteinatlas [41]. It is advisable to evaluate the expression of these receptors in cell lines in further studies, or to use other lines with proven 5-HT_6_R expression, such as NCE-G 130.

Interestingly, Protein-atlas reports little expression of the 5-HT_6_ receptor in the AsPC-1 and MCF7 lines. However, no correlation was observed in their case either. The antiproliferative activity of **PR 64** and **PR 74** may result from activity towards other proteins. Using the SEA predictor [42], the probability of interactions with proteins such as an Endothelin-1 receptor and ATP-citrate synthase is predicted. In the case of both proteins, their expression was found in some glioblastoma multiforme lines and their role in inhibiting tumor development was postulated. However, this issue requires experimental investigation [43,44]. It should be noted that most of the strong or moderate 5-HT_6_R antagonists showed the ability to inhibit cancer cell viability at a concentration of 25 µM, with the observed decrease in viability being 20–50% compared to the vehicle. This effect is too weak to speak of strong anticancer activity, but in combination with neuroprotective activity, 5-HT_6_R antagonists may constitute a valuable component of combination therapies. In order to obtain compounds with increased anticancer activity, further development of the chemotype was planned, based on analogs of **PR 64** and **PR 74**, taking into account the introduction of additional, amine functional groups at the aromatic ring of quinazoline.

### 2.4. Assessment of Pharmacokinetic Properties and Toxicity

A series of ADMET tests were performed for the selected compounds [45]. We began with an assay for lipophilicity, affinity for phospholipids (for 40 compounds), and drug binding to human serum albumin (HSA) (for 20 compounds), using chromatographic protocols developed by Valko [46] and implemented in our laboratory [47]. Compounds with the highest affinity for 5-HT_6_R and those showing the most promising ADMET parameters, including the possibility of BBB penetration, DDI, and lipophilicity, determined in silico in SwissADME [45], were selected for testing. These methods allowed the determination of chromatographic hydrophobicity indices (CHIs), binding to HSA (%HSA), and phospholipids (CHI_IAM_) using a single-gradient elution experiment and comparison to reference substances. The obtained results showed that the analyzed molecules are generally characterized by relatively high, but in most cases acceptable, lipophilicity (>100). Only the **PR 74** has significantly higher lipophilicity (CHI_C18 at pH 7.4_ = 123).

Lipophilicity is a well-known factor determining passive diffusion across the blood–brain barrier (BBB). Considering the lipophilic properties of some CNS-targeting drugs, measured with the same protocols (Table S1), we can confirm that the synthesized molecules are very close to substances available on the market.

Overall, the target molecules also showed strong binding to phospholipids. It is worth noting that the structures did not exceed the value of 50, which is a value indicating the potential for promiscuous binding and affecting phospholipidosis [48]. A comparison of the CHI values at different pH levels allows for a quick assessment of acid–base properties, and the analyzed substances are usually low-basic. The exception is the **PR 11** compound, which is expected to be acidic. This fact may result from the presence of the benzimidazole moiety in the structure of the ligand, which consists of an acidic nitrogen atom. The use of columns modified with plasma proteins, such as HSA, also allows for the estimation of binding to HSA, which is the most dominant plasma protein (PP). Binding to PP affects drug pharmacokinetics, such as distribution, half-life, and clearance. Although the target substances have a high % HSA binding (Table 8), higher than investigated market-available CNS-targeting drugs, these results are still acceptable since we can find substances with a similar affinity to HSA used in clinical practice like pimozide (98.6%) [49]. 

More detailed ADMET tests were performed for **PR 68**, as the compound with the highest affinity for the 5-HT_6_R (Table 9). The assessment of interaction with PP for **PR 68** was also performed and confirmed in a study using the commercial TRANSIL^XL^ PPB test. The test mimicked physiological plasma conditions in which HSA and AGP were present in a 24:1 ratio. Warfarin was used as a positive control that was highly bound to plasma proteins. The determined dissociation constant (k_D_) was 1.4, which corresponds to 99.8 ± 0.13% binding to plasma proteins.

The permeability of the most potent compound, **PR 68**, was tested in PAMPA. This test allows the determination of the compound’s passive diffusion through biological membranes. The ligand has an accepted permeability coefficient (Pe = 1.72 × 10^−6^ cm/s) in comparison with the well-permeable reference caffeine (Pe = 8.23 × 10^−6^ cm/s) and according to the breakpoint (1.5 × 10^−6^) for permeable compounds. However, the compound showed high membrane retention (R = 0.65), which was expected here because of its relatively high lipophilicity.

The metabolic stability of **PR 68**, **PR 73**, and **PR 76** were determined in mouse liver microsomes (MLMs). Two compounds with the highest affinity for 5-HT_6_R and the only agonist in the obtained set were selected for testing. The UPLC analysis of the tested compound after 120 min of incubation with MLMs indicated that **PR 68** was metabolized in 49%. This result is an improvement over unsubstituted **PR 40**, which was metabolized in 97% as previously reported [30]. We identified nine possible metabolites. The results show that the most probable metabolic pathway is the hydroxylation at the quinazoline aromatic ring. Prediction using The MetaSite 6.0.1 [50] suggests that positions *6-* and *7-* in the quinazoline ring are the most susceptible to hydroxylation. Interestingly, the analog of **PR 68** with a fluorine substituent instead of chlorine at the *8-* position showed much lower metabolic stability (remaining 24.59%) and its metabolism occurred mainly through double-bound reduction, as in the unsubstituted analog [30]. **PR 76** with a methylated nitrogen atom in the quinozaline ring (remaining 87.40%) showed unexpectedly high stability.

**Figure 2:**
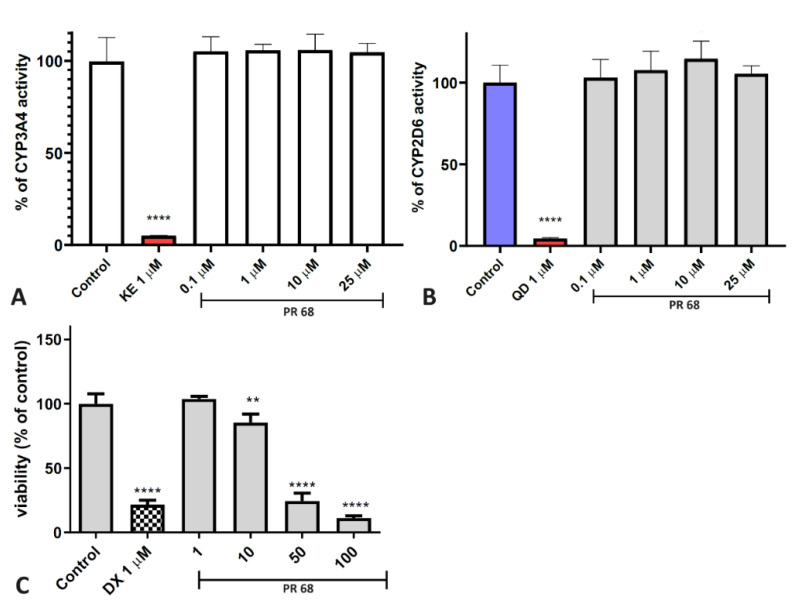
The influence of **PR 68** on (**A**) CYP3A4 activity and (**B**) CYP2D6 activity. Statistical significance (**** p < 0.0001), KE = reference inhibitor ketoconazole, QD = reference inhibitor quinidine. (**C**) The effect of cytostatic drug doxorubicin (DX) and **PR 68** on hepatoma HepG2 cell line viability after 48 h of incubation at 37 °C, 5% CO_2_. The statistical significance (GraphPad Prism 8.0.1) was evaluated by a one-way ANOVA, followed by Bonferroni’s comparison test (** p < 0.01 and **** p < 0.0001 compared with negative control DMSO 1% in growth media).

The potential risk of drug–drug interactions (DDIs) was examined in luminescence-based CYP3A4 and CYP2D6 P450-Glo assays (Promega, Madison, WI, USA) (Figure 2A,B). 

We found that **PR 68** should not exhibit drug–drug interactions with these isoforms. The safety profile was estimated in the hepatotoxicity assay in the hepatoma HepG2 cell line (Figure 2B). Compound **PR 68** did not exhibit hepatotoxic properties in the range concentration of 1–10 µM. The presented results suggest that the **PR 68** has pharmacokinetic properties acceptable for drugs acting on the CNS.

**Figure 3:**
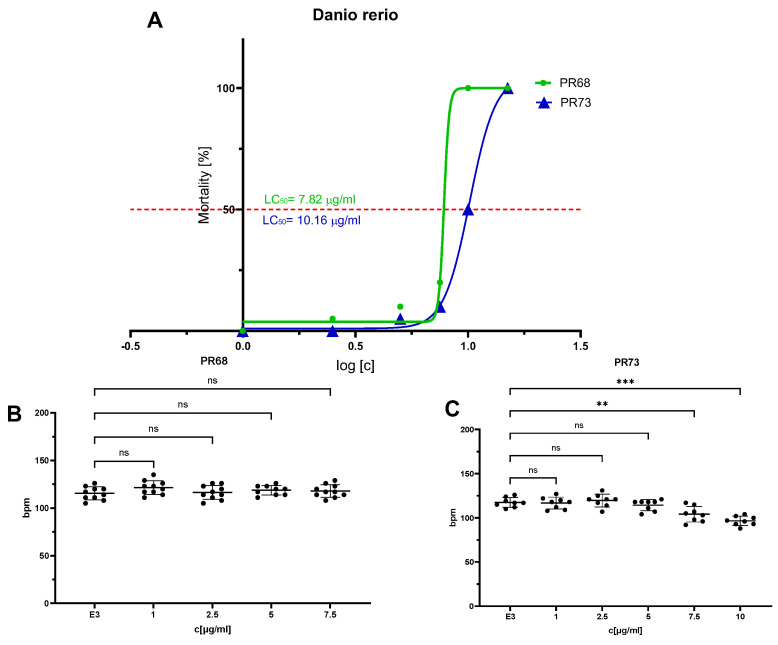
(**A**) Toxicity of **PR 68** and **PR 73** in vivo in *Danio reiro* embryo toxicity test. LC_50_ values calculated after 96 h of intoxication. Cardiotoxicity of (**B**) **PR 68** and (**C**) **PR 73** measured as heart rate (beats per minute) on 5 dpf *Danio rerio* larvae, E3—fish embryo growth medium. Statistical significance (GraphPad Prism 10.0.0) was evaluated by one-way ANOVA, followed by Dunnett’s Comparison Test (** *p* < 0.01 and *** *p* < 0.001 compared with negative control E3).

**Figure 4:**
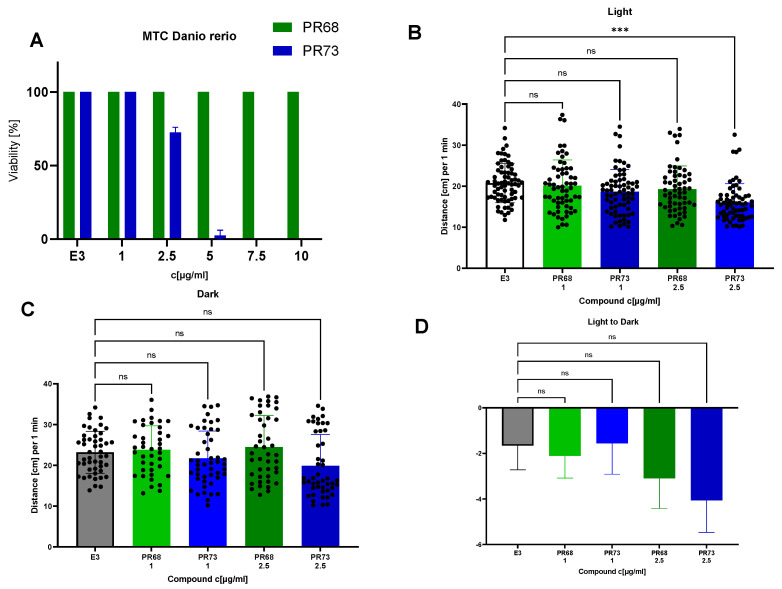
(**A**) The toxicity of **PR 68** and **PR 73** in vivo in the *Danio reiro* MTC toxicity test. Neurotoxicity evaluated as locomotor activity of **PR 68** and **PR 73** (**B**) in light conditions and measured as distance in cm per 1 min of activity of 5 dpf *Danio rerio* larvae, (**C**) in dark conditions and measured as distance in cm per 1 min of activity of 5 dpf *Danio rerio* larvae, (**D**) as the difference in activity in the transition through light to dark conditions; E3—fish embryo growth medium. The statistical significance (GraphPad Prism 10.0.0) was evaluated by a one-way ANOVA, followed by Tukey Comparison Test: *** *p* < 0.001 compared with negative control E3.

Selected compounds were evaluated for their cardiotoxicity on the *Danio rerio* experimental model following the OECD 236 test. The **PR 68** and **PR 73** do not show cardiotoxic properties in the nontoxic range, based on the heart rate measurement (Figure 3A–C). To explore specific toxic effects of tested compounds on the central nervous systems, locomotor activity in the *Danio rerio* experimental model was evaluated. Studying the reaction of zebrafish larvae to light and dark conditions can be a valuable method for assessing neurotoxicity in behavioral studies. Zebrafish larvae typically exhibit a startle response to sudden changes in their environment, including changes in light intensity. This response is characterized by a rapid, coordinated movement, often involving a quick burst of swimming or a change in direction. In our experimental model, we changed light into dark conditions. Zebrafish larvae have been shown to display light avoidance behavior, where they actively move away from brightly lit areas towards darker regions. This behavior helps them avoid potential predators and maintain optimal environmental conditions. Therefore, in our experimental design, we have observed increased locomotor activity in dark conditions, what proved to be low neurotoxic effects of examined compounds (Figure 4A–C).

### 2.5. Molecular Modeling 

To explain the result of SAR, the molecular modeling methods were used in the next part. First, the molecular docking of the crystal structures of 5-HT_6_R [51] was performed, using the basis of all obtained ligands. Selected complexes were optimized using the QM-Polarized Ligand Docking (QPLD) methodology [52]. The binding energy for the L-R complexes was calculated according to the MM-GBSA protocol [53] and their stabilization energies were estimated using the FMO-EDA method [54].

**Figure 5:**
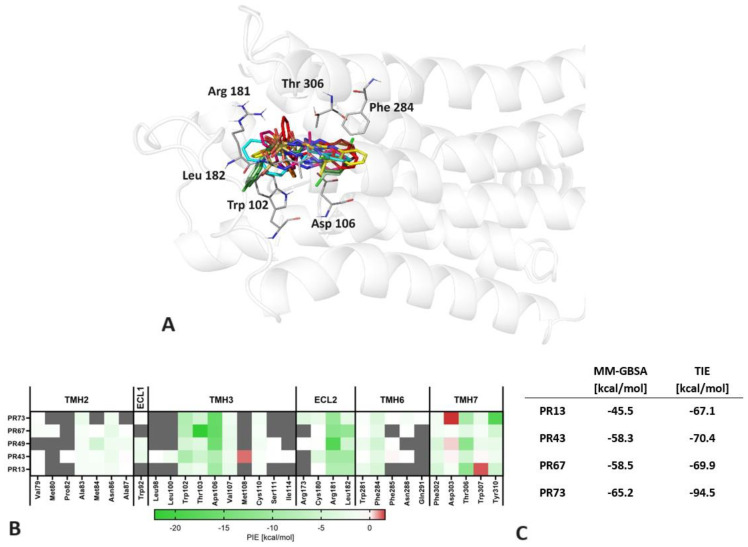
(**A**) Binding modes of **PR 13** (brown), **PR 40** (orange), **PR 41** (dark green), **PR 67** (pink), **PR 68** (cyan), **PR 71** (pale green), and **PR 76** (yellow) in 5-HT_6_R. (**B**) Calculated pair interaction energies (PIE; kcal/mol) between ligand and binding site residues of 5-HT_6_R. Data are presented as a heat map where strongly attractive interactions are marked as green, while strongly repulsive ones are red-colored. A gray square means none of the interactions. (**C**) The MM-GBSA binding energy calculated for L-R complexes and FMO total interaction energies (TIEs).

The analysis of the binding site was presented based on the active conformation of ligands, **PR 13** (brown), **PR 40** (orange), **PR 41** (dark green), **PR 67** (pink), **PR 68** (cyan), **PR 71** (pale green), and **PR 76** (yellow) (Figure 5A). 

Full data are provided in the Supplementary Materials. In the case of all complexes, interactions with Arg 181 and Leu 182 residues were observed. They were mostly hydrogen bonds with the SO_2_ group in the ligand structure, and in some cases also the π–cation. The π–π interactions with the Trp 102 in the arylsulfonamide region were equally frequently observed. In the case of naphthalene-1-sulfonamide derivatives, this bond was double, which may partially explain their increased affinity. An interesting fact is the relatively rare π–π interactions with Phe 284 and 285, which have been observed in the case of other groups of 5-HT_6_R ligands [21,55]. In no cases was a strong hydrogen bond with Asp 106 observed, considered crucial in the context of the high affinity of basic 5-HT_6_R ligands [55,56]. For derivatives with a chlorine atom in the *5-* or *8-* position of the quinazoline, halogen bonds with residues such as Asn 288 for **PR 68** or 103 for **PR 71** were distinguished.

To investigate the influence of the interactions of the ligand with individual residues on the binding energies, it was decided to analyze the pair interaction energy (PIE) according to the ab initio FMO methodology (Figure 5B) [53].

The results of the FMO calculations indicate that this ligand group forms strongly stabilizing interactions at three distinct sites within the binding pocket. Two are situated within helices 3 and 7, at the core of the binding site encompassing amino acid residues proximal to Asp 106 (D3.32). The deprotonation of ligands results in the absence of a salt bridge at this location, compensated for by a hydrogen bond network with the ligands’ amino moiety. The third set of amino acids strongly interacting with the ligands is located in the second extracellular loop. Such an energetic profile of L-R complexes is quite characteristic of aminergic receptor ligands. Interestingly and relatively unusually, a series of weak interactions with residues in helix 2 is observed. Additionally, the absence of interactions with residues in helix 5 (particularly S5.43) may suggest an antagonistic function of these compounds [57,58]. Interaction energies calculated by the FMO method (TIE in Figure 5C) correlate very well with experimental affinities (Ki).

**Figure 6:**
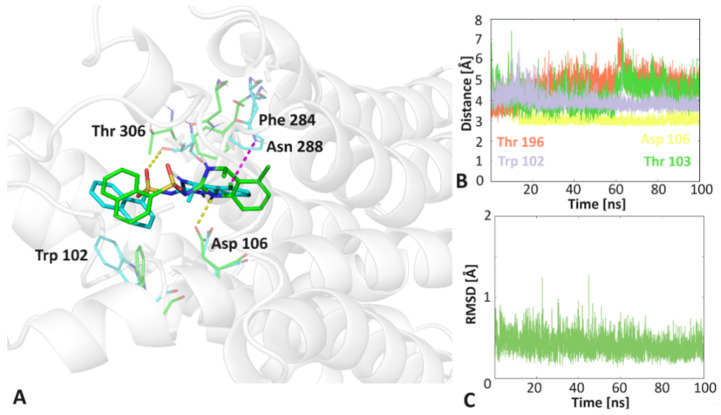
(**A**) Binding modes of **PR 68** (cyan) compared to the most representative pose (green) from molecular dynamics (clustering) into 5-HT_6_R. The yellow dash lines indicate the hydrogen bonds, and violet halogen bonds. Key residues are colored sticks. (**B**) The ligand–residue distance as a function of time. (**C**) RMSD of the ligand.

Next, for selected ligands (**PR 49**; **PR 68**; **PR 71**), the molecular dynamics simulations (100 ns) with the use of Charm++ in NAMD [59] were conducted, to confirm the stability of the complexes. The results were presented based on the example of the compound with the highest affinity, **PR 68**. It is worth noting that the poses were characterized by high stability, maintaining RMSD at the level of <2 Å. Interestingly, during the simulation, a slight rotation of the molecule was observed at the beginning towards helices 6 and 7, which resulted in their slight extension (up to 3 Å in the terminal part) towards helix 1, as seen in Figure 6A. 

The remaining helices did not change their position significantly. The change in the orientation of the ligand and the shift of important amino acid residues from helices 6 and 7 were associated with the appearance of a stable bond with Asp 106 (Figure 6B). The ligand also approached the Phe 284 residue, without losing contact with Trp 102. Importantly, halogen bonds with residues Ser 193 and Thr 196 periodically occurred in the complex.

Based on the obtained results of biological tests and docking, 3D-QSAR was performed, and a pharmacophore model was prepared. The dataset consisting of 83 compounds was randomly divided into a training set and a test set (50/50%). The training set was used to run various Gaussian field GFQSAR or atom-based (ABQSAR) 3D-QSAR models. The partial least squares (PLS) regression method was used to evaluate the robustness of the developed model during 3D QSAR model generation. A total of seven PLS coefficients were used to check the robustness and predictive power by calculating PLS statistics. The visualization of the best 3D-QSAR models was analyzed by recognizing colored regions highlighting preferred and unfavorable areas. For the best GFQSAR model, R^2^ 0.86 for the training set and R^2^ 0.69 for the test set were obtained, for ABQSAR R^2^ 0.92 for the training set and R^2^ 0.71 for the test set.

**Figure 7:**
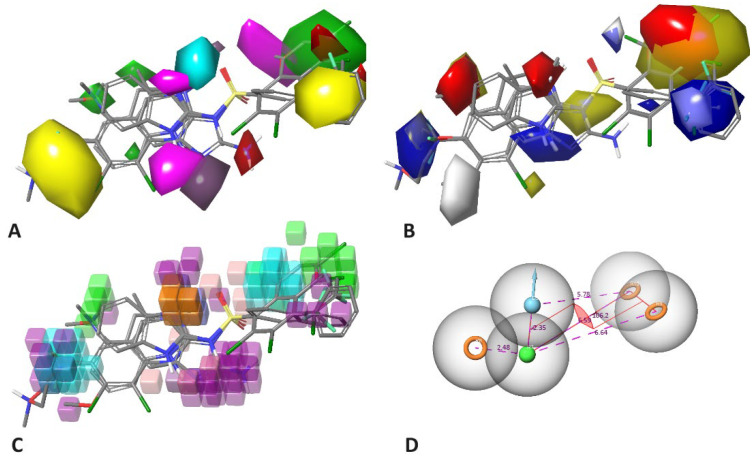
Contours obtained by GFQSAR: (**A**) steric (positive: green, negative: yellow); HBD (positive: violet, negative: cyan); HBA (positive: red, negative: magenta). (**B**) Electrostatic (positive: blue, negative: red); hydrophobic (positive: yellow, negative: white). (**C**) Contours obtained by ADQSAR: hydrophobic (positive: green, negative: purple); HBD (positive: blue, negative: orange); electron-withdrawing (positive: red, negative: cyan). (**D**) DHRRR pharmacophore model hypothesis consisting of an HBD (D5), three aromatic rings (R8, R9, R10), and a hydrophobic fragment H7 shown in cyan, orange, and green, respectively.

Based on the obtained model, it can be seen that the expansion of the quinazoline ring in positions *6-* and *7-* and the substitution of arylsulfonamide in positions *3*- and *4-* with respect to phenyl are sterically unfavorable (Figure 7A). The presence of a hydrogen bond donor (HBD) in the guanidine region (Figure 7A,C) is also important for affinity. The presence of an acceptor (HBA) is much less important, which is interesting in terms of building a pharmacophore model. The expansion of the arylsulfonamide ring in positions 2 and 3 relative to phenyl should be based on hydrophobic components.

In the next step, a pharmacophore model was developed based on the affinity of the tested ligands for the 5-HT_6_ receptor, assuming a pKi threshold of >= 6.7 for active compounds and <= 6.3 for inactive ones. Ten pharmacophore models were generated with a specific combination of pharmacophore variants whose main feature is DHRRR or AHRRR. DHRRR showed the highest match with the most active compounds, and was associated with and showed the best survival site score and selectivity score. Moreover, the 3D-QSAR analysis showed greater importance of HBD than HBA; therefore, the DHRRR mode was considered probably to best describe the obtained chemotype of ligands.

## 3. Materials and Methods

### 3.1. Chemical Synthesis

The ultrasound reactions were carried out in the Ultrasonic bath PS-08 (Jeken, Dongguan, China), with the ultrasound power at 80 W and a frequency of 40 kHz. The microwave-assisted synthesis was conducted in the CEM Discover microwave reactor. A thin-layer chromatography analysis (TLC) was performed on Sigma-Aldrich (St. Louis, MO, USA) sheets (silica gel on aluminum, with a fluorescent indicator at 254 nm, 200 µm layer thickness, 60 Å pore diameter, 8.0–12.0 µm particle size). The LC-MS system consisted of a Waters Acquity UPLC system coupled to a Waters TQD mass spectrometer (electrospray ionization mode ESI-tandem quadrupole). The analyses were carried out with an Acquity UPLC BEH C18, 1.7 µm, 2.1 × 100 mm column. IR spectra were taken on an FTS-165 spectrometer. NMR spectra were measured on a Bruker Avance 400 MHz spectrometer (TMS as an internal reference). The melting points were measured by a Boëtius apparatus. An elemental analysis was performed on the Vario EL II apparatus.

#### 3.1.1. Synthesis of Ligands

##### Method A

In a round-bottom flask, 1 mmol of benzimidazol-2-amine **1a**–**c** and 1 mmol of the corresponding aryl sulfonylchloride **2a**–**h** were placed and 3 mL of acetone was added. Reactions were carried out for 30 s in a CEM Discover microwave reactor at a power of 100 W. After this time, 20 mL of water was added to the reaction mixture and the resulting precipitate was filtered off, washing it with 5 mL of cold water. After drying, the crude products **PR1**–**9** and **12** were crystallized from methanol.

##### Method B

The 1 mmol of aryldiamine **3a**–**x**, 1 mmol dimethyl(arylsulfonyl)carbonodithioimidate **4a**–**c**, 0.415 g (2 mmol) K_2_CO_3_, 0.003 g (0.1 mmol) TBAB, and 5 mL of EtOH (**B^1^**) or 1.2 mL of H_2_O (**B^2^**) were mixed in a round-bottom flask. Reactions were carried out for 2 h in reflux (**B^1^**) or 5 min in a microwave reactor with a power of 50 W (**B^2^**). After this time, 5 mL of H_2_O was added, and the resulting precipitate was filtered off and rinsed in the funnel with 3 mL of cold H_2_O. The crude products were separated by column chromatography using CHCl_3_:MeOH 99:1–95:5 as an eluent.

##### Method C

The 1 mmol of 2-(methylsulfanyl)-1,4-dihydroquinazoline **5a**–**j** and 1 mmol of arylsulfonamide **6a**–**y** were mixed in a round-bottom flask. Then, 0.42 mL (3 mmol) of TEA (**C^1^**) or 0.138 g (1 mmol) K_2_CO_3_, 0.003 g (0.1 mmol) TBAB, and 0.2 mL of H_2_O (**C^2^**) were added. Reactions were carried out for 2 h in 180 °C (**C^1^**) or 30 s in a microwave reactor at a power of 50 W (**C^2^**). After this time, 20 mL of H_2_O was added to the mixture and extracted three times with 15 mL of CH_2_Cl_2_. After evaporating off the solvent, the obtained product was separated by column chromatography using CHCl_3_:MeOH 99:1–95:5 as an eluent.

#### 3.1.2. Synthesis of Diamines **3b–p**

Substituted 2-amino-benzonitrile **7a**–**v** (8.75 mmol) was transferred to an oven-dried 100 mL round-bottom flask and dissolved in dry THF (15 mL). The mixture was purged with nitrogen, and then, while cooling at 0 °C, an 8.75 mL borate solution (2.0 M in THF) was slowly added dropwise under a nitrogen atmosphere. The solution was stirred for 10 min at 0 °C and for another 5 h at 60 °C. The progress of the reaction was monitored by TLC. The reaction was stopped by adding H_2_O. After evaporating the THF, the mixture was extracted with CH_2_Cl_2_ and then separated on a CHCl_3_:MeOH 9:1 column. Yields in the range of 59–82% were achieved [27].

A total of 5.7 g of NaBH_4_ in 90 mL of anhydrous THF was transferred into a 250 mL round-bottom flask dried in an oven, and then 11.5 mL of CF_3_COOH in anhydrous THF was added dropwise while cooling with ice, maintaining the temperature <20 °C. Substituted 2-amino-benzonitrile **7a**–**v** (30 mmol) in 20 mL THF was then added dropwise. The whole was stirred at room temperature for 4 h. The progress of the reaction was monitored by TLC. The reaction was stopped by adding water. After evaporating the THF, the mixture was extracted with CH_2_Cl_2_ and then separated on a CHCl_3_:MeOH 9:1 column. Yields in the range of 12–61% were achieved [60].

#### 3.1.3. Synthesis of 3,4-Dihydroquinazoline-2(1H)-thione Derivatives **8a**–**b**

A total of 24 mmol of substituted 2-(aminomethyl)aniline **3b,d** dissolved in 60 mL of EtOH was transferred into a 100 mL flask and 12 mL of CS_2_ was added at room temperature. The mixture was stirred at 55 °C for 12 h. After this time, the reaction was cooled, and the obtained solid was filtered off and washed with Et_2_O. The crude product **8a**–**b** was crystallized from methanol to give a yield of 60–78% [34].

#### 3.1.4. Synthesis of Substituted 1-(2-Aminophenyl)ethan-1-one **9b–h**

To an oven-dried 250 mL round-bottom flask, 56 mL of methylmagnesium bromide (3 M in Et_2_O, 170 mmol) was added and the mixture was purged with nitrogen. A suspension of substituted 2-amino-benzonitrile **9a**–**h** (24 mmol) in 60 mL of Et_2_O was added portion-wise to an ice-cooled flask. The mixture was then heated to reflux for 5 h, monitoring the reaction progress by TLC. The mixture was then placed in an ice bath and 58 mL of HCl (6 M) was slowly added. The mixture was heated at reflux of Et_2_O for 6 h, then cooled and made basic by adding solid Na_2_CO_3_. The residue was extracted several times with EtOAc, the combined organic layers were dried (Na_2_SO_4_), and the solvent was evaporated under reduced pressure. The crude product **8c**–**j** was purified by crystallization from hexane. Yields in the range of 43–87% were achieved [28,29].

#### 3.1.5. Synthesis of Substituted 3,4-Dihydroquinazoline-2(1H)-thiones **8c–j**

A total of 0.411 g of NaBH_4_ (10.9 mmol) and 10 mL of EtOH were mixed in a 100 mL flask. After heating to 65 °C, substituted 1-(2-amino-phenyl)ethanone **9b**–**h** (18.1 mmol) was added and the mixture was heated for 12 h (65 °C). Then, 8 mL of water, 1.93 g of KSCN in 4 mL of H_2_O, and 7 mL of 20% HCl were added. The mixture was reheated for 3 h at 65 °C. After cooling, the crude product **8c-j** was filtered off and crystallized from methanol. Yields in the range of 64–75% were achieved [28,29].

#### 3.1.6. Synthesis of Substituted 2-(Methylsulfanyl)-3,4-dihydroquinazoline **5a–j**

Substituted 3,4-dihydro-1*H*-quinazoline-2-thion **8a**–**j** (6.2 mmol), methyl iodide (1.16 mL, 19 mmol), and 15 mL acetone (15 mL) were mixed in a round-bottom flask and stirred at room temperature for 12 h. The precipitated product was filtered off and used for the next step. Yields in the range of 88–96% were achieved [28,29].

#### 3.1.7. Synthesis of 2-[1-(Methylamino)ethyl]aniline **3w**

A total of 2.25 g of 2-amio-acetophenone **9a** (16.6 mmol), 8 mL of EtOH, and 3 g of molecular sieves (3 Å) were mixed in a round-bottom flask; then, the mixture was purged with nitrogen. Next, 4.4 mL of a solution of methylamine in EtOH (33 wt%) was added under an inert gas atmosphere. The mixture was stirred at room temperature for 48 h. The content of the flask was then filtered through celite and washed with dichloromethane. The solvent was removed in vacuo to give a yellow oil, which was dissolved in MeOH (35 mL). The mixture was cooled to <0 °C and 3.1 g of NaBH_4_ (80.8 mmol) was slowly added in portions over 2 h to maintain the temperature <10 °C. After the addition was complete, the reaction mixture was warmed to room temperature and stirred for 24 h. After this time, the mixture was acidified with HCl and then concentrated in vacuo. The precipitate was dissolved in an aqueous Na_2_CO_3_ solution and then extracted with CH_2_Cl_2_. The organic layer was dried over anhydrous MgSO_4_ and filtered and the solvent was removed in vacuo to give a yellow oil. The crude product **3w** was purified by column chromatography (CHCl_3_:MeOH 9:1), with a yield of 34% [35].

#### 3.1.8. Synthesis of 1-(2-Aminophenyl)ethan-1-ol **3x**

A total of 0.822 g of NaBH_4_ (21.8 mmol) and 10 mL of EtOH were mixed in a 100 mL flask. After heating to 65 °C, 1-(2-amino-phenyl)ethanone **9a** (18.1 mmol) was added and the mixture was heated for 12 h (65 °C). Then, 20 mL of H_2_O was added, and the mixture was extracted by CH_2_Cl_2_. The organic layer was dried over anhydrous MgSO_4_ and filtered and the solvent was removed in vacuo.

#### 3.1.9. Synthesis of Substituted N^1^-Substituted Nitrobenzonitrile **10a–d**

A total of 5 g of 6-Fluoro-2-nitrophenylamine or 5-Fluoro-2-nitrophenylamine (31.7 mmol) was dissolved in 10 mL of anhydrous DMF. Next, 9.7 mL of TEA (70 mmol) and amine (dimethylamine or morfoline) (35 mmol) were added. The mixture was stirred for 10 h. After this time, a yellow precipitate formed, which was collected by filtration, washed with Et_2_O, and dried with a yield of 87% [36].

#### 3.1.10. Synthesis of N-Substituted 2-Aminobenzonitriles **7s–v**

A total of 17.4 g of granulated Sn (0.15 mol) in 38 mL of concentrated HCl was prepared in a round-bottomed flask and heated to 45 °C. Substituted 2-nitrobenzonitrile **11a–d** (18 mmol) was added in one portion and the mixture was stirred at 55 °C for 20 min. The solution was decanted from the unreacted Sn and basified with a 50% sodium hydroxide solution while cooling with ice. The creamy precipitate was filtered off, washed with water, and crystallized from ethanol. Yields in the range of 66–69% were achieved [37].

#### 3.1.11. Synthesis of Chloronaphthalene Sulfonic Acid **13a–f**

In a round-bottomed flask, 10 g of aminonaphthalene sulfonic acids (44.8 mmol) were dissolved in 55 mL of a 0.85 M NaOH solution. Then, 25 mL of concentrated HCl was added to the mixture and the mixture was cooled to −5 °C. While stirring, a solution of 3.45 g of NaNO_2_ in 6.75 mL of H_2_O was added dropwise, keeping the temperature below 5 °C. After the dropwise addition, the suspension was stirred for 30 min at a temperature below 5 °C, and then 0.35 g of urea was added. The contents of the flask were transferred to a solution of 4.5 g of CuCl in 36 mL of concentrated HCl at a temperature of 80 °C. The resulting suspension was stirred at 80 °C until a clear solution was obtained, which was then concentrated to a volume of approximately 40 mL. After cooling, the resulting precipitate of chloronaphthalene sulfonic acid **11a-f** was filtered off. After drying, it was dissolved hot in 100 mL of H_2_O and then made alkaline with solid Na_2_CO_3_. After reaching room temperature, the precipitated copper salts were filtered off and the filtrate was heated again. While hot, 3 g of NaCl was added and the solution was cooled to 5 °C. Then, a precipitate of chloronaphthalene sulfonic acid sodium salt was formed, Ref. [38]. Yields in the range of 21–57% were achieved.

#### 3.1.12. Synthesis of Chloro-Substituted Derivatives of Naphthalenesulfonyl Chlorides **2v–za**

The 5 g of chloronaphthalene sulfonic acid sodium salt and 10 mL of POCl_3_ were mixed in a round-bottom flask. The mixture was heated at 100 °C for 4 h. After cooling to room temperature, the mixture was poured into ice and, after the decomposition of unreacted POCl_3_, it was extracted with CH_2_Cl_2_. The organic phase was dried over MgSO_4_ and then evaporated. The residue was crystallized from hexane. Yields in the range of 61–81% were achieved [39].

#### 3.1.13. Synthesis of Chloronaphthalene Sulfonamides **6r–v**

Chloronaphthalene sulfonyl chloride **2v–za v** (21.9 mmol) was dissolved in 200 mL of acetone and 240 mL of NH_4_OH was added at 0 °C for 30 min and stirred at room temperature for 3 h. Acetone was removed at reduced pressure. The formed precipitate was collected by filtration, washed with H_2_O, and dried. Yields in the range of 67–95% were achieved [40].

#### 3.1.14. Synthesis of Dimethyl (Arylsulfonyl)carbonodithioimidate **4a–c**

Arylsulfonamide **6a,p,w** (22 mmol) and 2.2 mL of CS_2_ (36 mol) were dissolved in 30 mL of DMF and the mixture was cooled in an ice bath. A solution of 2.9 g of KOH (52 mmol) in 10 mL H_2_O was added dropwise at a temperature below 10 °C. The mixture was stirred for 30 min at 5–10 °C. Subsequently, 3.2 mL of CH_3_I (51 mmol) was added dropwise at such a rate that the temperature was kept below 10 °C. Then, the mixture was allowed to warm to room temperature and stirred for another 30 min. After this time, 25 mL was added, and the formed white precipitate was filtered off and washed with water followed by EtOH. Yields in the range of 54–84% were achieved [27].

1-(benzenesulfonyl)-1*H*-1,3-benzodiazol-2-amine PR1 

Prepared in method **A**, described in [30].

3-[(2-amino-1*H*-1,3-benzodiazol-1-yl)sulfonyl]benzonitrile PR2

Prepared in method **A**, described in [30].

1-(naphthalene-1-sulfonyl)-1*H*-1,3-benzodiazol-2-amine PR3

Prepared in method **A**, described in [30].

1-(naphthalene-2-sulfonyl)-1*H*-1,3-benzodiazol-2-amine PR4

Prepared in method **A**, described in [30].

5-[(2-amino-1*H*-1,3-benzodiazol-1-yl)sulfonyl]-2,3-dihydro-1*H*-indol-2-one PR5

Prepared in method **A**, described in [30].

5-chloro-1-(naphthalene-1-sulfonyl)-1*H*-1,3-benzodiazol-2-amine PR6

Prepared in method **A**, described in [30].

1-[(5-fluoro-3-methyl-1-benzothiophen-2-yl)sulfonyl]-1*H*-1,3-benzodiazol-2-amine PR7

Prepared in method **A**, described in [31].

**1-(1-benzothiophene-3-sulfonyl)-1H-1,3-benzodiazol-2-amine PR8**

Prepared in method **A**, Formula weight for C_15_H_11_N_3_O_2_S_2_: 329.4 g/mol, UPLC-MS: [M + H]^+^ = 330.2, purity = 100%, R_t_ = 5.81 min, Y = 62%, mp = 192–195 °C. Composition: C(54.70%), H(3.37%), N(12.76%), O(9.71%), S(19.47%), found: C(54.62%), H(3.39%), N(12.66%), S(19.45%).^1^H NMR (400 MHz, DMSO) δ 9.27 (bp, 1H, NH), 8.22–8.11 (m, 2H, ArH), 7.62 (d, *J* = 7.9 Hz, 1H, ArH), 7.54–7.46 (m, 2H, ArH), 7.29 (bp, 2H, ArH, NH), 7.17–7.07 (m, 2H, ArH), 7.05–6.98 (m, 1H, ArH). ^13^C NMR (101 MHz, DMSO) δ 152.41 (C_Gua_), 143.02 (ArC), 141.63 (ArC), 140.26 (ArC), 132.77 (ArC), 130.32 (ArC), 129.45 (ArC), 126.76 (ArC), 126.66 (ArC), 125.09 (ArC), 124.44 (ArC), 122.29 (ArC), 120.91 (ArC), 116.50 (ArC), 112.49 (ArC). FT IR: 3437, 3306, 3256 (N-H, Str), 3100, 3060 (C-H Ar, Str), 3003 (C-H Aliph, Str), 1691, 1657, 1611 (C=N, Str), 1561 (N-H, Bend), 1489, 1459, 1422, 1364 (S=O, -SO_2_, Str), 1297, 1258 (C-N Ar, Str), 1225 (C-N, Str), 1168, 1147, 1124 (S=O, sulfonamide, Str), 1110, 1056, 1017 (C-N, Str), 971, 944, 922, 896 (C=C Ar, Bend), 852, 809, 754, 738, 730, 710, 688, 655, 612 (C-F, Str).

**1-(quinoline-8-sulfonyl)-1H-1,3-benzodiazol-2-amine PR9**

Prepared in method **A**, Formula weight for C_16_H_13_N_4_O_2_S: 324.4 g/mol, UPLC-MS: [M + H]^+^ = 325.2, purity = 93%, Y = 71%, R_t_ = 4.49 min, mp = 254–256 °C. ^1^H NMR (400 MHz, DMSO) δ 8.92 (dd, J = 4.1, 1.5 Hz, 1H, ArH), 8.77 (d, J = 6.6 Hz, 1H, ArH), 8.53 (dd, J = 8.3, 1.4 Hz, 1H, ArH), 8.42 (d, J = 7.7 Hz, 1H, ArH), 7.88 (t, J = 7.8 Hz, 1H, ArH), 7.69 (dd, J = 8.3, 4.2 Hz, 1H, ArH), 7.35 (d, J = 7.9 Hz, 1H, ArH), 7.17–7.06 (m, 3H, NH, ArH), 7.01 (t, J = 7.3 Hz, 1H, ArH), 6.87 (t, J = 7.7 Hz, 1H, ArH). ^13^C NMR (101 MHz, DMSO) δ 154.27 (C_Gua_), 152.24 (ArC), 143.13 (ArC), 142.72 (ArC), 137.76 (ArC), 136.74 (ArC), 133.89 (ArC), 133.55 (ArC), 130.58 (ArC), 128.96 (ArC), 126.27 (ArC), 124.46 (ArC), 123.62 (ArC), 120.43 (ArC), 116.20 (ArC), 112.09 (ArC). FT IR: 3415, 3298, 3136 (N-H, Str), 1652, 1615 (C=N, Str), 1594, 1557 (N-H, Bend), 1380, 1354 (S=O, -SO_2_, Str), 1289, 1252 (C-N Ar, Str), 1217, 1209 (C-N, Str), 1170, 1146 (S=O, sulfonamide, Str), 1118, 1106, 1088, 1042, 1001 (C-N, Str), 969, 929, 893 (C=C Ar, Bend).

***N*** **-(1*H*-1,3-benzodiazol-2-yl)benzenesulfonamide PR10**

Prepared in method **B^2^**, described in [30].

***N*** **-(1*H*-1,3-benzodiazol-2-yl)naphthalene-1-sulfonamide PR11**

Prepared in method **B^1^**, Formula weight for C_17_H_13_N_3_O_2_S: 323.4 g/mol, UPLC-MS: [M + H]^+^ = 324.2, purity = 99%, R_t_ = 6.13 min, Y = 76%, mp = >315 °C. Composition: C(63.14%) H(4.05%) N(12.99%) O(9.90%) S(9.91%), found: C(63.01%) H(4.07%) N(12.92%) S(9.93%),^1^H NMR (400 MHz, DMSO) δ 11.97 (bp, 2H, NH), 8.83 (d, *J* = 8.6 Hz, 1H, ArH), 8.32 (dd, *J* = 7.2, 1.1 Hz, 1H, ArH), 8.13 (d, *J* = 8.3 Hz, 1H, ArH), 8.03 (d, *J* = 8.0 Hz, 1H, ArH), 7.69 (ddd, *J* = 8.5, 6.9, 1.4 Hz, 1H, ArH), 7.64–7.57 (m, 2H, ArH), 7.27–7.22 (m, 2H, ArH), 7.14–7.09 (m, 2H, ArH). ^13^C NMR (101 MHz, DMSO) δ 150.21 (C_Gua_), 139.35 (ArC), 134.28 (ArC), 133.07 (ArC), 129.79 (2ArC), 128.99 (ArC), 128.39 (ArC), 127.66 (ArC), 126.99 (ArC), 126.75 (ArC), 126.47 (ArC), 124.85 (ArC), 122.94 (2ArC), 111.37 (2ArC). FT IR: 3310 (N-H, Str), 3061 (C-H Ar, Str), 2970, 2924, 2884 (C-H Aliph, Str), 1738 (C-H Ar, Bend), 1630 (C=N, Str), 1599 (N-H, Bend), 1505, 1474 (-CH_2_, Bend) 1365 (S=O, -SO_2_, Str), 1308, 1278 (C-N Ar, Str), 1229, 1201 (C-N, Str), 1167, 1152 (S=O, sulfonamide, Str), 1142, 1122, 1100, 1061, 1026 (C-N, Str), 980, 929, 920 (C=C Ar, Bend).

***N*** **-methyl-*N*-(1-methyl-1*H*-1,3-benzodiazol-2-yl)naphthalene-2-sulfonamide PR12**

Prepared in method **B^1^**, described in [31].

***N*-(3,4-dihydroquinazolin-2-yl)benzenesulfonamide PR13**

Prepared in method **B^2^**, described in [31].

***N-*(3,4-dihydroquinazolin-2-yl)-4-fluorobenzene-1-sulfonamide PR14**

Prepared in method **C^1^**, Formula weight for C_14_H_12_FN_3_O_2_S: 305.3 g/mol, UPLC-MS: [M + H]^+^ = 306.2, purity = 100%, Y = 43%, R_t_ = 5.24 min, mp = 215–216 °C. ^1^H NMR (400 MHz, DMSO) δ 10.10 (bp, 1H, NH), 7.99 (bp, 1H, NH), 7.95–7.87 (m, 2H, ArH), 7.41–7.31 (m, 2H, ArH), 7.17 (t, *J* = 7.7 Hz, 1H, ArH), 7.12 (d, *J* = 7.4 Hz, 1H, ArH), 6.98 (td, *J* = 7.5, 1.0 Hz, 1H, ArH), 6.88 (d, *J* = 7.8 Hz, 1H, ArH), 4.45 (s, 2H, AlH). ^13^C NMR (101 MHz, DMSO) δ 164.03 (d, J = 249.2 Hz, C-F), δ 152.57 (C_Gua_), 140.96 (ArC), 140.93 (ArC), 135.42 (ArC), 129.00 (ArC), 128.90 (ArC), 128.57 (ArC), 126.33 (ArC), 123.65 (ArC), 118.47 (ArC), 116.58 (d, J = 8.3 Hz, C-F), 116.43 (ArC), 116.2 (ArC)1, 115.19 (d, J = 23.0 Hz, C-F), 42.57 (AlC). FT IR: 3319, 3252, 3159 (N-H, Str), 3065, 3050 (C-H Ar, Str), 2962 (C-H Aliph, Str), 1624 (C=N, Str), 1588, 1532 (N-H, Bend), 1493, 1468 (-CH_2_, Bend) 1406, 1361, 1337 (S=O, -SO_2_, Str), 1302, 1283, 1261 (C-N Ar, Str), 1226, 1208, 1171 (C-N, Str), 1151 (S=O, sulfonamide, Str), 1125, 1097, 1077, 1019 (C-N, Str), 960, 940, 929, 880 (C=C Ar, Bend), 844, 812, 791, 746, 712, 694, 664, 613 (C-F, Str).

***N*** **-(3,4-dihydroquinazolin-2-yl)-3,4-difluorobenzene-1-sulfonamide PR15**

Prepared in method **C^1^**, described in [31].

***N*** **-(3,4-dihydroquinazolin-2-yl)-4-(trifluoromethyl)benzene-1-sulfonamide PR16**

Prepared in method **C^1^**, described in [31].

***N*** **-(3,4-dihydroquinazolin-2-yl)-4-(trifluoromethoxy)benzene-1-sulfonamide PR17**

Prepared in method **C^1^**, Formula weight for C_15_H_12_F_3_N_3_O_3_S: 371.3 g/mol, UPLC-MS: [M + H]^+^ = 372.3, purity = 100%, R_t_ = 6.35 min, Y = 18%, mp = 161–163 °C.^1^H NMR (400 MHz, DMSO) δ 10.13 (bp, 1H, NH), 8.06–7.92 (m, 3H, ArH, NH), 7.52 (t, *J* = 9.0 Hz, 2H, ArH), 7.17 (t, *J* = 7.7 Hz, 1H, ArH), 7.13 (d, *J* = 7.4 Hz, 1H, ArH), 6.99 (td, *J* = 7.5, 0.9 Hz, 1H, ArH), 6.89 (d, *J* = 7.9 Hz, 1H, ArH), 4.46 (s, 2H, AlH). ^13^C NMR (101 MHz, DMSO) δ 152.59 (C_Gua_), 150.58 (ArC), 143.56 (ArC), 135.34 (ArC), 128.58 (2ArC), 126.35 (ArC), 123.73 (ArC), 121.68 (2ArC), 119.08 (ArC), 118.45 (ArC), 115.18 (ArC), 42.60 (AlC). FT IR: 3303, 3246, 3159, 3103 (N-H, Str), 3028 (C-H Ar, Str), 2962, 2840 (C-H Aliph, Str), 1630 (C=N, Str), 1536 (N-H, Bend), 1490, 1458, 1445 (-CH_2_, Bend) 1403, 1366, 1337 (S=O, -SO_2_, Str), 1259 (C-F, Str), 1200 (C-N, Str), 1152 (S=O, sulfonamide, Str), 1128, 1098, 1079, 1038, 1015 (C-N, Str), 973, 936, 875, 850 (C=C Ar, Bend), 799, 775, 747, 730, 715, 702, 665, 620, 605 (C-F, Str).

**2-chloro-*N*-(3,4-dihydroquinazolin-2-yl)benzene-1-sulfonamide PR18**

Prepared in method **C^1^**, Formula weight for C_14_H_12_ClN_3_O_2_S: 321.8 g/mol, UPLC-MS: [M + H]^+^ = 322.1, purity = 89%, R_t_ = 6.16 min, Y =65%, mp = 217–222 °C.^1^H NMR (400 MHz, DMSO) δ 10.13 (bp, 1H, NH), 8.07 (dd, *J* = 7.8, 1.6 Hz, 1H, ArH), 8.00 (bp, 1H, NH), 7.65–7.54 (m, 3H, ArH), 7.24–7.09 (m, 2H, ArH), 7.00 (t, *J* = 7.0 Hz, 1H, ArH), 6.92 (d, *J* = 7.8 Hz, 1H, ArH), 4.47 (s, 2H, AlH).^13^C NMR (101 MHz, DMSO) δ 153.05 (C_Gua_), 141.56 (ArC), 135.44 (ArC), 133.42 (ArC), 131.95 (ArC), 131.27v, 129.48 (ArC), 128.60 (ArC), 127.73 (ArC), 126.37 (ArC), 123.72 (ArC), 118.61 (ArC), 115.21 (ArC), 42.50 (AlC). FT IR: 3318, 3272, 3186 (N-H, Str), 3093, 3045 (C-H Ar, Str), 2917, 2849 (C-H Aliph, Str), 1728 (C-H Ar, Bend), 1641 (C=N, Str), 1577, 1541 (N-H, Bend), 1508, 1496, 1451, 1428 (-CH_2_, Bend) 1371, 1340 (S=O, -SO_2_, Str), 1290, 1281, 1249 (C-N Ar, Str), 1212 (C-N, Str), 1154 (S=O, sulfonamide, Str), 1138, 1120, 1096, 1041 (C-N, Str), 971, 936 (C=C Ar, Bend), 797, 740, 713, 692, 668 (C-Cl, Str).

**3-chloro-*N*-(3,4-dihydroquinazolin-2-yl)benzene-1-sulfonamide PR19**

Prepared in method **C^1^**, Formula weight for C_14_H_12_ClN_3_O_2_S: 321.8 g/mol, UPLC-MS: [M + H]^+^ = 322.1, purity = 99%, R_t_ = 6.43 min, Y = 43%, mp = 176–180 °C.^1^H NMR (400 MHz, DMSO) δ 10.15 (bp, 1H, NH), 8.02 (bp, 1H, NH), 7.88 (t, *J* = 1.8 Hz, 1H, ArH), 7.84–7.78 (m, 1H, ArH), 7.68–7.62 (m, 1H, ArH), 7.58 (t, *J* = 7.9 Hz, 1H, ArH), 7.21–7.14 (m, 1H, ArH), 7.13 (d, *J* = 7.4 Hz, 1H, ArH), 6.99 (td, *J* = 7.5, 1.0 Hz, 1H, ArH), 6.89 (d, *J* = 7.9 Hz, 1H, ArH), 4.46 (s, 2H, AlH).^13^C NMR (101 MHz, DMSO) δ 152.58 (C_Gua_), 146.34 (ArC), 135.31 (ArC), 133.87 (ArC), 131.91 (ArC), 131.46 (ArC), 128.58 (ArC), 126.36 (ArC), 125.85 (ArC), 124.81 (ArC), 123.76 (ArC), 118.44 (ArC), 115.19 (ArC), 42.62 (AlC). FT IR: 3309, 3168 (N-H, Str), 3083, 3063 (C-H Ar, Str), 2959, 2920, 2857 (C-H Aliph, Str), 1728 (C-H Ar, Bend), 1628 (C=N, Str), 1579, 1540 (N-H, Bend), 1504, 1492, 1463, 1445 (-CH_2_, Bend) 1374, 1344, 1332 (S=O, -SO_2_, Str), 1265 (C-N Ar, Str), 1212 (C-N, Str), 1154 (S=O, sulfonamide, Str), 1132, 1099, 1082, 1039 (C-N, Str), 992, 975, 933 (C=C Ar, Bend), 841, 790, 746, 676 (C-Cl, Str).

**2,3-dichloro-*N*-(3,4-dihydroquinazolin-2-yl)benzene-1-sulfonamide PR20**

Prepared in method **C^2^**, Formula weight for C_14_H_11_Cl_2_N_3_O_2_S: 356.2 g/mol, UPLC-MS: [M + H]^+^ = 356.1, purity = 97%, R_t_ = 7.16 min, Y = 71%. ^1^H NMR (400 MHz, DMSO) δ 10.17 (bp, 1H, NH), 8.05 (s, 2H, ArH), 7.86 (s, 1H, ArH), 7.54 (bp, 1H, NH), 7.17 (d, J = 22.1 Hz, 2H, ArH), 6.98 (d, J = 33.3 Hz, 2H, ArH), 4.49 (s, 2H, AlH). ^13^C NMR (101 MHz, DMSO) δ 153.05 (C_Gua_), 143.78 (ArC), 135.32 (ArC), 134.29 (ArC), 133.79 (ArC), 129.49 (ArC), 128.68 (ArC), 128.62 (ArC), 128.24 (ArC), 126.39 (2ArC), 123.85 (ArC), 118.59 (ArC), 115.28 (ArC), 42.56 (AlC).

***N*** **-(3,4-dihydroquinazolin-2-yl)-3,4-dichlorobenzene-1-sulfonamide PR21**

Prepared in method **C^1^**, Formula weight for C_14_H_11_Cl_2_N_3_O_2_S: 356.2 g/mol, UPLC-MS: [M + H]^+^ = 356.2, purity = 100%, R_t_ = 6.48 min, Y = 41%, mp = 192–193 °C. ^1^H NMR (400 MHz, DMSO) δ 10.17 (bp, 1H, NH), 8.06 (d, *J* = 1.8 Hz, 1H, ArH), 8.01 (bp, 1H, NH), 7.85–7.77 (m, 2H, ArH), 7.18 (t, *J* = 7.7 Hz, 1H, ArH), 7.13 (d, *J* = 7.4 Hz, 1H, ArH), 6.99 (t, *J* = 7.0 Hz, 1H, ArH), 6.89 (d, *J* = 7.9 Hz, 1H, ArH), 4.45 (s, 2H, AlH). ^13^C NMR (101 MHz, DMSO) δ 152.55 (C_Gua_), 144.75 (ArC), 135.23 (ArC), 134.87 (ArC), 132.09 (ArC), 131.85 (ArC), 128.60 (ArC), 128.04 (ArC), 126.38 (ArC), 123.83 (ArC), 118.43 (ArC), 115.22 (ArC), 42.64 (AlC). FT IR: 3335, 3267, 3220 (N-H, Str), 3083 (C-H Ar, Str), 2962, 2939, 2854 (C-H Aliph, Str), 1699 (C-H Ar, Bend), 1615 (C=N, Str), 1541 (N-H, Bend), 1509, 1494, 1477, 1452, 1433, 1412 (-CH_2_, Bend), 1366, 1330 (S=O, -SO_2_, Str), 1288, 1268, 1253, 1241 (C-N Ar, Str), 1213, 1140, 1116, 1075, 1030, 1007 (C-N, Str), 987, 948, 935 (C=C Ar, Bend), 888, 876, 847, 810, 767, 753, 713, 703, 674, 643 (C-Cl, Str).

**2,6-dichloro-*N*-(3,4-dihydroquinazolin-2-yl)benzene-1-sulfonamide PR22**

Prepared in method **C^1^**, Formula weight for C_14_H_11_Cl_2_N_3_O_2_S: 356.2 g/mol, UPLC-MS: [M + H]^+^ = 356.1, purity = 100%, R_t_ = 6.82 min, Y = 65%, mp = 168–174 °C. ^1^H NMR (400 MHz, DMSO) δ 10.14 (bp, 1H, NH), 8.02 (bp, 1H, NH), 7.63–7.60 (m, 2H, ArH), 7.19 (t, J = 7.6 Hz, 1H, ArH), 7.15 (d, J = 7.4 Hz, 1H, ArH), 7.02 (td, J = 7.5, 1.0 Hz, 1H, ArH), 6.92 (d, J = 7.9 Hz, 1H, ArH), 4.50 (s, 2H, AlH). ^13^C NMR (101 MHz, DMSO) δ 152.76(C_Gua_), 139.01 (ArC), 135.30 (ArC), 133.84 (ArC), 133.37 (ArC), 133.18 (ArC), 132.74 (ArC), 131.94 (ArC), 128.63 (ArC), 126.40 (ArC), 123.86 (ArC), 118.60 (ArC), 115.29 (ArC), 42.49 (AlC). FT IR: 3362, 3290, 3246 (N-H, Str), 3027 (C-H Ar, Str), 2970 (C-H Aliph, Str), 1739 (C-H Ar, Bend), 1627, 1613 (C=N, Str), 1558, 1534 (N-H, Bend), 1494, 1471, 1426 (-CH_2_, Bend) 1380, 1353, 1326 (S=O, -SO_2_, Str), 1296, 1285, 1254, 1232 (C-N Ar, Str), 1212, 1199, 1192 (C-N, Str), 1158 (S=O, sulfonamide, Str), 1134, 1109, 1086, 1042 (C-N, Str), 980, 940, 925 (C=C Ar, Bend), 840, 781, 754, 733, 719, 696 (C-Cl, Str).

**3,5-dichloro-*N*-(3,4-dihydroquinazolin-2-yl)benzene-1-sulfonamide PR23**

Prepared in method **C^1^**, Formula weight for C_14_H_11_Cl_2_N_3_O_2_S: 356.2 g/mol, UPLC-MS: [M + H]+ = 356.1, purity = 100%, R_t_ = 7.67 min, Y = 76%, mp = 208–211 °C. ^1^H NMR (400 MHz, DMSO) δ 10.18 (bp, 1H, NH), 8.03 (bp, 1H, NH), 7.88–7.84 (m, 2H, ArH), 7.18 (t, *J* = 7.7 Hz, 1H, ArH), 7.13 (d, *J* = 7.3 Hz, 1H, ArH), 7.00 (dd, *J* = 7.4, 6.6 Hz, 1H, ArH), 6.90 (d, *J* = 7.9 Hz, 1H, ArH), 4.46 (s, 2H, AlH). ^13^C NMR (101 MHz, DMSO) δ 152.52 (C_Gua_), 147.51 (ArC), 135.14 (2ArC), 131.59 (ArC), 128.61 (ArC), 126.40 (ArC), 124.85 (2ArC), 123.90 (ArC), 118.42 (ArC), 115.27 (ArC), 42.67 (AlC). FT IR: 3319, 3262, 3163 (N-H, Str), 3080, 3036 (C-H Ar, Str), 2970, 2920, 2842 (C-H Aliph, Str), 1738 (C-H Ar, Bend), 1642, 1615 (C=N, Str), 1569, 1534 (N-H, Bend), 1493, 1450, 1417 (-CH_2_, Bend) 1395, 1371, 1338 (S=O, -SO_2_, Str), 1298, 1287, 1230 (C-N Ar, Str), 1212 (C-N, Str), 1159, 1145 (S=O, sulfonamide, Str), 1112, 1087 (C-N, Str), 995, 974, 944, 932 (C=C Ar, Bend), 857, 797, 775, 761, 731, 720 (C-Cl, Str).

***N*** **-(3,4-dihydroquinazolin-2-yl)-3-methoxybenzene-1-sulfonamide PR24**

Prepared in method **C^2^**, Formula weight for C_15_H_15_N_3_O_3_S: 317.4 g/mol, UPLC-MS: [M + H]^+^ = 318.2, purity = 99%, R_t_ = 5.87 min, Y = 76%, mp = 108–114 °C. ^1^H NMR (400 MHz, DMSO) δ 10.09 (bp, 1H, NH), 8.00 (bp, 1H, NH), 7.52–7.39 (m, 3H, ArH), 7.15 (dddd, *J* = 10.2, 7.7, 2.9, 2.0 Hz, 3H, ArH), 6.98 (td, *J* = 7.5, 1.0 Hz, 1H, ArH), 6.88 (d, *J* = 7.4 Hz, 1H, ArH), 4.44 (s, 2H, AlH), 3.81 (s, 3H, OCH_3_). ^13^C NMR (101 MHz, DMSO) δ 159.63 (C_Gua_), 152.62 (ArC), 145.71 (ArC), 135.46 (ArC), 130.52 (ArC), 128.57 (ArC), 126.34 (ArC), 123.61 (ArC), 118.28 (ArC), 118.08 (ArC), 117.74 (ArC), 115.10 (ArC), 111.29 (ArC), 55.99 (OCH_3_), 42.57 (AlC). FT IR: 3327, 3303, 3226 (N-H, Str), 3066 (C-H Ar, Str), 2962, 2921, 2837 (C-H Aliph, Str), 1748 (C-H Ar, Bend), 1627 (C=N, Str), 1597 (N-H, Bend), 1540 (C=C Ar, Str), 1476, 1441, 1431 (-CH_2_, Bend) 1368, 1325 (S=O, -SO_2_, Str), 1284, 1273 (C-O, Str), 1254, 1240 (C-N Ar, Str), 1211 (C-N, Str), 1182, 1149 (S=O, sulfonamide, Str), 1118, 1091, 1076, 1037 (C-N, Str), 990, 963, 944, 924 (C=C Ar, Bend).

**5-chloro-*N*-(3,4-dihydroquinazolin-2-yl)-2-methoxybenzene-1-sulfonamide PR25**

Prepared in method **C^2^**, Formula weight for C_15_H_14_ClN_3_O_3_S: 351.8 g/mol, UPLC-MS: [M + H]^+^ = 352.1, purity = 91%, R_t_ = 6.25 min, Y = 63%, mp = 157–165 °C. ^1^H NMR (400 MHz, DMSO) δ 10.01 (s, 1H, NH), 8.03 (s, 1H, ArH), 7.77 (d, J = 2.7 Hz, 1H, ArH, ArH), 7.65 (d, J = 2.7 Hz, 1H, ArH), 7.62 (dd, J = 8.8, 2.7 Hz, 1H, ArH), 7.57 (dd, J = 8.8, 2.7 Hz, 1H, ArH), 7.29–7.21 (m, 3H, ArH), 7.03 (td, J = 7.5, 0.9 Hz, 1H, ArH), 6.95 (d, J = 7.7 Hz, 1H, ArH), 4.56 (s, 2H, AlH), 3.80 (s, 3H, OCH_3_). ^13^C NMR (101 MHz, DMSO) δ 155.73 (C_Gua_), 155.41 (ArC), 153.44 (ArC), 135.56 (ArC), 133.66 (ArC), 133.13 (ArC), 128.61 (ArC), 127.77 (ArC), 127.46 (ArC), 126.40 (ArC), 123.67 (ArC), 118.66 (ArC), 115.18 (ArC), 56.96 (OCH3), 42.50 (AlC). FT IR: 3318, 3258 (N-H, Str), 3097, 3077 (C-H Ar, Str), 2937, 2848 (C-H Aliph, Str), 1626, 1605 (C=N, Str), 1589 (N-H, Bend), 1531, 1513 (C=C Ar, Str), 1476, 1461, 1433 (-CH_2_, Bend) 1392 (S=O, -SO_2_, Str), 1271 (C-O, Str), 1248 (C-N Ar, Str), 1209 (C-N, Str), 1164, 1155 (S=O, sulfonamide, Str), 1102, 1063, 1018 (C-N, Str), 967, 930, 905 (C=C Ar, Bend), 887, 873, 835, 820, 809, 758, 724 (C-Cl, Str).

***N*** **-(3,4-dihydroquinazolin-2-yl)-[1,1′-biphenyl]-4-sulfonamide PR26**

Prepared in method **C^1^**, described in [31].

***N*** **-(6-chloro-3,4-dihydroquinazolin-2-yl)benzenesulfonamide PR27**

Prepared in method **C^1^**, described in [31].

***N*** **-(4-methyl-3,4-dihydroquinazolin-2-yl)benzenesulfonamide PR28**

Prepared in method **C^1^**, described in [31].

***N*** **-(3,4-dihydroquinazolin-2-yl)naphthalene-2-sulfonamide PR29**

Prepared in method **B^2^**, described in [31].

**5-chloro-*N*-(3,4-dihydroquinazolin-2-yl)naphthalene-2-sulfonamide PR30**

Prepared in method **C^2^**, Formula weight for C_18_H_14_ClN_3_O_2_S: 371.8 g/mol, UPLC-MS: [M + H]^+^ = 372.2, purity = 99%, R_t_ = 6.77 min, Y = 58%, mp = 192–195 °C, Composition: C(58.14%) H(3.80%) Cl(9.53%) N(11.30%) O(8.61%) S(8.62%), found: C(58.08%) H(3.89%), N(11.32%), S(8.60%).^1^H NMR (400 MHz, DMSO) δ 10.14 (s, 1H, bp), 8.61 (d, *J* = 1.6 Hz, 1H, ArH), 8.32 (d, *J* = 8.9 Hz, 1H, ArH), 8.15 (d, *J* = 8.3 Hz, 1H, ArH), 8.11–8.00 (m, 2H, ArH. NH), 7.85 (dd, *J* = 7.5, 0.9 Hz, 1H, ArH), 7.65 (q, *J* = 8.0 Hz, 1H, ArH), 7.16 (t, *J* = 7.7 Hz, 1H, ArH), 7.12 (d, *J* = 7.3 Hz, 1H, ArH), 6.98 (td, *J* = 7.5, 0.9 Hz, 1H, ArH), 6.89 (d, *J* = 7.9 Hz, 1H, ArH), 4.46 (s, 2H, AlH). ^13^C NMR (101 MHz, DMSO) δ 152.62 (C_Gua_), 142.55 (ArC), 135.36 (ArC), 133.69 (ArC), 131.16 (ArC), 131.08 (ArC), 129.27 (ArC), 128.95 (ArC), 128.57 (ArC), 128.15 (ArC), 126.68 (ArC), 126.34 (ArC), 125.44 (ArC), 124.51 (ArC), 123.69 (ArC), 118.46 (ArC), 115.16 (ArC), 42.61 (AlC). FT IR: 3304, 3173 (N-H, Str), 3041 (C-H Ar, Str), 1636, 1586 (C=N, Str), 1540, 1505 (N-H, Bend), 1492, 1444, 1414 (-CH_2_, Bend) 1366, 1333 (S=O, -SO_2_, Str), 1275, 1237, 1210 (C-N Ar, Str), 1156, 1115 (S=O, sulfonamide, Str), 1074, 1038 (C-N, Str), 993, 969, 936, 884, 868 (C=C Ar, Bend), 838, 824, 811, 790, 747, 717, 697, 680, 650 (C-Cl, Str).

***N*** **-(5-chloro-3,4-dihydroquinazolin-2-yl)naphthalene-2-sulfonamide PR31**

Prepared in method **C^2^**, Formula weight for C_18_H_14_ClN_3_O_2_S: 371.8 g/mol, UPLC-MS: [M + H]^+^ = 372.3, purity = 100%, R_t_ = 6.76 min, Y = 73%, mp = 202–204 °C.^1^H NMR (400 MHz, DMSO) δ 10.27 (bp, 1H, NH), 8.50 (s, 1H, ArH), 8.13–8.08 (m, 1H, ArH), 8.06 (d, *J* = 8.5 Hz, 1H, ArH), 8.01 (d, *J* = 6.9 Hz, 1H, ArH), 7.88 (dd, *J* = 8.6, 1.8 Hz, 1H, ArH), 7.68–7.60 (m, 2H, ArH), 7.17 (t, *J* = 7.6 Hz, 1H, ArH), 7.03 (d, *J* = 8.0 Hz, 1H, ArH), 6.80 (d, *J* = 7.6 Hz, 1H, ArH), 4.50 (s, 2H, AlH). ^13^C NMR (101 MHz, DMSO) δ 134.26 (C_Gua_), 134.24 (ArC), 132.22 (ArC), 130.80 (ArC), 129.85 (ArC), 129.52 (ArC), 129.48 (ArC), 129.22 (ArC), 128.60 (ArC), 128.24 (ArC), 128.19 (ArC), 127.79 (ArC), 127.74 (ArC), 126.22 (ArC), 123.25 (ArC), 122.96 (ArC), 116.61 (ArC), 41.41 (AlC). FT IR: 3313, 3263, 3206 (N-H, Str), 3055 (C-H Ar, Str), 1616, 1601 (C=N, Str), 1527 (N-H, Bend), 1477, 1432 (-CH_2_, Bend) 1373, 1325 (S=O, -SO_2_, Str), 1284, 1256, 1214, 1194 (C-N Ar, Str), 1150, 1136 (S=O, sulfonamide, Str), 1099, 1069 (C-N, Str), 1006, 947, 886, 860 (C=C Ar, Bend), 845, 817, 785, 777, 746, 705, 672, 651 (C-Cl, Str).

***N*** **-(6-chloro-3,4-dihydroquinazolin-2-yl)naphthalene-2-sulfonamide PR32**

Prepared in method **B^2^**, described in [31].

***N*** **-(7-chloro-3,4-dihydroquinazolin-2-yl)naphthalene-2-sulfonamide PR33**

Prepared in method **C^2^**, Formula weight for C_18_H_14_ClN_3_O_2_S: 371.8 g/mol, UPLC-MS: [M + H]^+^ = 372.3, purity = 100%, R_t_ = 6.66 min, Y = 64%, mp = 220–222 °C, Composition: C(58.14%) H(3.80%) Cl(9.53%) N(11.30%) O(8.61%) S(8.62%), found: C(58.11%) H(3.82%), N(11.28%), S(8.56%). ^1^H NMR (400 MHz, DMSO) δ 10.09 (bp, 1H, NH), 8.51 (s, 1H, ArH), 8.13–8.09 (m, 1H, ArH), 8.05 (t, *J* = 8.2 Hz, 1H, ArH), 8.02 (dd, *J* = 7.8, 5.5 Hz, 1H, ArH), 7.87 (dd, *J* = 8.6, 1.8 Hz, 1H, ArH), 7.69–7.61 (m, 2H, ArH), 7.14 (d, *J* = 8.2 Hz, 1H, ArH), 6.99 (dd, *J* = 8.1, 2.1 Hz, 1H, ArH), 6.89 (d, *J* = 2.0 Hz, 1H, ArH), 4.43 (s, 2H, AlH). ^13^C NMR (101 MHz, DMSO) δ 152.52 (C_Gua_), 141.45 (ArC), 137.70 (ArC), 137.67 (ArC), 134.25 (ArC), 132.61 (ArC), 132.21 (ArC), 129.46 (ArC), 129.26 (ArC), 128.63 (ArC), 128.20 (ArC), 128.04 (ArC), 127.76 (ArC), 126.22 (ArC), 122.90 (ArC), 117.65 (ArC), 114.85 (ArC), 42.29 (AlC). FT IR: 3293, 3257, 3151 (N-H, Str), 3047, 3004 (C-H Ar, Str), 2928 (C-H Aliph, Str), 1636, 1614 (C=N, Str), 1534 (N-H, Bend), 1484, 1412 (-CH_2_, Bend) 1360, 1326 (S=O, -SO_2_, Str), 1285, 1269, 1243, 1231, 1206 (C-N Ar, Str), 1153, 1138 (S=O, sulfonamide, Str), 1108, 1086, 1067, 1019 (C-N, Str), 973, 948, 940, 929, 900, 864, 854 (C=C Ar, Bend), 817, 793, 771, 750, 722, 692, 659 (C-Cl, Str).

***N*** **-(4-methyl-3,4-dihydroquinazolin-2-yl)naphthalene-2-sulfonamide PR34**

Prepared in method **C^1^**, described in [31].

***N*** **-(6-chloro-4-methyl-3,4-dihydroquinazolin-2-yl)naphthalene-2-sulfonamide PR35**

Prepared in method **C^1^**, described in [31].

***N*** **-(6-bromo-4-methyl-3,4-dihydroquinazolin-2-yl)naphthalene-2-sulfonamide PR36**

Prepared in method **C^1^**, described in [31].

***N*** **-(7-chloro-4-methyl-3,4-dihydroquinazolin-2-yl)naphthalene-2-sulfonamide PR37**

Prepared in method **C^2^**, Formula weight for C_19_H_16_ClN_3_O_2_S: 385.7 g/mol, UPLC-MS: [M + H]^+^ = 386.3, purity = 94%, R_t_ = 6.97 min, Y = 57%, mp = 167–171 °C. ^1^H NMR (400 MHz, DMSO) δ 10.35 (bp, 1H, NH), 8.54 (s, 1H, ArH), 8.21 (bp, 1H, NH), 8.15–8.04 (m, 2H, ArH), 8.04–7.98 (m, 1H, ArH), 7.86 (dd, *J* = 8.6, 1.8 Hz, 1H, ArH), 7.72–7.58 (m, 2H, ArH), 7.20 (d, *J* = 8.3 Hz, 1H, ArH), 7.06–7.02 (m, 1H, ArH), 6.93 (d, *J* = 2.1 Hz, 1H, ArH), 4.73 (dd, *J* = 6.4, 3.3 Hz, 1H, AlH), 1.14 (d, *J* = 6.5 Hz, 3H, AlH). ^13^C NMR (101 MHz, DMSO) δ 151.40 (C_Gua_), 141.12 (ArC), 135.86 (ArC), 134.28 (ArC), 132.67 (ArC), 132.19 (ArC), 129.39 (ArC), 129.36 (ArC), 128.71 (ArC), 128.24 (ArC), 128.04 (ArC), 127.85 (ArC), 126.40 (ArC), 123.37 (ArC), 122.79 (ArC), 122.57 (ArC), 114.72 (ArC), 48.50 (AlC), 25.00 (AlC). FT IR: 3314, 3287 (N-H, Str), 3175 (C-H Ar, Str), 2975, 2922 (C-H Aliph, Str), 1615 (C=N, Str), 1526 (N-H, Bend), 1483, 1445, 1408 (-CH_2_, Bend) 1369, 1359, 1345, 1315 (S=O, -SO_2_, Str), 1288, 1272, 1255, 1240, 1208 (C-N Ar, Str), 1151, 1117 (S=O, sulfonamide, Str), 1102, 1088, 1075, 1067, 1047, 1022 (C-N, Str),994, 969, 947, 938, 917, 900 (C=C Ar, Bend), 875, 861, 812, 767, 755, 737, 717, 695, 662 (C-Cl, Str).

***N*** **-(4,5-dihydro-1H-1,3-benzodiazepin-2-yl)naphthalene-2-sulfonamide PR38**

Prepared in method **B^1^**, Formula weight for C_19_H_17_N_3_O_2_S: 352.4 g/mol, UPLC-MS: [M + H]^+^ = 352.2, purity = 89%, R_t_ = 6.65 min, Y = 54%, mp = 175–180 °C. ^1^H NMR (400 MHz, DMSO) δ 9.73 (bp, 1H, NH), 8.49 (s, 1H, ArH), 8.34 (bp, 1H, NH), 8.14–7.99 (m, 4H, ArH), 7.87 (dd, *J* = 8.6, 1.8 Hz, 1H, ArH), 7.66 (ddd, *J* = 9.2, 4.9, 2.5 Hz, 2H, ArH), 7.17 (ddd, *J* = 9.3, 7.6, 3.9 Hz, 2H, ArH), 6.99–6.89 (m, 1H, ArH), 3.49 (dd, *J* = 9.3, 4.7 Hz, 2H, AlH), 2.99–2.86 (m, 2H, ArH). ^13^C NMR (101 MHz, DMSO) δ 155.08 (C_Gua_), 141.61 (ArC), 137.74 (ArC), 134.22 (ArC), 132.26 (ArC), 130.74 (ArC), 129.77 (ArC), 129.50 (ArC), 129.45 (ArC), 129.31 (ArC), 128.59 (ArC), 128.20 (ArC), 127.76 (ArC), 126.11 (ArC), 123.46 (ArC), 122.84 (ArC), 120.70 (ArC), 43.71 (AlC), 35.08 (AlC). FT IR: 3334, 3241, 3125 (N-H, Str), 3008 (C-H Ar, Str), 2970, 2947 (C-H Aliph, Str), 1748 (C-H Ar, Bend), 1657 (C=N, Str), 1595, 1551, 1503 (N-H, Bend), 1458 (-CH_2_, Bend) 1343, 1332 (S=O, -SO_2_, Str), 1280, 1256, 1206 (C-N Ar, Str), 1158, 1147, 1131, 1106, 1068, 1014 (C-N, Str), 962, 902, 884, 864 (C=C Ar, Bend).

***N*** **-(3,4-dihydroquinazolin-2-yl)naphthalene-2-sulfonamide PR39**

Prepared in method **C^1^**, described in [31].

***N*** **-(3,4-dihydroquinazolin-2-yl)naphthalene-1-sulfonamide PR40**

Prepared in method **B^2^**, described in [30].

**2-chloro-*N*-(3,4-dihydroquinazolin-2-yl)naphthalene-1-sulfonamide PR41**

Prepared in method **C^2^**, Formula weight for C_18_H_14_ClN_3_O_2_S: 371.8 g/mol, UPLC-MS: [M + H]^+^ = 372.3, purity = 95%, R_t_ = 6.55 min, Y = 46%, mp = 241–244 °C, Composition: C(58.14%) H(3.80%) Cl(9.53%) N(11.30%) O(8.61%) S(8.62%), found: C(57.99%) H(3.82%), N(11.23%), S(8.67%).^1^H NMR (400 MHz, DMSO) δ 10.05 (bp, 1H, NH), 9.19 (d, *J* = 8.6 Hz, 1H, ArH), 8.10 (d, *J* = 8.7 Hz, 1H, ArH), 8.06–7.99 (m, *J* = 6.7 Hz, 2H, ArH, NH), 7.73–7.66 (m, 1H, ArH), 7.63 (dd, *J* = 10.9, 4.9 Hz, 2H, ArH), 7.21–7.11 (m, 2H, ArH), 7.00 (t, *J* = 7.0 Hz, 1H, ArH), 6.90 (d, *J* = 7.8 Hz, 1H, ArH), 4.49 (s, 2H, AlH). ^13^C NMR (101 MHz, DMSO) δ 152.63 (C_Gua_), 136.82 (ArC), 135.30 (ArC), 133.72 (ArC), 133.01 (ArC), 131.60 (ArC), 130.87 (ArC), 129.41 (ArC), 129.06 (ArC), 128.60 (ArC), 128.31 (ArC), 127.02 (ArC), 126.88 (ArC), 126.37 (ArC), 123.81 (ArC), 118.58 (ArC), 115.29 (ArC), 42.48 (AlC). FT IR: 3384, 3320 (N-H, Str), 3056 (C-H Ar, Str), 2907, 2854 (C-H Aliph, Str), 1624, 1606 (C=N, Str), 1537 (N-H, Bend), 1495, 1473, 1422 (-CH_2_, Bend) 1368, 1330 (S=O, -SO_2_, Str), 1299, 1281, 1260, 1232 (C-N Ar, Str), 1178, 1162, 1146, 1105, 1035, 1012 (C-N, Str), 985, 941, 927 (C=C Ar, Bend), 860, 838, 808, 768, 758, 748, 740, 711, 682, 663 (C-Cl, Str).

**4-chloro-*N*-(3,4-dihydroquinazolin-2-yl)naphthalene-1-sulfonamide PR42**

Prepared in method **C^1^**, Formula weight for C_18_H_14_ClN_3_O_2_S: 371.8 g/mol, UPLC-MS: [M + H]^+^ = 372.1, purity = 92%, R_t_ = 7.68 min, Y = 90%, mp = 188–191 °C, Composition: C(58.14%) H(3.80%) Cl(9.53%) N(11.30%) O(8.61%) S(8.62%), found: C(58.23%) H(3.91%), N(11.25%), S(8.68%). ^1^H NMR (400 MHz, DMSO) δ 10.06 (bp, 1H, NH), 8.95–8.85 (m, 1H, ArH), 8.33–8.28 (m, 1H, ArH), 8.23 (d, *J* = 7.9 Hz, 1H, ArH), 7.95 (bp, 1H, NH), 7.84–7.79 (m, 3H, ArH), 7.14 (t, *J* = 7.6 Hz, 1H, ArH), 7.09 (d, *J* = 7.4 Hz, 1H, ArH), 6.96 (td, *J* = 7.5, 1.0 Hz, 1H, ArH), 6.89 (d, *J* = 7.9 Hz, 1H, ArH), 4.39 (d, *J* = 19.6 Hz, 2H, AlH). ^13^C NMR (101 MHz, DMSO) δ 152.56 (C_Gua_), 139.05 (ArC), 135.49 (ArC), 135.33 (ArC), 130.89 (ArC), 129.58 (ArC), 128.71 (ArC), 128.60 (ArC), 128.53 (ArC), 127.20 (ArC), 127.02 (ArC), 126.28 (ArC), 125.50 (ArC), 124.81 (ArC), 123.70 (ArC), 118.42 (ArC) 115.20 (ArC), 42.61 (ArC). FT IR: 3348, 3311, 3248 (N-H, Str), 3075, 3029 (C-H Ar, Str), 2970, 2922 (C-H Aliph, Str), 1738 (C-H Ar, Bend), 1670, 1621, 1606 (C=N, Str), 1562, 1533 (N-H, Bend), 1494, 1472 (-CH_2_, Bend) 1371, 1354, 1332 (S=O, -SO_2_, Str), 1300, 1278, 1258, 1236 (C-N Ar, Str), 1215, 1202 (C-N, Str), 1164, 1151 (S=O, sulfonamide, Str), 1136, 1112, 1092, 1032 (C-N, Str), 994, 965, 924 (C=C Ar, Bend), 833, 790, 754, 680 (C-Cl, Str).

**5-chloro-*N*-(3,4-dihydroquinazolin-2-yl)naphthalene-1-sulfonamide PR43**

Prepared in method **C^1^**, Formula weight for C_18_H_14_ClN_3_O_2_S: 371.8 g/mol, UPLC-MS: [M + H]^+^ = 372.1, purity = 87%, R_t_ = 7.64 min, Y = 77%, mp = 185–191 °C, Composition: C(58.14%) H(3.80%) Cl(9.53%) N(11.30%) O(8.61%) S(8.62%), found: C(58.01%) H(3.84%), N(11.23%), S(8.67%). ^1^H NMR (400 MHz, DMSO) δ 10.06 (bp, 1H, NH), 8.84 (d, *J* = 8.7 Hz, 1H, ArH), 8.43 (d, *J* = 8.5 Hz, 1H, ArH), 8.37 (dd, *J* = 7.3, 1.0 Hz, 1H, ArH), 7.96 (bp, 1H, NH), 7.84–7.79 (m, 2H, ArH), 7.70 (dd, *J* = 7.2, 1.4 Hz, 1H, ArH), 7.14 (t, *J* = 7.6 Hz, 1H, ArH), 7.09 (d, *J* = 7.3 Hz, 1H, ArH), 6.97–6.91 (m, 1H, ArH), 6.89 (d, *J* = 7.8 Hz, 1H, ArH), 4.41 (s, 2H, AlH). ^13^C NMR (101 MHz, DMSO) δ 152.57 (C_Gua_), 140.19 (ArC), 135.34 (ArC), 131.64 (ArC), 131.13 (ArC), 129.84 (ArC), 128.62 (ArC), 128.54 (ArC), 127.89 (ArC), 127.81 (ArC), 127.59 (ArC), 126.64 (ArC), 126.29 (ArC), 126.11 (ArC), 123.69 (ArC), 118.42 (ArC), 115.20 (ArC), 42.60 (AlC). FT IR: 3284, 3244 (N-H, Str), 3028 (C-H Ar, Str), 2970, 2922, 2852 (C-H Aliph, Str), 1738 (C-H Ar, Bend), 1629, 1613 (C=N, Str), 1563, 1534 (N-H, Bend), 1496, 1454, 1403 (-CH_2_, Bend) 1372, 1327 (S=O, -SO_2_, Str), 1301, 1278, 1268, 1232 (C-N Ar, Str), 1216 (C-N, Str), 1137, 1097, 1083, 1040, 1011 (C-N, Str), 969, 936 (C=C Ar, Bend), 844, 814, 783, 738, 721 (C-Cl, Str).

**6-chloro-*N*-(3,4-dihydroquinazolin-2-yl)naphthalene-1-sulfonamide PR44**

Prepared in method **C^2^**, Formula weight for C_18_H_14_ClN_3_O_2_S: 371.8 g/mol, UPLC-MS: [M + H]^+^ = 372.1, purity = 97%, R_t_ = 7.59 min, Y = 78%, mp = 210–212 °C.^1^H NMR (400 MHz, DMSO) δ 10.05 (bp, 1H, NH), 8.80 (d, *J* = 9.2 Hz, 1H, ArH), 8.25 (dd, *J* = 7.3, 1.0 Hz, 1H, ArH), 8.19 (t, *J* = 8.3 Hz, 1H, ArH), 8.14 (d, *J* = 8.3 Hz, 1H, ArH), 7.95 (bp, 1H, NH), 7.74 (dd, *J* = 9.2, 2.3 Hz, 1H, ArH), 7.71–7.63 (m, 1H, ArH), 7.15 (t, *J* = 7.7 Hz, 1H, ArH), 7.09 (d, *J* = 7.3 Hz, 1H, ArH), 7.00–6.94 (m, 1H, ArH), 6.88 (d, *J* = 7.9 Hz, 1H, ArH), 4.40 (s, 2H, AlH). ^13^C NMR (101 MHz, DMSO) δ 152.55 (C_Gua_), 139.64 (ArC), 135.36 (ArC), 135.18 (ArC), 132.36 (ArC), 131.71 (ArC), 128.63 (ArC), 128.54 (ArC), 128.14 (ArC), 127.58 (ArC), 127.12 (ArC), 126.80 (ArC), 126.30 (2ArC), 123.68 (ArC), 118.43 (ArC), 115.18 (ArC), 42.59 (AlC). FT IR: 3372, 3298 (N-H, Str), 3029 (C-H Ar, Str), 2970, 2853 (C-H Aliph, Str), 1738 (C-H Ar, Bend), 1625, 1611 (C=N, Str), 1539 (N-H, Bend), 1495, 1474, 1436 (-CH_2_, Bend) 1367, 1328 (S=O, -SO_2_, Str), 1304, 1275, 1254 (C-N Ar, Str), 1216, 1202 (C-N, Str), 1152 (S=O, sulfonamide, Str), 1137, 1096 (C-N, Str), 991, 938 (C=C Ar, Bend), 837, 816, 789, 751, 709, 685 (C-Cl, Str).

**8-chloro-*N*-(3,4-dihydroquinazolin-2-yl)naphthalene-1-sulfonamide PR45**

Prepared in method **C^2^**, Formula weight for C_18_H_14_ClN_3_O_2_S: 371.8 g/mol, UPLC-MS: [M + H]^+^ = 372.3, purity = 98%, R_t_ = 5.98 min, Y = 42%, mp = 103–105 °C.^1^H NMR (400 MHz, DMSO) δ 10.05 (bp, 1H, NH), 8.55 (dd, *J* = 7.5, 1.3 Hz, 1H, ArH), 8.23 (dd, *J* = 8.2, 1.1 Hz, 1H, ArH), 8.07 (dd, *J* = 8.2, 1.1 Hz, 1H, ArH), 7.90 (bp, 1H, NH), 7.82 (dd, *J* = 7.5, 1.3 Hz, 1H, ArH), 7.67 (t, *J* = 7.8 Hz, 1H, ArH), 7.58 (t, *J* = 7.8 Hz, 1H, ArH), 7.19 (t, *J* = 7.7 Hz, 1H, ArH), 7.14 (d, *J* = 7.4 Hz, 1H, ArH), 7.02–6.97 (m, 1H, ArH), 6.92 (d, *J* = 7.9 Hz, 1H, ArH), 4.44 (s, 2H, AlH). ^13^C NMR (101 MHz, DMSO) δ 152.64 (C_Gua_), 139.84 (ArC), 137.05 (ArC), 135.73 (ArC), 134.71 (ArC), 132.33 (ArC), 130.92 (ArC), 129.58 (ArC), 129.23 (ArC), 128.57 (ArC), 127.15 (ArC), 126.33 (ArC), 125.46 (ArC), 123.48 (ArC), 118.58 (ArC), 115.12 (ArC), 42.60 (AlC). FT IR: 3303 (N-H, Str), 2957, 2916, 2848 (C-H Aliph, Str), 1625, 1602 (C=N, Str), 1533 (N-H, Bend), 1493, 1471 (-CH_2_, Bend) 1369, 1323 (S=O, -SO_2_, Str), 1259 (C-N Ar, Str), 1209, 1196 (C-N, Str), 1138, 1092, 1034 (C-N, Str), 1007, 988, 936 (C=C Ar, Bend), 882, 866, 819, 749, 729 (C-Cl, Str).

***N*** **-(5-chloro-3,4-dihydroquinazolin-2-yl)naphthalene-1-sulfonamide PR46**

Prepared in method **B^2^**, Formula weight for C_18_H_14_ClN_3_O_2_S: 371.8 g/mol, UPLC-MS: [M + H]^+^ = 372.2, purity = 100%, R_t_ = 7.49 min, Y = 73%, mp = 206–201 °C, Composition: C(58.14%) H(3.80%) Cl(9.53%) N(11.30%) O(8.61%) S(8.62%), found: C(58.23%) H(3.76%), N(11.42%), S(8.49%).^1^H NMR (400 MHz, DMSO) δ 8.81 (d, *J* = 8.2 Hz, 1H, ArH), 8.22 (d, *J* = 7.3 Hz, 1H, ArH), 8.11 (d, *J* = 8.2 Hz, 1H, ArH), 8.02 (d, *J* = 8.2 Hz, 1H, ArH), 7.65 (t, *J* = 7.1 Hz, 1H, ArH), 7.63–7.56 (m, 2H, ArH), 7.09 (t, *J* = 8.0 Hz, 1H, ArH), 6.94 (d, *J* = 7.5 Hz, 1H, ArH), 6.70 (s, 1H, ArH), 4.41 (s, 2H, AlH). ^13^C NMR (101 MHz, DMSO) δ 134.20 (C_Gua_), 132.63 (ArC), 132.60 (ArC), 130.67 (ArC), 130.62 (ArC), 129.61 (ArC), 129.56 (ArC), 129.55 (ArC), 128.85 (ArC), 128.58 (ArC), 127.39 (ArC), 127.04 (ArC), 126.99 (ArC), 126.77, (ArC) 126.69 (ArC), 124.89 (ArC), 124.82 (ArC), 41.48 (AlC).

***N*** **-(6-chloro-3,4-dihydroquinazolin-2-yl)naphthalene-1-sulfonamide PR47**

Prepared in method **B^2^**, Formula weight for C_18_H_14_ClN_3_O_2_S: 371.8 g/mol, UPLC-MS: [M + H]^+^ = 372.2, purity = 100%, R_t_ = 7.39 min, Y = 71%, mp = 275 -277 °C. ^1^H NMR (400 MHz, DMSO) δ 10.13 (bp, 1H, NH), 8.78 (d, *J* = 8.5 Hz, 1H, ArH), 8.25 (d, *J* = 6.7 Hz, 1H, ArH), 8.15 (d, *J* = 8.2 Hz, 1H, ArH), 8.05 (d, *J* = 8.0 Hz, 1H, ArH), 7.98 (bp, 1H, NH), 7.68 (dd, *J* = 13.6, 6.5 Hz, 1H, ArH), 7.66–7.57 (m, 2H, ArH), 7.21 (dd, *J* = 11.9, 3.3 Hz, 2H, ArH), 6.88 (d, *J* = 8.4 Hz, 1H, ArH), 4.40 (s, 2H, A;H). ^13^C NMR (101 MHz, DMSO) δ 152.28 (C_Gua_), 139.22 (ArC), 134.59 (ArC), 134.27 (ArC), 133.19 (ArC), 129.02 (ArC), 128.38 (ArC), 128.37 (ArC), 127.73 (ArC), 127.11 (ArC), 127.00 (ArC), 126.88 (ArC), 126.37 (ArC), 126.17 (ArC), 124.87 (ArC), 120.63 (ArC), 116.73 (ArC), 42.30 (AlC). FT IR: 3352, 3245, 3146 (N-H, Str), 3008 (C-H Ar, Str), 2970 (C-H Aliph, Str), 1739 (C-H Ar, Bend), 1630 (C=N, Str), 1568, 1508 (N-H, Bend), 1478, 1411 (-CH_2_, Bend) 1362, 1344, 1322 (S=O, -SO_2_, Str), 1289, 1280, 1264, 1231 (C-N Ar, Str), 1217, 1200 (C-N, Str), 1155, 1144 (S=O, sulfonamide, Str), 1104, 1083, 1027 (C-N, Str), 980, 957, 927 (C=C Ar, Bend), 817, 807, 769, 752, 728, 700, 679 (C-Cl, Str).

***N*** **-(7-chloro-3,4-dihydroquinazolin-2-yl)naphthalene-1-sulfonamide PR48**

Prepared in method **B^2^**, Formula weight for C_18_H_14_ClN_3_O_2_S: 371.8 g/mol, UPLC-MS: [M + H]^+^ = 372.2, purity = 100%, R_t_ = 7.43 min, Y = 64%, mp = 214–219 °C. ^1^H NMR (400 MHz, DMSO) δ 10.13 (bp, 1H, NH), 8.79 (d, *J* = 8.5 Hz, 1H, ArH), 8.26 (d, *J* = 7.2 Hz, 1H, ArH), 8.16 (d, *J* = 8.2 Hz, 1H, ArH), 8.05 (bp, 2H, ArH, NH), 7.70 (t, *J* = 7.3 Hz, 1H, ArH), 7.63 (td, *J* = 7.7, 3.1 Hz, 2H, ArH), 7.14 (d, *J* = 8.1 Hz, 1H, ArH), 7.00 (dd, *J* = 8.1, 1.7 Hz, 1H, ArH), 6.92 (d, *J* = 1.6 Hz, 1H, ArH), 4.40 (s, 2H, AlH).^13^C NMR (101 MHz, DMSO) δ 152.27 (C_Gua_), 139.18 (ArC), 137.11 (ArC), 134.28 (ArC), 133.23 (ArC), 132.66 (ArC), 129.04 (ArC), 128.39 (ArC), 128.06 (ArC), 127.76 (ArC), 127.02 (ArC), 126.90 (ArC), 126.35 (ArC), 124.88 (ArC), 123.14 (ArC), 117.50 (ArC), 114.68 (ArC), 42.25 (AlC). FT IR: 3322 (N-H, Str), 3059 (C-H Ar, Str), 2970, 2945 (C-H Aliph, Str), 1739 (C-H Ar, Bend), 1617 (C=N, Str), 1528, 1506 (N-H, Bend), 1493, 1469, 1452, 1428, 1401 (-CH_2_, Bend) 1362, 1323 (S=O, -SO_2_, Str), 1295, 1271, 1250, 1232 (C-N Ar, Str), 1212, 1198, 1170 (C-N, Str), 1155, 1138 (S=O, sulfonamide, Str), 1082, 1024 (C-N, Str), 994, 940 (C=C Ar, Bend), 817, 797, 769, 745, 711, 679 (C-Cl, Str).

***N*** **-(8-chloro-3,4-dihydroquinazolin-2-yl)naphthalene-1-sulfonamide PR49**

Prepared in method **B^1^**, Formula weight for C_18_H_14_ClN_3_O_2_S: 371.8 g/mol, UPLC-MS: [M + H]^+^ = 372.1, purity = 92%, R_t_ = 7.70 min, Y = 86%, mp = 250–256 °C, Composition: C(58.14%) H(3.80%) Cl(9.53%) N(11.30%) O(8.61%) S(8.62%), found: C(57.64%) H(3.83%), N(11.22%), S(8.52%). ^1^H NMR (400 MHz, DMSO) δ 9.75 (bp, 1H, NH), 8.77 (d, *J* = 8.6 Hz, 1H, ArH), 8.50 (bp, 1H, NH), 8.24 (dd, *J* = 7.3, 1.0 Hz, 1H, ArH), 8.16 (d, *J* = 8.3 Hz, 1H, ArH), 8.05 (d, *J* = 7.7 Hz, 1H, ArH), 7.72–7.61 (m, 3H, ArH), 7.38 (d, *J* = 7.0 Hz, 1H, ArH), 7.11 (d, *J* = 7.2 Hz, 1H, ArH), 7.04 (t, *J* = 7.8 Hz, 1H, ArH), 4.42 (s, 2H, AlH). ^13^C NMR (101 MHz, DMSO) δ 152.22 (C_Gua_), 139.09 (ArC), 134.30 (ArC), 133.49 (ArC), 131.04 (ArC), 129.07 (ArC), 128.69 (ArC), 128.24 (ArC), 127.89 (ArC), 127.14 (ArC), 126.65 (ArC), 126.28 (ArC), 125.66 (ArC), 125.07 (ArC), 124.89 (ArC), 121.03 (ArC), 119.06 (ArC), 41.75 (AlC). FT IR: 3281, 3103 (N-H, Str), 3054 (C-H Ar, Str), 2920 (C-H Aliph, Str), 1746 (C-H Ar, Bend), 1619, 1592 (C=N, Str), 1532, 1506 (N-H, Bend), 1480, 1463, 1440 (-CH_2_, Bend) 1363, 1346, 1324 (S=O, -SO_2_, Str), 1293, 1278, 1246, 1229, (C-N Ar, Str), 1191, 1169 (C-N, Str), 1153, 1135 (S=O, sulfonamide, Str), 1091, 1028 (C-N, Str), 988, 953 (C=C Ar, Bend), 829, 809, 769, 733, 700, 681 (C-Cl, Str).

***N*** **-(5,6-dichloro-3,4-dihydroquinazolin-2-yl)naphthalene-1-sulfonamide PR50**

Prepared in method **B^1^**, Formula weight for C_18_H_13_Cl_2_N_3_O_2_S: 406.3 g/mol, UPLC-MS: [M + H]^+^ = 406.0, purity = 94%, R_t_ = 8.25 min, Y = 31%, mp = 233–240 °C. ^1^H NMR (400 MHz, DMSO) δ 8.82 (bp, 1H, NH), 8.67 (d, J = 8.4 Hz, 1H, ArH), 8.25 (t, J = 13.7 Hz, 1H, ArH), 8.20–8.12 (m, 2H, ArH, NH), 8.07 (dd, J = 14.2, 7.8 Hz, 2H, ArH), 7.69–7.64 (m, 3H, ArH), 7.12 (d, J = 8.7 Hz, 1H, ArH), 3.86–3.73 (m, 2H, AlH). ^13^C NMR (101 MHz, DMSO) δ 151.37 (C_Gua_), 144.55 (ArC), 139.72 (ArC), 134.23 (ArC), 133.46 (ArC), 129.25 (ArC), 128.38 (ArC), 128.15 (ArC), 128.01 (ArC), 127.88 (ArC), 127.16 (ArC), 126.91 (ArC), 125.55 (ArC), 124.93 (ArC), 118.46 (ArC), 113.94 (ArC), 63.65 (AlC). FT IR: 3301 (N-H, Str), 3086, 3062, 3016 (C-H Ar, Str), 2970, 2948, 2862 (C-H Aliph, Str), 1738 (C-H Ar, Bend), 1629, 1601, 1572 (C=N, Str), 1508 (N-H, Bend), 1470, 1433, 1408 (-CH_2_, Bend) 1383, 1349, 1311 (S=O, -SO_2_, Str), 1286, 1238, 1231, 1217, (C-N Ar, Str), 1178, 1168 (C-N, Str), 1155, 1144, 1134 (S=O, sulfonamide, Str), 1085, 1024, 1007 (C-N, Str), 974, 896 (C=C Ar, Bend), 833, 817, 803, 771, 746, 708, 679 (C-Cl, Str).

***N*** **-(6,8-dichloro-3,4-dihydroquinazolin-2-yl)naphthalene-1-sulfonamide PR51**

Prepared in method **B^1^**, Formula weight for C_18_H_13_Cl_2_N_3_O_2_S: 406.3 g/mol, UPLC-MS: [M + H]^+^ = 406.0, purity = 92%, R_t_ = 8.47 min, Y = 27%, mp = 214–219 °C. ^1^H NMR (400 MHz, DMSO) δ 9.80 (bp, 1H, NH), 8.75 (d, *J* = 8.5 Hz, 1H, ArH), 8.52 (bp, 1H, NH), 8.22 (dd, *J* = 7.3, 1.1 Hz, 1H, ArH), 8.17 (d, *J* = 8.2 Hz, 1H, ArH), 8.05 (d, *J* = 7.5 Hz, 1H, ArH), 7.72–7.57 (m, 4H, ArH), 7.28 (d, *J* = 1.8 Hz, 1H, ArH), 4.41 (s, 2H, AlH).^13^C NMR (101 MHz, DMSO) δ 151.96 (C_Gua_), 138.98 (ArC), 134.30 (ArC), 133.54 (ArC), 130.48 (ArC), 129.08 (ArC), 128.22 (ArC), 128.09 (ArC), 127.95 (ArC), 127.91 (ArC), 127.15 (ArC), 126.69 (ArC), 126.24 (ArC), 125.71 (ArC), 124.90 (ArC), 122.66 (ArC), 119.87 (ArC), 41.59 (ALC). FT IR: 3372, 3277, 3219 (N-H, Str), 3064 (C-H Ar, Str), 2920 (C-H Aliph, Str), 1716 (C-H Ar, Bend), 1628, 1588 (C=N, Str), 1519, 1505 (N-H, Bend), 1472, 1406 (-CH_2_, Bend) 1370, 1344, 1322 (S=O, -SO_2_, Str), 1276, 1248, 1212 (C-N Ar, Str), 1194, 1169 (C-N, Str), 1154, 1142 (S=O, sulfonamide, Str), 1091, 1024 (C-N, Str), 977, 974, 890 (C=C Ar, Bend), 858, 827, 801, 764, 733, 683 (C-Cl, Str).

**2-chloro-*N*-(5-chloro-3,4-dihydroquinazolin-2-yl)naphthalene-1-sulfonamide PR52**

Prepared in method **C^1^**, Formula weight for C_18_H_13_Cl_2_N_3_O_2_S: 406.3 g/mol, UPLC-MS: [M + H]^+^ = 406.1, purity = 99%, R_t_ = 8.28 min, Y = 45%, mp = 267–270 °C. ^1^H NMR (400 MHz, DMSO) δ 10.24 (bp, 1H, NH), 9.19 (d, *J* = 9.1 Hz, 1H, ArH), 8.11 (d, *J* = 8.6 Hz, 2H, ArH), 8.03 (d, *J* = 7.3 Hz, 1H, ArH), 7.74–7.68 (m, 1H, ArH), 7.63 (ddd, *J* = 9.0, 5.8, 1.7 Hz, 2H, ArH), 7.21 (t, *J* = 8.0 Hz, 1H, ArH), 7.09 (dd, *J* = 8.0, 0.9 Hz, 1H, ArH), 6.88–6.81 (m, 1H, NH), 4.56 (d, *J* = 1.2 Hz, 2H, AlH). ^13^C NMR (101 MHz, DMSO) δ 151.85 (C_Gua_), 137.00 (ArC), 136.63 (ArC), 133.82 (ArC), 132.99 (ArC), 131.80 (ArC), 130.88 (ArC), 130.84 (ArC), 129.99 (ArC), 129.42 (ArC), 129.09 (ArC), 128.38 (ArC), 127.03 (ArC), 126.75 (ArC), 123.80 (ArC), 116.57 (ArC), 114.11 (ArC), 41.26 (AlC). FT IR: 3368, 3304, 3101 (N-H, Str), 3055 (C-H Ar, Str), 2969, 2871 (C-H Aliph, Str), 1746 (C-H Ar, Bend), 1624, 1598, 1554 (C=N, Str), 1530, 1501 (N-H, Bend), 1473, 1431, 1422 (-CH_2_, Bend) 1376, 1361, 1327 (S=O, -SO_2_, Str), 1301, 1282, 1262, 1216 (C-N Ar, Str), 1202, 1178, 1164 (C-N, Str), 1147 (S=O, sulfonamide, Str), 1103, 1075, 1034, 1005 (C-N, Str), 976, 955, 947, 891 (C=C Ar, Bend), 865, 851, 822, 809, 783, 769, 740, 718, 704, 685 (C-Cl, Str).

**4-chloro-*N*-(5-chloro-3,4-dihydroquinazolin-2-yl)naphthalene-1-sulfonamide PR53**

Prepared in method **C^1^**, Formula weight for C_18_H_13_Cl_2_N_3_O_2_S: 406.3 g/mol, UPLC-MS: [M + H]^+^ = 106.1, purity = 100%, R_t_ = 8.61 min, Y = 42%, mp = 217–223 °C. ^1^H NMR (400 MHz, DMSO) δ 10.25 (bp, 1H, NH), 8.92–8.83 (m, 1H, ArH), 8.35–8.28 (m, 1H, ArH), 8.23 (d, J = 7.9 Hz, 1H, ArH), 8.02 (bp, 1H, NH), 7.89–7.77 (m, 3H, ArH), 7.17 (t, J = 8.0 Hz, 1H, ArH), 7.05 (dd, J = 8.1, 0.8 Hz, 1H, ArH), 6.83 (d, J = 8.0 Hz, 1H, ArH), 4.47 (d, J = 1.3 Hz, 2H, AlH). ^13^C NMR (101 MHz, DMSO) δ 151.83 (C_Gua_), 138.81 (ArC), 137.00 (ArC), 135.60 (ArC), 130.89 (ArC), 130.80 (ArC), 129.93 (ArC), 129.55 (ArC), 128.78 (ArC), 128.65 (ArC), 127.14 (2ArC), 125.54 (ArC), 124.83 (ArC), 123.70 (ArC), 116.42 (ArC), 114.02 (ArC), 41.38 (AlC). FT IR: 3289, 3173 (N-H, Str), 3046 (C-H Ar, Str), 2920 (C-H Aliph, Str), 1645, 1613, (C=N, Str), 1540, 1502 (N-H, Bend), 1481, 1444 (-CH_2_, Bend) 1367, 1338, 1306 (S=O, -SO_2_, Str), 1294, 1272, 1216 (C-N Ar, Str), 1199, 1162 (C-N, Str), 1107, 1093, 1035, 1016 (C-N, Str), 1000, 980, 909, 888 (C=C Ar, Bend), 857, 829, 779, 754, 725, 684, 665 (C-Cl, Str).

***N*** **-(5-fluoro-3,4-dihydroquinazolin-2-yl)naphthalene-1-sulfonamide PR54**

Prepared in method **B^2^**, Formula weight for C_18_H_14_FN_3_O_2_S: 355.4 g/mol, UPLC-MS: [M + H]^+^ = 356.2, purity = 95%, R_t_ = 7.07 min, Y = 75%, mp = 191–193 °C, Composition: C(60.83%) H(3.97%) F(5.35%) N(11.82%) O(9.00%) S(9.02%), found: C(60.66%) H(3.97%) N(11.80%), S(9.12%). ^1^H NMR (400 MHz, DMSO) δ 8.82 (d, *J* = 8.5 Hz, 1H, ArH), 8.21 (dd, *J* = 7.3, 1.1 Hz, 1H, ArH), 8.09 (d, *J* = 8.2 Hz, 1H, ArH), 8.01 (d, *J* = 7.4 Hz, 1H, ArH), 7.62 (ddd, *J* = 21.1, 11.2, 6.6 Hz, 3H, ArH), 7.08 (dd, *J* = 14.8, 7.6 Hz, 1H, ArH), 6.67 (t, *J* = 8.7 Hz, 1H, ArH), 6.54 (d, *J* = 7.6 Hz, 1H, ArH), 4.39 (s, 2H, AlH). ^13^C NMR (101 MHz, DMSO) δ 158.61 (d, J = 242.7 Hz, C-F), 134.19 (C_Gua_), 132.46 (ArC), 132.43 (ArC), 129.35 (d, J = 8.5 Hz, C-F), 129.31 (ArC), 128.81 (ArC), 128.63 (ArC), 127.30 (ArC), 126.95 (ArC), 126.77 (ArC), 126.71 (ArC), 124.89 (ArC), 124.81 (ArC), 106.40 (d, J = 20.5 Hz, C-F), 106.30 (ArC), 37.93 (AlC). FT IR: 3363, 3297 (N-H, Str), 3029 (C-H Ar, Str), 2970 (C-H Aliph, Str), 1738 (C-H Ar, Bend), 1635, 1612 (C=N, Str), 1537, 1506 (N-H, Bend), 1483, 1442 (-CH_2_, Bend) 1378, 1334 (S=O, -SO_2_, Str), 1278, 1255, 1237, 1217 (C-N Ar, Str), 1155, 1140 (C-N, Str), 1088, 1059, 1024, 1011 (C-N, Str), 980, 894 (C=C Ar, Bend), 829, 811, 798, 783, 766, 733, 690 (C-F, Str).

***N*** **-(6-fluoro-3,4-dihydroquinazolin-2-yl)naphthalene-1-sulfonamide PR55**

Prepared in method **B^2^**, Formula weight for C_18_H_14_FN_3_O_2_S: 355.4 g/mol, UPLC-MS: [M + H]^+^ = 356.2, purity = 94%, R_t_ = 6.79 min, Y = 75%, mp = 206–210 °C. ^1^H NMR (400 MHz, DMSO) δ 10.05 (bp, 1H, NH), 8.78 (d, *J* = 8.6 Hz, 1H, ArH), 8.24 (dd, *J* = 7.3, 1.1 Hz, 1H, ArH), 8.05 (d, *J* = 7.5 Hz, 1H, ArH), 7.95 (bp, 1H, NH), 7.69–7.61 (m, 4H, ArH), 7.06–6.99 (m, 2H, ArH), 6.89 (dd, *J* = 8.7, 4.9 Hz, 1H, ArH), 4.40 (s, 2H, AlH). ^13^C NMR (101 MHz, DMSO) δ 158.52 (d, *J* = 238.9 Hz, C-F), δ 152.37 (C_Gua_), 139.33 (ArC), 134.27 (ArC), 133.14 (ArC), 129.01 (ArC), 128.39 (ArC), 127.70 (ArC), 126.99 (ArC), 126.81 (ArC), 126.40 (ArC), 124.87 (ArC), 120.54 (ArC), 116.58 (d, J = 8.3 Hz, C-F), 115.19 (d, J = 23.0 Hz, C-F), 115.07 (ArC), 113.34 (ArC), 113.10 (ArC), 42.48 (AlC). FT IR: 3347, 3261, 3169 (N-H, Str), 3064 (C-H Ar, Str), 2919, 2850 (C-H Aliph, Str), 1734 (C-H Ar, Bend), 1639, 1615 (C=N, Str), 1540, 1499 (N-H, Bend), 1426 (-CH_2_, Bend) 1366, 1344, 1323 (S=O, -SO_2_, Str), 1283, 1260, 1234 (C-N Ar, Str), 1198, 1165, 1154, 1135 (C-N, Str), 1097, 1025 (C-N, Str), 980, 948, 911, 868 (C=C Ar, Bend), 839, 807, 771, 739, 728, 702, 684 (C-F, Str).

***N*** **-(7-fluoro-3,4-dihydroquinazolin-2-yl)naphthalene-1-sulfonamide PR56**

Prepared in method **B^2^**, Formula weight for C_18_H_14_FN_3_O_2_S: 355.4 g/mol, UPLC-MS: [M + H]^+^ = 356.2, purity = 97%, R_t_ = 6.96 min, Y = 56%, mp = 222–226 °C, Composition: C(60.83%) H(3.97%) F(5.35%) N(11.82%) O(9.00%) S(9.02%), found: C(60.51%) H(3.82%) N(11.86%), S(9.19%). ^1^H NMR (400 MHz, DMSO) δ 8.79 (d, *J* = 8.6 Hz, 1H, ArH), 8.25 (dd, *J* = 7.3, 1.0 Hz, 1H, ArH), 8.14 (d, *J* = 8.2 Hz, 1H, ArH), 8.04 (d, *J* = 7.8 Hz, 1H, ArH), 7.67 (ddd, *J* = 13.3, 7.4, 3.8 Hz, 1H, ArH), 7.62 (ddd, *J* = 8.1, 3.3, 1.9 Hz, 2H, ArH), 7.11 (dd, *J* = 8.2, 6.3 Hz, 1H, ArH), 6.76 (td, *J* = 8.7, 2.5 Hz, 1H, ArH), 6.66 (dd, *J* = 10.1, 2.3 Hz, 1H, ArH), 4.37 (s, 2H, AlH). ^13^C NMR (101 MHz, DMSO) 162.08 (d, J = 241.7 Hz), 152.59 (C_Gua_), 139.48 (ArC), 138.04 (d, J = 12.9 Hz, C-F), 134.25 (ArC), 133.05 (ArC), 128.98 (ArC), 128.43 (ArC), 127.99 (ArC), 127.64 (ArC), 126.94 (ArC), 126.45 (ArC), 124.86 (ArC), 114.81, (ArC) 114.79 (ArC), 109.67 (d, J = 21.8 Hz, C-F), 102.50 (d, J = 25.9 Hz, C-F), 42.23 (AlC). FT IR: 3280, 3184 (N-H, Str), 3064 (C-H Ar, Str), 2969 (C-H Aliph, Str), 1737 (C-H Ar, Bend), 1622 (C=N, Str), 1543, 1502 (N-H, Bend), 1464, 1451, 1414 (-CH_2_, Bend) 1373, 1343, 1323 (S=O, -SO_2_, Str), 1300, 1267, 1258, 1213 (C-N Ar, Str), 1172, 1146, 1132 (C-N, Str), 1095, 1068, 1026, 1013 (C-N, Str), 994, 979, 953, 924, 884, 868 (C=C Ar, Bend), 828, 803, 769, 719, 711, 694 (C-F, Str).

***N*** **-[6-(trifluoromethyl)-3,4-dihydroquinazolin-2-yl]naphthalene-1-sulfonamide PR57**

Prepared in method **B^1^**, Formula weight for C_19_H_14_F_3_N_3_O_2_S: 405.4 g/mol, UPLC-MS: [M + H]^+^ = 406.1, purity = 95%, R_t_ = 7.91 min, Y = 45%, mp = 215–222 °C. ^1^H NMR (400 MHz, DMSO) δ 10.35 (bp, 1H, NH), 8.78 (d, *J* = 8.7 Hz, 1H, ArH), 8.27 (dd, *J* = 7.3, 1.1 Hz, 1H, ArH), 8.15 (t, *J* = 7.5 Hz, 1H, ArH), 8.10–8.02 (m, 2H, NH, ArH), 7.69 (ddd, *J* = 8.5, 6.9, 1.4 Hz, 1H, ArH), 7.64 (dt, *J* = 6.1, 2.5 Hz, 2H, ArH), 7.56–7.49 (m, 2H, ArH), 7.03 (d, *J* = 8.2 Hz, 1H, ArH), 4.49 (s, 2H, AlH). ^13^C NMR (101 MHz, DMSO) δ 152.26 (C_Gua_), 139.25 (ArC), 139.04 (ArC), 134.27 (ArC), 133.29 (ArC), 129.05 (ArC), 128.37 (ArC), 127.78 (ArC), 127.03 (ArC), 126.98 (ArC), 126.32 (ArC), 125.85 (q, J = 3.4 Hz, C-F), 124.88 (ArC), 123.73 (d, J = 3.7 Hz, C-F), 123.43 (q, J = 9.5, C-F), 119.36 (ArC), 115.44 (ArC), 42.36 (AlC). FT IR: 3359, 3262, 3153 (N-H, Str), 3024 (C-H Ar, Str), 2970, 2920 (C-H Aliph, Str), 1739 (C-H Ar, Bend), 1637 (C=N, Str), 1505 (N-H, Bend), 1465, 1423 (-CH_2_, Bend) 1377, 1327 (S=O, -SO_2_, Str), 1286, 1262, 1233 (C-N Ar, Str), 1196, 1166, 1155, 1142, 1131 (C-N, Str), 1104, 1074 (C-N, Str), 981, 961, 934, 910, 891 (C=C Ar, Bend), 826, 806, 767,732, 709, 676 (C-F, Str).

***N*** **-(5-methoxy-3,4-dihydroquinazolin-2-yl)naphthalene-1-sulfonamide PR58**

Prepared in method **B^1^**, Formula weight for C_19_H_17_N_3_O_3_S: 367.4 g/mol, UPLC-MS: [M + H]^+^ = 368.2, purity = 99%, R_t_ = 7.30 min, Y = 43%, mp = 245–249 °C, Composition: C(62.11%) H(4.66%) N(11.44%) O(13.06%) S(8.73%), found: C(61.97%) H(4.61%) N(11.49%) S(8.79%). ^1^H NMR (400 MHz, DMSO) δ 9.95 (bp, 1H, NH), 8.79 (bp, *J* = 8.1 Hz, 1H, ArH), 8.23 (dd, *J* = 7.3, 1.1 Hz, 1H, ArH), 8.14 (d, *J* = 8.2 Hz, 1H, ArH), 8.04 (d, *J* = 7.6 Hz, 1H, ArH), 7.92 (bp, 1H, NH), 7.68 (ddd, *J* = 8.5, 6.9, 1.4 Hz, 1H, ArH), 7.65–7.59 (m, 2H, ArH), 7.10 (t, *J* = 8.2 Hz, 1H, ArH), 6.60 (d, *J* = 8.1 Hz, 1H, ArH), 6.47 (d, *J* = 7.9 Hz, 1H, ArH), 4.33 (s, 2H, AlH), 3.74 (s, 3H, OCH_3_). ^13^C NMR (101 MHz, DMSO) δ 155.61 (C_Gua_), 152.01 (ArC), 139.42 (ArC), 136.16 (ArC), 134.27 (ArC), 133.10 (ArC), 129.32 (ArC), 129.00 (ArC), 128.40 (ArC), 127.70 (ArC), 126.98 (ArC), 126.74 (ArC), 126.40 (ArC), 124.87 (ArC), 107.82 (ArC), 105.95 (ArC), 105.88 (ArC), 55.97 (OCH_3_), 38.85 (AlC). FT IR: 3327, 3275 (N-H, Str), 3001 (C-H Ar, Str), 2959, 2933, 2854, 2835 (C-H Aliph, Str), 1604 (C=N, Str), 1528, 1506 (C=C Ar, Str), 1484, 1460, 1430 (-CH_2_, Bend) 1375, 1338, 1304 (S=O, -SO_2_, Str), 1273 (C-O, Str), 1251, 1229 (C-N Ar, Str), 1159, 1144 (S=O, sulfonamide, Str), 1113, 1096, 1025, 1009 (C-N, Str), 956, 882 (C=C Ar, Bend).

***N*** **-(6-methoxy-3,4-dihydroquinazolin-2-yl)naphthalene-1-sulfonamide PR59**

Prepared in method **B^1^**, Formula weight for C_19_H_17_N_3_O_3_S: 367.4 g/mol, UPLC-MS: [M + H]^+^ = 368.2, purity = 96%, R_t_ = 6.73 min, Y = 68%, mp = 263–266 °C. ^1^H NMR (400 MHz, DMSO) δ 9.89 (bp, 1H, NH), 8.79 (d, *J* = 8.4 Hz, 1H, ArH), 8.23 (d, *J* = 7.3 Hz, 1H, ArH), 8.14 (d, *J* = 8.2 Hz, 1H, ArH), 8.04 (d, *J* = 7.8 Hz, 1H, ArH), 7.87 (bp, 1H, NH), 7.72–7.56 (m, 3H, ArH), 6.82 (d, *J* = 9.1 Hz, 1H, ArH), 6.78–6.69 (m, 2H, ArH), 4.38 (s, 2H, AlH), 3.67 (s, 3H, OCH_3_). ^13^C NMR (101 MHz, DMSO) δ 155.86 (C_Gua_), 152.34 (ArC), 139.54 (ArC), 134.27 (ArC), 133.03 (ArC), 128.98 (ArC), 128.64 (ArC), 128.41 (ArC), 127.64 (ArC), 126.96 (ArC), 126.72 (ArC), 126.46 (ArC), 124.87 (ArC), 119.65 (ArC), 116.26 (ArC), 114.16 (ArC), 111.50 (ArC), 55.78 (OCH_3_), 42.74 (ArC). FT IR: 3367, 3322, 3244, 3155 (N-H, Str), 3082, 3043, 3009 (C-H Ar, Str), 2960, 2870 (C-H Aliph, Str), 1629, 1615 (C=N, Str), 1568 (N-H, Bend), 1532 (C=C Ar, Str), 1498, 1444, 1421 (-CH_2_, Bend) 1359, 1344, 1321 (S=O, -SO_2_, Str), 1279 (C-O, Str), 1243 (C-N Ar, Str), 1197 (C-N, Str), 1171, 1153, 1143 (S=O, sulfonamide, Str), 1097, 1035 (C-N, Str), 978, 963, 939, 904 (C=C Ar, Bend).

***N*** **-(7-methoxy-3,4-dihydroquinazolin-2-yl)naphthalene-1-sulfonamide PR60**

Prepared in method **B^2^**, Formula weight for C_19_H_17_N_3_O_3_S: 367.4 g/mol, UPLC-MS: [M + H]^+^ = 368.2, purity = 100%, R_t_ = 7.07 min, Y = 65%, mp = 213–217 °C, ^1^H NMR (400 MHz, DMSO) δ 9.93 (bp, 1H, NH), 8.79 (d, *J* = 8.5 Hz, 1H, ArH), 8.24 (dd, *J* = 7.3, 1.1 Hz, 1H, ArH), 8.15 (d, *J* = 8.4 Hz, 1H, ArH), 8.04 (d, *J* = 7.7 Hz, 1H, ArH), 7.95 (bp, 1H, NH), 7.72–7.68 (m, 1H, ArH), 7.67–7.64 (m, 1H, ArH), 7.63–7.61 (m, 1H, ArH), 7.00 (d, *J* = 8.4 Hz, 1H, ArH), 6.55 (dd, *J* = 8.4, 2.5 Hz, 1H, ArH), 6.49 (d, *J* = 2.5 Hz, 1H, ArH), 4.33 (s, 2H, AlH), 3.67 (s, 3H, OCH_3_). ^13^C NMR (101 MHz, DMSO) δ 159.56 (C_Gua_), 152.48 (ArC), 139.36 (ArC), 136.41 (ArC), 134.27 (ArC), 133.13 (ArC), 129.01 (ArC), 128.39 (ArC), 127.69 (ArC), 127.22 (ArC), 126.99 (ArC), 126.79 (ArC), 126.41 (ArC), 124.87 (ArC), 110.47 (ArC), 109.11 (ArC), 101.03 (ArC), 55.58 (OCH_3_), 42.14 (AlC). FT IR: 3328 (N-H, Str), 3081 (C-H Ar, Str), 2992, 2970, 2835 (C-H Aliph, Str), 1739 (C-H Ar, Bend), 1623 (C=N, Str), 1552 (N-H, Bend), 1504 (C=C Ar, Str), 1458, 1438 (-CH_2_, Bend) 1362, 1334 (S=O, -SO_2_, Str), 1295 (C-O, Str), 1256 (C-N Ar, Str), 1219 (C-N, Str), 1197, 1155, 1140, 1123 (S=O, sulfonamide, Str), 1094, 1046, 1025 (C-N, Str), 989, 949, 922, 846, 828 (C=C Ar, Bend).

***N*** **-(5-methyl-3,4-dihydroquinazolin-2-yl)naphthalene-1-sulfonamide PR61**

Prepared in method **B^1^**, Formula weight for C_19_H_17_N_3_O_2_S: 351.4 g/mol, UPLC-MS: [M + H]^+^ = 352.2, purity = 99%, R_t_ = 7.41 min, Y = 32%, mp = 233–237 °C. ^1^H NMR (400 MHz, DMSO) δ 9.88 (bp, 1H, NH), 8.80 (d, *J* = 8.5 Hz, 1H, ArH), 8.25 (d, *J* = 6.8 Hz, 1H, ArH), 8.14 (d, *J* = 8.2 Hz, 1H, ArH), 8.04 (d, *J* = 7.9 Hz, 1H, ArH), 7.68 (t, *J* = 7.1 Hz, 1H, ArH), 7.65–7.58 (m, 2H, ArH), 7.01 (t, *J* = 7.7 Hz, 1H, ArH), 6.78 (d, *J* = 7.5 Hz, 1H, ArH), 6.69 (d, *J* = 7.9 Hz, 1H, ArH), 4.38 (s, 2H, AlH), 2.08 (s, 3H, AlH). ^13^C NMR (101 MHz, DMSO) δ 152.04 (C_Gua_), 139.45 (ArC), 135.13 (ArC), 134.86 (ArC), 134.28 (ArC), 133.08 (ArC), 128.99 (ArC), 128.42 (ArC), 128.14 (ArC), 127.67 (ArC), 126.97 (ArC), 126.81 (ArC), 126.44 (ArC), 124.91 (ArC), 124.87 (ArC), 116.81 (ArC), 112.98 (ArC), 41.02 (AlC), 18.26 (AlC). FT IR: 3356, 3299, 3250 (N-H, Str), 3049 (C-H Ar, Str), 2967, 2861 (C-H Aliph, Str) 1630, 1607 (C=N, Str), 1544, 1504 (N-H, Bend), 1484, 1446 (-CH_2_, Bend), 1374 (-CH_3_, Bend), 1328 (S=O, -SO_2_, Str), 1294, 1270, 1250, 1215 (C-N Ar, Str), 1201 (C-N amine, Str) 1173, 1154, 1139, (S=O, sulfonamide, Str), 1083, 1025, 1012 (C-N, Str), 993, 976, 889 (C=C Ar, Bend)

***N*** **-(7-methyl-3,4-dihydroquinazolin-2-yl)naphthalene-1-sulfonamide PR62**

Prepared in method **B^1^**, Formula weight for C_19_H_17_N_3_O_2_S: 351.4 g/mol, UPLC-MS: [M + H]^+^ = 352.2, purity = 93%, R_t_ = 7.25 min, Y = 43%, mp = 166–175 °C. ^1^H NMR (400 MHz, DMSO) δ 9.96 (bp, 1H, NH), 8.80 (t, *J* = 9.3 Hz, 1H, ArH), 8.23 (dt, *J* = 11.2, 5.6 Hz, 1H, ArH), 8.14 (d, *J* = 8.3 Hz, 1H, ArH), 8.04 (d, *J* = 7.5 Hz, 1H, ArH), 7.91 (bp, 1H, NH), 7.70–7.59 (m, 3H, ArH), 6.96 (d, *J* = 7.7 Hz, 1H, ArH), 6.77 (d, *J* = 7.7 Hz, 1H, ArH), 6.69 (s, 1H, ArH), 4.36 (s, 2H, AlH), 2.20 (d, *J* = 9.5 Hz, 3H, AlH). ^13^C NMR (101 MHz, DMSO) δ 152.62 (C_Gua_), 139.39 (ArC), 137.91 (ArC), 135.29 (ArC) 134.27 (ArC), 133.11 (ArC), 129.00 (ArC), 128.40 (ArC), 127.69 (ArC), 126.98 (ArC), 126.79 (ArC), 126.40 (ArC), 126.13 (ArC), 124.87 (ArC), 124.30 (ArC), 115.49 (2ArC), 42.39 (AlC), 21.24 (AlC). FT IR: 3292 (N-H, Str), 3062 (C-H Ar, Str), 2918, 2849 (C-H Aliph, Str), 1729 (C-H Ar, Bend), 1628 (C=N, Str), 1587, 1542, 1505 (N-H, Bend), 1455 (-CH_3_, Bend), 1414 (-CH_2_, Bend), 1358, 1329 (S=O, -SO_2_, Str), 1280, 1262, 1212 (C-N Ar, Str), 1185, 1152, 1124, 1101 (S=O, sulfonamide, Str), 1065, 1036, 1026 (C-N, Str), 983, 908, 868 (C=C Ar, Bend).

***N*** **-[5-(dimethylamino)-3,4-dihydroquinazolin-2-yl]naphthalene-1-sulfonamide PR63**

Prepared in method **B^1^**, Formula weight for C_20_H_20_N_4_O_2_S: 380.5 g/mol, UPLC-MS: [M + H]^+^ = 381.2, purity = 94%, R_t_ = 7.27 min, Y = 41%,^1^H NMR (400 MHz, DMSO) δ 9.82 (bp, 1H, NH), 8.52 (ddq, *J* = 8.0, 1.8, 0.5 Hz, 1H, ArH), 8.04–7.98 (m, 2H, ArH), 7.93 (dtt, *J* = 7.5, 1.5, 0.4 Hz, 1H, ArH), 7.82 (bp, 1H, ArH), 7.88–7.63 (m, 4H, ArH), 7.18–7.12 (m, 2H, ArH), 6.57 (dd, *J* = 7.8, 1.5 Hz, 1H, ArH), 4.35 (d, J = 18.1 Hz, 2H, AlH), 2.81 (s, 6H, AlH). ^13^C NMR (101 MHz, DMSO) δ 153.12 (C_Gua_), 146.34 (ArC), 140.32 (ArC), 136.23 (ArC), 129.23 (ArC), 129.12 (ArC), 128. 45 (ArC), 127.12 (ArC), 127.01 (ArC), 126.68 (ArC), 126.22 (ArC), 126.01 (ArC), 125.99 (ArC), 125.52 (ArC), 124.12 (ArC), 118.23 (ArC), 116.64 (ArC), 45.34 (AlC), 40.30 (AlC).

***N*** **-[6-(dimethylamino)-3,4-dihydroquinazolin-2-yl]naphthalene-1-sulfonamide PR64**

Prepared in method **B^1^**, Formula weight for C_20_H_20_N_4_O_2_S: 380.5 g/mol, UPLC-MS: [M + H]^+^ = 381.3, purity = 94%, R_t_ = 5.01 min, Y = 43%, mp = 216–222 °C, Composition: C(63.14%) H(5.30%) N(14.73%) O(8.41%) S(8.43%), found: C(63.32%) H(5.16%) N(14.71%) S(8.41%). ^1^H NMR (400 MHz, DMSO) δ 9.76 (bp, 1H, NH), 8.80 (d, *J* = 8.6 Hz, 1H, ArH), 8.23 (dd, *J* = 7.3, 1.1 Hz, 1H, ArH), 8.13 (d, *J* = 8.2 Hz, 1H, ArH), 8.04 (d, *J* = 7.5 Hz, 1H, ArH), 7.80 (bp, 1H, ArH), 7.65 (dddd, *J* = 10.3, 7.9, 6.2, 2.8 Hz, 4H, ArH), 6.76 (d, *J* = 8.7 Hz, 1H, ArH), 6.49 (s, 1H, ArH), 4.35 (s, 2H, AlH), 2.81 (d, *J* = 11.8 Hz, 6H, AlH). ^13^C NMR (101 MHz, DMSO) δ 152.16 (C_Gua_), 139.73 (ArC), 134.27 (ArC), 133.45 (ArC), 132.94 (ArC), 129.25 (ArC), 128.96 (ArC), 128.44 (ArC), 128.01 (ArC), 127.60 (ArC), 126.94 (ArC), 126.64 (ArC), 126.51 (ArC), 125.54 (ArC), 124.86 (ArC), 119.13 (ArC), 116.05 (ArC), 42.97 (AlC), 41.00 (AlC). FT IR: 3365, 3333, 3302, 3167 (N-H, Str), 3029 (C-H Ar, Str), 2970, 2921, 2802 (C-H Aliph, Str), 1738 (C-H Ar, Bend), 1615 (C=N, Str), 1544, 1520 (N-H, Bend), 1448, 1370 (-CH_3_, Bend), 1324 (S=O, -SO_2_, Str), 1281, 1261, 1233, 1206 (C-N Ar, Str), 1154, 1123, 1100 (S=O, sulfonamide, Str), 1069, 1026, 1010 (C-N, Str), 977, 962, 931, 890 (C=C Ar, Bend).

***N*** **-[6-(morpholin-4-yl)-3,4-dihydroquinazolin-2-yl]naphthalene-1-sulfonamide PR65**

Prepared in method **B^1^**, Formula weight for C_22_H_22_N_4_O_3_S: 422.5 g/mol, UPLC-MS: [M + H]^+^ = 423.2, purity = 99%, R_t_ = 6.46 min, Y = 81%, mp = 278–281 °C. ^1^H NMR (400 MHz, DMSO) δ 9.84 (bp, *J* = 1.5 Hz, 1H, NH), 8.80 (d, *J* = 8.6 Hz, 1H, ArH), 8.23 (dd, *J* = 7.3, 1.1 Hz, 1H, ArH), 8.13 (d, *J* = 8.3 Hz, 1H, ArH), 8.05 (t, *J* = 9.8 Hz, 1H, ArH), 7.84 (bp, 1H, NH), 7.68 (ddd, *J* = 8.5, 6.9, 1.4 Hz, 1H, ArH), 7.65–7.58 (m, 2H, ArH), 6.82–6.73 (m, 2H, ArH), 6.70 (s, 1H, ArH), 4.36 (s, 2H, AlH), 3.76–3.62 (m, 4H, AlH), 3.08–2.89 (m, 4H, AlH). ^13^C NMR (101 MHz, DMSO) δ 152.28 (C_Gua_), 147.94 (ArC), 139.61 (ArC), 134.27 (ArC), 133.00 (ArC), 128.97 (ArC), 128.43 (ArC), 127.70 (ArC), 127.63 (ArC), 126.95 (ArC), 126.70 (ArC), 126.48 (ArC), 124.87 (ArC), 119.06 (ArC), 115.91 (ArC), 115.62 (ArC), 113.12 (ArC), 66.49 (2AlC), 49.41 (2AlC), 42.90 (AlC). FT IR: 3293, 3102 (N-H, Str), 3053 (C-H Ar, Str), 2963, 2906, 2885, 2845 (C-H Aliph, Str), 1615 (C=N, Str), 1533, 1505 (N-H, Bend), 1448, 1431, 1373 (-CH_3_, Bend), 1320 (S=O, -SO_2_, Str), 1274, 1241, 1228 (C-N Ar, Str), 1198, 1183, 1148, 1122 (S=O, sulfonamide, Str), 1091, 1049, 1025 (C-N, Str), 984, 973, 951, 927, 880, 860 (C=C Ar, Bend)

***N*** **-[6-(4-methylpiperazin-1-yl)-3,4-dihydroquinazolin-2-yl]naphthalene-1-sulfonamide PR66**

Prepared in method **B^1^**, Formula weight for C_23_H_25_N_5_O_2_S: 435.5 g/mol, UPLC-MS: [M + H]^+^ = 436.4, purity = 91%, R_t_ = 6.12 min, Y = 43%, mp = oil. ^1^H NMR (400 MHz, DMSO) δ 8.84 (d, *J* = 8.6 Hz, 1H, ArH), 8.21 (dd, *J* = 7.3, 1.5 Hz, 1H, ArH), 8.18 (d, *J* = 8.1 Hz, 1H, ArH), 8.00 (t, *J* = 9.8 Hz, 1H, ArH), 7.69–7.59 (m, 3H, ArH), 6.82–6.73 (m, 2H, ArH), 6.65 (s, 1H, ArH), 4.36 (s, 2H, AlH), 3.74–3.61 (m, 4H, AlH), 3.08–2.91 (m, 4H, AlH), 2.13–1.99 (m, 3H, AlH). ^13^C NMR (101 MHz, DMSO) δ 151.12 (C_Gua_), 143.12 (ArC), 138.62 (ArC), 133.21 (ArC), 133.20 (ArC), 129.23 (ArC), 128.12 (ArC), 127.72 (ArC), 127.06 (ArC), 126.12 (ArC), 126.01(ArC), 125.21 (ArC), 124.43 (ArC), 119.12 (ArC), 116.44 (ArC), 115.62 (ArC), 113.15 (ArC), 63.41 (2AlC), 44.34 (2AlC), 41.23(AlC), 28.12(AlC).

***N*** **-(4-methyl-3,4-dihydroquinazolin-2-yl)naphthalene-1-sulfonamide PR67**

Prepared in method **C^2^**, Formula weight for C_19_H_17_N_3_O_2_S: 351.4 g/mol, UPLC-MS: [M + H]^+^ =352.3, purity = 95%, R_t_ = 7.07 min, Y = 55%. mp = 169–174 °C. ^1^H NMR (400 MHz, DMSO) δ 10.12 (s, 1H, NH), 8.81 (d, J = 8.5 Hz, 1H, ArH), 8.25 (d, J = 7.2 Hz, 1H, ArH), 8.16 (d, J = 8.1 Hz, 1H, ArH), 8.04–7.96 (m, 2H, ArH), 7.73–7.53 (m, 3H, ArH), 7.17–7.12 (m, 2H, ArH), 6.97 (dd, J = 16.0, 7.7 Hz, 2H, ArH), 6.89 (d, J = 7.9 Hz, 1H, ArH), 4.68 (dd, J = 6.5, 3.3 Hz, 1H, AlH), 1.06 (d, J = 6.5 Hz, 3H, AlH).^13^C NMR (101 MHz, DMSO) δ 151.63 (C_Gua_), 139.34 (ArC), 134.74 (ArC), 134.25 (ArC), 134.18 (ArC), 133.15 (ArC), 129.02 (ArC), 128.49 (ArC), 127.65 (ArC), 127.08 (ArC), 126.98 (ArC), 126.37 (ArC), 126.10 (ArC), 124.80 (ArC), 123.77 (ArC), 123.60 (ArC), 115.29 (ArC), 48.73 (AlC), 25.03 (AlC). FT IR: 3316, 3217 (N-H, Str), 3028, 3016 (C-H Ar, Str), 2970, 2862 (C-H Aliph, Str), 1738 (C-H Ar, Bend), 1622 (C=N, Str), 1534 (N-H, Bend), 1489, 1454 (-CH_2_, Bend), 1366 (-CH_3_, Bend), 1327 (S=O, -SO_2_, Str), 1279, 1229, 1217 (C-N Ar, Str), 1154, (S=O, sulfonamide, Str), 1099, 1084, 1034, 1002 (C-N, Str), 936, 915, 839 (C=C Ar, Bend).

***N*** **-(5-chloro-4-methyl-3,4-dihydroquinazolin-2-yl)naphthalene-1-sulfonamide PR68**

Prepared in method **C^2^**, Formula weight for C_19_H_16_ClN_3_O_2_S: 385.9 g/mol, UPLC-MS: [M + H]^+^ = 386.2, purity = 90%, R_t_ = 7.97 min, Y = 85%, mp = 144–149 °C, Composition: C(59.14%) H(4.18%) Cl(9.19%) N(10.89%) O(8.29%) S(8.31%), found: C(59.22%) H(4.09%) N(10.82%) S(8.39%). ^1^H NMR (400 MHz, DMSO) δ 10.43 (bp, *J* = 1.7 Hz, 1H, NH), 8.79 (d, *J* = 8.6 Hz, 1H, ArH), 8.33–8.26 (m, 1H, ArH), 8.20 (bp, *J* = 10.1 Hz, 1H, NH), 8.15 (t, *J* = 7.4 Hz, 1H, ArH), 8.05 (d, *J* = 7.5 Hz, 1H, ArH), 7.68 (ddd, *J* = 8.5, 6.9, 1.5 Hz, 1H, ArH), 7.66–7.59 (m, 2H, ArH), 7.25–7.17 (m, 1H, ArH), 7.12–7.06 (m, 1H, ArH), 6.89 (dd, *J* = 8.0, 0.8 Hz, 1H, ArH), 4.92–4.77 (m, 1H, AlH), 0.97 (d, J = 6.5 Hz, 3H, AlH). ^13^C NMR (101 MHz, DMSO) δ 151.76 (C_Gua_), 139.06 (ArC), 136.36 (ArC), 134.25 (ArC), 133.30 (ArC), 130.13 (ArC), 129.97 (ArC), 129.05 (ArC), 128.40 (ArC), 127.71 (ArC), 127.28 (ArC), 127.02 (ArC), 126.29 (ArC), 124.79 (ArC), 124.03 (ArC), 121.39 (ArC), 114.47 (ArC), 46.91 (AlC), 22.14 (AlC). FT IR: 3369, 3329, 3258, 3184, 3111 (N-H, Str), 3084, 3016 (C-H Ar, Str), 2970, 2927, 2859 (C-H Aliph, Str), 1739 (C-H Ar, Bend), 1622, 1594 (C=N, Str), 1572, 1548, 1532 (N-H, Bend), 1464, 1446 (-CH_2_, Bend) 1375, 1362, 1346, 1318 (S=O, -SO_2_, Str), 1272, 1252 (C-N Ar, Str), 1228, 1215, 1193 (C-N, Str), 1154 (S=O, sulfonamide, Str), 1138, 1096, 1044, 1008 (C-N, Str), 977, 951, 934 (C=C Ar, Bend), 849, 826, 802, 784, 768,753, 734, 686, 680 (C-Cl, Str).

***N*** **-(6-chloro-4-methyl-3,4-dihydroquinazolin-2-yl)naphthalene-1-sulfonamide PR69**

Prepared in method **C^2^**, Formula weight for C_19_H_16_ClN_3_O_2_S: 385.9 g/mol, UPLC-MS: [M + H]^+^ = 386.1, purity = 92%, R_t_ = 7.94 min, Y = 65%, mp = 144–153 °C. ^1^H NMR (400 MHz, DMSO) δ 9.09 (d, *J* = 8.9 Hz, 1H, NH), 8.15 (d, *J* = 8.7 Hz, 1H, ArH), 8.08–8.04 (m, 1H, ArH), 7.90 (s, 2H, ArH), 7.75–7.62 (m, 3H, ArH), 7.16 (t, *J* = 7.9 Hz, 1H, ArH), 7.05 (dd, *J* = 8.0, 0.9 Hz, 1H), 6.86 (d, *J* = 7.8 Hz, 1H, ArH), 4.68 (s, 1H, AlH), 1.18 (d, *J* = 6.4 Hz, 3H, AlH). ^13^C NMR (101 MHz, DMSO) δ 136.78 (C_Gua_), 134.12 (2ArC), 132.97 (ArC), 131.80 (ArC), 130.40 (ArC), 130.13 (ArC), 129.34 (2ArC), 129.28 (2ArC), 129.20 (ArC), 128.69 (2ArC), 127.13 (ArC), 125.95 (ArC), 124.00 (ArC), 22.80 (AlC), 12.99 (AlC). FT IR: 3377, 3276 (N-H, Str), 3099, 3014 (C-H Ar, Str), 2970, 2927 (C-H Aliph, Str), 1738 (C-H Ar, Bend), 1621, 1596 (C=N, Str), 1568, 1525, 1506 (N-H, Bend), 1488, 1450 (-CH_2_, Bend) 1365, 1349, 1322 (S=O, -SO_2_, Str), 1258 (C-N Ar, Str), 1217, 1202 (C-N, Str), 1157, 1145 (S=O, sulfonamide, Str), 1130, 1089, 1030 (C-N, Str), 992, 979, 928, 911 (C=C Ar, Bend), 824, 804, 792, 766, 738, 719, 677 (C-Cl, Str).

***N*** **-(7-chloro-4-methyl-3,4-dihydroquinazolin-2-yl)naphthalene-1-sulfonamide PR70**

Prepared in method **C^2^**, Formula weight for C_19_H_16_ClN_3_O_2_S: 385.7 g/mol, UPLC-MS: [M + H]^+^ =386.2, purity = 100%, R_t_ = 7.74 min, Y = 76%, mp = 214–219 °C. ^1^H NMR (400 MHz, DMSO) δ 10.25 (bp, 1H, NH), 8.78 (d, *J* = 8.6 Hz, 1H, ArH), 8.28 (dd, *J* = 7.3, 1.1 Hz, 1H, ArH), 8.16 (d, *J* = 8.3 Hz, 1H, ArH), 8.11 (bp, 1H, NH), 8.05 (d, *J* = 7.5 Hz, 1H, ArH), 7.68 (ddd, *J* = 8.5, 6.9, 1.5 Hz, 1H, ArH), 7.66–7.60 (m, 2H, ArH), 7.18 (d, *J* = 8.2 Hz, 1H, ArH), 7.02 (dd, *J* = 8.2, 2.1 Hz, 1H, ArH), 6.93 (d, *J* = 2.1 Hz, 1H, ArH), 4.73–4.63 (m, 1H, AlH), 1.06 (d, *J* = 6.5 Hz, 3H, AlH).^13^C NMR (101 MHz, DMSO) δ 151.35 (C_Gua_), 139.11 (ArC), 135.82 (ArC), 134.25 (ArC), 133.27 (ArC), 132.64 (ArC), 129.05 (ArC), 128.38 (ArC), 127.99 (ArC), 127.72 (ArC), 127.17 (ArC), 127.02 (ArC), 126.28 (ArC), 124.80 (ArC), 123.37 (ArC), 122.54 (ArC), 114.77 (ArC), 48.44 (AlC), 24.96 (AlC). FT IR: 3296 (N-H, Str), 3065 (C-H Ar, Str), 2970, 2919, 2850 (C-H Aliph, Str), 1738 (C-H Ar, Bend), 1615 (C=N, Str), 1532, 1507, 1489 (N-H, Bend), 1373, 1352, 1311 (S=O, -SO_2_, Str), 1293, 1255 (C-N Ar, Str), 1206 (C-N, Str), 1155 (S=O, sulfonamide, Str), 1097, 1026, 1000 (C-N, Str), 977, 943, 919 (C=C Ar, Bend), 825, 803, 776, 757, 738, 705, 681 (C-Cl, Str).

***N*** **-(8-chloro-4-methyl-3,4-dihydroquinazolin-2-yl)naphthalene-1-sulfonamide PR71**

Prepared in method **C^2^**, Formula weight for C_19_H_16_ClN_3_O_2_S: 385.9 g/mol, UPLC-MS: [M + H]^+^ = 386.2, purity = 91%, R_t_ = 8.04 min, Y = 64%, mp = 215–219 °C. Composition: C(59.14%) H(4.18%) Cl(9.19%) N(10.89%) O(8.29%) S(8.31%), found: C(58.89%) H(4.21%) N(10.79%) S(8.24%). ^1^H NMR (400 MHz, DMSO) δ 9.86 (bp, 1H, NH), 8.79 (d, J = 8.4 Hz, 1H, ArH), 8.54 (bp, 1H, NH), 8.25 (d, J = 7.2 Hz, 1H, ArH), 8.14 (d, J = 7.9 Hz, 1H, ArH), 8.03 (d, J = 7.8 Hz, 1H, ArH), 7.72–7.59 (m, 3H, ArH), 7.37 (d, J = 7.2 Hz, 1H, ArH), 7.13 (s, 1H, ArH), 7.03 (s, 1H, ArH), 4.65 (d, J = 5.9 Hz, 1H, AlH), 1.27 (d, J = 6.2 Hz, 3H, AlH). ^13^C NMR (101 MHz, DMSO) δ 151.49 (C_Gua_), 139.24 (ArC), 134.28 (ArC), 133.45 (ArC), 133.36 (ArC), 129.24 (ArC), 129.04 (ArC), 128.68 (ArC), 128.30 (ArC), 128.01 (ArC), 127.81 (ArC), 127.08 (ArC), 126.77 (ArC), 126.32 (ArC), 126.04 (ArC), 125.25 (ArC), 124.87 (ArC), 48.25 (AlC), 24.09 (AlC). FT IR: 3259, 3213 (N-H, Str), 3054 (C-H Ar, Str), 2970 (C-H Aliph, Str), 1738 (C-H Ar, Bend), 1619 (C=N, Str), 1529, 1506, 1479, 1450 (N-H, Bend), 1356, 1343, 1331 (S=O, -SO_2_, Str), 1280, 1267, 1250, 1225 (C-N Ar, Str), 1196, 1170 (C-N, Str), 1144 (S=O, sulfonamide, Str), 1093, 1063, 1025, 1006 (C-N, Str), 974, 892, 873 (C=C Ar, Bend), 824, 806, 772, 729, 706, 677 (C-Cl, Str).

***N*** **-(6,8-dichloro-4-methyl-3,4-dihydroquinazolin-2-yl)naphthalene-1-sulfonamide PR72**

Prepared in method **C^2^**, Formula weight for C_19_H_15_Cl_2_N_3_O_2_S: 420.3 g/mol, UPLC-MS: [M + H]^+^ = 420.0, purity = 91%, R_t_ = 8.80 min, Y = 42%, mp = 109–116 °C. ^1^H NMR (400 MHz, DMSO) δ 9.91 (bp, *J* = 1.0 Hz, 1H, NH), 8.76 (d, *J* = 8.6 Hz, 1H, ArH), 8.64 (bp, 1H, NH), 8.24 (dd, *J* = 7.3, 1.1 Hz, 1H, ArH), 8.15 (d, *J* = 8.3 Hz, 1H, ArH), 8.04 (d, *J* = 7.7 Hz, 1H, ArH), 7.69 (ddd, *J* = 8.5, 6.9, 1.4 Hz, 1H, ArH), 7.65–7.59 (m, 2H, ArH), 7.57 (d, *J* = 2.2 Hz, 1H, ArH), 7.33 (d, *J* = 2.1 Hz, 1H, ArH), 4.77–4.53 (m, 1H, AlH), 1.27 (d, *J* = 6.6 Hz, 3H, AlH). ^13^C NMR (101 MHz, DMSO) δ 151.08 (C_Gua_), 138.97 (ArC), 134.28 (ArC), 133.54 (ArC), 129.49 (ArC), 129.07 (ArC), 128.23 (ArC), 128.18 (ArC), 128.16 (ArC), 127.91 (ArC), 127.37 (ArC), 127.14 (ArC), 126.77 (ArC), 126.21 (ArC), 125.45 (ArC), 124.87 (ArC), 119.98 (ArC), 48.12 (AlC), 23.93 (AlC). FT IR: 3270 (N-H, Str), 3083, 3055 (C-H Ar, Str), 2970 (C-H Aliph, Str), 1738 (C-H Ar, Bend), 1615 (C=N, Str), 1506, 1481, 1445, 1404 (N-H, Bend), 1362, 1322, 1299 (S=O, -SO_2_, Str), 1281, 1250 (C-N Ar, Str), 1215, 1199 (C-N, Str), 1154 (S=O, sulfonamide, Str), 1102, 1058, 1025, 1004 (C-N, Str), 976, 956 (C=C Ar, Bend), 827, 798, 769, 733, 683, 669 (C-Cl, Str).

***N*** **-(5-fluoro-4-methyl-3,4-dihydroquinazolin-2-yl)naphthalene-1-sulfonamide PR73**

Prepared in method **C^2^**, Formula weight for C_19_H_16_FN_3_O_2_S: 369.4 g/mol, UPLC-MS: [M + H]+ = 370.2, purity = 90%, R_t_ = 7.55 min, Y = 78%, mp = 248–251 °C, Composition: C(61.78%) H(4.37%) F(5.14%) N(11.38%) O(8.66%) S(8.68%), found: (61.69%) H(4.31%) N(11.28%) S(8.61%). ^1^H NMR (400 MHz, DMSO) δ 10.41 (bp, 1H, NH), 8.79 (d, *J* = 8.5 Hz, 1H, ArH), 8.28 (dd, *J* = 7.3, 1.0 Hz, 1H, ArH), 8.20–8.12 (m, *J* = 8.3 Hz, 2H, ArH, NH), 8.05 (d, *J* = 7.7 Hz, 1H, ArH), 7.68 (ddd, *J* = 8.5, 6.9, 1.4 Hz, 1H, ArH), 7.65–7.59 (m, 2H, ArH), 7.20 (td, *J* = 8.1, 6.3 Hz, 1H, ArH), 6.83 (t, *J* = 8.8 Hz, 1H, ArH), 6.73 (d, *J* = 8.0 Hz, 1H, ArH), 4.92–4.77 (m, 1H, AlH), 1.03 (t, *J* = 6.4 Hz, 3H, AlH). ^13^C NMR (101 MHz, DMSO) δ 158.16 (d, *J* = 243.4 Hz, C-F),δ 151.37 (C_Gua_), 139.07 (ArC), 136.29 (d, J = 7.2 Hz, C-F), 134.25 (ArC), 133.28 (ArC), 130.09 (d, J = 9.5 Hz, C-F), 130.04 (ArC), 129.05 (ArC), 128.39 (ArC), 127.71 (ArC), 127.22 (ArC), 127.02 (ArC), 126.29 (ArC), 124.78 (ArC), 111.40 (d, J = 2.8 Hz, C-F), 111.15 (ArC), 109.93 (ArC), 44.18 (AlC), 23.82 (AlC). FT IR: 3275, 3237, 3145 (N-H, Str), 3014 (C-H Ar, Str), 2968, 2932 (C-H Aliph, Str), 1748 (C-H Ar, Bend), 1628, 1611 (C=N, Str), 1529, 1507, 1480, 1471, 1448 (N-H, Bend), 1363, 1342, 1325 (S=O, -SO_2_, Str), 1276, 1255, 1226, 1214 (C-N Ar, Str), 1206 (C-N, Str), 1152 (S=O, sulfonamide, Str), 1107, 1089, 1056, 1039, 1028 (C-N, Str), 980, 952, 939 (C=C Ar, Bend), 873, 804, 791, 781, 765, 737, 727, 713, 689, 669 (F-Cl, Str).

**4-chloro-*N*-(6,8-dichloro-4-methyl-3,4-dihydroquinazolin-2-yl)naphthalene-1-sulfonamide PR74**

Prepared in method **C^1^**, Formula weight for C_19_H_14_Cl_3_N_3_O_2_S: 454.8 g/mol, UPLC-MS: [M + H]^+^ = 454.2, purity = 97%, R_t_ = 9.73 min, Y = 39%, mp = 215–219 °C. ^1^H NMR (400 MHz, DMSO) δ 9.87 (bp, 1H, NH), 8.89–8.80 (m, 1H, ArH), 8.68 (bp, 1H, NH), 8.34–8.27 (m, 1H, ArH), 8.20 (d, *J* = 8.0 Hz, 1H, ArH), 7.88–7.78 (m, 3H, ArH), 7.59 (d, *J* = 2.2 Hz, 1H, ArH), 7.34 (d, *J* = 2.1 Hz, 1H, ArH), 4.66 (dt, *J* = 19.1, 9.5 Hz, 1H, ArH), 1.27 (d, *J* = 6.6 Hz, 3H, ArH). ^13^C NMR (101 MHz, DMSO) δ 151.11 (C_Gua_), 138.63 (ArC), 135.90 (ArC), 130.91 (ArC), 129.44 (ArC), 129.38 (ArC), 128.94 (ArC), 128.78 (ArC), 128.26 (ArC), 128.22 (ArC), 127.37 (ArC), 127.02 (ArC), 126.98 (ArC), 125.53 (ArC), 125.48 (ArC), 124.89 (ArC), 120.07 (ArC), 48.17 (AlC), 23.97 (AlC). FT IR: 3306, 3268 (N-H, Str), 3095 (C-H Ar, Str), 2978, 2928 (C-H Aliph, Str), 1710 (C-H Ar, Bend), 1619, 1562, 1519 (C=N, Str), 1503, 1483, 1439, 1405 (N-H, Bend), 1367, 1305 (S=O, -SO_2_, Str), 1285, 1247 (C-N Ar, Str), 1218, 1199 (C-N, Str), 1152 (S=O, sulfonamide, Str), 1111, 1095, 1061, 1033, 1009 (C-N, Str), 989, 953, 907 (C=C Ar, Bend), 841, 829, 798, 785,768, 755, 732, 686 (C-Cl, Str).

***N*** **-(6-bromo-4-methyl-3,4-dihydroquinazolin-2-yl)naphthalene-1-sulfonamide PR75**

Prepared in method **C^2^**, Formula weight for C_19_H_16_BrN_3_O_2_S: 430.3 g/mol, UPLC-MS: [M + H]^+^ = 432.1, purity = 100%, R_t_ = 7.85 min, Y = 65%, mp = 119–123 °C. ^1^H NMR (400 MHz, DMSO) δ 10.22 (s, 1H, NH), 8.74 (d, J = 8.4 Hz, 1H, ArH), 8.23 (d, J = 7.2 Hz, 1H, ArH), 8.15 (d, J = 8.2 Hz, 1H, ArH), 8.12–8.07 (m, 2H, ArH, NH), 7.61 (ddd, J = 23.1, 11.1, 4.8 Hz, 3H, ArH), 7.42 (d, J = 2.1 Hz, 1H, ArH), 7.33 (dd, J = 8.5, 2.2 Hz, 1H, ArH), 6.81 (d, J = 8.5 Hz, 1H, ArH), 4.71–4.67 (m, 1H, AlH), 1.04 (d, J = 6.5 Hz, 3H^13^C NMR (101 MHz, DMSO) δ 151.39 (C_Gua_), 139.15 (ArC), 134.24 (ArC), 133.74 (ArC), 133.22 (ArC), 131.26 (ArC), 129.03 (ArC), 128.80 (ArC), 128.39 (ArC), 127.69 (ArC), 127.19 (ArC), 126.99 (ArC), 126.31 (ArC), 126.02 (ArC), 124.79 (ArC), 117.33 (ArC), 115.16 (ArC), 48.39 (AlC), 24.87 (AlC). FT IR: 3291, 3133 (N-H, Str), 2970, 2924 (C-H Aliph, Str), 1738 (C-H Ar, Bend), 1630, 1600 (C=N, Str), 1518, 1487, 1441, 1411 (N-H, Bend), 1376 (-CH_3_, Bend), 1343 (S=O, -SO_2_, Str), 1288, 1252, 1217 (C-N Ar, Str), 1205 (C-N, Str), 1150 (S=O, sulfonamide, Str), 1100, 1050, 1025 (C-N, Str), 992, 946, 913 (C=C Ar, Bend), 891, 872, 852, 826, 794, 763 (Br-Cl, Str).

***N*** **-(3,4-dimethyl-3,4-dihydroquinazolin-2-yl)naphthalene-1-sulfonamide PR76**

Prepared in method **B^1^**, Formula weight for C_20_H_19_N_3_O_2_S: 365.5 g/mol, UPLC-MS: [M + H]^+^ = 366.2, purity = 98%, R_t_ = 8.07 min, Y = 52%, mp = 215–217 °C, Composition: C(65.73%) H(5.24%) N(11.50%) O(8.76%) S(8.77%), found: C(65.69%) H(5.22%) N(11.59%) S(8.73%). ^1^H NMR (400 MHz, CDCl3) δ 9.55 (s, 1H, NH), δ 8.08–8.04 (m, 1H, ArH), 8.04–7.98 (m, 2H, ArH), 7.91 (dd, J = 7.9, 1.8 Hz, 1H, ArH), 7.73 (dtd, J = 9.3, 7.8, 1.9 Hz, 2H, ArH), 7.65 (t, J = 8.1 Hz, 1H, ArH), 7.53 (ddd, J = 14.7, 7.5, 1.2 Hz, 2H, ArH), 7.35–7.12 (m, 2H, ArH), 4.92 (q, J = 6.8 Hz, 1H, AlH), 3.29 (s, 3H, AlH), 1.53 (d, J = 6.8 Hz, 3H, AlH). ^13^C NMR (100 MHz, CDCl3) δ 150.59 (ArH), 139.22 (ArH), 136.26 (ArH), 133.74 (ArH), 129.51 (ArH), 129.40 (ArH), 128.81 (ArH), 128.40 (ArH), 128.00 (ArH), 127.70 (ArH), 126.75 (ArH), 124.10 (ArH), 116.00 (ArH), 57.80 (AlH), 36.97 (AlH), 18.10 (AlH). FT IR: 3309 (N-H, Str), 3076 (C-H Ar, Str), 2978, 2917, 2850 (C-H Aliph, Str), 1737 (C-H Ar, Bend), 1621 (C=N, Str), 1590, 1529 (N-H, Bend), 1496, 1462, 1453, 1408 (-CH_2_, Bend), 1375 (-CH_3_, Bend), 1345, 1311 (S=O, -SO_2_, Str), 1289, 1270, 1260, 1240, 1210 (C-N Ar, Str), 1193, 1165, 1152, 1118, 1100 (S=O, sulfonamide, Str), 1072, 1046, 1030 (C-N, Str), 992, 964, 933, 889 (C=C Ar, Bend)

***N*** **-(4-methyl-4H-3,1-benzoxazin-2-yl)naphthalene-1-sulfonamide PR77**

Prepared in method **B^1^**, Formula weight for C_19_H_16_N_2_O_3_S: 352.4 g/mol, UPLC-MS: [M + H]^+^ = 352.2, purity = 93%, R_t_ = 7.26 min, Y = 54%, mp = 229–233 °C, Composition: C(64.57%) H(4.85%) N(7.93%) O(13.58%) S(9.07%), found: C(64.47%) H(4.73%) N(7.99%) S(8.98%). ^1^H NMR (400 MHz, DMSO) δ 9.65 (bp, 1H, NH), 8.83 (d, J = 8.6 Hz, 1H, ArH), 8.25–8.21 (m, 2H, ArH), 8.14 (d, J = 8.3 Hz, 1H, ArH), 8.01 (d, J = 7.6 Hz, 1H, ArH), 7.73–7.59 (m, 5H, ArH), 7.14 (s, 1H, ArH), 7.11 (d, J = 7.4 Hz, 1H, ArH), 6.97–6.92 (m, 1H, ArH), 3.94 (q, J = 7.1 Hz, 1H, AlH), 3.35 (s, 3H, AlH). ^13^C NMR (101 MHz, DMSO) δ 155.09 (ArC), 139.57 (ArC), 137.67 (ArC), 134.29 (ArC), 133.00 (ArC), 130.71 (ArC), 129.82 (ArC), 128.96 (ArC), 128.48 (ArC), 127.65 (ArC), 127.61 (ArC), 126.98 (ArC), 126.72 (ArC), 126.48 (ArC), 124.87 (ArC), 123.51 (ArC), 120.77 (AlC), 43.76 (AlC), 34.98 (AlC). FT IR: 3313, 3292, 3202, 3138, 3103 (N-H, Str), 3004 (C-H Ar, Str), 2976 (C-H Aliph, Str), 1639, 1605 (C=N, Str), 1592, 1546, 1503 (N-H, Bend), 1443 (-CH_3_, Bend), 1388, 1348, 1339, 1321 (S=O, -SO_2_, Str), 1291, 1265, 1248, 1223 (C-N Ar, Str), 1199 (C-N, Str), 1148 (S=O, sulfonamide, Str), 1100 (C-O, Str), 1062, 1029, 1016 (C-N, Str), 982, 959, 942, 910 (C=C Ar, Bend).

***N*** **-(4,5-dihydro-3H-1,3-benzodiazepin-2-yl)naphthalene-1-sulfonamide PR78**

Prepared in method **B^1^**, Formula weight for C_19_H_17_N_3_O_2_S: 351.4 g/mol, UPLC-MS: [M + H]^+^ = 352.2, purity = 94%, R_t_ = 7.01 min, Y = 34%, mp = 178–180 °C. Composition: C(64.94%) H(4.88%) N(11.96%) O(9.11%) S(9.12%), C(64.94%) H(4.88%) N(11.96%) O(9.11%) S(9.12%), found: C(64.81%) H(4.89%) N(11.92%) S(9.10%). ^1^H NMR (400 MHz, DMSO) δ 9.67 (bp, *J* = 36.9 Hz, 1H, NH), 8.82 (d, *J* = 8.5 Hz, 1H, ArH), 8.21 (bp, *J* = 7.3 Hz, 2H, NH, ArH), 8.14 (d, *J* = 8.2 Hz, 1H, ArH), 8.04 (d, *J* = 7.9 Hz, 1H, ArH), 7.74–7.57 (m, 4H, ArH), 7.14 (d, *J* = 2.4 Hz, 1H, ArH), 7.10 (d, *J* = 7.6 Hz, 1H, ArH), 6.99–6.90 (m, 1H, ArH), 3.48–3.40 (m, 2H, AlH), 2.95–2.87 (m, 2H, AlH).^13^C NMR (101 MHz, DMSO) δ 155.09 (C_Gua_), 139.56 (ArC), 137.67 (ArC), 134.29 (ArC), 133.00 (ArC), 130.71 (ArC), 129.82 (ArC), 128.97 (ArC), 128.48 (ArC), 127.65 (ArC), 127.61 (ArC), 126.98 (ArC), 126.72 (ArC), 126.48 (ArC), 124.88 (ArC), 123.51 (ArC), 120.77 (ArC), 43.75 (AlC), 34.98 (AlC). FT IR: 3313, 3293, 3204, 3138 (N-H, Str), 3005 (C-H Ar, Str), 2970 (C-H Aliph, Str), 1738 (C-H Ar, Bend), 1639, 1593, 1546 (C=N, Str), 1504, 1443 (N-H, Bend), 1350, 1340, 1323 (S=O, -SO_2_, Str), 1291, 1264, 1248 (C-N Ar, Str), 1217, 1200 (C-N, Str), 1151 (S=O, sulfonamide, Str), 1101, 1065, 1029, 1016 (C-N, Str), 982, 959, 909 (C=C Ar, Bend).

### 3.2. Radioligand Assay

#### 3.2.1. Cell Culture and Preparation of Cell Membranes for Radioligand Binding Assays

The HEK293 with a stable expression of human 5-HT_6,_ 5-HT_1A_, 5-HT_5A_, 5-HT_7b_, and D_2L_ receptors were prepared with the use of Lipofectamine 2000. The cells were maintained at 37 °C (humidified atmosphere, with 5% CO_2_) and grown in Dulbecco’s Modifier Eagle Medium (10% dialyzed fetal bovine serum, 500 µg/cm^3^ G418 sulfate). For membrane preparation, the cells were subcultured in 150 cm^2^ flasks, grown to 90% confluence, and then washed twice with phosphate-buffered saline (PBS) prewarmed to 37 °C and pelleted under centrifugation (using × 200× *g*) in PBS containing 0.1 mM EDTA and 1 mM dithiothreitol. The pellets were stored at −80 °C.

#### 3.2.2. Radioligand Binding Assays

Cell pellets were thawed and homogenized in 10 volumes of an assay buffer using an Ultra Turrax tissue homogenizer and centrifuged twice at 35,000× *g* for 15 min at 4 °C. The composition of the assay buffers is as follows:

5-HT_6_—50 mM Tris–HCl, 0.5 mM EDTA, and 4 mM MgCl_2_; 5-HT_5A_—50 mM Tris HCl, 0.5 mM EDTA, and 4 mM MgCl_2_; 5-HT_1A_—50 mM Tris–HCl, 0.1 mM EDTA, 4 mM MgCl_2_, 10 μM pargyline, and 0.1% ascorbate; 5-HT_7_—50 mM Tris–HCl, 4 mM MgCl_2_, 10 μM pargyline, and 0.1% ascorbate; D_2_—50 mM Tris HCl, 1 mM EDTA, 4 mM MgCl_2_, 120 mM NaCl, 5 mM KCl, 1.5 mM CaCl_2_, and 0.1% ascorbate. 

The assays were incubated in a total volume of 200 μL on 96-well microtiter plates for 1 h (37 °C, except for 5-HT_1A_, which was incubated at room temperature). The process of equilibration was terminated with rapid filtration through Unifilter plates (96-well cell harvester, PerkinElmer Waltham, MA, USA), and the radioactivity retained on the filters was quantified on a Microbeta plate reader (PerkinElmer). For displacement studies, the assay samples contained the following as radioligands: 2 nM [^3^H]-LSD (85k Ci/mmol)—5-HT_6_; 2.5 nM [^3^H]-8-OHDPAT (187 Ci/mmol)—5-HT_1A_; 3.5 nM [^3^H]-LSD–5—HT_5A_; 0.8 nM [^3^H]-5-CT (39.2 Ci/mmol)—5-HT_7_; 2.5 nM [^3^H]-raclopride (76.0 Ci/mmol)—D_2_. Nonspecific binding was defined with 10 μM of methiothepine for 5-HT_6_ assays, 10 µM SB-699551 for 5-HT_5A_, chlorpromazine for D_2_, and 5-HT for (5-HT_1A_, 5-HT_7_). Each compound was tested in triplicate at 8 different concentrations (10^−4^ to 10^−11^ M). The inhibition constants (*K*i) were calculated from the Cheng–Prusoff equation [60]. The results are expressed as the means of at least two separate experiments. The reference compounds are as follows: olanzapine for 5-HT_6_, buspirone for 5-HT_1A_, clozapine for 5-HT_7_, and risperidone for D_2_.

#### 3.2.3. Functional Evaluation

*HEK293* cell lines with a *stable* expression of *human* D_2_, 5-HT_1A_, 5-HT_6_, and 5-HT_7_ were maintained at 37 °C in a humidified atmosphere with 5% CO_2_ and were grown in Dulbeco’s Modifier Eagle Medium containing 10% dialyzed fetal bovine serum and 500 µg/mL G418 sulfate. For functional experiments, cells were subcultured in 25 cm^2^ flasks, grown to 90% confluence, washed twice with phosphate-buffered saline (PBS) prewarmed to 37 °C, and centrifuged for 5 min (160× *g*). The supernatant was aspirated, and the cell pellet was resuspended in a stimulation buffer (1 × HBSS, 5 mM HEPES, 0.5 mM IBMX, 0.1% BSA). 

The functional properties of compounds were evaluated using the LANCE Ultra cAMP detection kit (PerkinElmer). Additionally, cells with receptors coupled to the G_i_ subtype (D_2_ and 5-HT_1A_) were stimulated with 1 μM of forskolin (EC_90_). To investigate the antagonist activity, dopamine for D_2_R was added at a concentration of 10 nM, and for cell lines with 5-HT_7_R, 5-HT_1A_R, or 5-HT_6_R, 5-CT concentrations were adjusted, being, respectively, 10, 100, and 1000 nM, according to the receptor subtype.

For the quantification of cAMP levels, cells (5 µL) were incubated with a 5 µL mixture of necessary compounds for 30 min at room temperature in a 384-well white opaque microtiter plate (PerkinElmer). After incubation, the reaction was stopped, and cells were lysed by the addition of a 10 µL working solution (5 µL Eu-cAMP and 5 µL ULight-anti-cAMP). The assay plate was incubated for 1 h at room temperature. The time-resolved fluorescence resonance energy transfer (TR-FRET) signal was detected by an Infinite M1000 Pro (Tecan, Männedorf, Switzerland) using instrument settings from the LANCE Ultra cAMP detection kit manual. Each compound was tested in triplicate at 8 concentrations. K_b_ for each inhibitor was determined using the Cheng–Prusoff equation [56], specific for the analysis of functional inhibition curves: K_b_ = IC_50_/(1 + A/EC_50_), where A denotes the concentration of the used agonist, IC_50_ the concentration of the antagonist that achieves a 50% reduction in the agonist effect, and EC_50_ the agonist concentration, which causes half of the maximal response.

### 3.3. Cytotoxicity Assessment

The human glioblastoma cell line LN-229 (ATCC CRL-2611) and human breast carcinoma cell line MCF7 (ATCC HTB-22) were purchased from ATCC. The human astrocytoma 1321N1 (ECACC 86030402) cell line was purchased from ECACC. The glioblastoma U-251MG and U87MG cells were kindly provided by prof. G. Kramer-Marek from the Institute of Cancer Research in London (United Kingdom) and by prof. Irving W. Wainer from the National Institute on Aging in Baltimore (MD, USA), respectively. The human pancreas adenocarcinoma cell line AsPC-1 was bought from Sigma-Aldrich (St. Louis, MO, USA). The normal human astrocytes (NHAs) were purchased from Gibco (Waltham, MA, USA). The 1321N1, LN-229, MCF7, and U-251 cell lines were cultured in Dulbecco’s Modified Eagle’s Medium (DMEM) that had been supplemented with 10% heat-inactivated fetal bovine serum—FBS (all from Sigma-Aldrich). The U87MG cell line was maintained in MEM with 10% heat-inactivated FBS. The pancreatic AsPC-1 cells were cultured in an RPMI-1640 medium (Merck), which contained 10% heat-inactivated FBS. Each complete medium contained a mix of two antibiotics—penicillin and streptomycin (1% *v*/*v*; Gibco). The NHA cells were cultured in an astrocyte medium with 10% FBS, an N-2 supplement, EGF (20 ng/mL, Gibco), and an antibiotic–antimitotic (1% *v*/*v*; Gibco). Normal brain cat (Feline catus) astrocyte cell line PG-4 (S + L) (ATCC-CRL-2032™) was cultured using McCoy’s 5A medium supplemented with 10% heat-inactivated FBS (*v*/*v*), 100 U/mL penicillin, and 100 μg streptomycin (complete medium). Cultures were subcultivated every 3–4 days by trypsinization. Cell lines were cultured at 37 °C with a 5% CO_2_ humidified atmosphere. All of the cell lines were tested for *Mycoplasma* contamination. 

The cells were seeded in 96-well plates (Nunc) at a density of 5000 cells per well and incubated under standard conditions at 37 °C for 24 h. The assay was carried out following a 72 h incubation with the various concentrations of the tested compounds (6–25 μM). Then, a medium without phenol red with a CellTiter 96^®^AQueous One Solution-MTS (Promega) solution was added to each well and incubated for 1 h at 37 °C. The optical densities of the samples were measured at 490 nm using a multi-plate reader. The obtained results were compared to the control and were estimated as the inhibitory concentration (IC_50_) values (using GraphPad Prism 8.0). Each individual compound was tested in triplicate in a single experiment with each experiment being performed three or four times.

### 3.4. Biochromatographic Experiments 

For HPLC analyses, the Prominence-1 LC-2030C 3D HPLC system (Shimadzu, Tokyo, Japan) equipped with a DAD detector and controlled by the LabSolution system (version 5.90, Shimadzu, Japan) was used. During this study, three chromatographic columns varied in terms of chemical modifications of stationary phases that were applied with the following mobile phases and elution modes:

- IAM.PC.DD2 (100 mm × 4.6 mm × 10.0 µm with a guard column; Regis Technologies; Morton Grove, IL, USA), mobile phase A: a 50 mM ammonium acetate water solution adjusted to pH 7.4, mobile phase B: acetonitrile, linear gradient from 0 to 85% B in 5.25 min was applied and then held at 85% ACN for 0.5 min. The mobile phase flow rate was 1.5 mL/min, and the column was maintained at 30 °C.
- C18 Hypersil GOLDTM (50 mm × 4.6 mm; 5.0 µm with a guard column; Thermo Scientific, Waltham, MA, USA); mobile phase A: a 50 mM ammonium acetate water solution adjusted to pH 7.4 and 10.5 and acetic acid adjusted to 2.6; mobile phase B: acetonitrile, linear gradient from 0 to 98% B in 6.5 min was applied and then held at 98% ACN for 0.5 min. The mobile phase flow rate was 1.5 mL/min, and the column was maintained at 30 °C.
- Chiralpak^®^ HSA (50 mm × 4 mm; 5 µm with safety guard column; Daicel Chiral Technologies, West Chester, PA, USA), mobile phase A: 50 mM ammonium acetate, mobile phase B: HPLC-grade isopropanol (VWR International, Leuven, Belgium). For the first 15 min, the linear gradient from 0 to 20% isopropanol was applied and then held at 20% isopropanol for 12 min. In the last 5 min of the sequence, the mobile phase mixture received a pure ammonium acetate solution. The column temperature was held at 30 °C, whereas the flow rate was 0.9 mL/min.

The reference substances for the calibration of HSA, C_18_, and IAM columns were purchased, respectively: acetanilide, butyrophenone, diclofenac, and octanonophenone (Alfa Aesar, Haverhill, MA, USA); acetophenone, benzimidazole, colchicine, indole, indometacin, paracetamol, and theophylline (Sigma-Aldrich, Steinheim, Germany); nicardipine and nizatidine (Cayman Chemical, Ann Arbor, MI, USA); carbamazepine, heptanophenone, hexanophenone, propiophenone, and valerophenone (Acros Organic, Pittsburg, PA, USA). Before chromatographic experiments, solutes were dissolved in dimethyl sulfoxide (Avantor Performance Materials Poland S.A., Gliwice, Poland) to obtain a 200 µg/mL concentration. The detection was performed in the UV region at the wavelength from 190 nm to 300 nm. The injected volume was 5 μL, and each compound was analyzed in triplicate. Retention times for studied molecules and reference standards are collected in Supplementary Materials, Table S1.

### 3.5. ADMET Tests

The ADMET parameters were analyzed according to the previously described protocols [61,62] and included Parallel Artificial Membrane Permeability Assay (PAMPA) passive permeability testing, the influence on CYP3A4 and CYP2D6 activity, plasma protein binding, metabolic stability in mouse liver microsomes, and hepatotoxicity assessment with HepG2 cells.

#### 3.5.1. PAMPA Test

Precoated PAMPA Plate System Gentest was obtained from Corning (Tewksbury, MA, USA). Test compounds and caffeine solutions (200 µM) were prepared in a PBS buffer (pH = 7.4) and then added to the PAMPA plate. The plate was incubated at room temperature for 5 h without agitation. Then, 150 µL was aspirated from both acceptor (A) and donor (D) wells and then diluted with a 150 µL internal standard (IS) solution. The concentrations of the tested compounds in wells A and D were estimated using an LC/MS Waters ACQUITY™ TQD system with a TQ detector (Waters, Milford, CT, USA). The values of Pe were estimated according to the exact formulas provided by Corning and previously described in the literature [63].

#### 3.5.2. Drug–Drug Interactions

The influence on CYP3A4 and CYP2D6 activity by ligands was analyzed with luminescent CYP3A4 P450-Glo and CYP2D6 P450-Glo^TM^ (Madison, WI, USA). The compounds were tested in triplicate. The bioluminescence signal was measured with a microplate reader, Spark Cyto (Tecan, Männedorf, Switzerland), in the luminescence mode. Compounds were tested in triplicate in the range of 0.01–25 µM for both isoforms’ P450 cytochrome. KE and QD were used as the reference compounds for CYP3A4 and CYP2D6, respectively.

#### 3.5.3. Metabolic Stability

The metabolic stability of **PR 68** was estimated with mouse liver microsomes (MLMs) (Sigma-Aldrich, St. Louis, MO, USA). The test compound (50 µM) was incubated in the presence of MLMs (1 mg/mL) for 120 min in a buffer (10 mM Tris-HCl buffer at 37 °C). Cold methanol was then added to the reaction mixture to quench the reaction. The precipitated MLMs were centrifuged, and the supernatant was analyzed by LC/MS with a Waters ACQUITY™ TQD system with a TQ detector (Waters, Milford, USA).

#### 3.5.4. Hepatotoxicity

Hepatotoxicity was evaluated employing an MTS assay with a HepG2 human hepatoma cell line (ATCC: HB-8065) [64,65]. In brief, the cells were incubated for 48 h in 96-well plates with **PR 68** (concentration range: 1–100 μM), or doxorubicin (DX, 1 µM; Sigma-Aldrich), which served as a control. Compounds were tested in a single experiment in quadruplicate. The absorbance in the MTS assay was measured by the Spark Cyto microplate reader (Tecan, Männedorf, Switzerland).

#### 3.5.5. Plasma Protein Binding

This study was performed with the use of the commercial TRANSIL^XL^ PPB Assay (Sovicell, Leipzig, Germany). The test was performed according to recommendations from the protocol provided by the manufacturer. Before the assay, the plate was thawed at room temperature for 3 h. The compounds (stock solution in DMSO) were solved in a PBS buffer up to 320 µM. To each well of the eight-well tube units (six wells containing different concentrations of human serum albumin (HSA) and α_1_-acid glycoprotein (AGP) mixed in a physiological ratio of 24:1 and two wells of references), 15 µL of the tested compound solution was added to obtain a 20 µM final concentration of the reference warfarin (WFN) and **PR 68**. The plate was incubated on a plate shaker at 1000 rpm for 12 min. Next, the plate was centrifugated at 750 g for 10 min. The supernatants were collected and analyzed by LC/MS. The PPB parameters of compound **PR 68** and the highly bound reference WFN were calculated using the following equation:

$$K_{D}=\frac{\left[A\right][P]}{\left[AP\right]}$$

where

- [*A*]—free concentration of drug;
- [*P*]—free concentration of protein;
- [*AP*]—concentration of drug *A* bound to protein *P*.

The total fraction bound was estimated by

$$f_{b}=1-\frac{1}{1+\frac{\left[HSA\right]}{K_{D}^{HSA}}+\frac{\left[AGP\right]}{K_{D}^{AGP}}}$$

#### 3.5.6. Statistical Analysis

The statistical significance determination was evaluated with GraphPad Prism 5.0.1 software using one-way ANOVA, followed by Bonferroni’s comparison test: *p* < 0.001 for DDI and hepatotoxicity, and *p* < 0.05 for other tests.

#### 3.5.7. In Vivo Cardiotoxicity and Neurotoxicity

To determine the toxicity of the compounds [66], the fish embryo toxicity (FET) test was performed on zebrafish (*Danio rerio*) according to OECD Test 236. The collected embryos were transferred to a Petri dish with an E3 medium, consisting of 5 mM NaCl; 0.33 mM MgCl_2_; 0.33 mM CaCl_2_; and 0.17 mM KCl, and placed in 6-well plates, 10 embryos per well. Stock solutions were prepared in DMSO. The range of different concentrations of the solutions was prepared by dissolving stock solutions in the E3 medium each time directly before addition to the wells. The solutions were changed once daily, and the embryos were maintained in the incubator at 28.5 °C. At the end of the exposure period (96 hpf—hours postfertilization), acute toxicity was determined based on a positive outcome in any of the four visual indicators of lethality, including the coagulation of fertilized eggs, lack of somite formation, lack of detachment of the tailbud from the yolk sac, and lack of heartbeat. The value of LD_50_ was calculated. Heartbeats were recorded to observe cardiotoxic effects. A dose–response curve was generated using Prism 8.0.1 (GraphPad Software). The concentrations of the compounds of interest causing 50% mortality (LD_50_) of 96 hpf larvae of *Danio rerio* were calculated.

For neurotoxicity determination, a locomotor activity test was used. First, the Maximum Tolerated Concentration (MTC) test was performed to pick proper doses for behavioral tests. Briefly, 4-dpf zebrafish larvae were immersed for 24 h in solutions of PP10 and PP15 compounds of concentrations ranging from 0 to 10 µg/mL to evaluate toxic doses. Then, applying appropriate doses (1 and 2.5 µg/mL), 4-dpf larvae were immersed for 24 h and then their locomotor activity was measured in a 10 min program consisting of 6 min in light and 4 min in dark conditions. The distances moved by the larvae were analyzed using EthoVision XT15 software (Version number: 15.0.1418; Noldus, Wageningen, The Netherlands). Mean distance per 1 min was calculated for each 10 min. Then, activity in the light and in the dark was calculated and compared.

### 3.6. Molecular Modeling

#### 3.6.1. Protein–Ligand Docking

The estimation of the pKa of nitrogen atoms was performed in the Jaguar pKa prediction system [67]. Calculations include the geometry optimization of ionic and neutral species, single-point energy and frequency calculations, and empirical fitting. The software PyMOL 3.0 [68] was used to graphically represent the selected structures. Three-dimensional constructs were made using LigPrep 3.7 [69]. The appropriate ionization states at pH ¼ 7.4 were determined using Epik [70]. The Protein Preparation Wizard [71] was used to determine the bond order and ionization states of the appropriate amino acids. Docking was performed using the Schrödinger induced fit (IFD) protocol. This method combines flexible ligand docking using the Glide algorithm with receptor structure prediction and side chain refinement in Prime (refines residues within 5.0 Å of ligand positions). An extended sampling protocol was used, generating up to 80 positions per ligand using automatic docking. In each case, the grid center was set at D3.32 and allowed residues to be refined within 15 Å of the ligand positions. The final validation of the selected receptor forms was performed by docking the synthesized library (Glide SP mode), retaining only those with a coherent binding mode [72].

#### 3.6.2. FMO

To investigate the importance and nature of the L-R interactions, single-point FMO-EDA [54] calculations were performed at the MP2/6-31G** [73] level of the fully optimized complexes using the GAMESS software [74]. FMO calculations were performed for the ligand and receptor binding site. This strategy has recently been applied to other GPCRs [75]. FMO input commands have been set to default values. Pair interaction energies (PIEs) and all contributions to the total energy (electrostatic—E_es_, dispersion—E_dis_, charge transfer—E_ct_, exchange repulsion—E_ex_, and the Gibbs solvation energy—ΔG_solv_) were calculated as previously described [54]. The Gibbs solvation energy was calculated based on the PCM model. FACIO 25.1.1.64 [76] was used for the preparation of the systems and analysis of the results. 

#### 3.6.3. Molecular Dynamics

The long-time-scale molecular dynamics simulations were performed, employing an all-atom approach using program NAMD 2.13; standard all-atom forcefield CHARMM [77] is usually employed, which is available in all the above. The membrane and simulation system was built with the QwikMD beta tool in VMD 1.9.3 [78], using POPC (1-palmitoyl-2-oleoylphosphatidylcholine), solvent explicit, and buffer 15 A salt cons at 0.15 mol/L NaCl. 

#### 3.6.4. Three-Dimensional QSAR

A field-based 3D-QSAR analysis was performed using the QSAR tool from the Maestro Schrodinger Suite [79]. The 3D-QSAR method builds a model by associating known activities and molecular elements of the training set using a Gaussian field (GFQSAR). Interaction energy calculations were performed using steric, electrostatic, hydrogen bond donor (HBD), and hydrogen bond acceptor (HBA) potential fields, using Gaussian equations for field calculations. The fields were calculated on an orthohedral grid spanning the training set molecules, with a spacing of 1 Å and extending 3 Å beyond the boundaries of the set. The threshold for van der Waals and electrostatic interactions was set at 30 kcal/mol, eliminating points closer than 2 Å from any atom in the training set. In the PLS procedure, all variables (grid points) with a standard deviation less than 0.01 were removed. A partial least squares (PLS) analysis was used to construct the best model through GFQSAR linear correlation [80]. The maximum number of PLS factors was set to 7. The leave-one-out cross-analysis was performed. The Phase program [81] was used to generate 5-HT_6_ receptor pharmacophores and a QSAR model. In 3D QSAR atomic modeling, the ligand space was divided into a fine cubic grid, in which the atoms of the ligand molecules are fitted to the corners of the cubic grid, and the occupancy of the ligand atoms is encoded as numerical information. A total of 7 PLS coefficients were used when generating the atomic-based 3D QSAR model. The generated model was validated internally on the training set and then externally on the testing set to check its stability and predictive power using the regression coefficient (R^2^), cross-validation correlation coefficient (Q^2^), and other PLS components. Ligands were randomly distributed to the training set and test set in a 50/50 ratio and shuffled arbitrarily to obtain a good 3D QSAR model with high predictive power. To create a pharmacophore phase model, the prepared ligands were imported along with their corresponding p*K*_i_ biological activity values. The level of critical fit of the hypothesis was set at 50%, with 4–5 features.

## 4. Conclusions 

As part of the conducted research, a new core of sulfonamide derivatives of cyclic arylguanidines was developed, matching the chemotype of low-basicity 5-HT_6_R ligands. Several antagonists with an affinity constant *K*_i_ < 50 nM (**PR 68** *K*_i_ = 37 nM) were selected. These compounds were characterized by very high selectivity. The SAR analysis supported by molecular modeling methods allowed us to conclude that the most favorable moieties in terms of high affinity are a 1-naphthalene group or its halogen-substituted analog in the arylsulfone part. In the arylguanidine group, substituting the quinazoline ring in the *8-* or *5-* position turns out to be the most preferred.

Importantly, in this publication, we refuted the initial hypothesis about the potential use of selective 5-HT_6_R antagonists in the treatment of high-grade gliomas, as no correlation between high affinity and antiproliferative activity was observed. However, the selective nature of the developed compounds makes it possible to use them in the treatment of other CNS diseases, mainly those related to cognitive deficits.

The analysis of pharmacokinetic parameters for the lead compound **PR 68** confirmed favorable properties related to administration, predicting the ability of passive diffusion and acceptable metabolic stability (metabolized in 49%, MLMs). The compound did not exhibit the potential for drug–drug interactions. Distribution remains the biggest problem in ADME parameters because **PR 68** binds strongly to PPB. However, ADME tests should be performed in in vivo conditions, to definitively assess the usefulness of the compound in pharmacotherapy.

## Figures and Tables

**Table 1:** Binding data of synthesized compounds from 1st group for 5-HT_6_R, with SD. Data are presented as a heat map where the highest affinities are marked in green, moderate in yellow, and the lowest in red.

	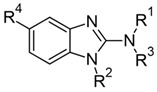				5-HT_6_
Name	R^1^	R^2^	R^3^	R^4^	*K*_i_ [µM]
**PR 1**	H	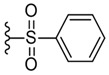	H	H	2.157 ± 0.539
**PR 2**	H	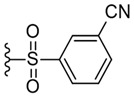	H	H	49.220 ± 1.2305
**PR 3**	H	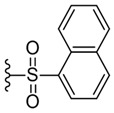	H	H	0.148 ± 0.037
**PR 4**	H	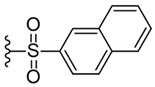	H	H	0.330 ± 0.083
**PR 5**	H	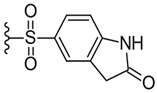	H	H	14.760 ± 3.690
**PR 6**	H	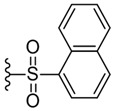	H	Cl	0.704 ± 0.176
**PR 7**	H	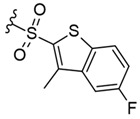	H	H	2.946 ± 0.737
**PR 8**	H	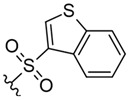	H	H	0.133 ± 0.033
**PR 9**	H	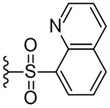	H	H	0.318 ± 0.018
**PR 10**	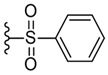	H	H	H	1.716 ± 0.429
**PR 11**	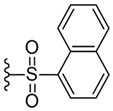	H	H	H	0.325 ± 0.081
**PR 12**	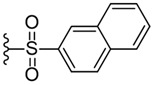	CH_3_	CH_3_	H	41.930 ± 10.483

**Table 2:** Binding data of synthesized compounds from 2nd group for 5-HT_6_R, with SD. Data are presented as a heat map where the highest affinities are marked in green, moderate in yellow, and the lowest in red.

	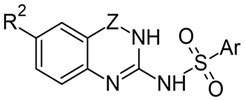			5-HT_6_
Name	Z	R^2^	Ar	*K*_i_ [µM]
**PR 13**	CH_2_	H	Ph	2.606 ± 0.651
**PR 14**	CH_2_	H	4-F-Ph	2.441 ± 0.610
**PR 15**	CH_2_	H	3,4-F-Ph	5.273 ± 1.328
**PR 16**	CH_2_	H	4-CF_3_-Ph	5.097 ± 1.274
**PR 17**	CH_2_	H	4-OCF_3_-Ph	9.851 ± 2.463
**PR 18**	CH_2_	H	2-Cl-Ph	1.803 ± 0.451
**PR 19**	CH_2_	H	3-Cl-Ph	0.521 ± 0.130
**PR 20**	CH_2_	H	2,3-Cl-Ph	0.293 ± 0.073
**PR 21**	CH_2_	H	3,4-Cl-Ph	1.025 ± 0.256
**PR 22**	CH_2_	H	2,6-Cl-Ph	1.457 ± 0.364
**PR 23**	CH_2_	H	3,5-Cl-Ph	0.609 ± 0.152
**PR 24**	CH_2_	H	3-OCH_3_-Ph	0.906 ± 0.227
**PR 25**	CH_2_	H	2-OCH_3_,5-Cl-Ph	4.638 ± 1.160
**PR 26**	CH_2_	H	4-Ph-Ph	4.310 ± 1.078
**PR 27**	CH_2_	Cl	Ph	1.688 ± 0.422
**PR 28**	CH(CH_3_)	H	Ph	4.025 ± 1.006

**Table 3:** Binding data of synthesized compounds from 3rd group for 5-HT_6_ receptors, with SD. Data are presented as a heat map where the highest affinities are marked in green, moderate in yellow, and the lowest in red.

	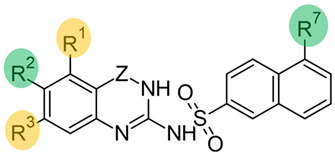					5-HT_6_
**Name**	**Z**	**R^1^**	**R^2^**	**R^3^**	**R^7^**	***K*_i_ [µM]**
**PR 29**	CH_2_	H	H	H	H	3.634 ± 0.909
**PR 30**	CH_2_	H	H	H	Cl	0.972 ± 0.242
**PR 31**	CH_2_	Cl	H	H	H	2.167 ± 0.538
**PR 32**	CH_2_	H	Cl	H	H	0.948 ± 0.212
**PR 33**	CH_2_	H	H	Cl	H	1.961 ± 0.434
**PR 34**	CH(CH_3_)	H	H	H	H	0.702 ± 0.152
**PR 35**	CH(CH_3_)	H	Cl	H	H	2.383 ± 0.593
**PR 36**	CH(CH_3_)	H	Br	H	H	29.440 ± 7.356
**PR 37**	CH(CH_3_)	H	H	Cl	H	2.216 ± 0.534
**PR 38**	CH_2_CH_2_	H	H	H	H	1.407 ± 0.331

**Table 4:** Binding data of synthesized compounds from the 4th group for 5-HT_6_R, with SD. Data are presented as a heat map where the highest affinities are marked in green, moderate in yellow, and the lowest in red.

	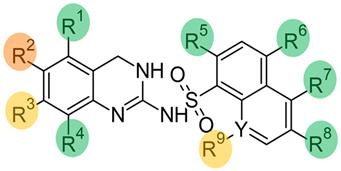										5-HT_6_
**Name**	**R^1^**	**R^2^**	**R^3^**	**R^4^**	**R^5^**	**R^6^**	**R^7^**	**R^8^**	**R^9^**	**Y**	***K*_i_ [µM]**
**PR 39**	H	H	H	H	H	H	H	H	-	N	4.271 ± 0.983
**PR 40**	H	H	H	H	H	H	H	H	H	C	0.651 ± 0.135
**PR 41**	H	H	H	H	Cl	H	H	H	H	C	0.210 ± 0.048
**PR 42**	H	H	H	H	H	Cl	H	H	H	C	0.268 ± 0.063
**PR 43**	H	H	H	H	H	H	Cl	H	H	C	0.258 ± 0.046
**PR 44**	H	H	H	H	H	H	H	Cl	H	C	0.282 ± 0.065
**PR 45**	H	H	H	H	H	H	H	H	Cl	C	0.736 ± 0.169
**PR 46**	Cl	H	H	H	H	H	H	H	H	C	0.160 ± 0.036
**PR 47**	H	Cl	H	H	H	H	H	H	H	C	0.341 ± 0.076
**PR 48**	H	H	Cl	H	H	H	H	H	H	C	0.668 ± 0.156
**PR 49**	H	H	H	Cl	H	H	H	H	H	C	0.077 ± 0.018
**PR 50**	Cl	Cl	H	H	H	H	H	H	H	C	0.451 ± 0.102
**PR 51**	H	Cl	H	Cl	H	H	H	H	H	C	0.866 ± 0.199
**PR 52**	Cl	H	H	H	Cl	H	H	H	H	C	0.133 ± 0.033
**PR 53**	Cl	H	H	H	H	Cl	H	H	H	C	0.189 ± 0.042
**PR 54**	F	H	H	H	H	H	H	H	H	C	0.381 ± 0.088
**PR 55**	H	F	H	H	H	H	H	H	H	C	1.792 ± 0.412
**PR 56**	H	H	F	H	H	H	H	H	H	C	0.876 ± 0.201
**PR 57**	H	CF_3_	H	H	H	H	H	H	H	C	1.025 ± 0.235
**PR 58**	OCH_3_	H	H	H	H	H	H	H	H	C	0.172 ± 0.041
**PR 59**	H	OCH_3_	H	H	H	H	H	H	H	C	1.964 ± 0.452
**PR 60**	H	H	OCH_3_	H	H	H	H	H	H	C	1.414 ± 0.326
**PR 61**	CH_3_	H	H	H	H	H	H	H	H	C	0.412 ± 0.095
**PR 62**	H	H	CH_3_	H	H	H	H	H	H	C	0.653 ± 0.151
**PR 63**	N(CH_3_)_2_	H	H	H	H	H	H	H	H	C	0.151 ± 0.035
**PR 64**	H	N(CH_3_)_2_	H	H	H	H	H	H	H	C	0.851 ± 0.192
**PR 65**	H	4-CH_3_-pip	H	H	H	H	H	H	H	C	4.703 ± 1.081
**PR 66**	H	morfoline	H	H	H	H	H	H	H	C	11.560 ± 2.656

**Table 5:** Binding data of synthesized compounds from the 5th group for 5-HT_6_R, with SD. Data are presented as a heat map where the highest affinities are marked in green, moderate in yellow, and the lowest in red.

	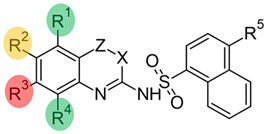							5-HT_6_
**Name**	**Z**	**X**	**R^1^**	**R^2^**	**R^3^**	**R^4^**	**R^5^**	***K*_i_ [µM]**
**PR 67**	CH(CH_3_)	NH	H	H	H	H	H	0.236 ± 0.058
**PR 68**	CH(CH_3_)	NH	Cl	H	H	H	H	0.037 ± 0.008
**PR 69**	CH(CH_3_)	NH	H	Cl	H	H	H	0.372 ± 0.093
**PR 70**	CH(CH_3_)	NH	H	H	Cl	H	H	0.985 ± 0.245
**PR 71**	CH(CH_3_)	NH	H	H	H	Cl	H	0.061 ± 0.014
**PR 72**	CH(CH_3_)	NH	H	Cl	H	Cl	H	0.997 ± 0.249
**PR 73**	CH(CH_3_)	NH	F	H	H	H	H	0.043 ± 0.011
**PR 74**	CH(CH_3_)	NH	H	Cl	H	Cl	Cl	1.038 ± 0.260
**PR 75**	CH(CH_3_)	NH	H	Br	H	H	H	0.484 ± 0.121
**PR 76**	CH(CH_3_)	NCH_3_	H	H	H	H	H	0.103 ± 0.025
**PR 77**	CH(CH_3_)	O	H	H	H	H	H	0.373 ± 0.092
**PR 78**	CH_2_CH_2_	NH	H	H	H	H	H	0.273 ± 0.068

**Table 6:** Extended receptor affinity study, with functional tests and cytotoxic activity of selected compounds on HEK293.

Name	D_2_	5-HT_1A_	5-HT_5A_	5-HT_6_			5-HT_7_	HEK293
	*K*_i_ [µM]	*K*_i_ [µM]	*K*_i_ [µM]	*K*_i_ [µM]	EC_50_ [µM]	*K*_B_ [µM]	*K*_i_ [µM]	IC_50_ [µM]
**PR 49**	9999.999	206.900	ND	0.077	NA	0.081	141.500	38.8
**PR 66**	ND	ND	3.444	ND	ND	ND	ND	NT
**PR 68**	622.100	35.080	1.111	0.037	NA	0.079	19.310	81.8
**PR 71**	141.900	134.300	ND	0.061	NA	0.035	37.450	NT
**PR 73**	145.800	80.000	ND	0.043	NA	0.134	35.780	NT
**PR 76**	173.900	11.000	ND	0.103	0.199	NA	35.630	NT
**PR 77**	222.400	7.539	ND	0.373	NA	NA	56.650	NT
**Ref.**	0.0009 ^a^	0.032 ^b^	0.061 ^c^	0.007 ^d^	-	0.0003 ^e^	0.062 ^f^	-

NA = not active, ND = not determined, NT = nontoxic in 50 µM, ^a^ ziprasidone, ^b^ buspirone, ^c^ SB269970, ^d^ olanzapine, ^e^ SB258585, ^f^ clozapine.

**Table 7:** Antiproliferative activity of selected compounds on a panel of cancer cells and normal astrocytes, with SD.

	IC_50_ Values [µM]							
Name	1321N1	U87MG	LN-229	U-251	MCF7	AsPC-1	NHA	PG-4
**PR 9**	>25	>25	ND	ND	ND	ND	ND	ND
**PR 11**	>25	>25	>25	>25	>25	>25	ND	ND
**PR 17**	>25	>25	ND	ND	ND	ND	ND	ND
**PR 19**	>25	>25	>25	>25	>25	>25	ND	ND
**PR 30**	ND	ND	>25	>25	>25	>25	ND	ND
**PR 36**	>25	>25	>25	>25	>25	>25	ND	ND
**PR 41**	>25	>25	ND	ND	ND	ND	ND	ND
**PR 42**	>25	>25	ND	ND	ND	ND	ND	ND
**PR 43**	>25	>25	ND	ND	ND	ND	ND	ND
**PR 44**	>25	>25	ND	ND	ND	ND	ND	ND
**PR 46**	>25	>25	ND	ND	ND	ND	ND	ND
**PR 47**	>25	>25	ND	ND	ND	ND	ND	ND
**PR 49**	>25	>25	>25	>25	>25	>25	ND	ND
**PR 51**	>25	>25	ND	ND	ND	ND	ND	ND
**PR 53**	>25	>25	>25	>25	>25	>25	ND	ND
**PR 54**	>25	>25	ND	ND	ND	ND	ND	ND
**PR 58**	>25	>25	>25	>25	>25	>25	ND	ND
**PR 61**	>25	>25	ND	ND	ND	ND	ND	ND
**PR 64**	>25	>25	>25	**7.7 ± 0.8**	**9.7 ± 1.7**	>25	**15.1 ± 2.1**	ND
**PR 66**	>25	>25	>25	>25	>25	>25	ND	ND
**PR 67**	>25	>25	ND	ND	ND	ND	ND	ND
**PR 68**	>25	>25	>25	>25	>25	>25	ND	>25
**PR 71**	>25	>25	>25	>25	>25	>25	ND	ND
**PR 73**	>25	>25	>25	>25	>25	>25	ND	>25
**PR 74**	**13.4 ± 0.8**	**17.0 ± 0.4**	ND	ND	ND	ND	ND	ND
**PR 75**	>25	>25	ND	ND	ND	ND	ND	ND
**PR 76**	>25	>25	>25	>25	>25	>25	ND	ND
**PR 77**	>25	>25	>25	>25	>25	>25	ND	ND
**PR 78**	>25	>25	>25	>25	>25	>25	ND	ND
**Doxorubicin**	1.1	ND	ND	ND	ND	ND	ND	ND
**Gefitinib**	ND	ND	>25	24.0	>25	ND	ND	ND
**Afatinib**	ND	ND	9.8	27.0	25.9	ND	7.5	ND

ND = not determined.

**Table 8:** Lipophilicity, affinity to phospholipids, and binding to plasma proteins determined by GlaxoSmithKline (Valko) protocol.

	CHI C18					
Name	pH 2.6	pH 7.4	pH 10.5	CHI IAM	logKHSA	%HSA
**PR 11**	78.8	76.7	58.4	39.3	1.3	95.7
**PR 41**	95.8	93.1	96.2	42.9	1.4	97.1
**PR 42**	100.1	99.2	101.6	45.5	1.5	97.9
**PR 44**	98.2	96.5	99.5	44.3	1.6	98.3
**PR 54**	90.8	89.4	92.4	40.8	1.3	96.4
**PR 58**	92.8	90.8	96.6	41.4	1.4	97.3
**PR 68**	101.4	99.2	103.0	45.8	1.5	97.7
**PR 71**	102.2	100.7	103.8	44.8	1.4	97.5
**PR 73**	94.6	94.9	96.5	42.3	1.3	96.6
**PR 74**	125.7	123.9	128.1	45.2	ND	ND
**PR 77**	102.5	99.9	103.2	40.8	1.3	95.9

**Table 9:** ADME parameters for lead compounds.

	PAMPA	Metabolic Stability	PPB	
**Compound**	**Pe (10^−6^ cm/s)**	**% Remaining**	**kD [μM]**	**fb [%] ± SD**
**PR 68**	1.72	51.06	1.4	99.8 ± 0.13%
**PR 73**	ND	24.59	ND	ND
**PR 76**	ND	87.40	ND	ND

kD = dissociation constant, fb = fraction bound, reference compounds: caffeine Pe = 8.23 (10^−6^ cm/s); verapamil remaining = 23.93%; warfarin kD = 9.5 [μM], fb = 98.5 ± 2.30 [%] ± SD. ND = not determined.

## Data Availability

No new data were created or analyzed in this study. Data sharing is not applicable to this article.

## Supplementary Materials

The following supporting information can be downloaded at: https://www.mdpi.com/article/10.3390/ijms251910287/s1.
