# Supplementary material for: Low-Basicity 5-HT6 Receptor Ligands from the Group of Cyclic Arylguanidine Derivatives and Their Antiproliferative Activity Evaluation
Source: Int J Mol Sci. 2024 Sep 24;25(19):10287. doi: 10.3390/ijms251910287 (PMC11477289; doi:10.3390/ijms251910287)
Supplement: Supplementary file 1 [file ijms-25-10287-s001.zip › SI_biological_research.pdf]

Supplementary materials - Biological research

1. 5-HTR assays

1.1. Representative curves for the 5-HT_6R binding.

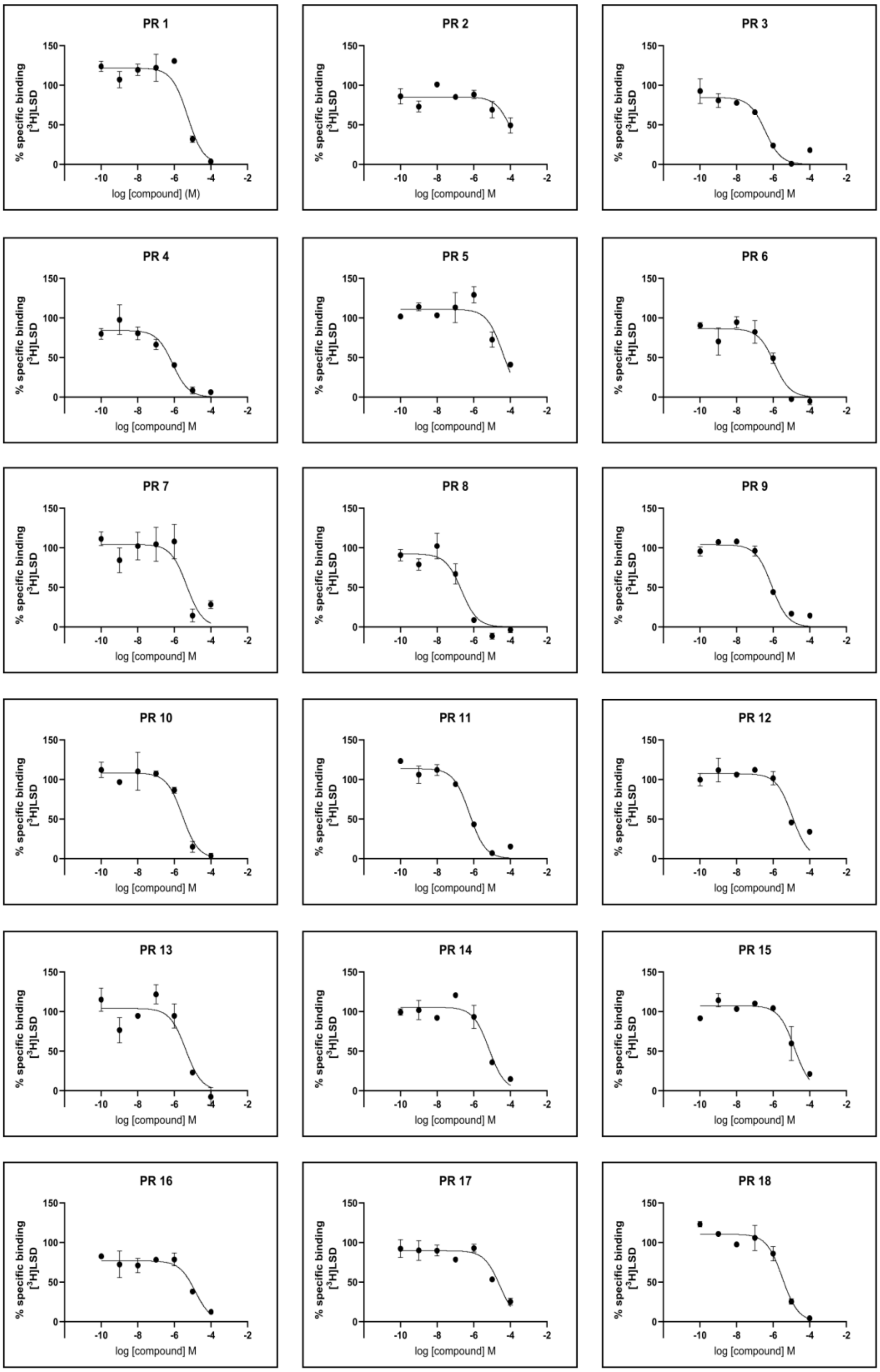

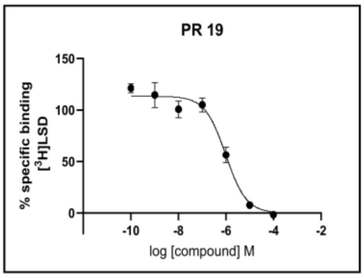
PR 19
% specific binding
[3H]LSD
150
100
50
0
-10
-8
-6
-4
-2
log [compound] M

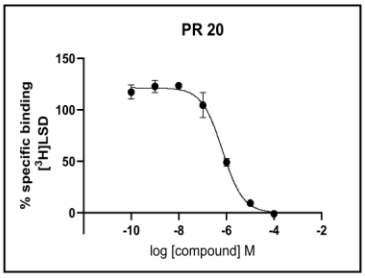
PR 20
% specific binding
[3H]LSD
150
100
50
0
-10
-8
-6
-4
-2
log [compound] M

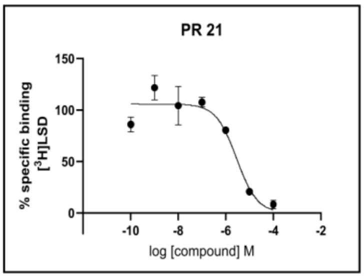
PR 21
% specific binding
[3H]LSD
150
100
50
0
-10
-8
-6
-4
-2
log [compound] M

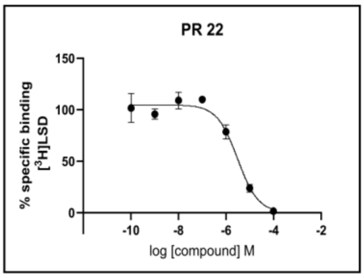
PR 22
% specific binding
[3H]LSD
150
100
50
0
-10
-8
-6
-4
-2
log [compound] M

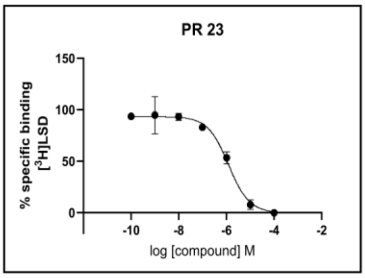
PR 23
% specific binding
[3H]LSD
150
100
50
0
-10
-8
-6
-4
-2
log [compound] M

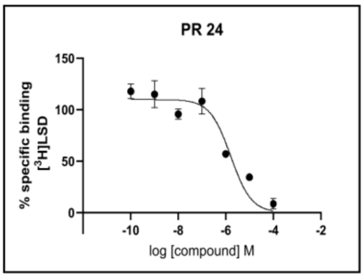
PR 24
% specific binding
[3H]LSD
150
100
50
0
-10
-8
-6
-4
-2
log [compound] M

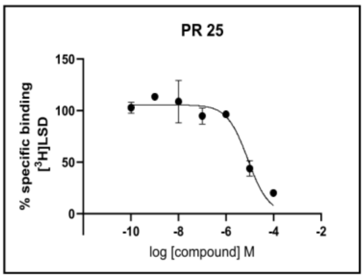
PR 25
% specific binding
[3H]LSD
150
100
50
0
-10
-8
-6
-4
-2
log [compound] M

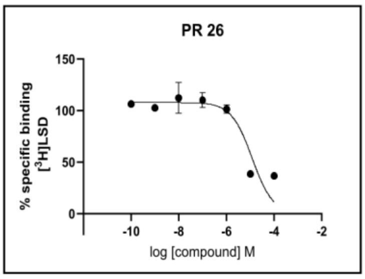
PR 26
% specific binding
[3H]LSD
150
100
50
0
-10
-8
-6
-4
-2
log [compound] M

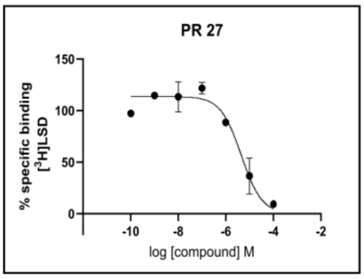
PR 27
% specific binding
[3H]LSD
150
100
50
0
-10
-8
-6
-4
-2
log [compound] M

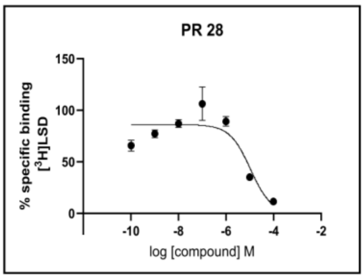
PR 28
% specific binding
[3H]LSD
150
100
50
0
-10
-8
-6
-4
-2
log [compound] M

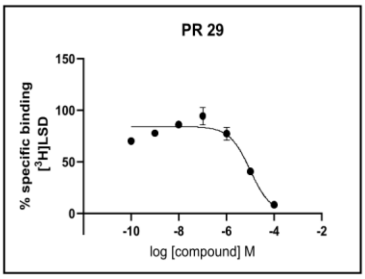
PR 29
% specific binding
[3H]LSD
150
100
50
0
-10
-8
-6
-4
-2
log [compound] M

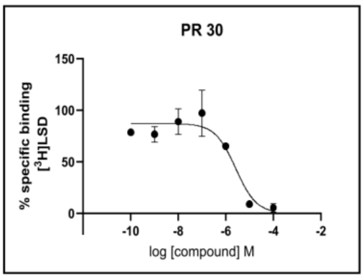
PR 30
% specific binding
[3H]LSD
150
100
50
0
-10
-8
-6
-4
-2
log [compound] M

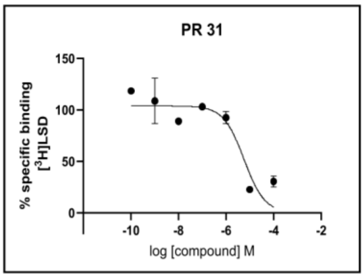
PR 31
% specific binding
[3H]LSD
150
100
50
0
-10
-8
-6
-4
-2
log [compound] M

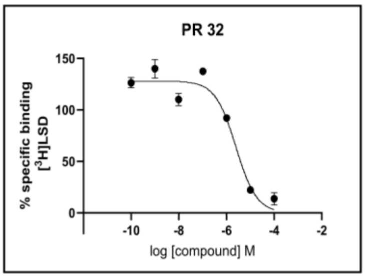
PR 32
% specific binding
[3H]LSD
150
100
50
0
-10
-8
-6
-4
-2
log [compound] M

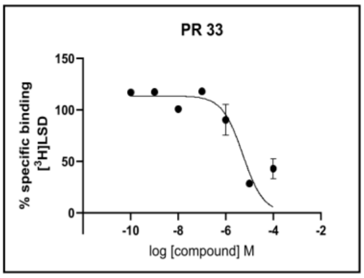
PR 33
% specific binding
[3H]LSD
150
100
50
0
-10
-8
-6
-4
-2
log [compound] M

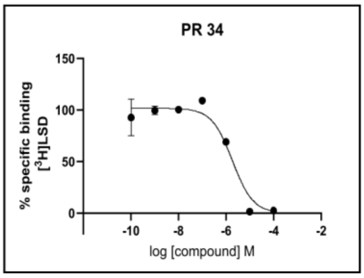
PR 34
% specific binding
[3H]LSD
150
100
50
0
-10
-8
-6
-4
-2
log [compound] M

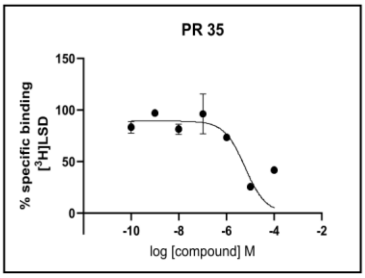
PR 35
% specific binding
[3H]LSD
150
100
50
0
-10
-8
-6
-4
-2
log [compound] M

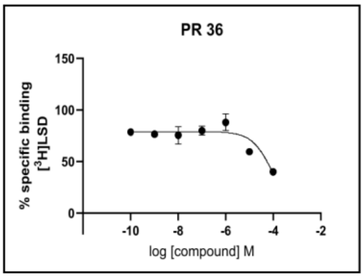
PR 36
% specific binding
[3H]LSD
150
100
50
0
-10
-8
-6
-4
-2
log [compound] M

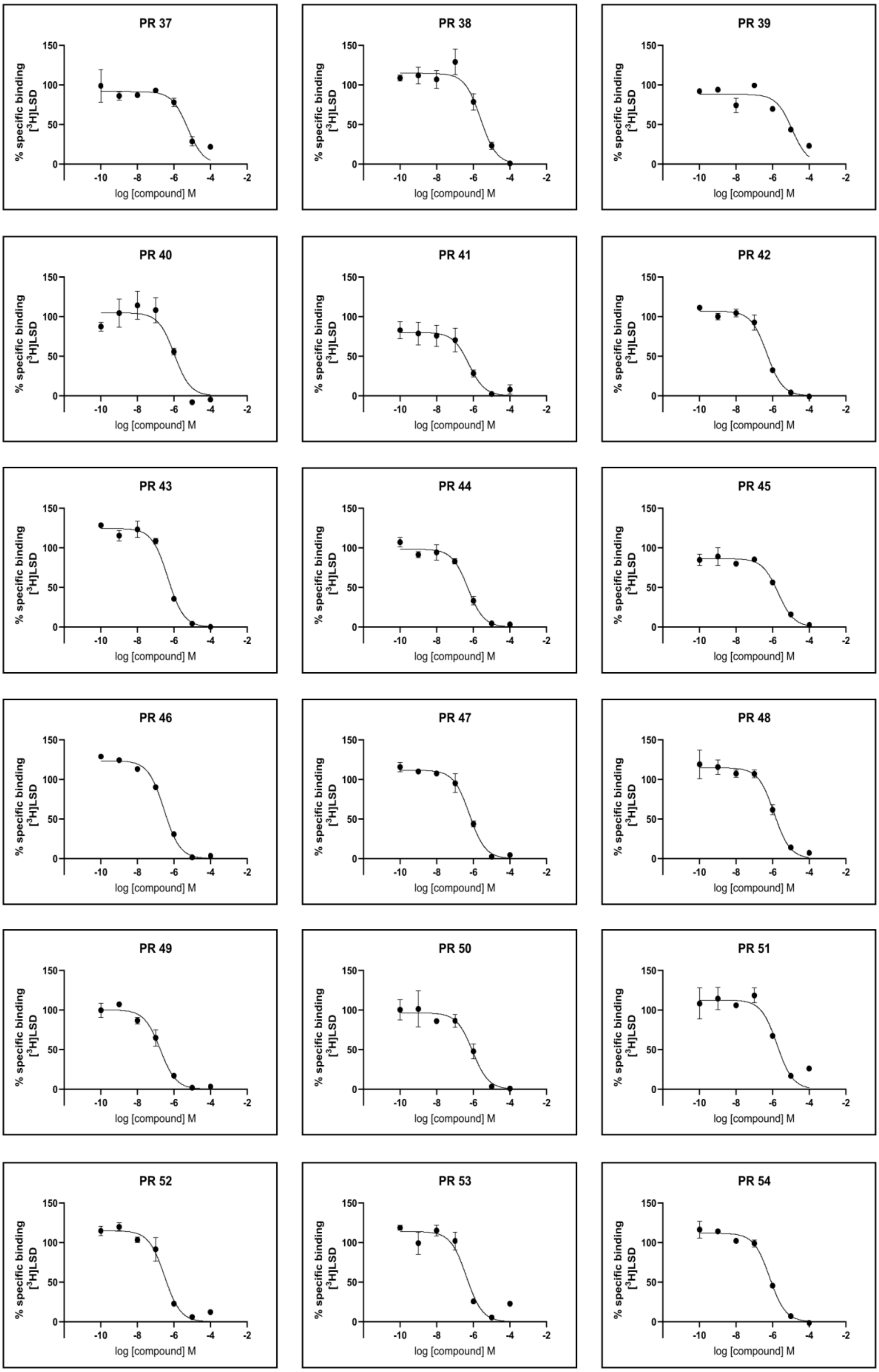
PR 37
PR 38
PR 39
PR 40
PR 41
PR 42
PR 43
PR 44
PR 45
PR 46
PR 47
PR 48
PR 49
PR 50
PR 51
PR 52
PR 53
PR 54
% specific binding
[3H]LSD
log [compound] M
150
100
50
0
-10
-8
-6
-4
-2

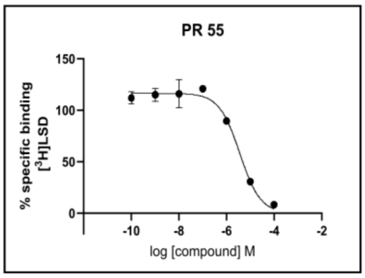
PR 55
% specific binding [3H]LSD
150
100
50
0
-10
-8
-6
-4
-2
log [compound] M

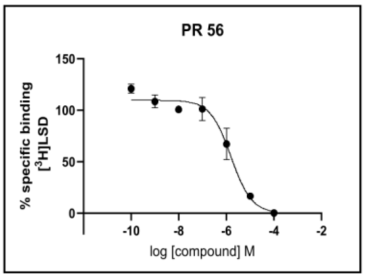
PR 56
% specific binding [3H]LSD
150
100
50
0
-10
-8
-6
-4
-2
log [compound] M

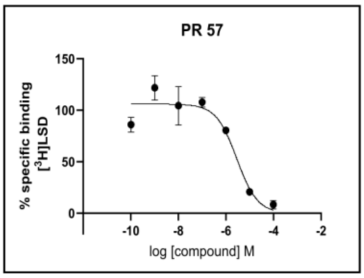
PR 57
% specific binding [3H]LSD
150
100
50
0
-10
-8
-6
-4
-2
log [compound] M

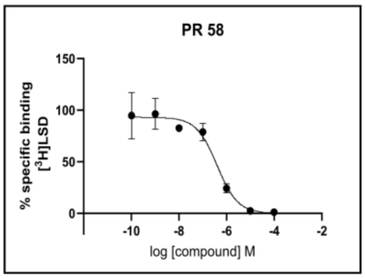
PR 58
% specific binding [3H]LSD
150
100
50
0
-10
-8
-6
-4
-2
log [compound] M

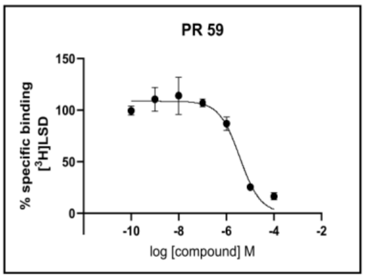
PR 59
% specific binding [3H]LSD
150
100
50
0
-10
-8
-6
-4
-2
log [compound] M

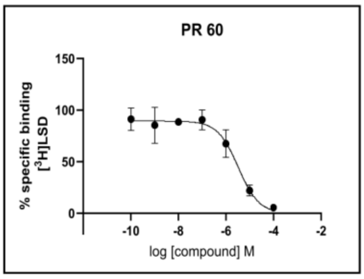
PR 60
% specific binding [3H]LSD
150
100
50
0
-10
-8
-6
-4
-2
log [compound] M

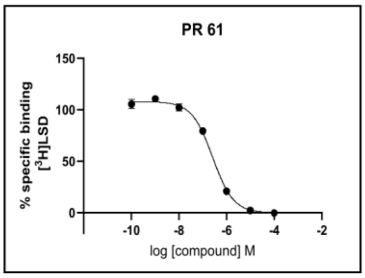
PR 61
% specific binding [3H]LSD
150
100
50
0
-10
-8
-6
-4
-2
log [compound] M

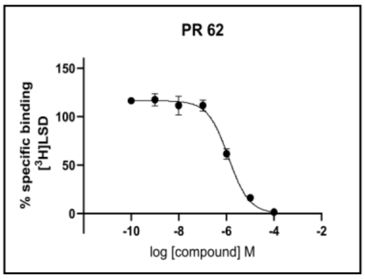
PR 62
% specific binding [3H]LSD
150
100
50
0
-10
-8
-6
-4
-2
log [compound] M

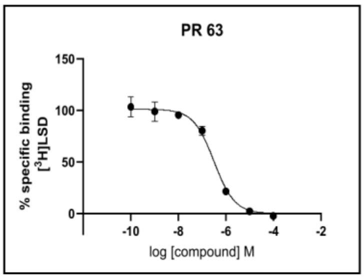
PR 63
% specific binding [3H]LSD
150
100
50
0
-10
-8
-6
-4
-2
log [compound] M

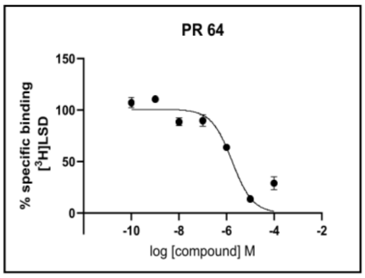
PR 64
% specific binding [3H]LSD
150
100
50
0
-10
-8
-6
-4
-2
log [compound] M

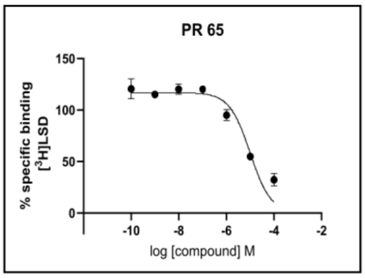
PR 65
% specific binding [3H]LSD
150
100
50
0
-10
-8
-6
-4
-2
log [compound] M

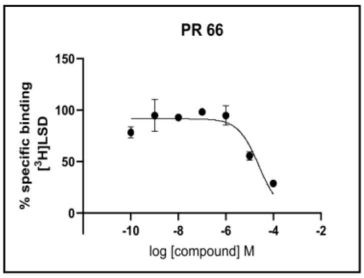
PR 66
% specific binding [3H]LSD
150
100
50
0
-10
-8
-6
-4
-2
log [compound] M

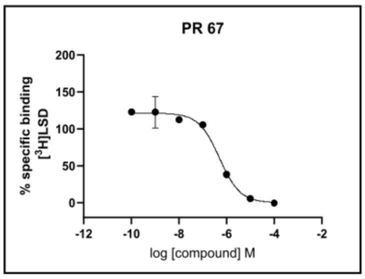
PR 67
% specific binding [3H]LSD
200
150
100
50
0
-12
-10
-8
-6
-4
-2
log [compound] M

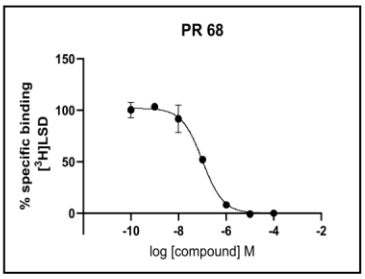
PR 68
% specific binding [3H]LSD
150
100
50
0
-10
-8
-6
-4
-2
log [compound] M

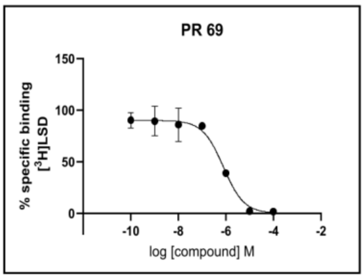
PR 69
% specific binding [3H]LSD
150
100
50
0
-10
-8
-6
-4
-2
log [compound] M

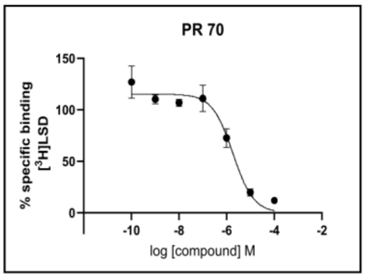
PR 70
% specific binding [3H]LSD
150
100
50
0
-10
-8
-6
-4
-2
log [compound] M

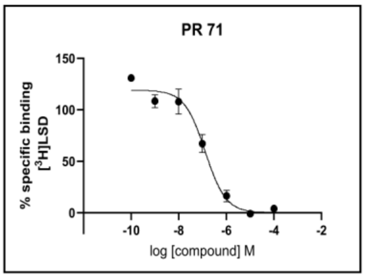
PR 71
% specific binding [3H]LSD
150
100
50
0
-10
-8
-6
-4
-2
log [compound] M

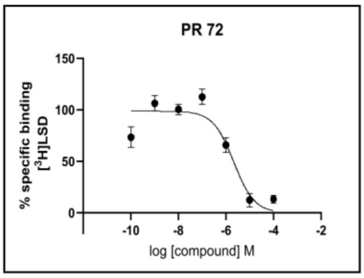
PR 72
% specific binding [3H]LSD
150
100
50
0
-10
-8
-6
-4
-2
log [compound] M

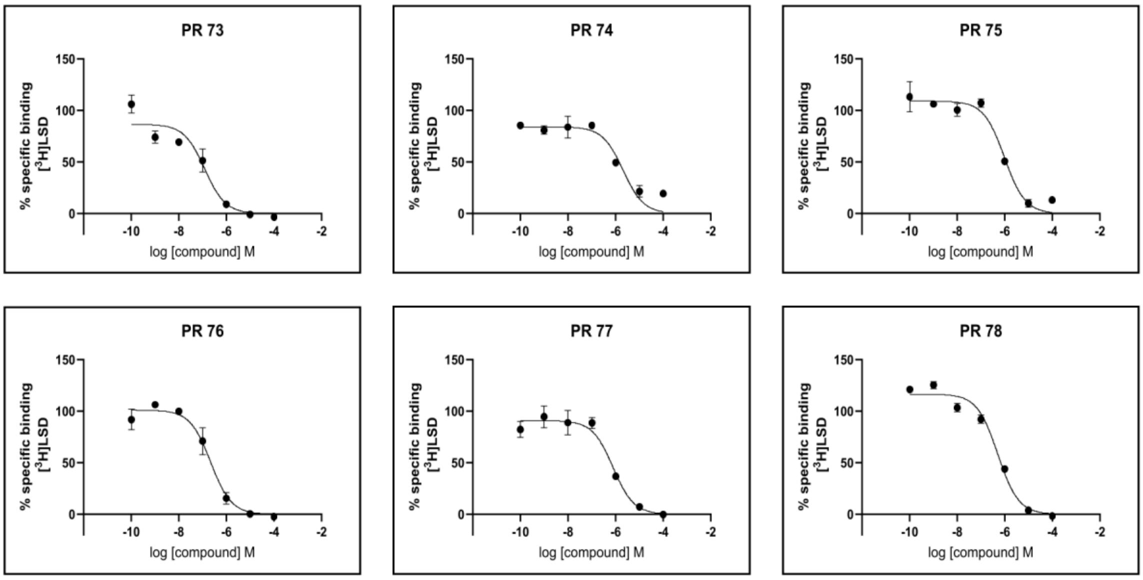

1.2. Representative curves for the 5-HT$_{1A}$R binding.

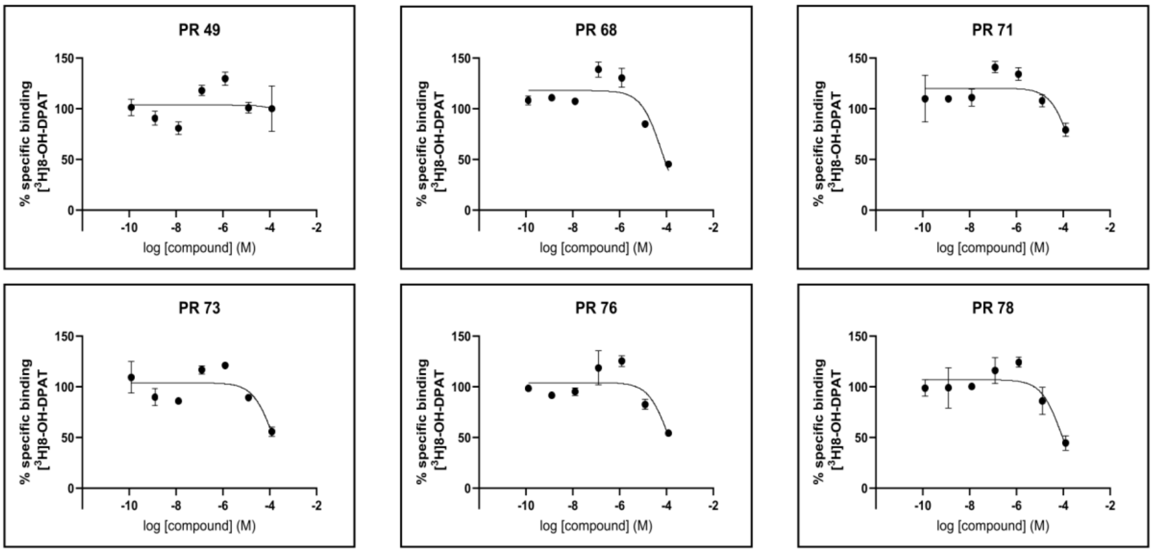

1.3. Representative curves for the 5-HT$_{5A}$R binding.

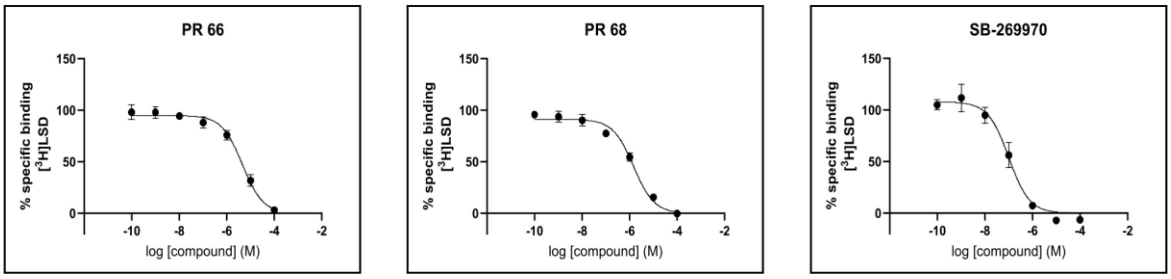

1.4. Representative curves for the 5-HT_7R binding.

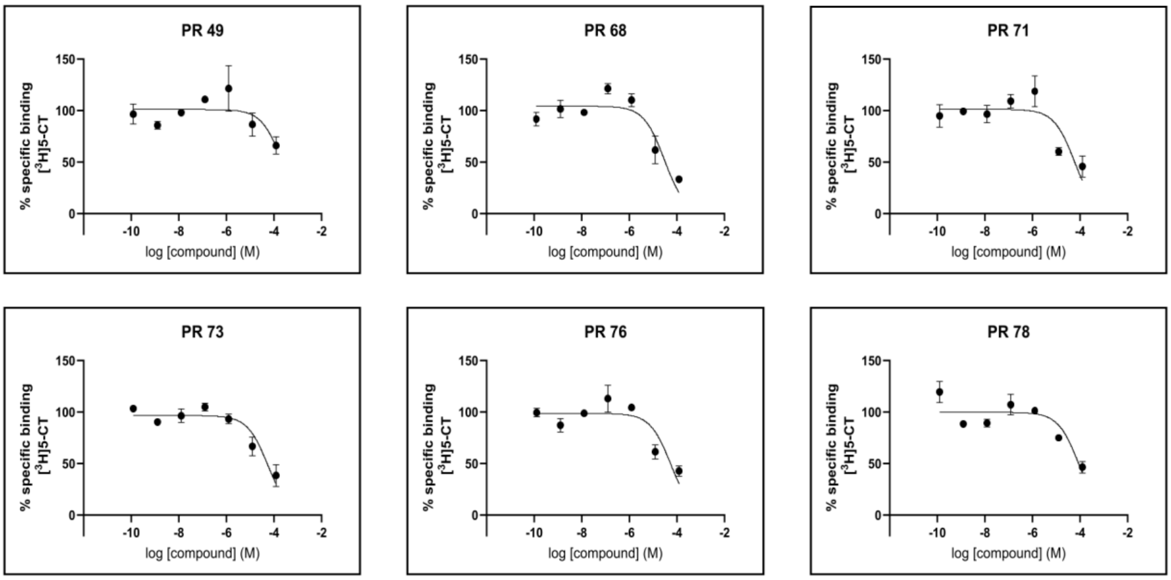

1.5. Representative curves for the D_2R binding.

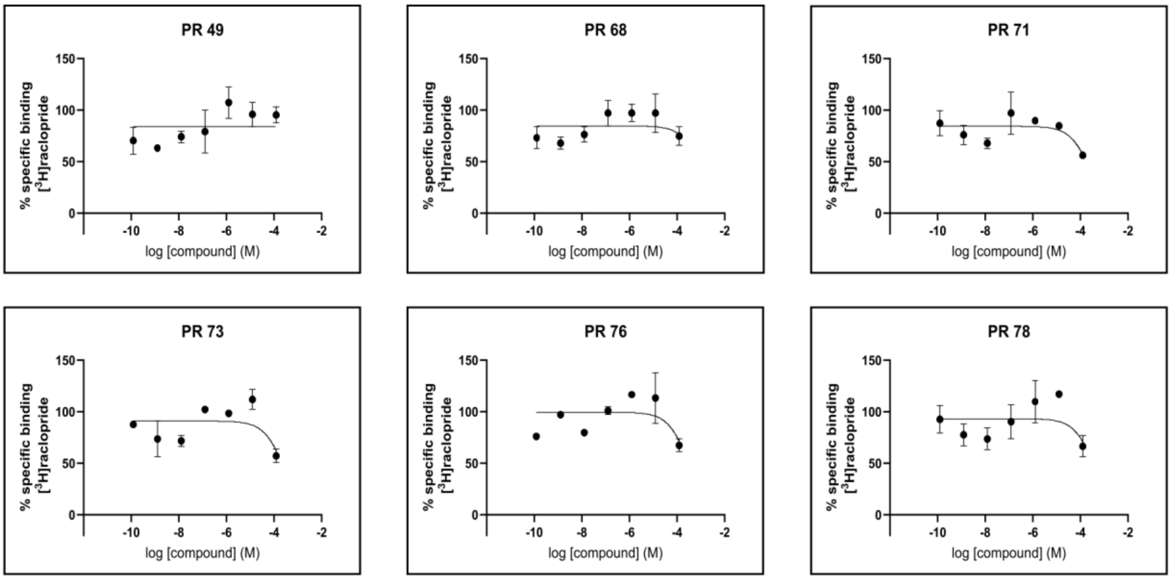

1.6. Representative curves for the 5-HT$_6$R functional tests.

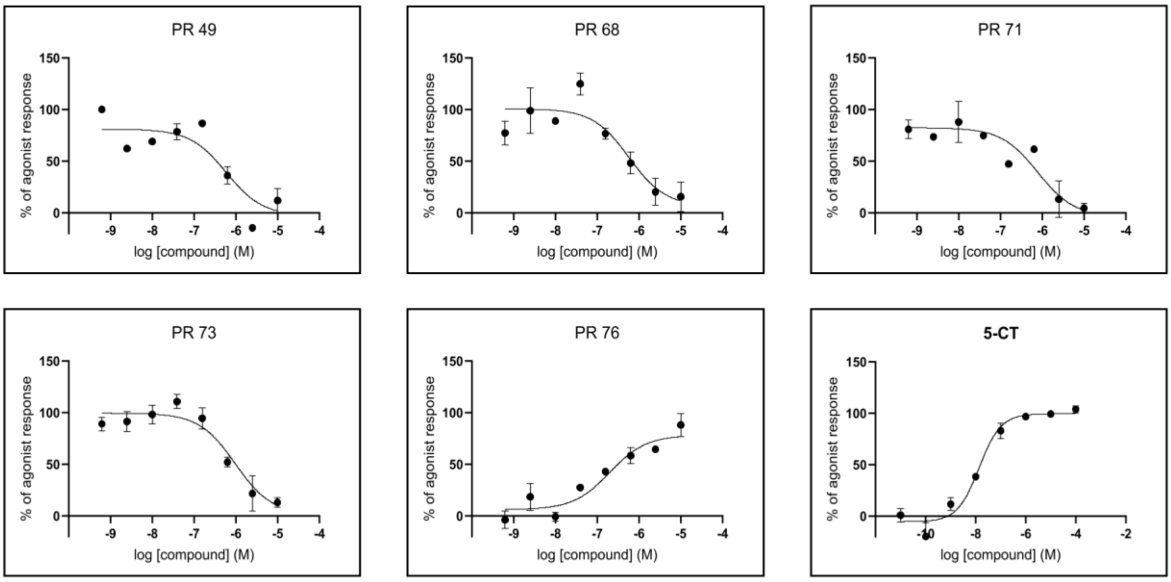

1.7. Effect of tested ligands on the viability on the HEK293.

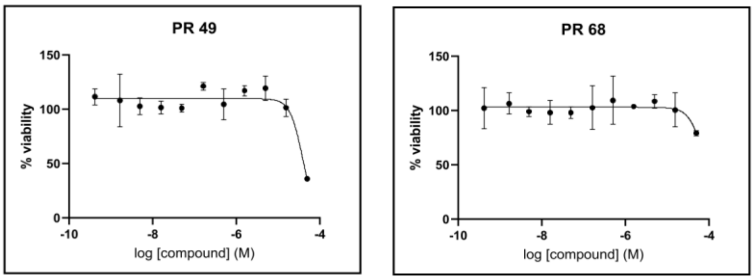

2. Cytotoxicity

Effect of tested ligands on the viability on the **A** 1321 N1 human astrocytoma cell line **B** U87MG glioblastoma multiforme line. The cells were exposed to either vehicle (0.1 % DMSO), or inhibitor in 25 µM for 72 h. Then, cell viability was assessed by the means of MTS assay. In **C** and **D**, the cytotoxic properties of the compound **PR 74** and in E and F cytotoxic properties of the compound PR 64 were studied in dose-dependent manner. The IC_{50} were calculated as a dose that causes a 50 % decrease in cell viability relative to the maximum inhibition observed.

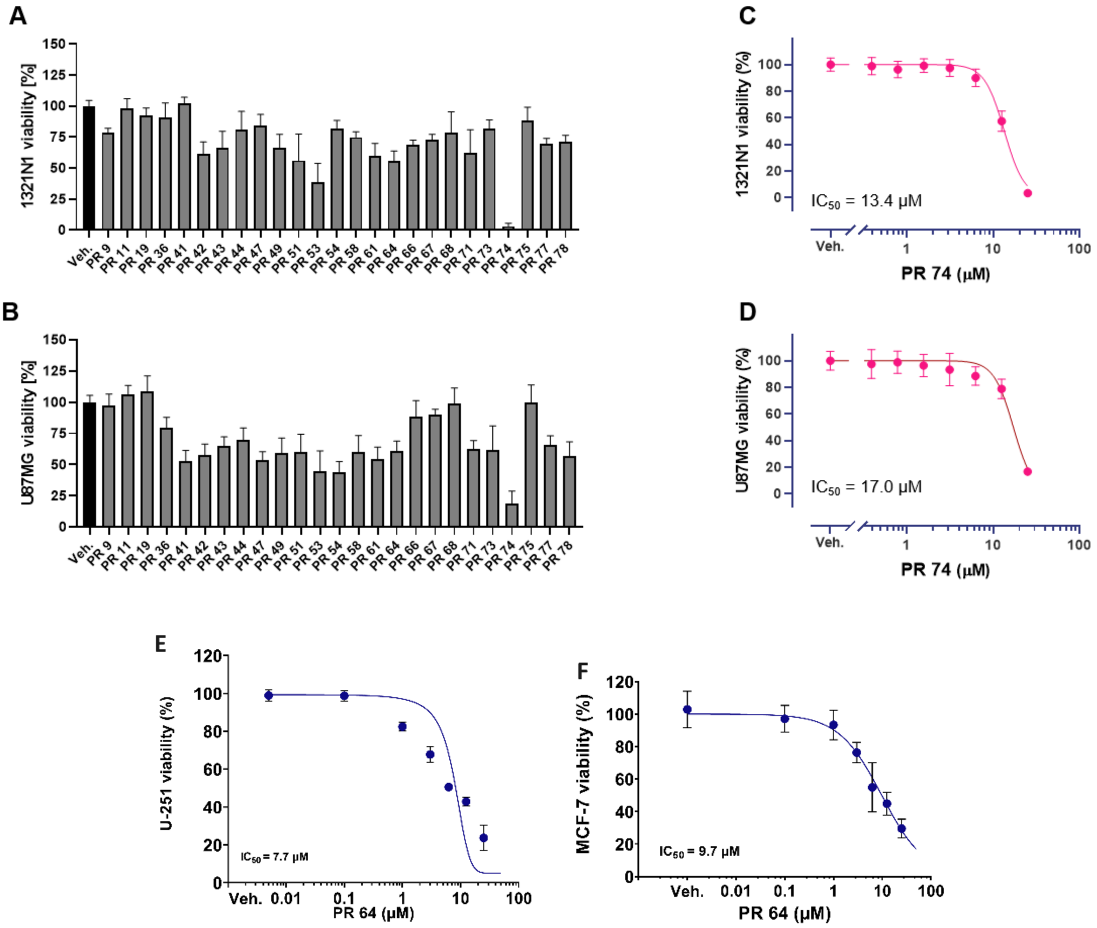

3. Solubility

Solubility in DMSO and 10% DMSO in PBS buffer.

	100% DMSO, 10 mM	10% DMSO, 1 mM
**PR 68**	**+ + -**	**+ - -**

4. Absorption – PAMPA permeability test

Comp.	*Pe** (10^{-6} cm/s)
CFN**	8.23
**PR 68**	**1.72**

*Permeability coefficient. Compound is permeable if **Pe≥1.5×10^{-6} cm/s**

**CFN = caffeine, well-permeable control

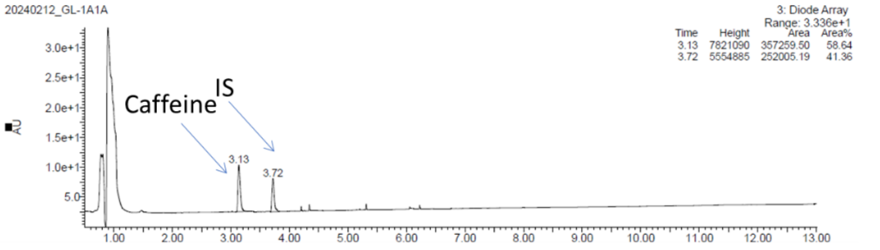

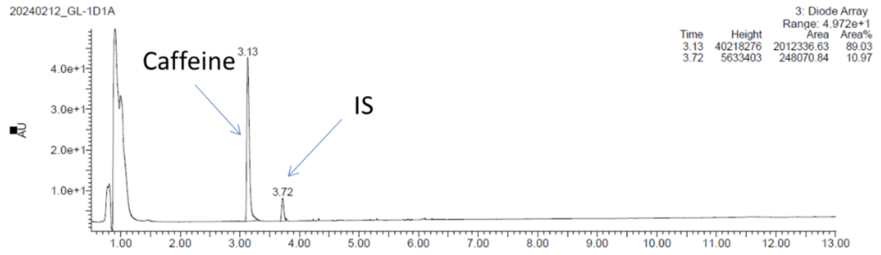

UPLC of **Caffeine** solution in PBS (pH=7.4) after 5h of incubation: acceptor well (above), donor well (below). IS – internal standard.

.

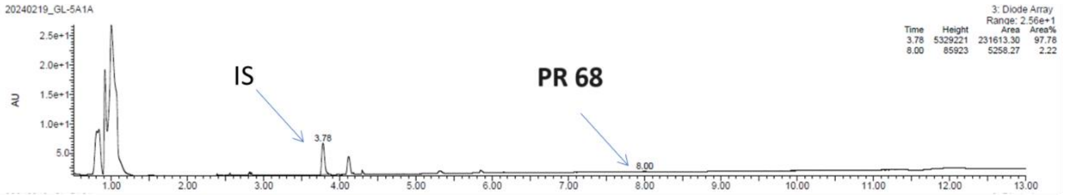

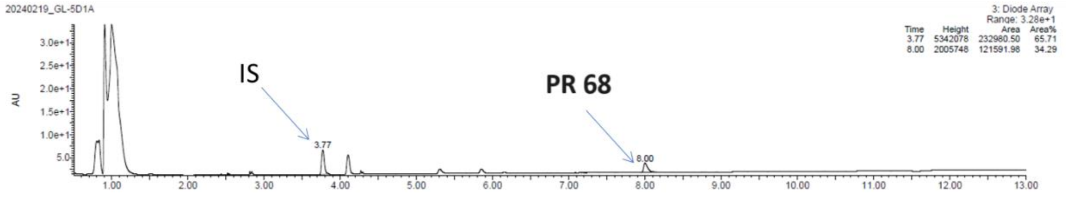

UPLC of **PR 68** solution in PBS (pH=7.4) after 5h of incubation: acceptor well (above), donor well (below). IS – internal standard.

5. Metabolic stability

Metabolic stability summary: the % remaining, molecular masses, and metabolic pathways of tested compounds after incubation with mouse liver microsomes (MLMs). Main metabolic pathways are marked in red.

Substrate	Molecular mass (*m/z*)	% remaining	Molecular mass of the metabolite (*m/z*)	Metabolic pathway
**PR 68**	386.04	**51.06**	402.07 (**M1**)<br>420.02 (**M2**)<br>420.02 (**M3**)<br>436.05 (**M4**)<br>243.08 (**M5**)<br>243.01 (**M6**)<br>243.01 (**M7**)<br>401.67 (**M8**)<br>258.91 (**M9**)	***hydroxylation***<br>*double hydroxylation and double bound reduction*<br>*double hydroxylation and double bound reduction*<br>*triple hydroxylation and double bound reduction*<br>*decomposition*<br>*decomposition*<br>*double hydroxylation*<br>*decomposition*
**PR 73**	369.94	**24.59**	371.14 (**M1**)<br>386.10 (**M2**)	***double bound reduction***<br>*hydroxylation*
**PR 76**	366.15	**87.40**	397.94 (**M1**)	***double hydroxylation***

* Reference unstable drug

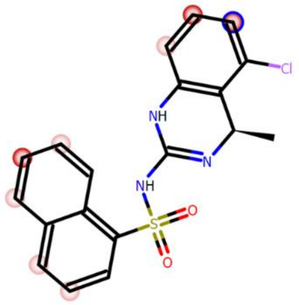

The MetaSite 6.0.1. software prediction of the most probable sites of compound **PR 68** metabolism. The darker red color - the higher probability to be involved in the metabolism pathway. The blue circle marked the site of compound with the highest probability of metabolic bioconversion.

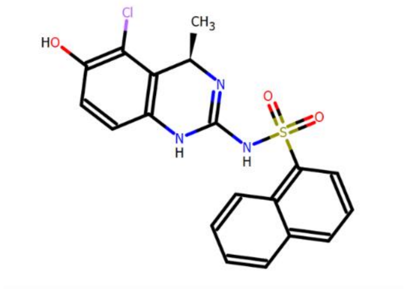

100%

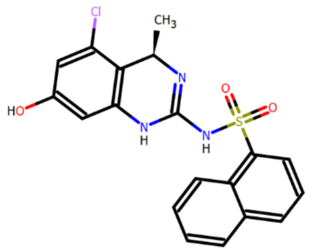

83.8%

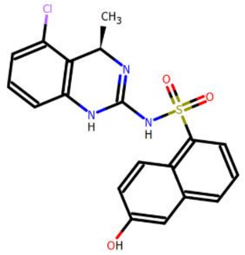

61.7%

The MetaSite 6.0.1. software prediction of the most probable hydroxylation site of **PR 68**

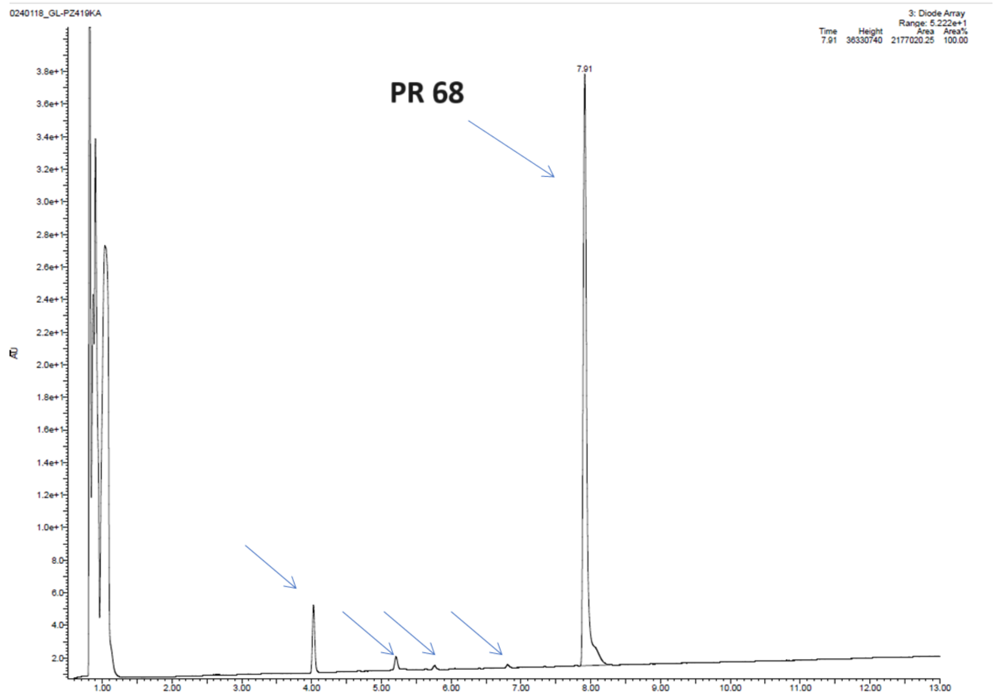

UPLC spectra after 120 min incubation of compound **PR 68** in buffer. Contaminations determined.

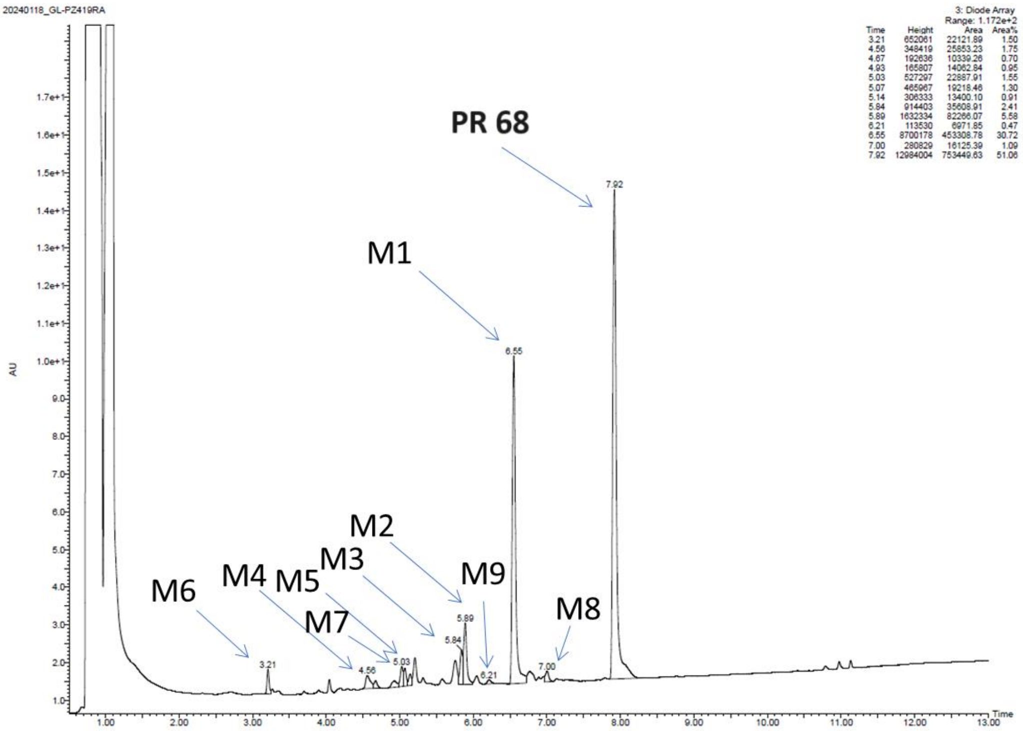

UPLC spectra after 120 min incubation of compound **PR 68** with MLMs.

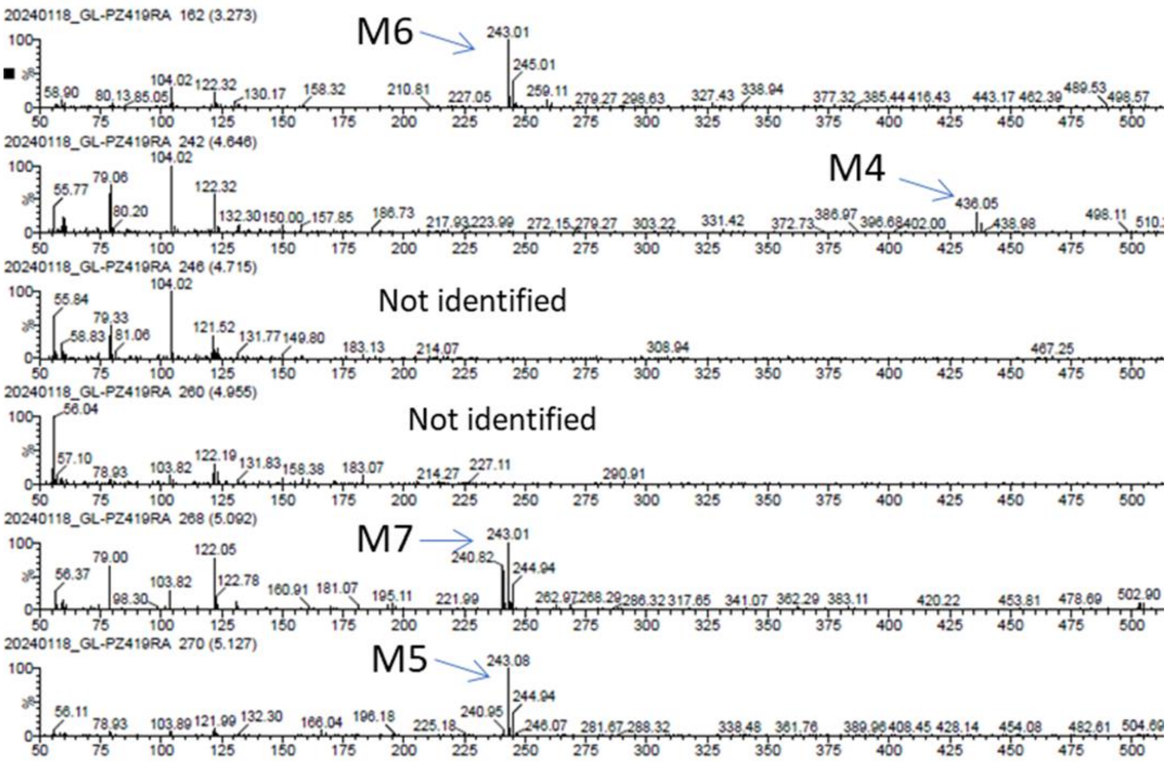

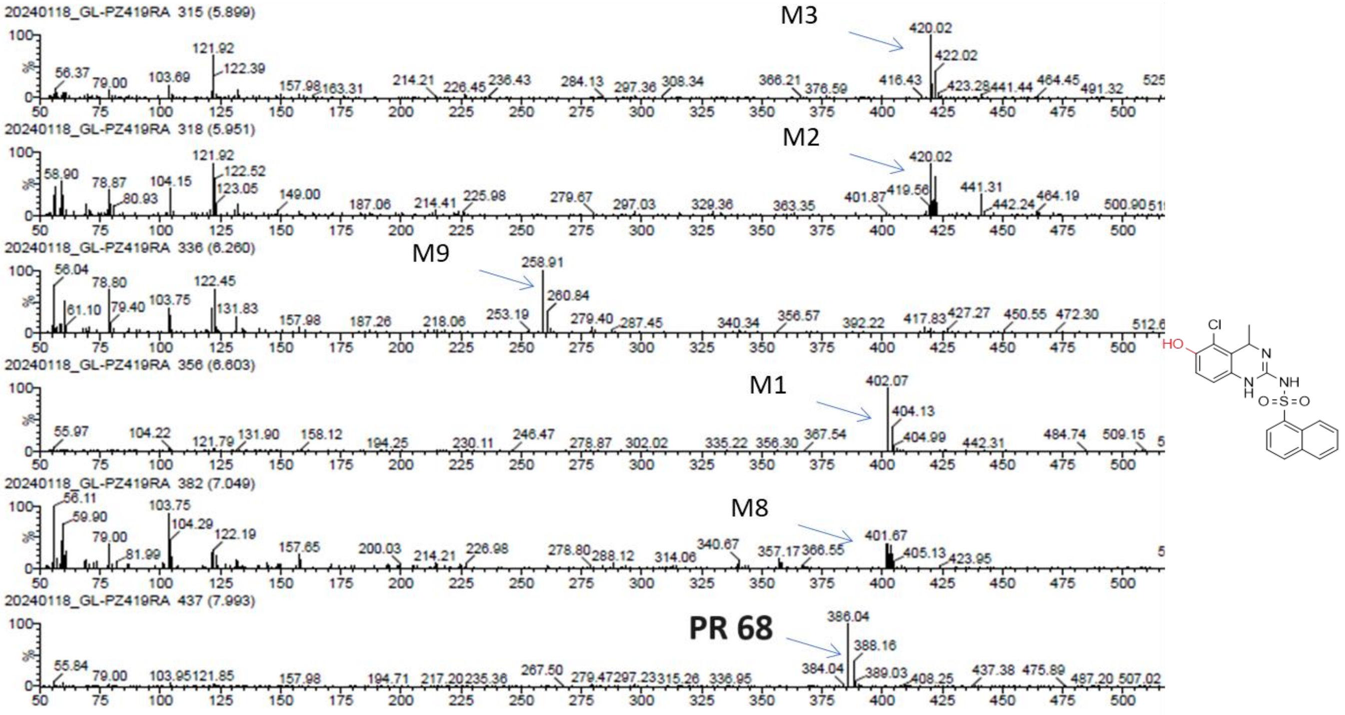

MS analyses of **PR 68** and metabolites.

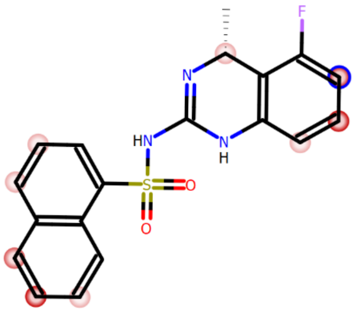

The MetaSite 6.0.1. software prediction of the most probable sites of compounds metabolism. The darker red color - the higher probability to be involved in the metabolism pathway of **PR 73**. The blue circle marked the site of compound with the highest probability of metabolic bioconversion.

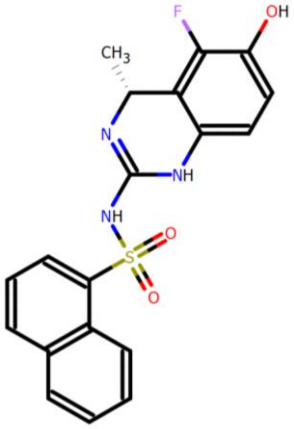

The MetaSite 6.0.1. software prediction of the most probable hydroxylation site of **PR 73.**

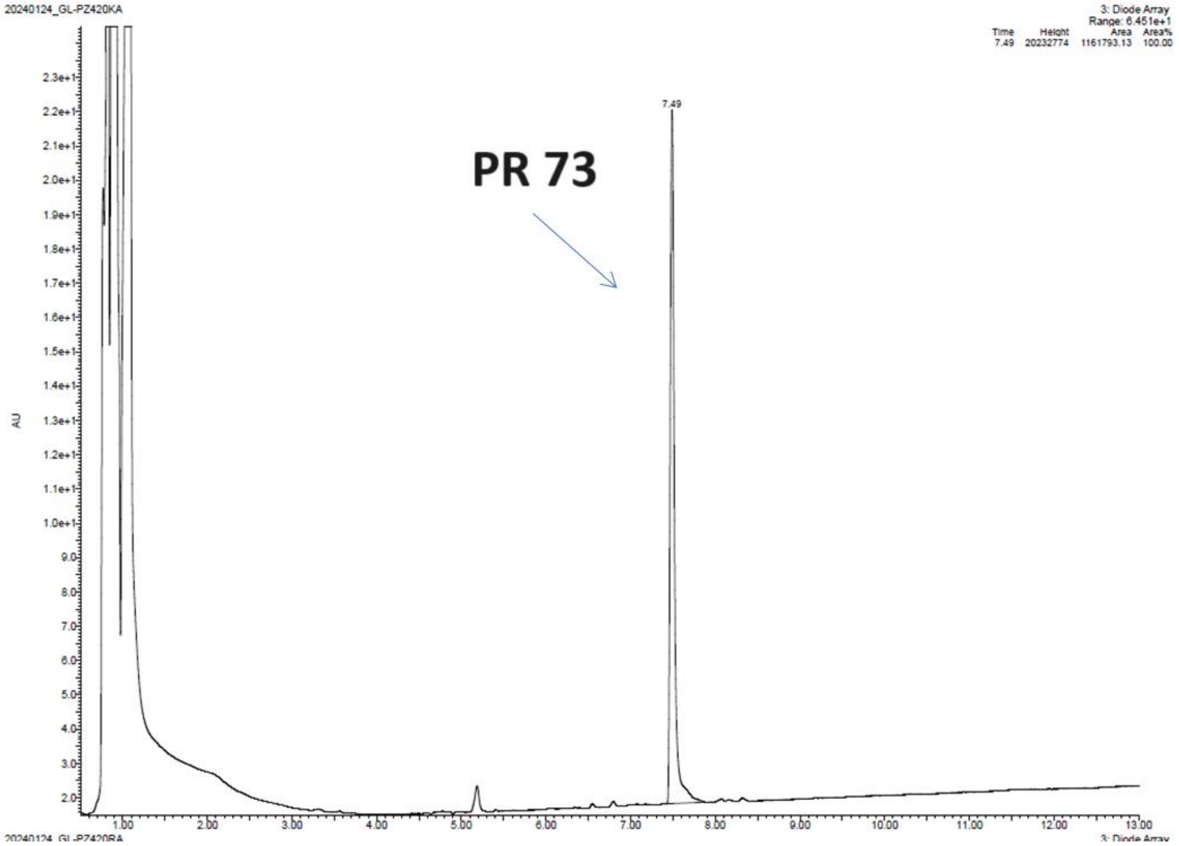

UPLC spectra after 120 min incubation of compound of **PR 73** in buffer

3: Diode Array
Range: 1.235e+2

Time	Height	Area	Area%
6.03	299898	15012.07	33.98
6.24	115636	8580.59	19.42
7.49	239915	10862.08	24.59
8.55	65518	4504.91	10.20
8.68	44188	2141.31	4.85
8.74	48759	3074.72	6.96

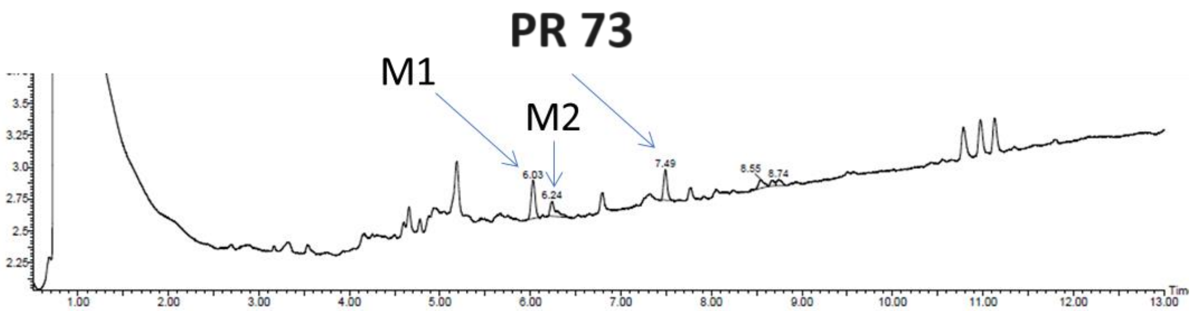

UPLC spectra after 120 min incubation of compound of **PR 73** with MLMs.

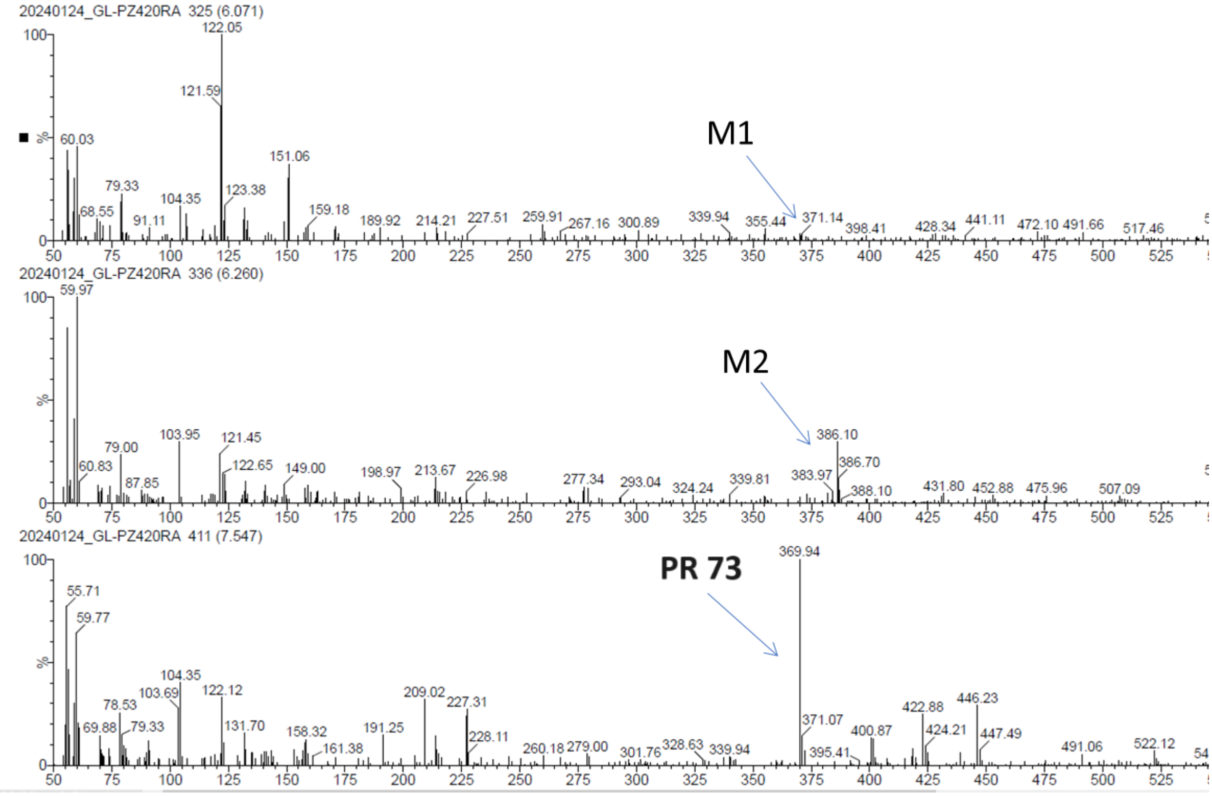

MS analyses of **PR 73** and metabolites.

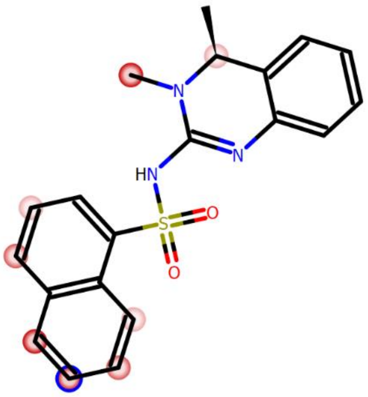

The MetaSite 6.0.1. software prediction of the most probable sites of compound **PR 76** metabolism. The darker red color - the higher probability to be involved in the metabolism pathway. The blue circle marked the site of compound with the highest probability of metabolic bioconversion.

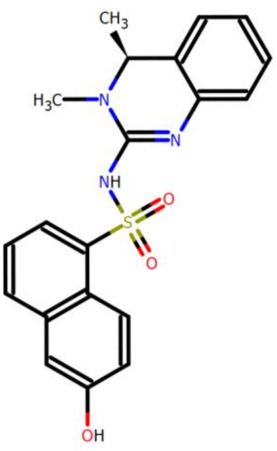

The MetaSite 6.0.1. software prediction of the most probable hydroxylation site of **PR 76.**

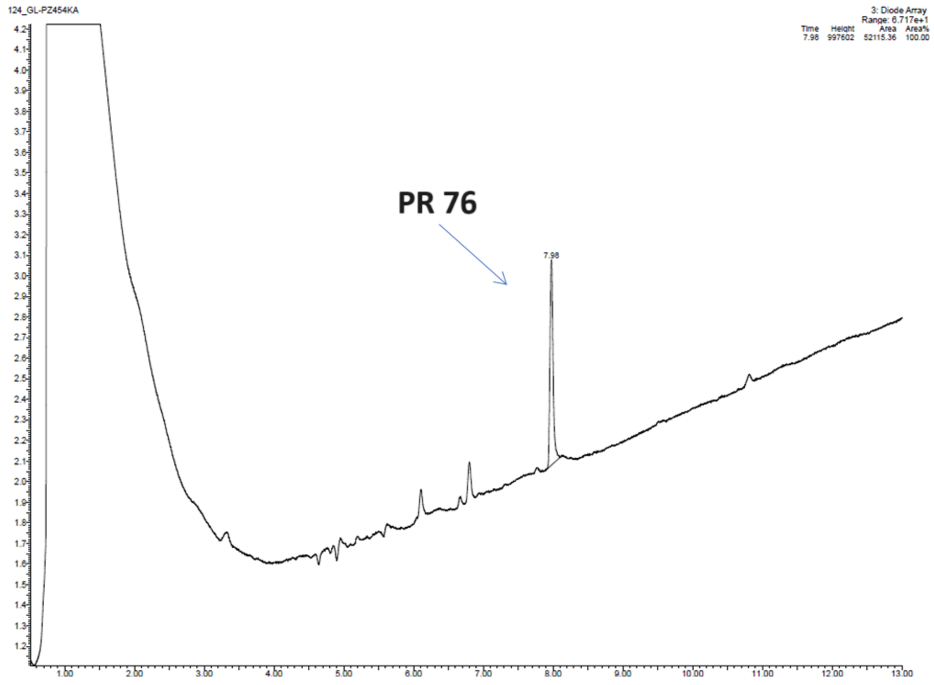

UPLC spectra after 120 min incubation of compound **PR 76** in buffer.

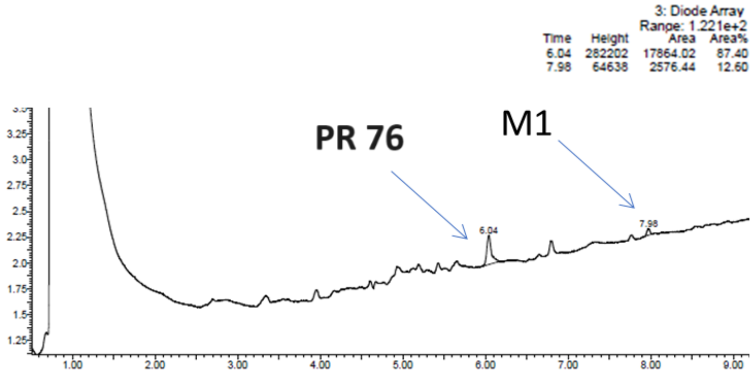

UPLC spectra after 120 min incubation of compound **PR 76** with MLMs.

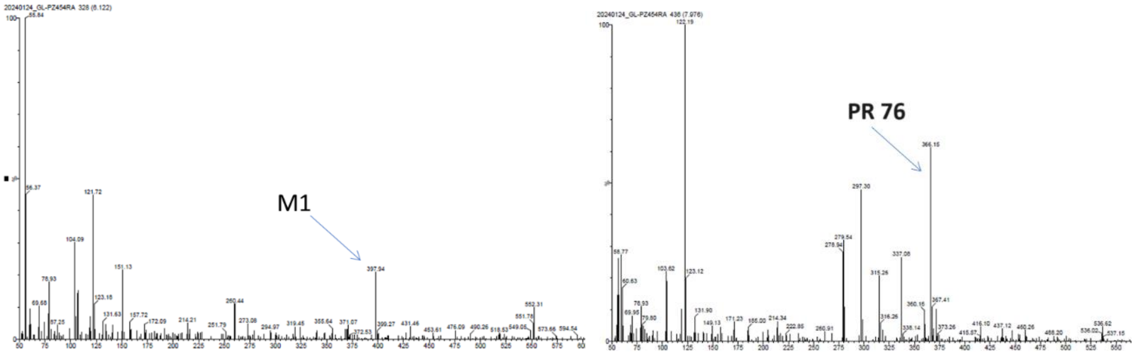

MS analyses of **PR 76** and metabolite.

6. Distribution - plasma protein binding (PPB)

Comp.	k_D µM	f_b % ± SD
Warfarin	9.5	98.5 ± 2.30
**PR 68**	**1.4**	**99.8** ± 0.13

k_D = dissociation constant, **f_b =** fraction bound

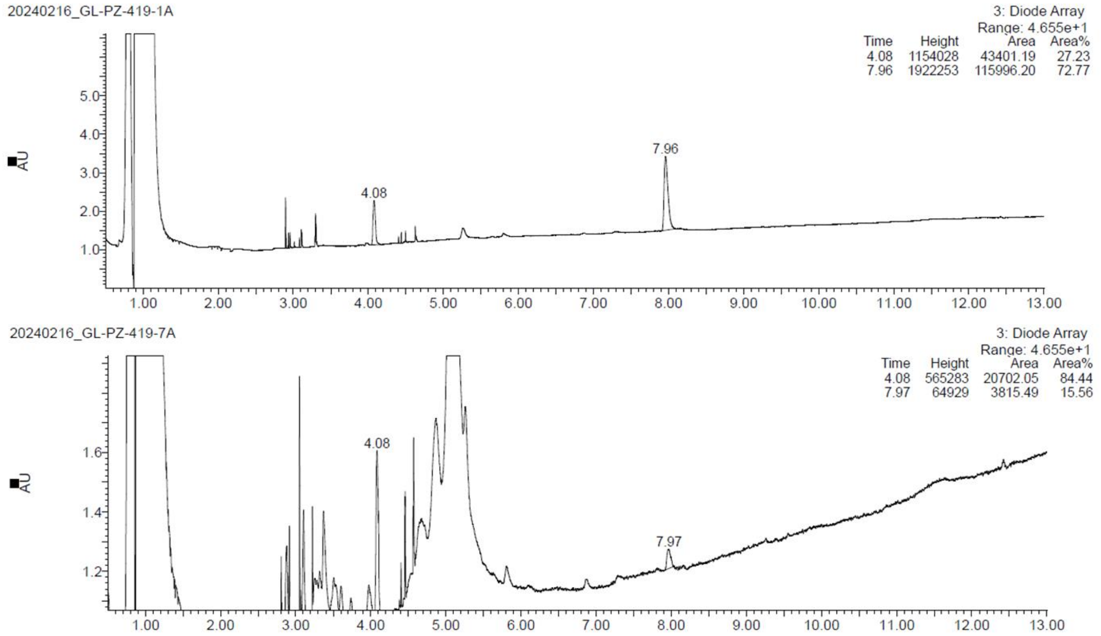

UPLC of **PR 68** solution in PBS - control (above). The amount of **PR 68** remaining after incubation with human plasma proteins (below)
