# Supplementary material for: Low-Basicity 5-HT6 Receptor Ligands from the Group of Cyclic Arylguanidine Derivatives and Their Antiproliferative Activity Evaluation
Source: Int J Mol Sci. 2024 Sep 24;25(19):10287. doi: 10.3390/ijms251910287 (PMC11477289; doi:10.3390/ijms251910287)
Supplement: Supplementary file 1 [file ijms-25-10287-s001.zip › SI_synthesis.pdf]

Supplementary materials - Structural analyses

Table of contents

1-(1-benzothiophene-3-sulfonyl)-1*H*-1,3-benzodiazol-2-amine PR 8 3

1-(quinoline-8-sulfonyl)-1*H*-1,3-benzodiazol-2-amine PR 9 5

N-(1*H*-1,3-benzodiazol-2-yl)naphthalene-1-sulfonamide PR 11 7

N-(3,4-dihydroquinazolin-2-yl)-4-fluorobenzene-1-sulfonamide PR 14 9

N-(3,4-dihydroquinazolin-2-yl)-4-(trifluoromethoxy)benzene-1-sulfonamide PR 17 11

2-chloro-*N*-(3,4-dihydroquinazolin-2-yl)benzene-1-sulfonamide PR 18 14

3-chloro-*N*-(3,4-dihydroquinazolin-2-yl)benzene-1-sulfonamide PR 19 16

2,3-dichloro-*N*-(3,4-dihydroquinazolin-2-yl)benzene-1-sulfonamide PR 20 18

N-(3,4-dihydroquinazolin-2-yl)-3,4-dichlorobenzene-1-sulfonamide PR 21 19

2,6-dichloro-*N*-(3,4-dihydroquinazolin-2-yl)benzene-1-sulfonamide PR 22 22

3,5-dichloro-*N*-(3,4-dihydroquinazolin-2-yl)benzene-1-sulfonamide PR 23 24

N-(3,4-dihydroquinazolin-2-yl)-3-methoxybenzene-1-sulfonamide PR 24 26

5-chloro-*N*-(3,4-dihydroquinazolin-2-yl)-2-methoxybenzene-1-sulfonamide PR 25 28

5-chloro-*N*-(3,4-dihydroquinazolin-2-yl)naphthalene-2-sulfonamide PR 30 30

N-(5-chloro-3,4-dihydroquinazolin-2-yl)naphthalene-2-sulfonamide PR 31 32

N-(7-chloro-3,4-dihydroquinazolin-2-yl)naphthalene-2-sulfonamide PR 33 35

N-(7-chloro-4-methyl-3,4-dihydroquinazolin-2-yl)naphthalene-2-sulfonamide PR 37 37

N-(4,5-dihydro-1*H*-1,3-benzodiazepin-2-yl)naphthalene-2-sulfonamide PR 38 39

2-chloro-*N*-(3,4-dihydroquinazolin-2-yl)naphthalene-1-sulfonamide PR 41 41

4-chloro-*N*-(3,4-dihydroquinazolin-2-yl)naphthalene-1-sulfonamide PR 42 43

5-chloro-*N*-(3,4-dihydroquinazolin-2-yl)naphthalene-1-sulfonamide PR 43 45

6-chloro-*N*-(3,4-dihydroquinazolin-2-yl)naphthalene-1-sulfonamide PR 44 47

8-chloro-*N*-(3,4-dihydroquinazolin-2-yl)naphthalene-1-sulfonamide PR 45 49

N-(5-chloro-3,4-dihydroquinazolin-2-yl)naphthalene-1-sulfonamide PR 46 51

N-(6-chloro-3,4-dihydroquinazolin-2-yl)naphthalene-1-sulfonamide PR 47 53

N-(6-chloro-3,4-dihydroquinazolin-2-yl)naphthalene-1-sulfonamide PR 48 55

N-(8-chloro-3,4-dihydroquinazolin-2-yl)naphthalene-1-sulfonamide PR 49 57

N-(5,6-dichloro-3,4-dihydroquinazolin-2-yl)naphthalene-1-sulfonamide PR 50 59

N-(6,8-dichloro-3,4-dihydroquinazolin-2-yl)naphthalene-1-sulfonamide PR 51 60

2-chloro-*N*-(5-chloro-3,4-dihydroquinazolin-2-yl)naphthalene-1-sulfonamide PR 52 62

4-chloro-*N*-(5-chloro-3,4-dihydroquinazolin-2-yl)naphthalene-1-sulfonamide PR 53 64

N-(5-fluoro-3,4-dihydroquinazolin-2-yl)naphthalene-1-sulfonamide PR 54 66

N-(6-fluoro-3,4-dihydroquinazolin-2-yl)naphthalene-1-sulfonamide PR 55 68

N-(7-fluoro-3,4-dihydroquinazolin-2-yl)naphthalene-1-sulfonamide PR 56 70
N-[6-(trifluoromethyl)-3,4-dihydroquinazolin-2-yl]naphthalene-1-sulfonamide PR 57 72
N-(5-methoxy-3,4-dihydroquinazolin-2-yl)naphthalene-1-sulfonamide PR 58 74
N-(6-methoxy-3,4-dihydroquinazolin-2-yl)naphthalene-1-sulfonamide PR 59 76
N-(7-methoxy-3,4-dihydroquinazolin-2-yl)naphthalene-1-sulfonamide PR 60 78
N-(5-methyl-3,4-dihydroquinazolin-2-yl)naphthalene-1-sulfonamide PR 61 80
N-(7-methyl-3,4-dihydroquinazolin-2-yl)naphthalene-1-sulfonamide PR 62 82
N-[6-(dimethylamino)-3,4-dihydroquinazolin-2-yl]naphthalene-1-sulfonamide PR 64........................ 84
N-[6-(morpholin-4-yl)-3,4-dihydroquinazolin-2-yl]naphthalene-1-sulfonamide PR 65 86
N-(4-methyl-3,4-dihydroquinazolin-2-yl)naphthalene-1-sulfonamide PR 67 88
N-(5-chloro-4-methyl-3,4-dihydroquinazolin-2-yl)naphthalene-1-sulfonamide PR 68 90
N-(6-chloro-4-methyl-3,4-dihydroquinazolin-2-yl)naphthalene-1-sulfonamide PR 69 92
N-(7-chloro-4-methyl-3,4-dihydroquinazolin-2-yl)naphthalene-1-sulfonamide PR 70 94
N-(8-chloro-4-methyl-3,4-dihydroquinazolin-2-yl)naphthalene-1-sulfonamide PR 71 96
N-(6,8-dichloro-4-methyl-3,4-dihydroquinazolin-2-yl)naphthalene-1-sulfonamide PR72 98
N-(5-fluoro-4-methyl-3,4-dihydroquinazolin-2-yl)naphthalene-1-sulfonamide PR 73 100
4-chloro-*N*-(6,8-dichloro-4-methyl-3,4-dihydroquinazolin-2-yl)naphthalene-1-sulfonamide PR 74.. 102
N-(6-bromo-4-methyl-3,4-dihydroquinazolin-2-yl)naphthalene-1-sulfonamide PR 75...................... 104
N-(3,4-dimethyl-3,4-dihydroquinazolin-2-yl)naphthalene-1-sulfonamide PR 76 106
N-(4-methyl-4H-3,1-benzoxazin-2-yl)naphthalene-1-sulfonamide PR 77.. 108
N-(4,5-dihydro-3H-1,3-benzodiazepin-2-yl)naphthalene-1-sulfonamide PR 78 110

1-(1-benzothiophene-3-sulfonyl)-1*H*-1,3-benzodiazol-2-amine PR 8

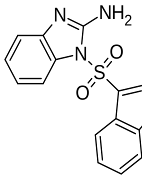

UPLC-MS:

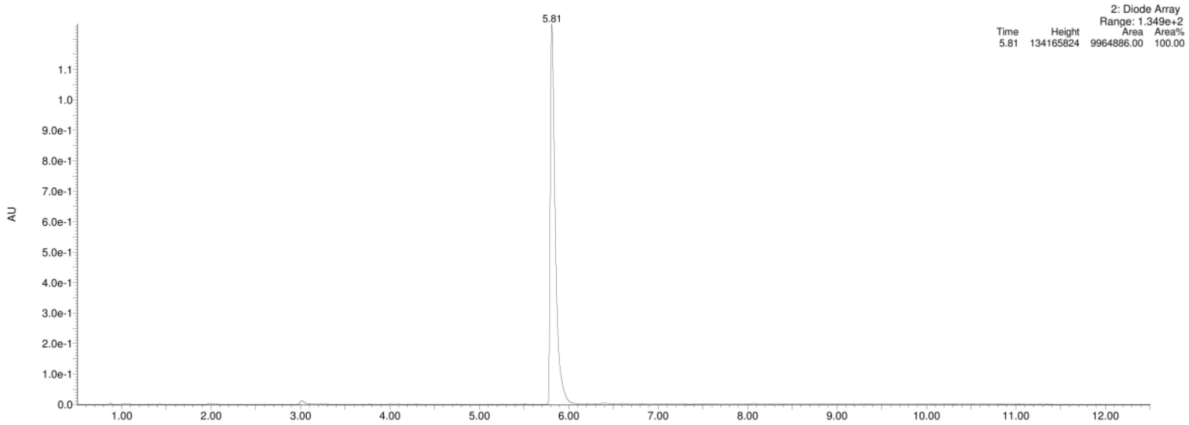

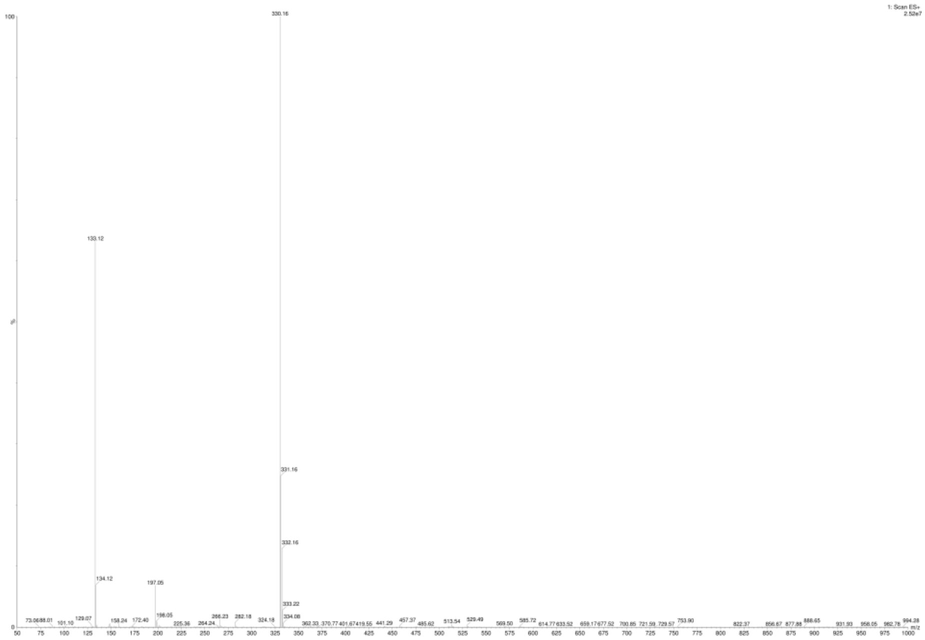

^{1}H NMR:

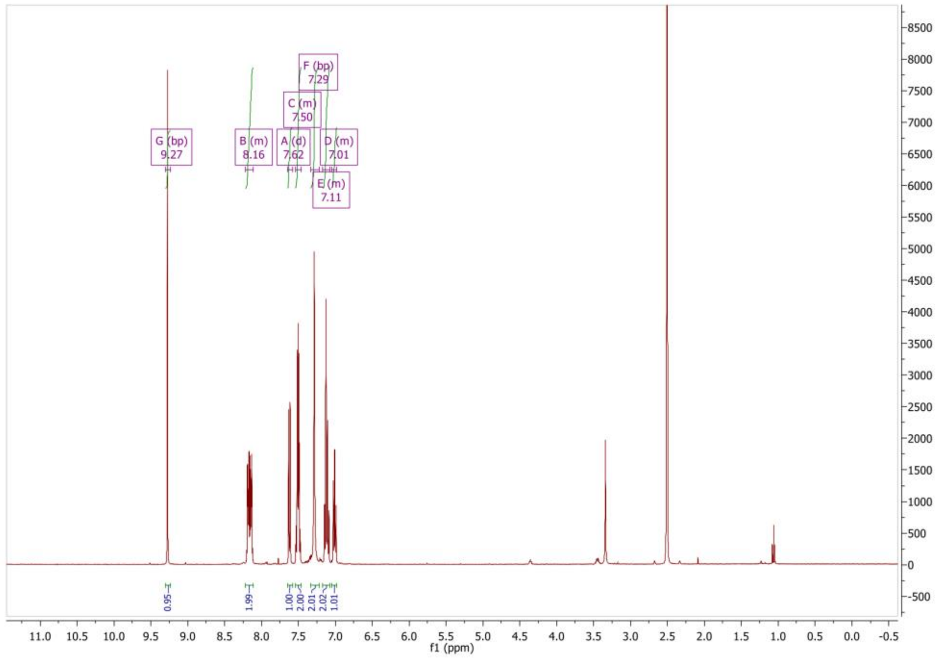

^{13}C NMR:

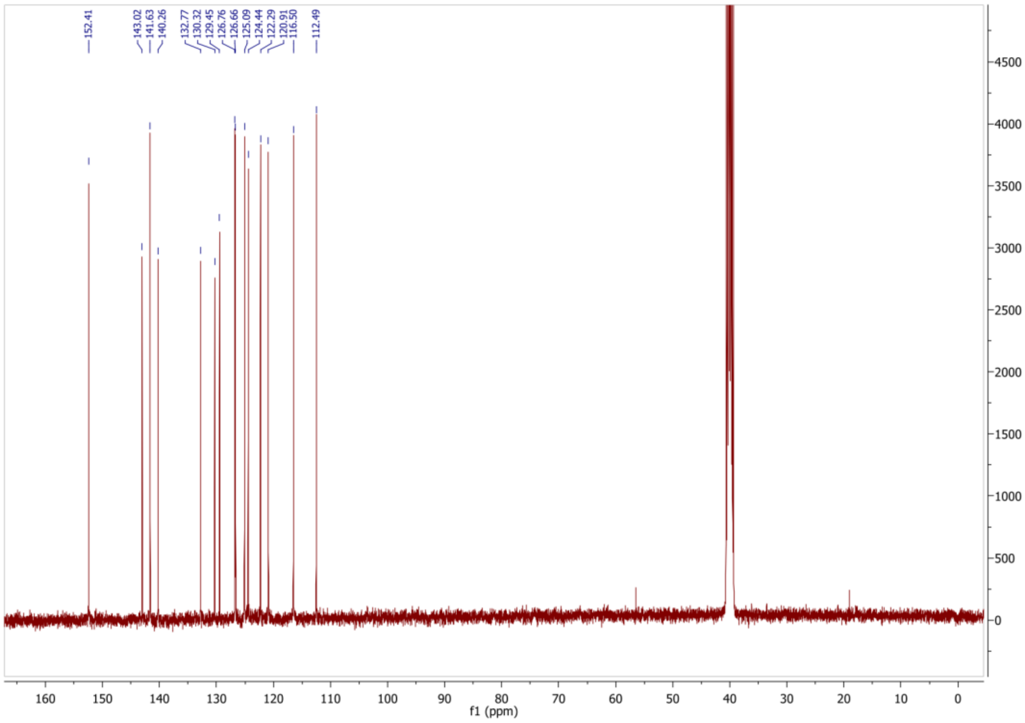

FT-IR:

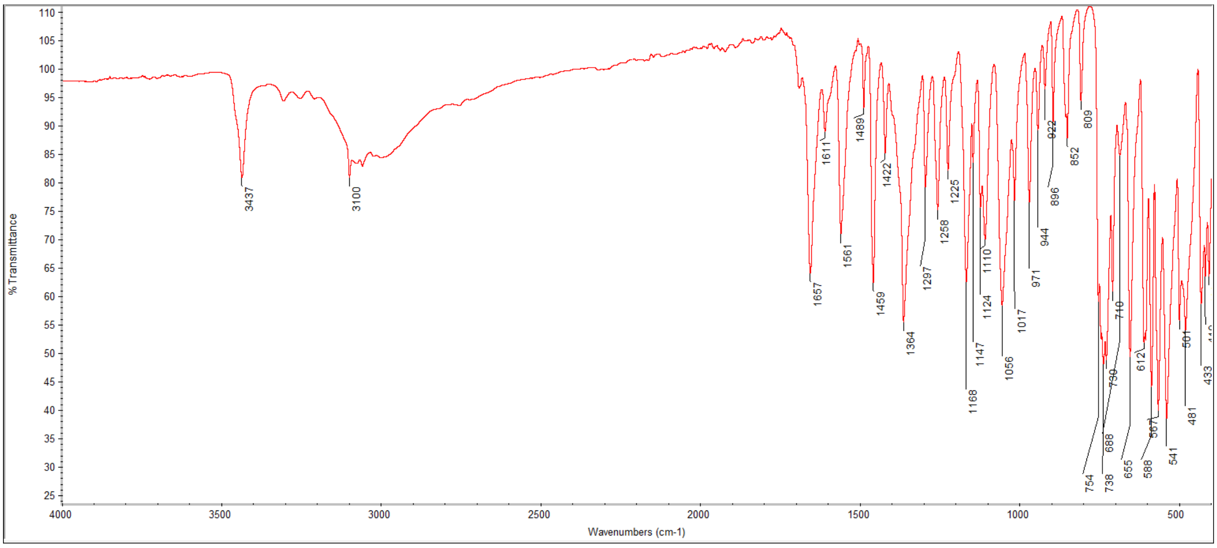

1-(quinoline-8-sulfonyl)-1*H*-1,3-benzodiazol-2-amine PR 9

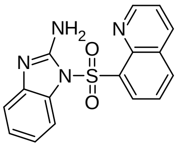

UPLC-MS:

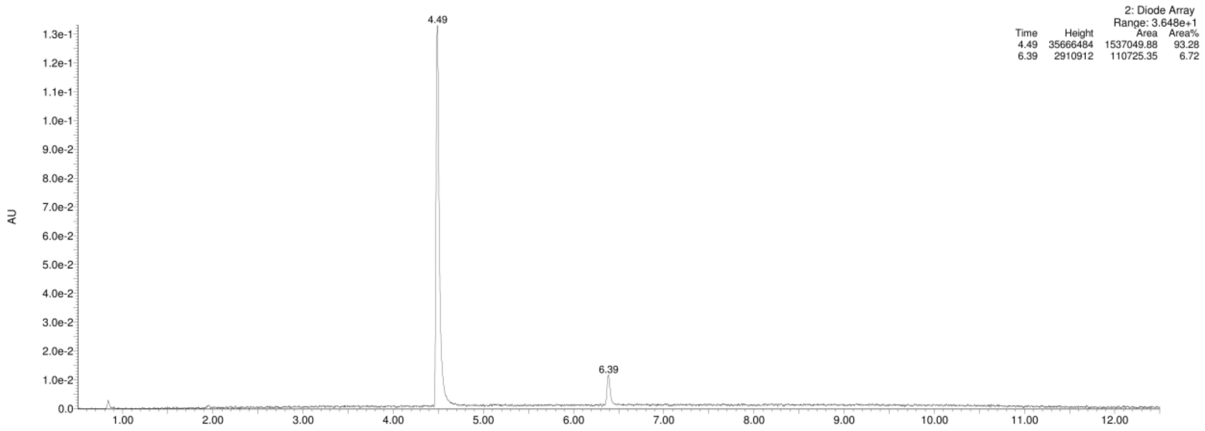

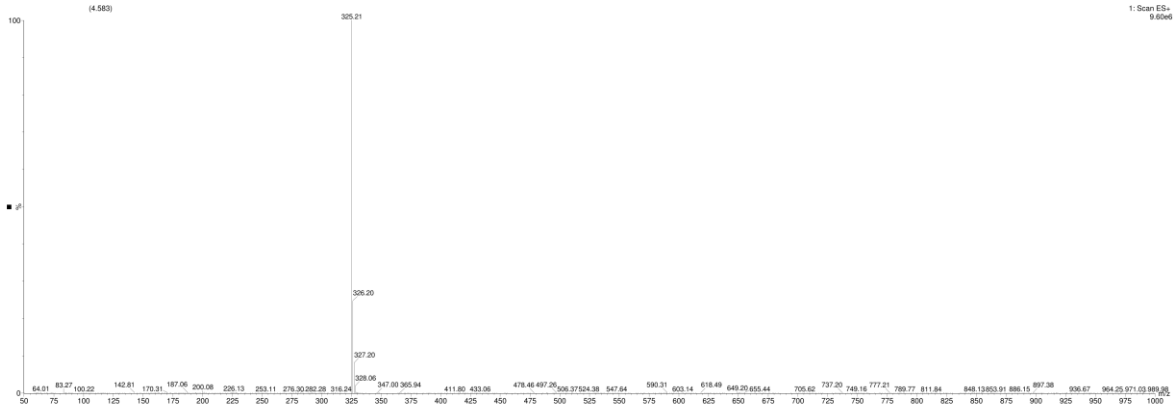

^{1}H NMR:

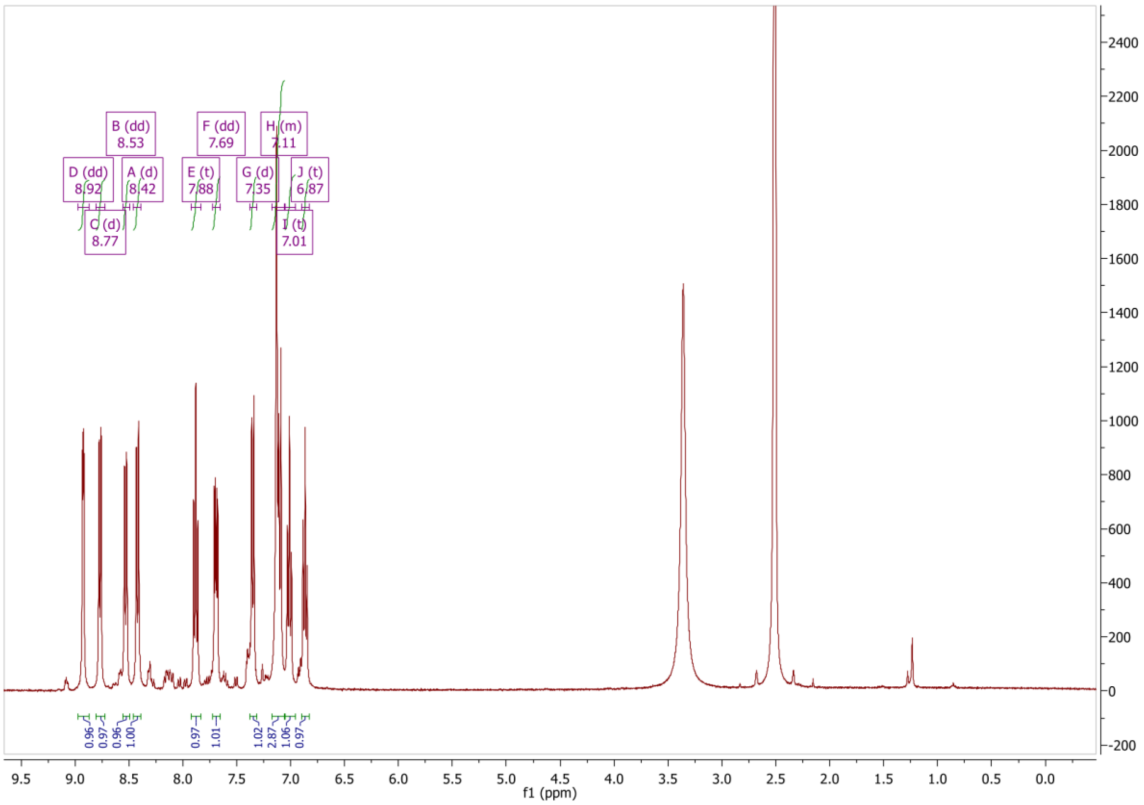

^{13}C NMR:

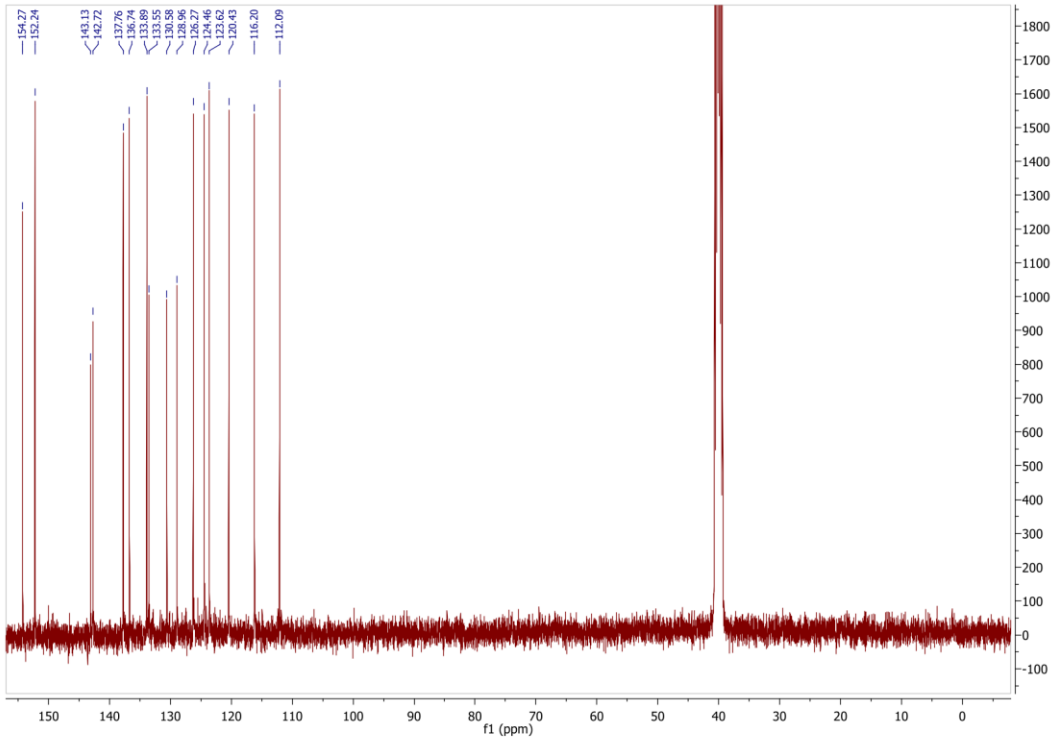

FT-IR:

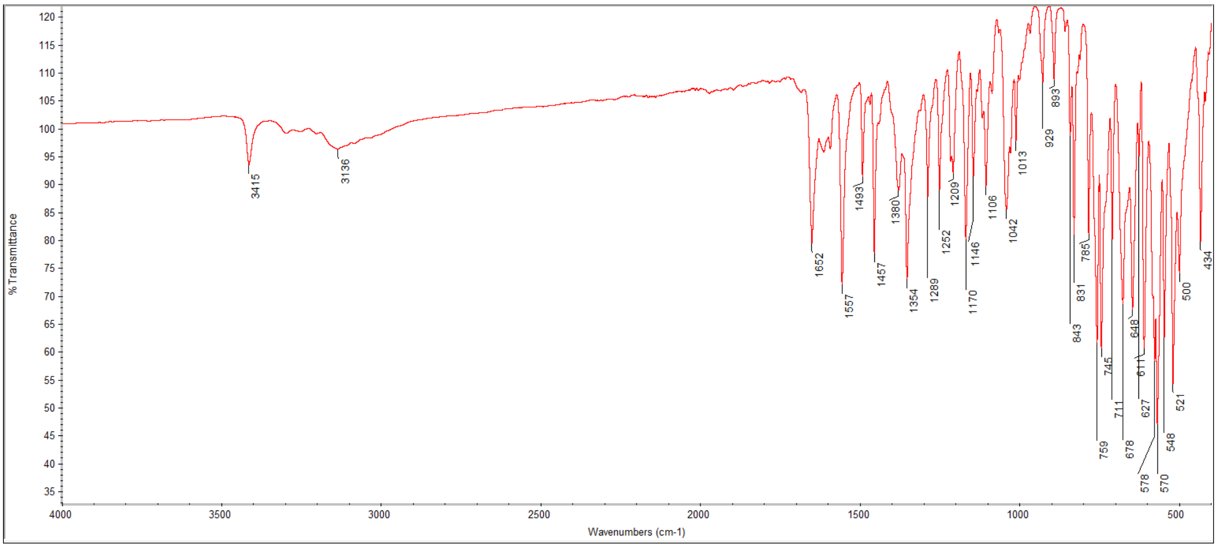

N-(1*H*-1,3-benzodiazol-2-yl)naphthalene-1-sulfonamide PR 11

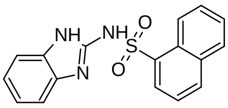

UPLC-MS:

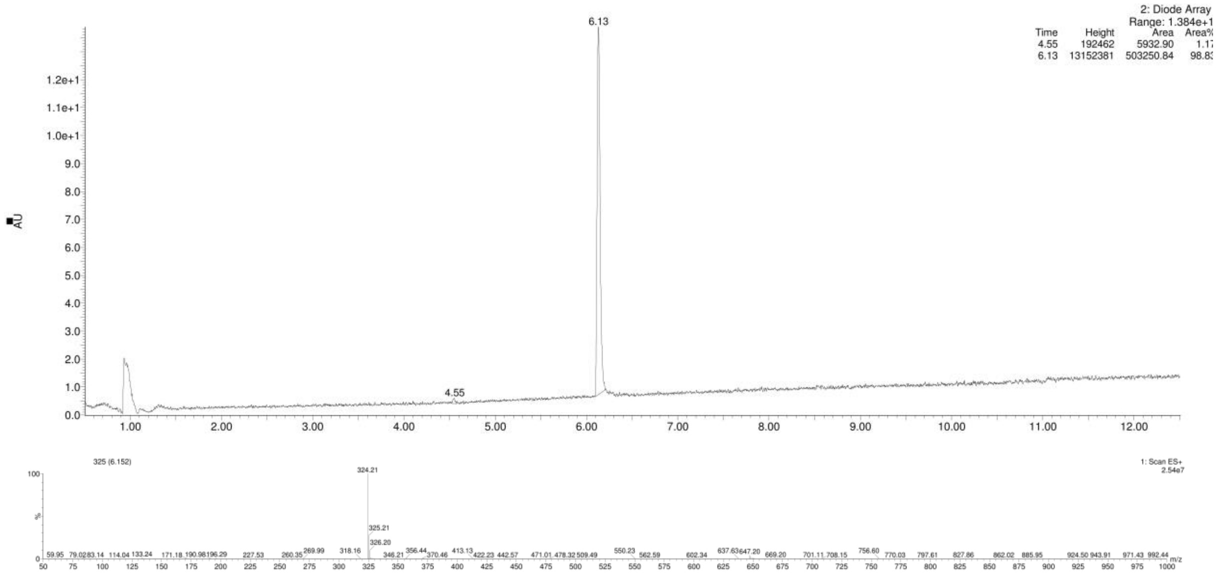

^{1}H NMR:

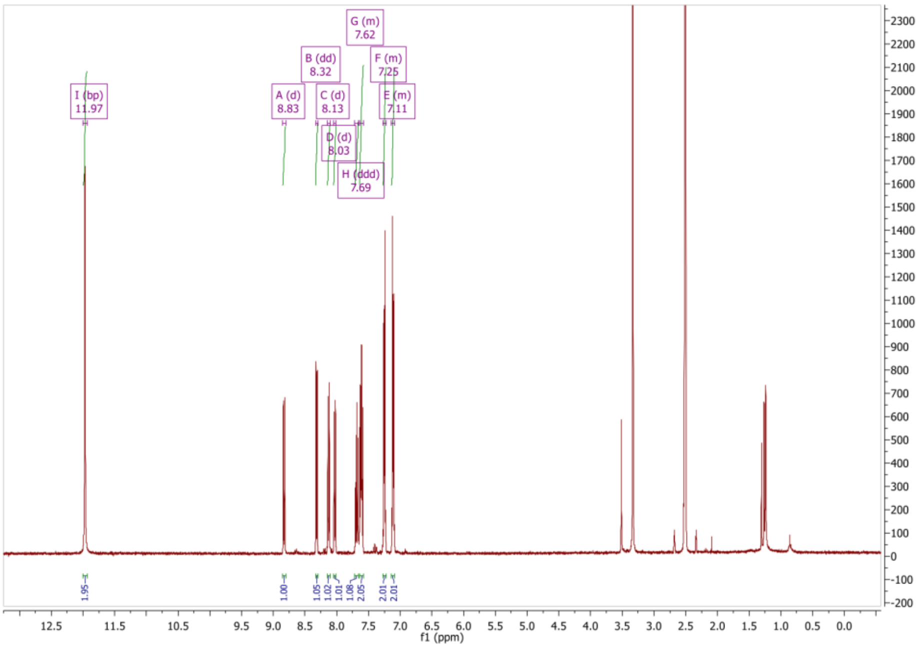

^{13}C NMR:

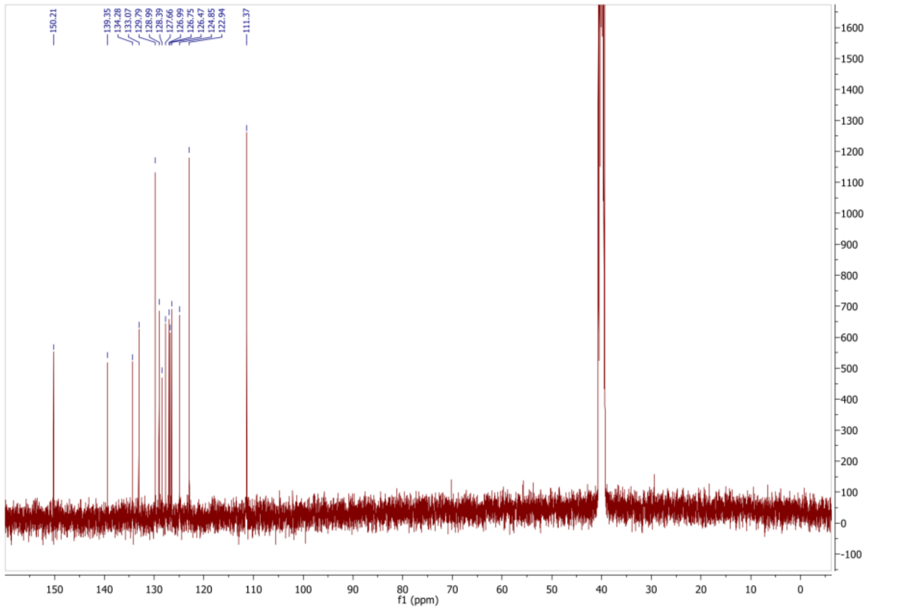

FT-IR:

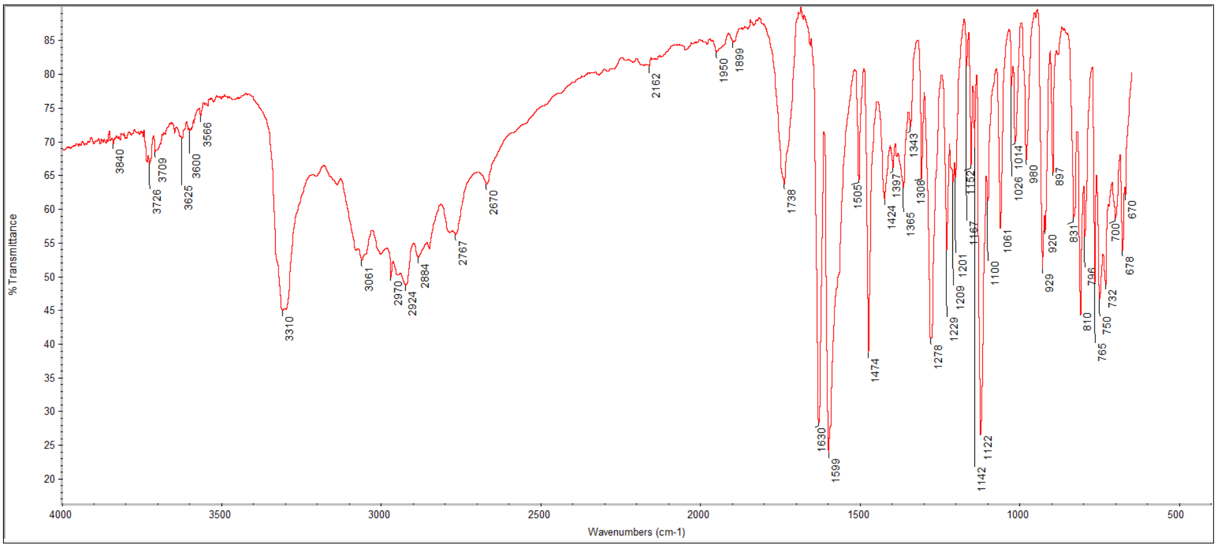

N-(3,4-dihydroquinazolin-2-yl)-4-fluorobenzene-1-sulfonamide PR 14

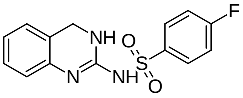

UPLC-MS:

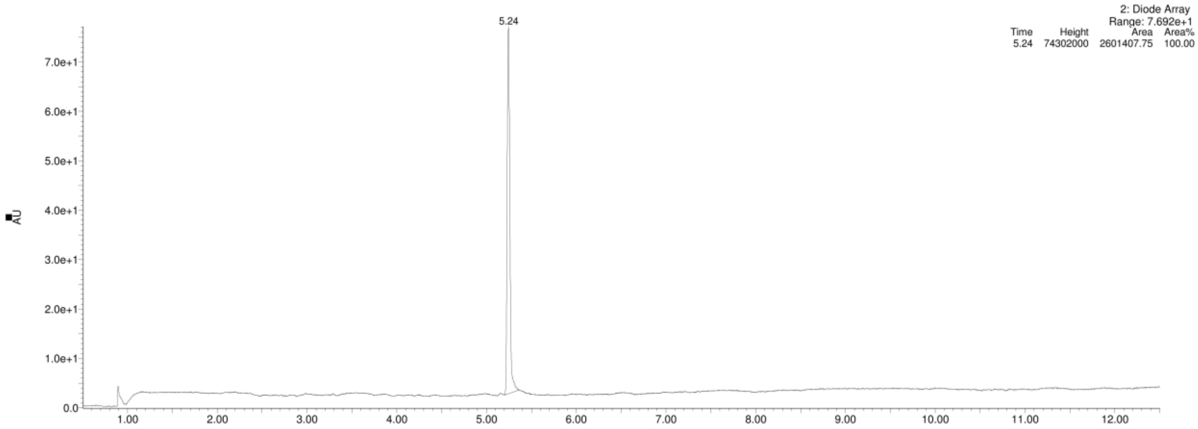

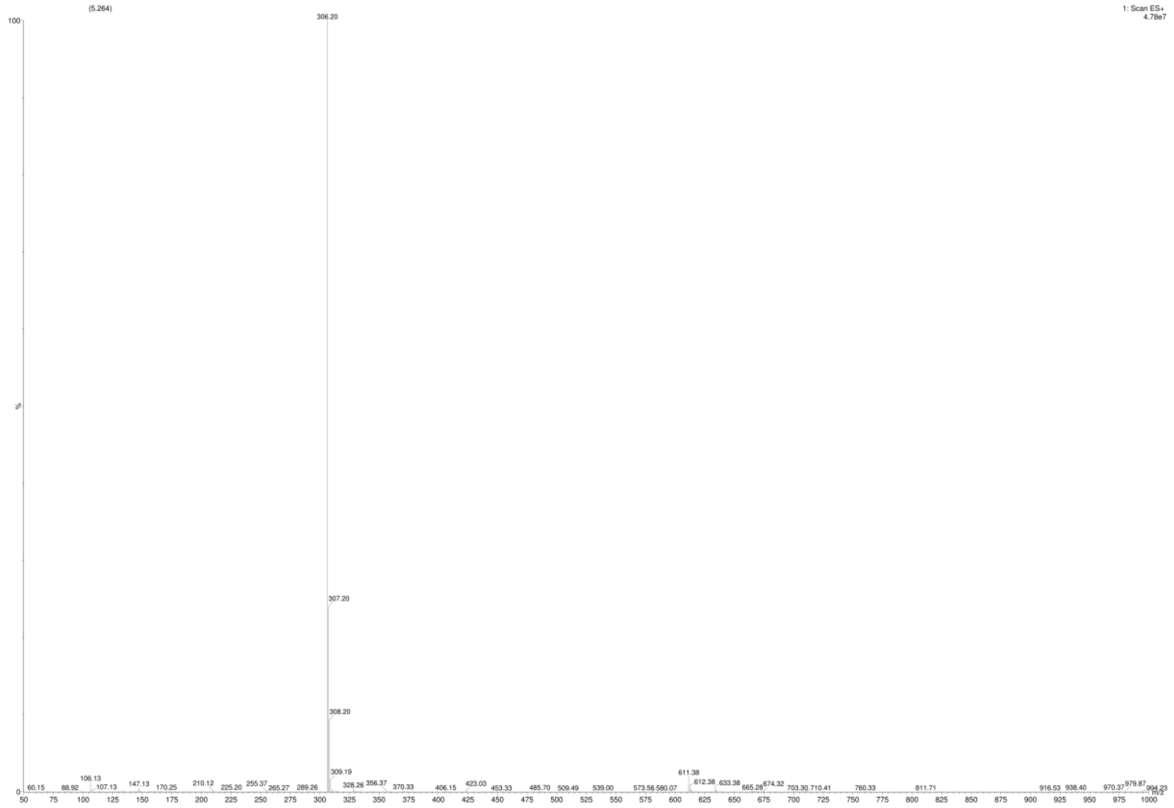
(5.264)
306.20
1: Scan ES+
4.78e7
307.20
308.20
611.38
m/z

^{1}H NMR:

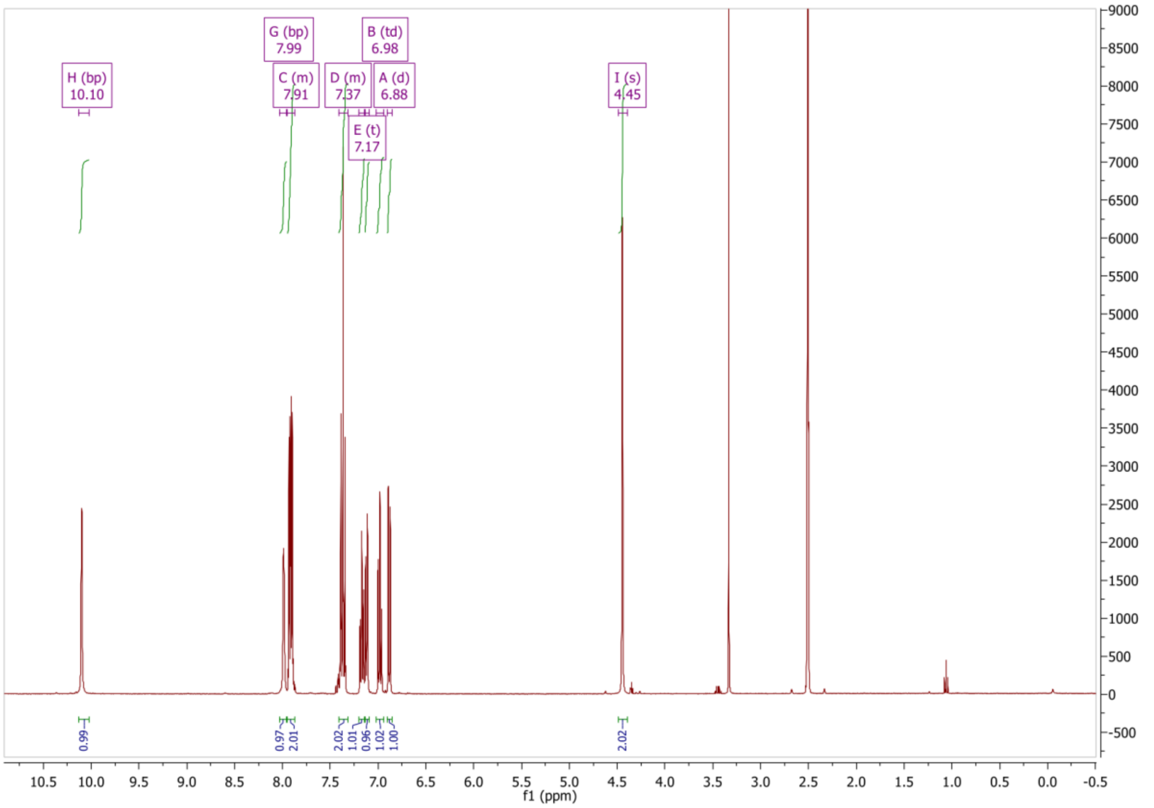
H (bp)
10.10
G (bp)
7.99
C (m)
7.91
D (m)
7.37
E (t)
7.17
B (td)
6.98
A (d)
6.88
I (s)
4.45
f1 (ppm)

^{13}C NMR:

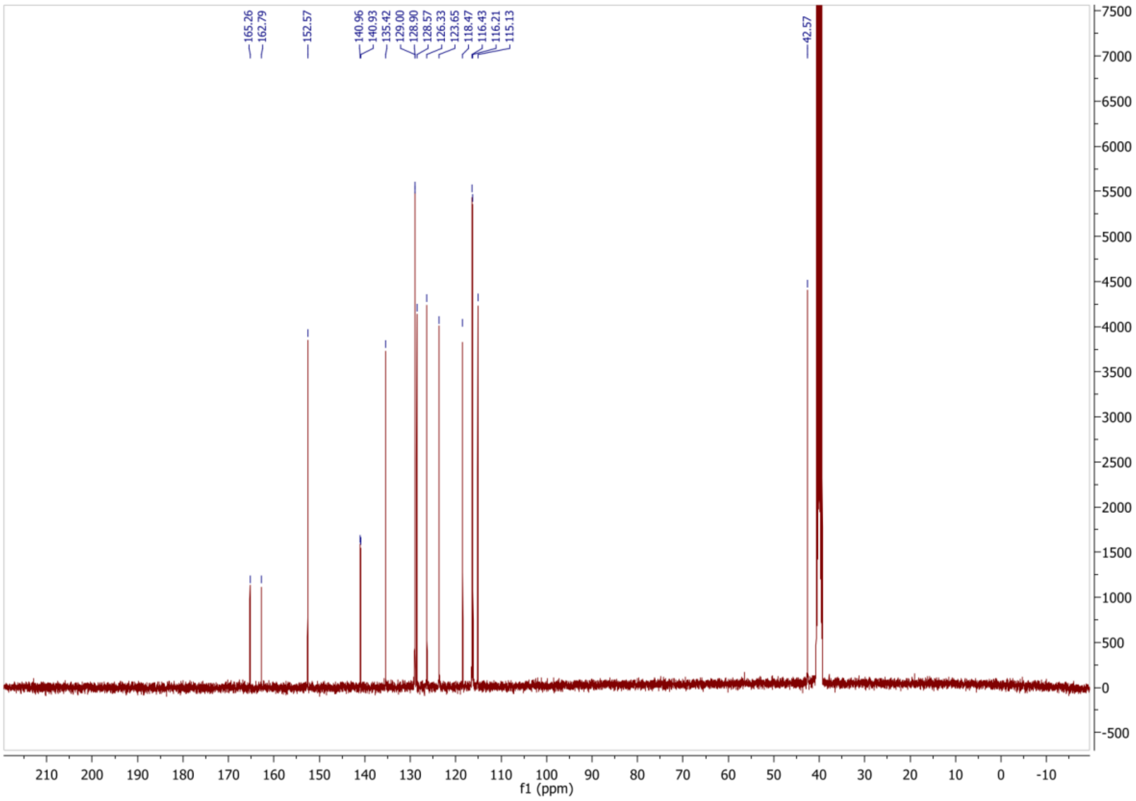

FT-IR:

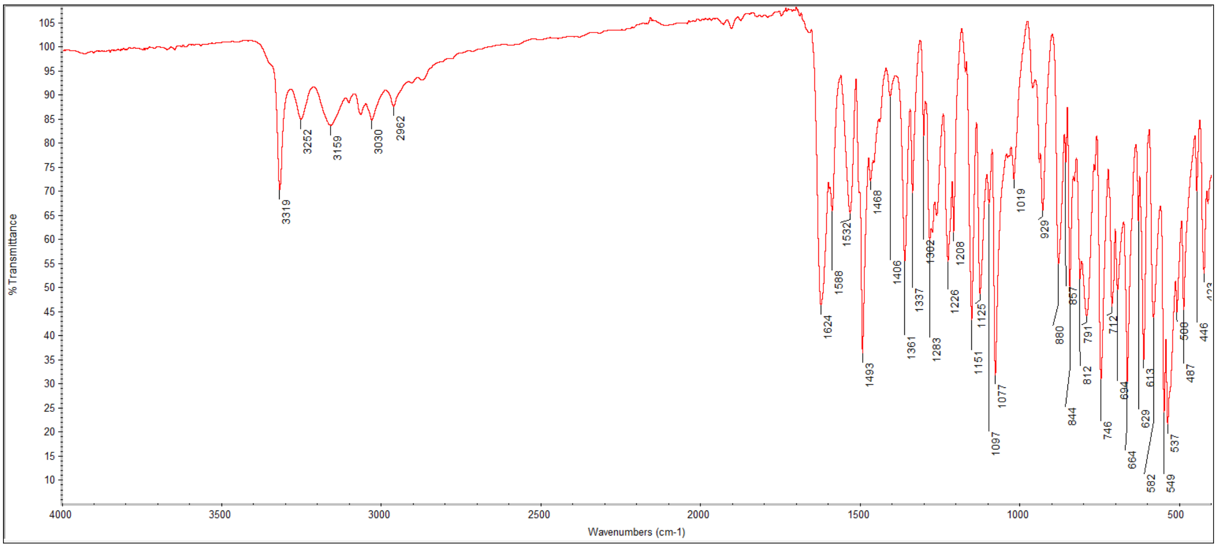

N-(3,4-dihydroquinazolin-2-yl)-4-(trifluoromethoxy)benzene-1-sulfonamide PR 17

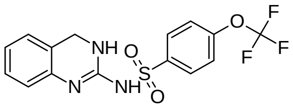

UPLC-MS:

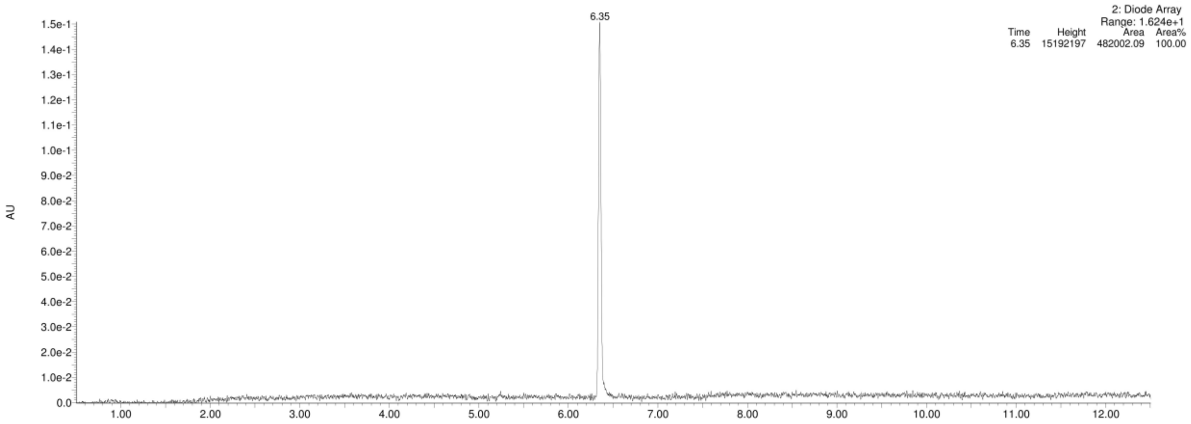
6.35
2: Diode Array
Range: 1.624e+1
Time Height Area Area%
6.35 15192197 482002.09 100.00
AU

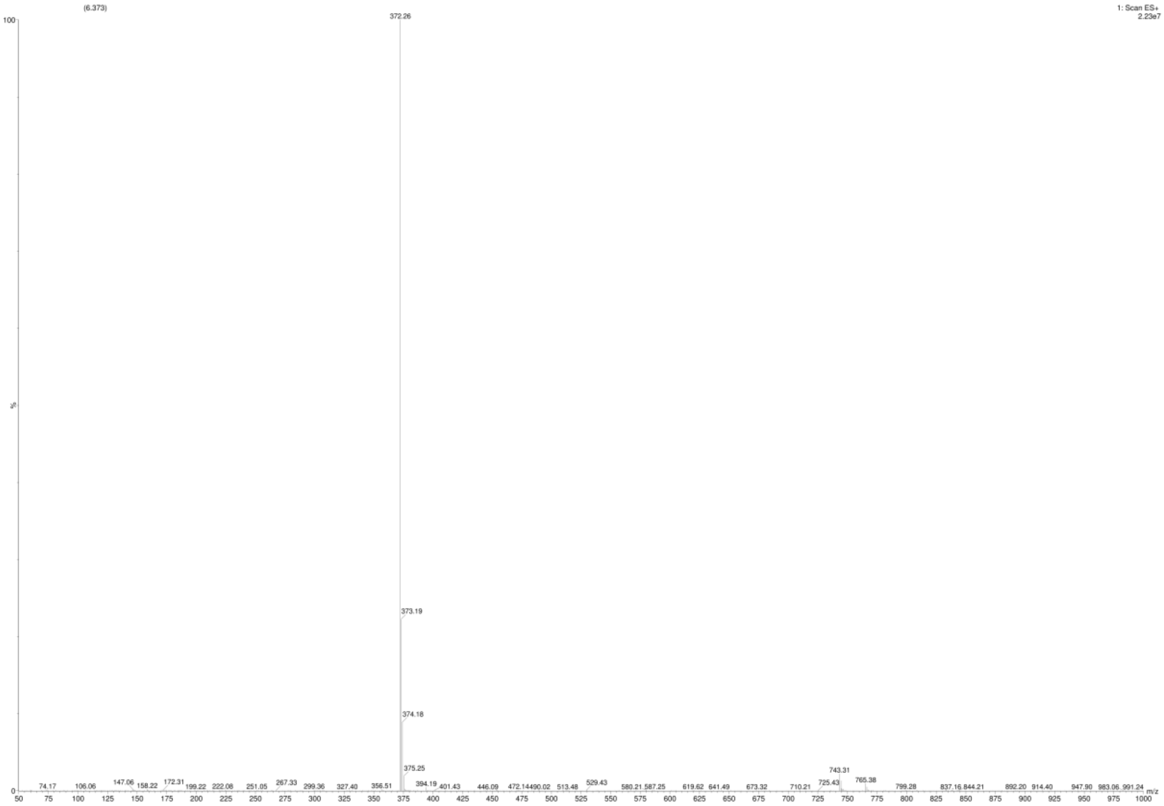
(6.373)
372.26
1: Scan ES+
2.23e7
373.19
374.18
375.25
743.31
765.38
m/z

^{1}H NMR:

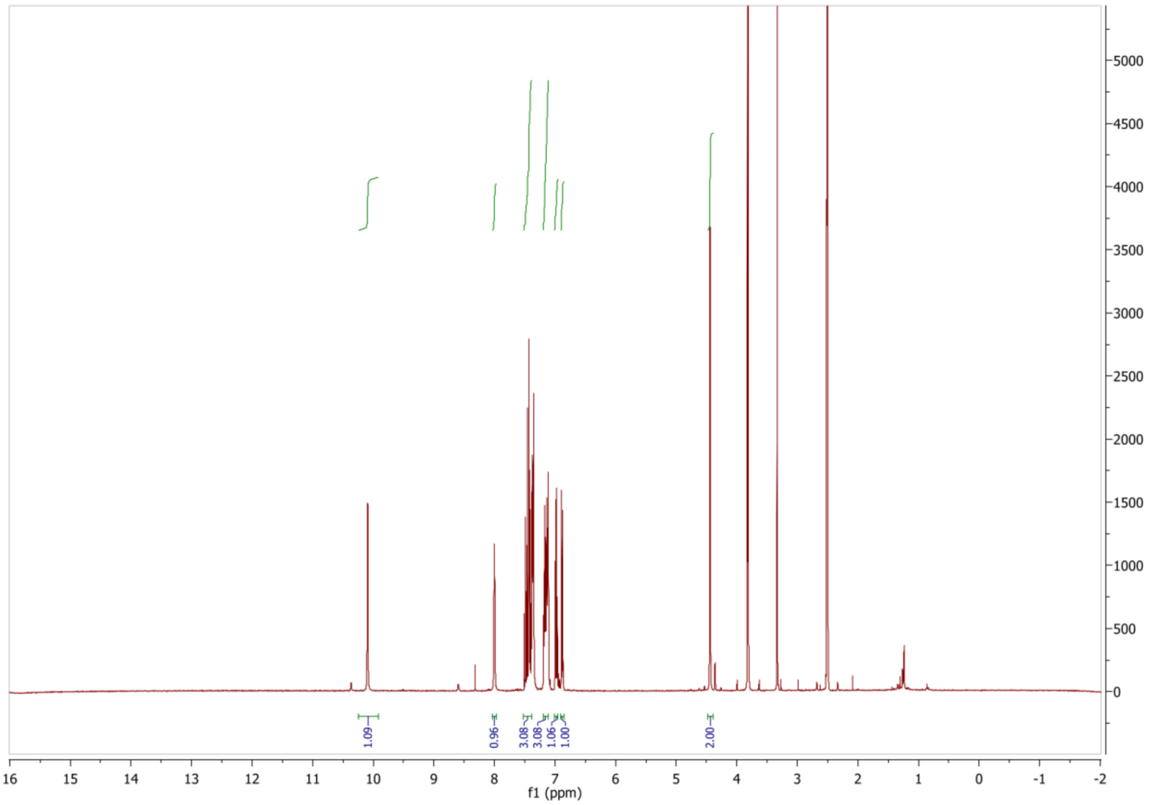

^{13}C NMR:

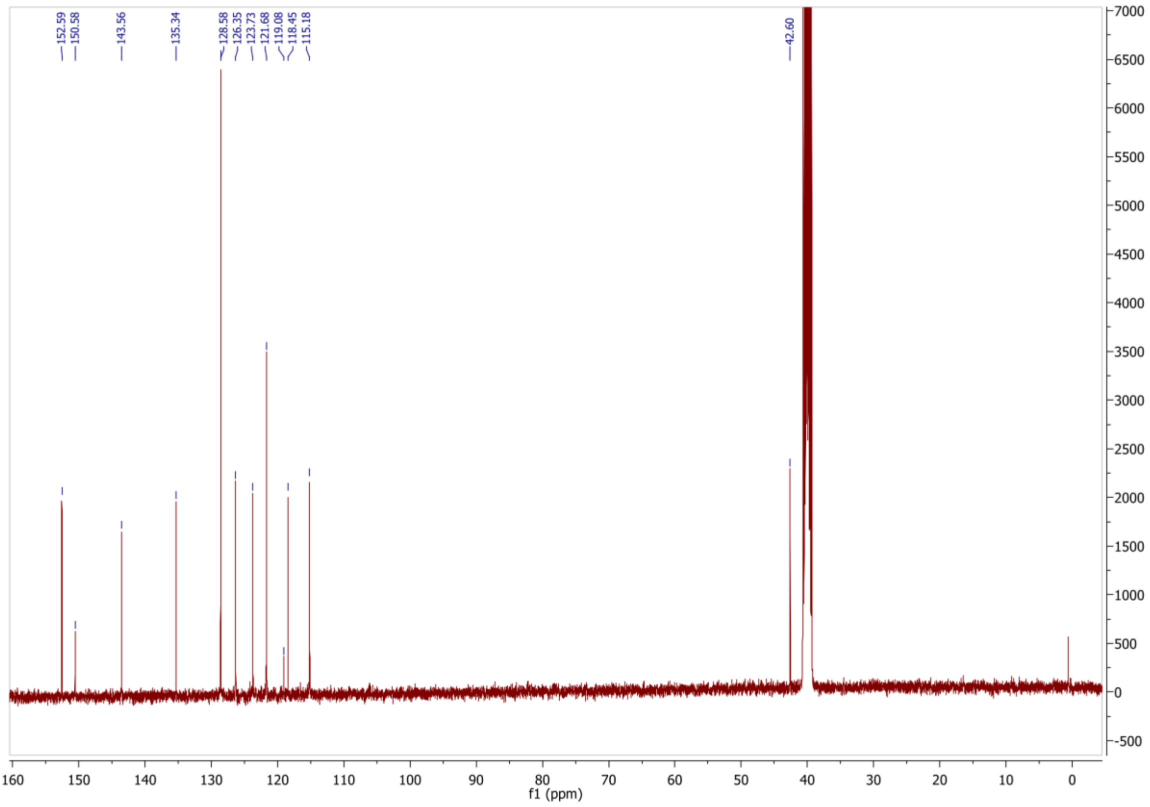

FT-IR:

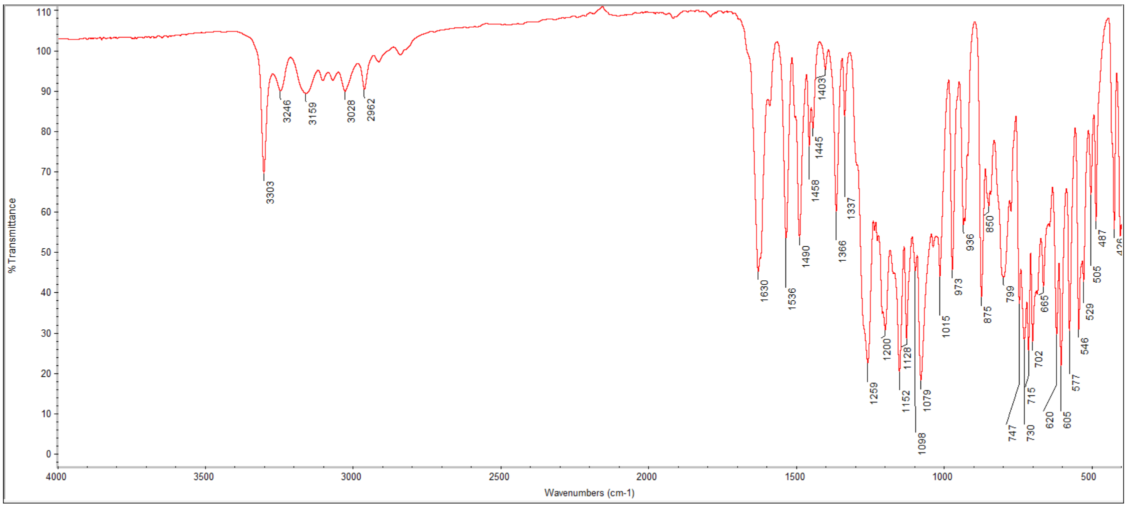

2-chloro-*N*-(3,4-dihydroquinazolin-2-yl)benzene-1-sulfonamide PR 18

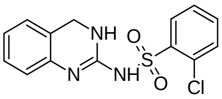

UPLC-MS:

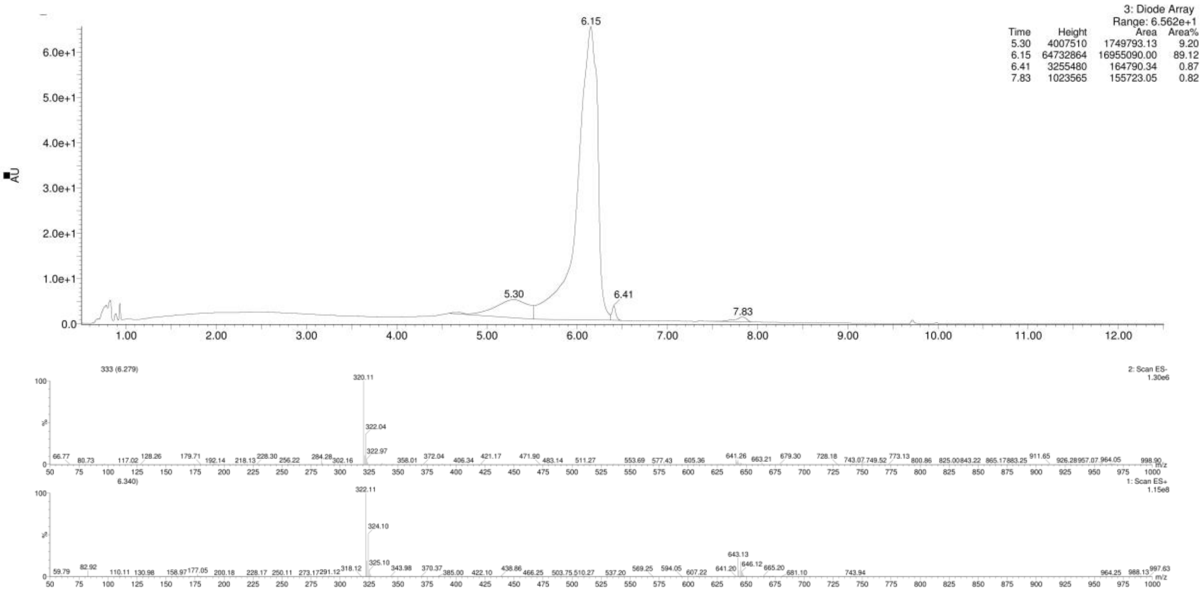

^{1}H NMR:

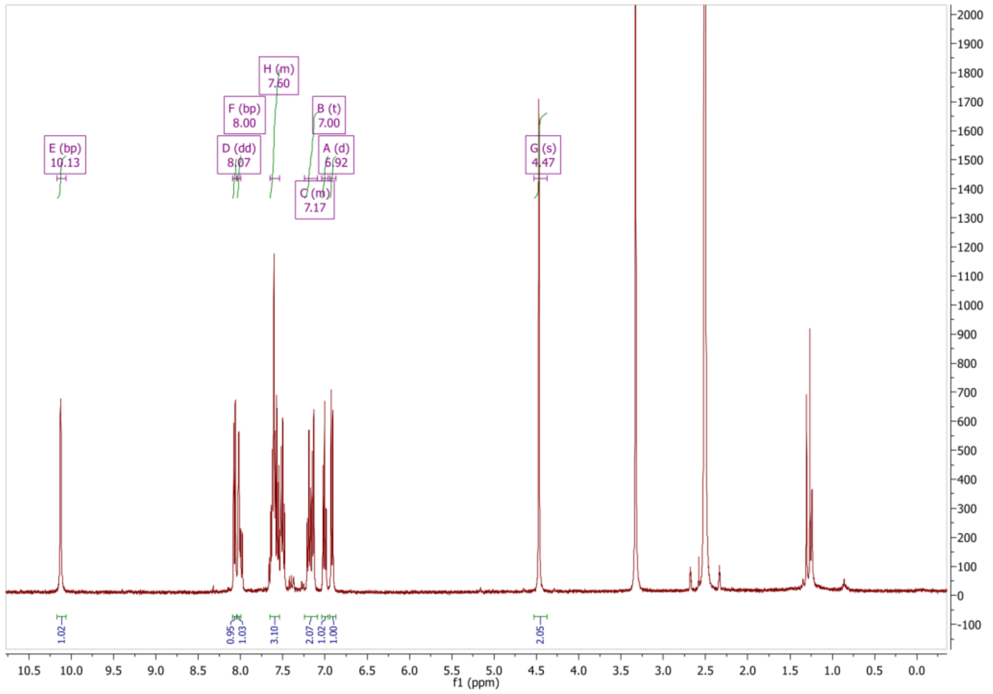

^{13}C NMR:

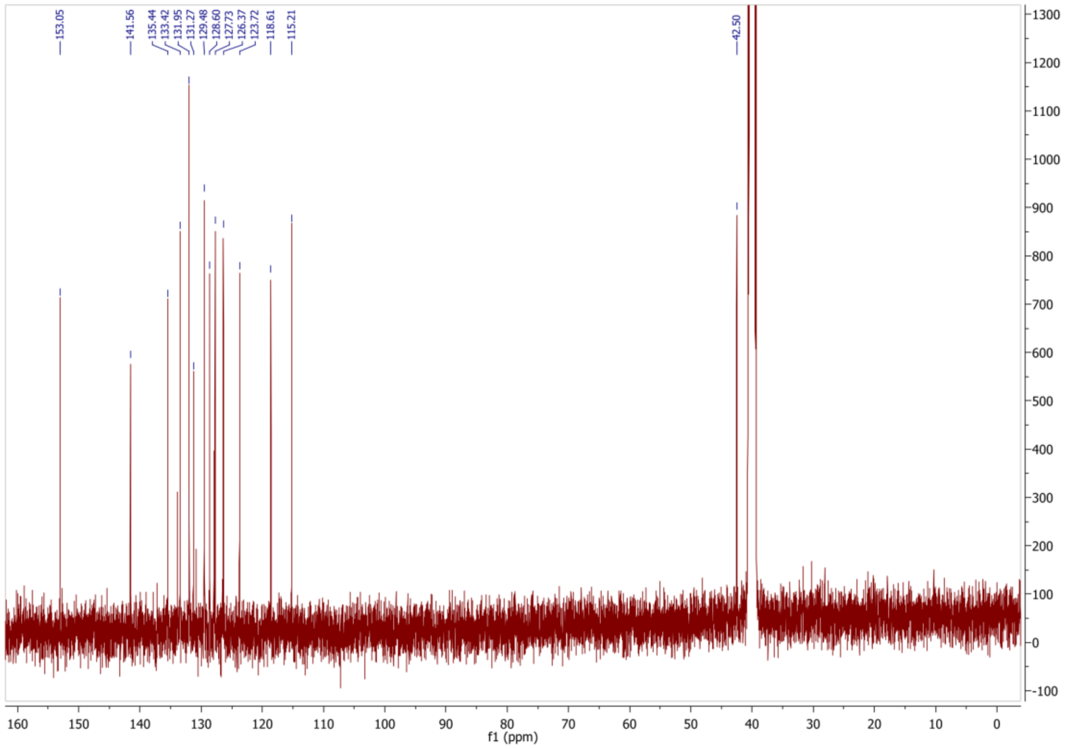

FT-IR:

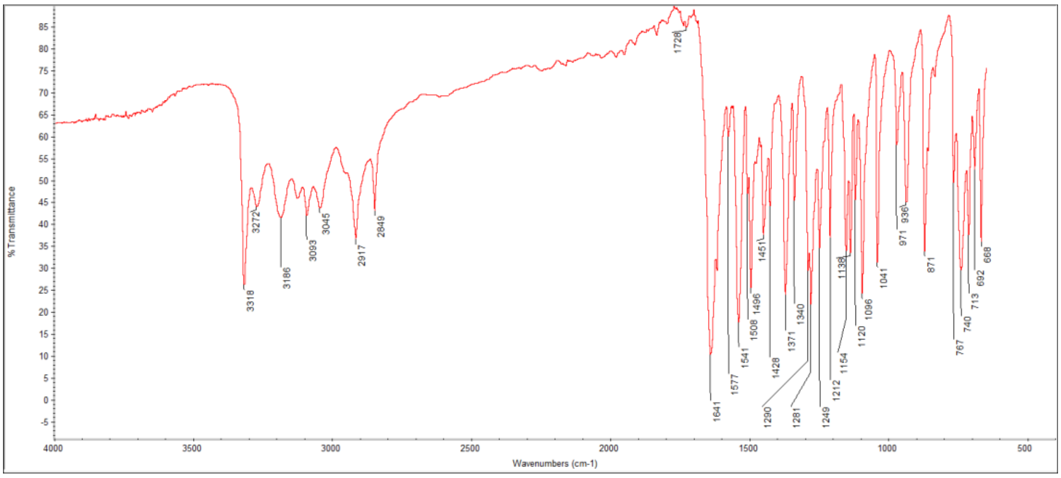

3-chloro-*N*-(3,4-dihydroquinazolin-2-yl)benzene-1-sulfonamide PR 19

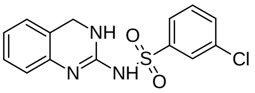

UPLC-MS:

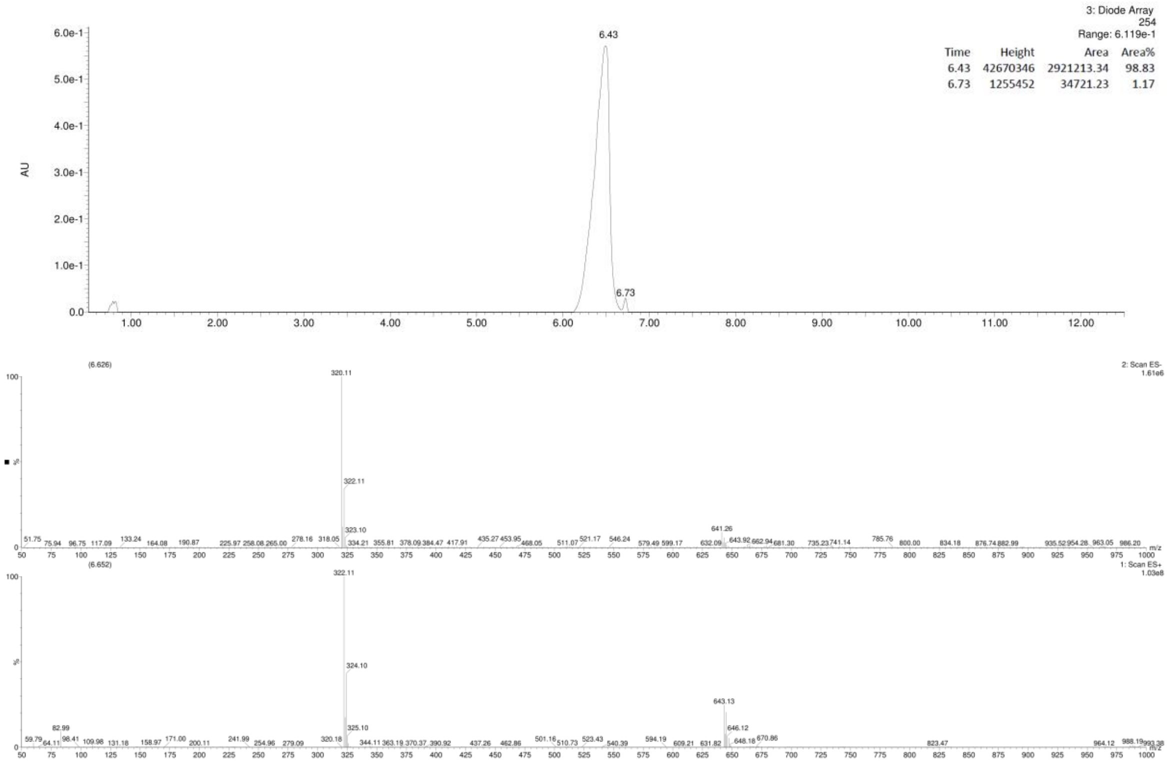

^{1}H NMR:

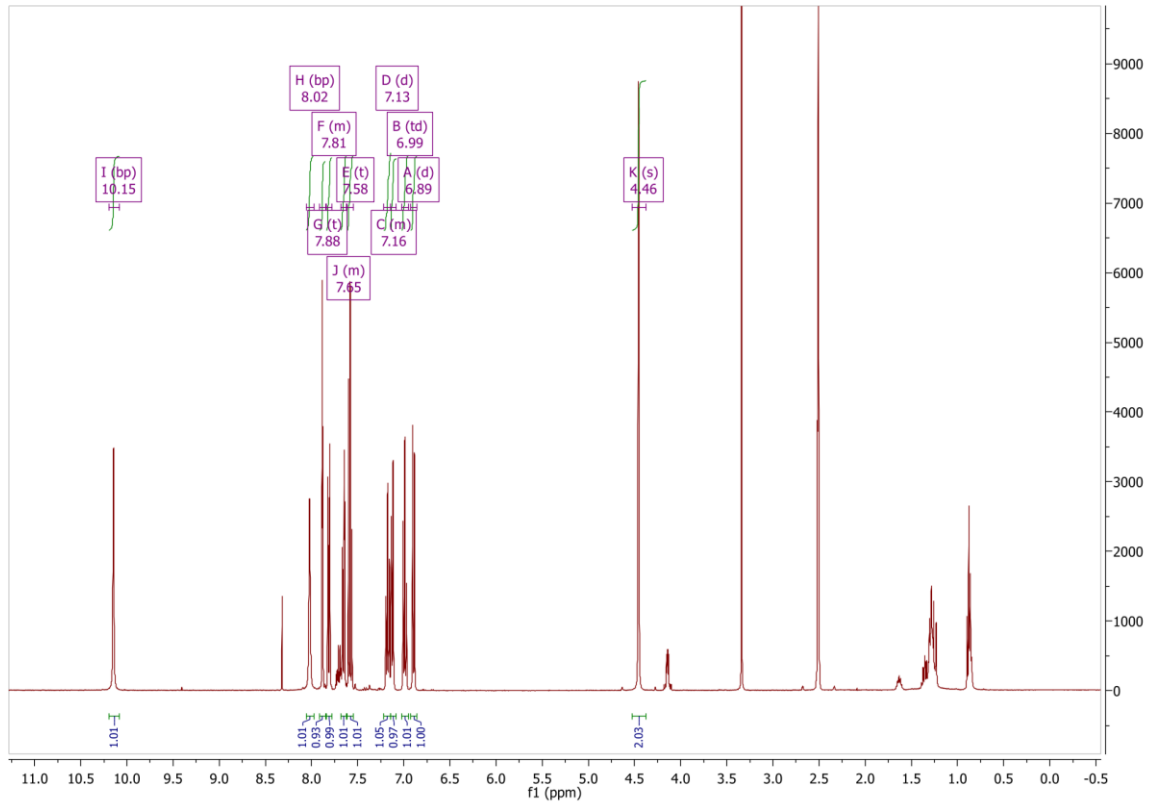

^{13}C NMR:

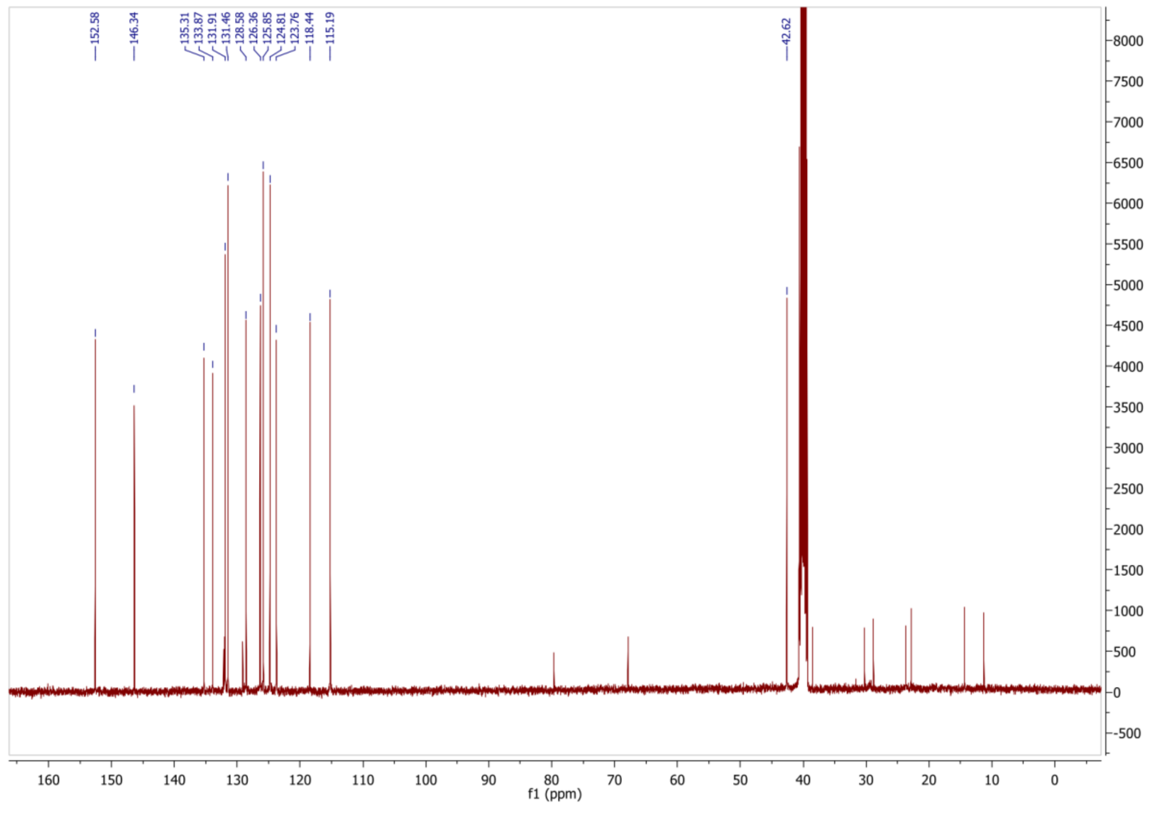

FT-IR:

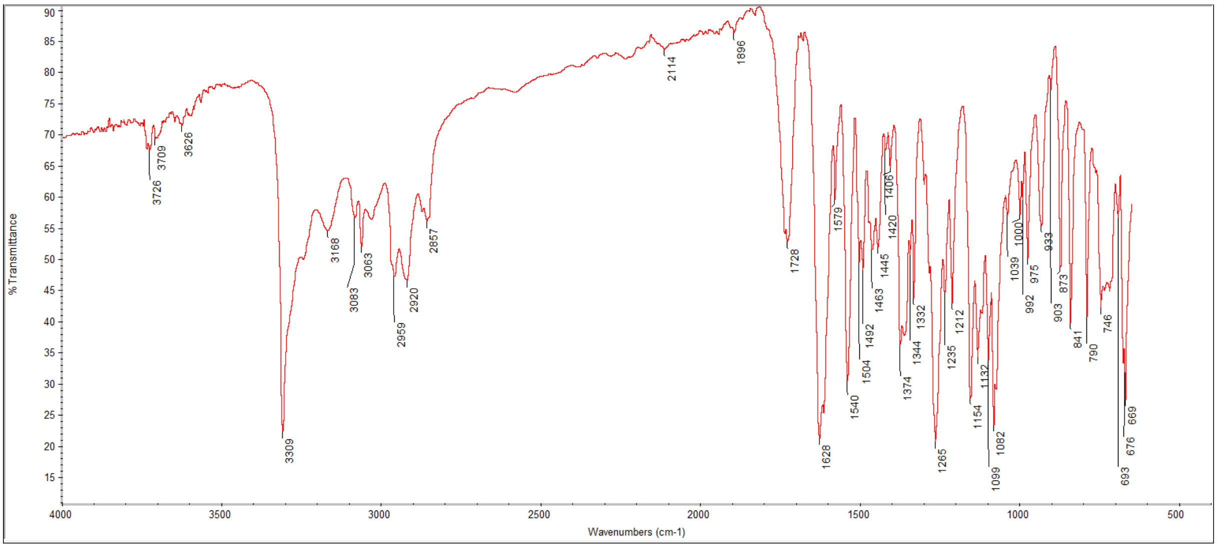

2,3-dichloro-*N*-(3,4-dihydroquinazolin-2-yl)benzene-1-sulfonamide PR 20

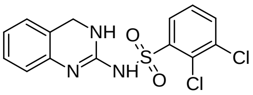

UPLC-MS:

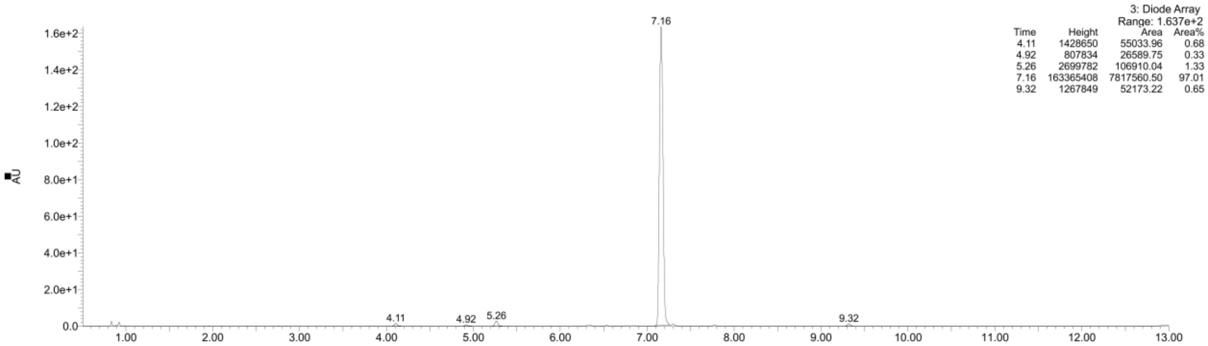

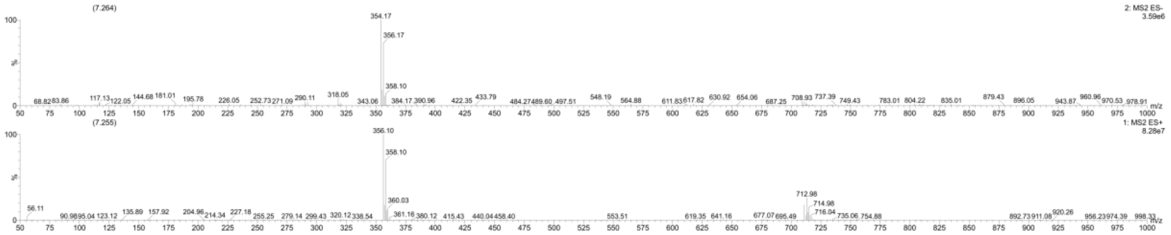

^{1}H NMR:

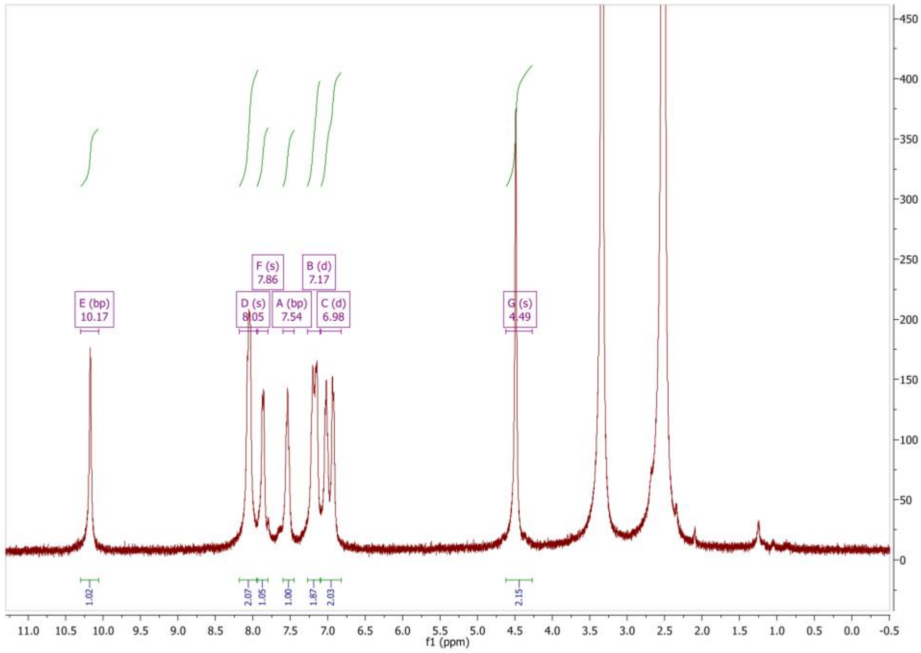

^{13}C NMR:

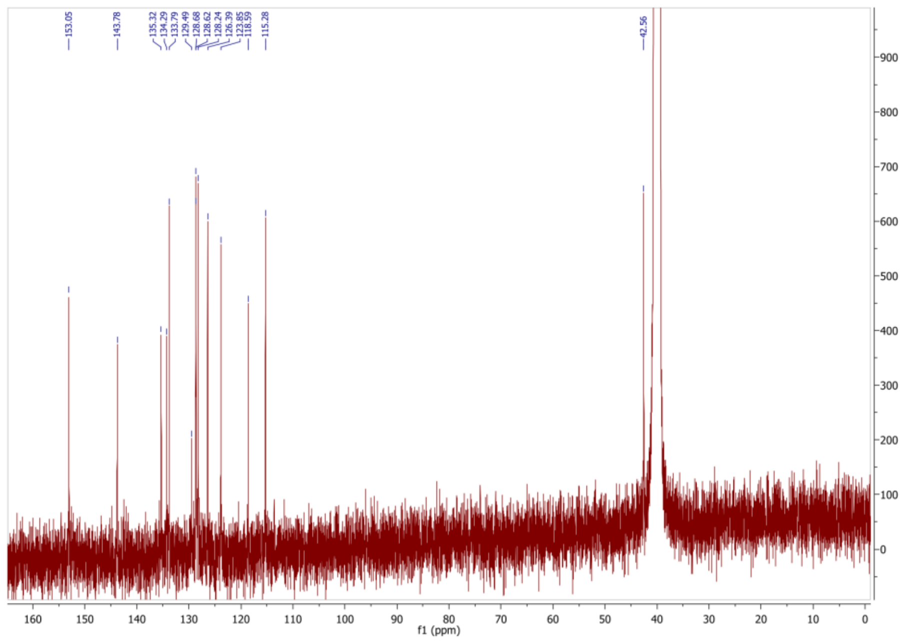

N-(3,4-dihydroquinazolin-2-yl)-3,4-dichlorobenzene-1-sulfonamide PR 21

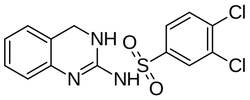

UPLC-MS:

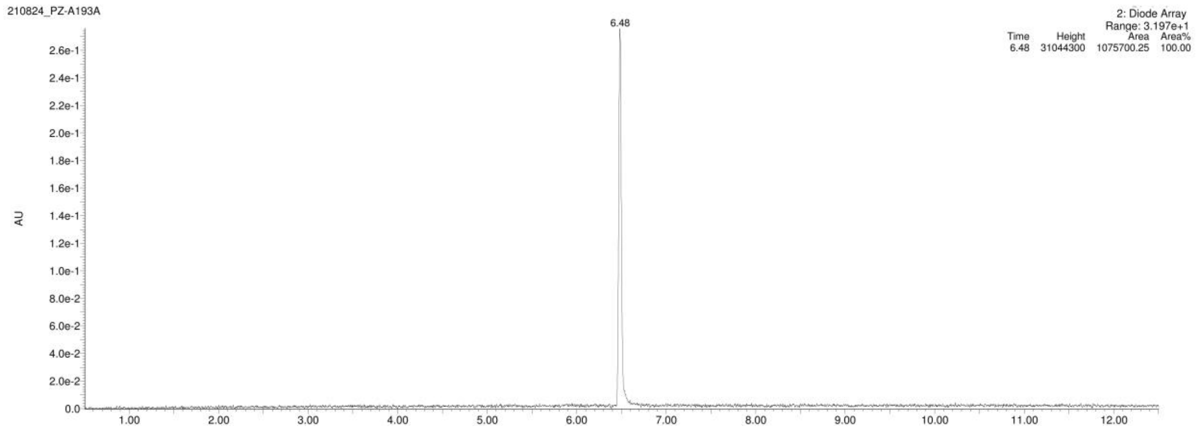
210824_PZ-A193A
6.48
2: Diode Array
Range: 3.197e+1
Time Height Area Area%
6.48 31044300 1075700.25 100.00
AU

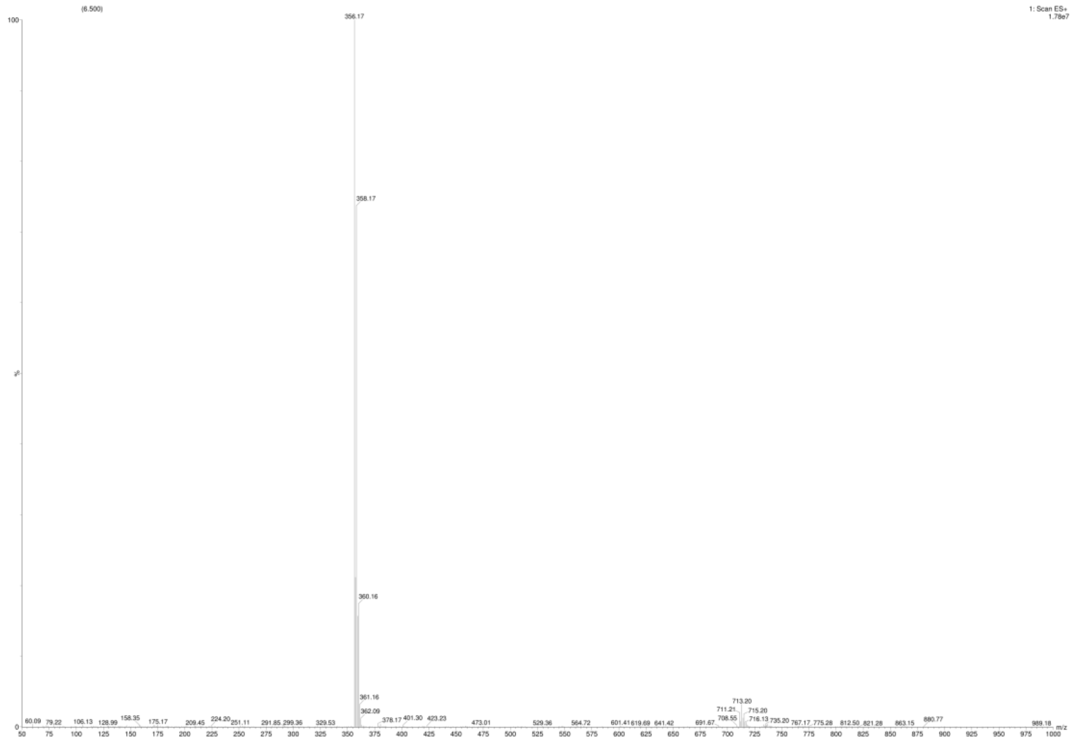
(6.500)
1: Scan ES+
356.17
358.17
360.16
361.16
362.09
713.20
m/z

^{1}H NMR:

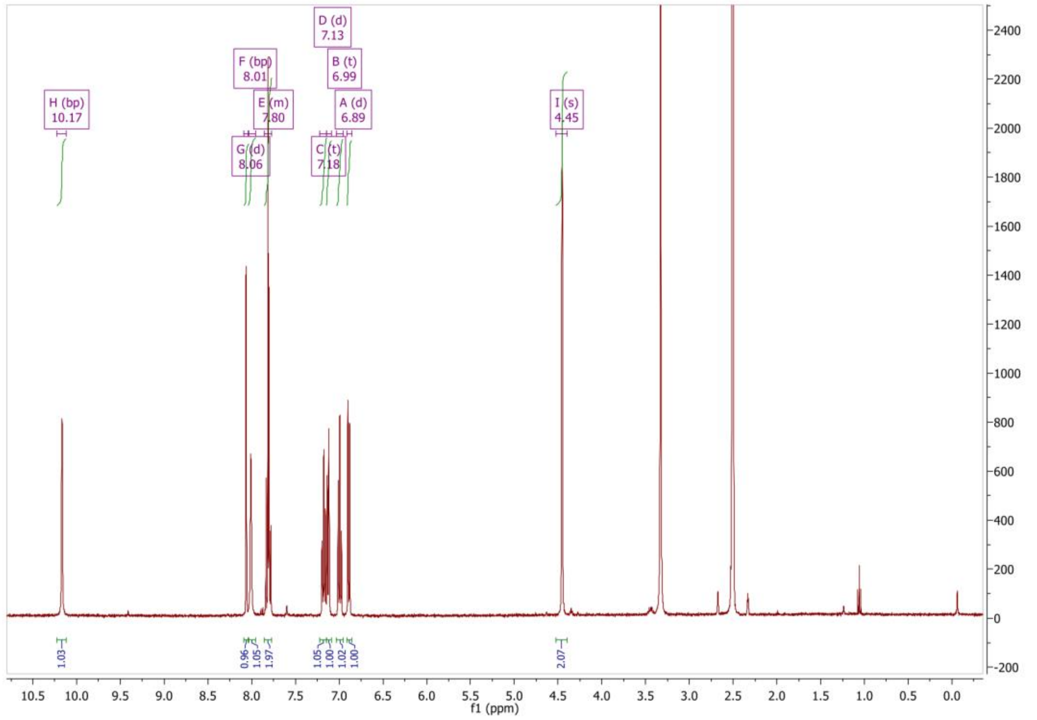

^{13}C NMR:

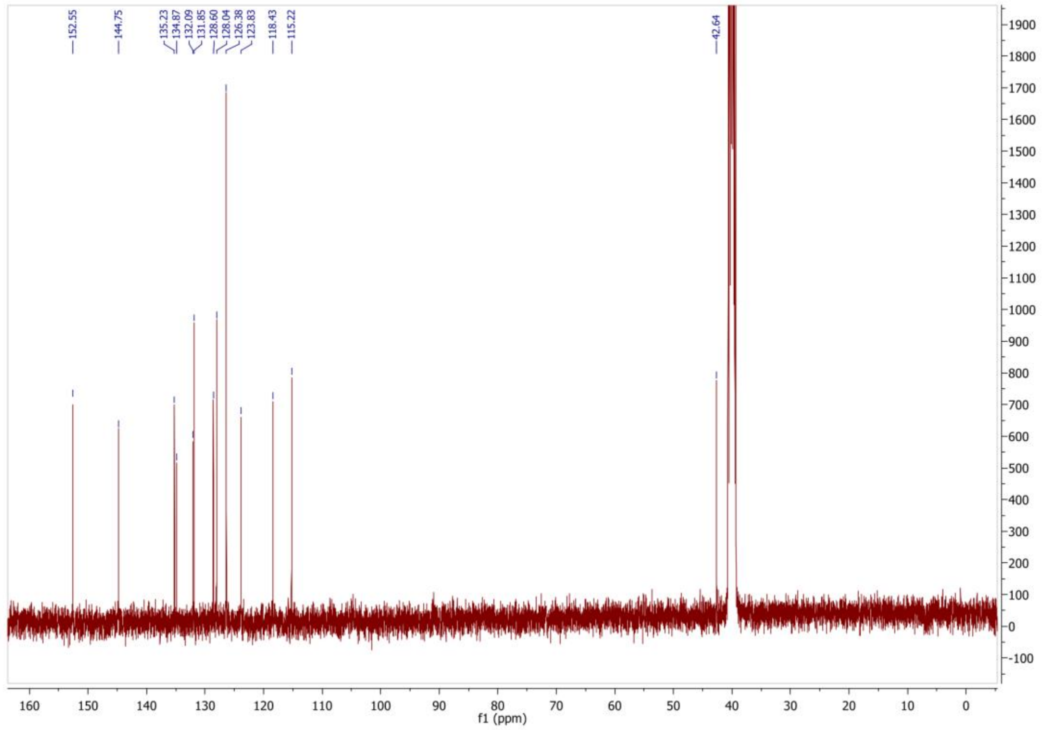

FT-IR:

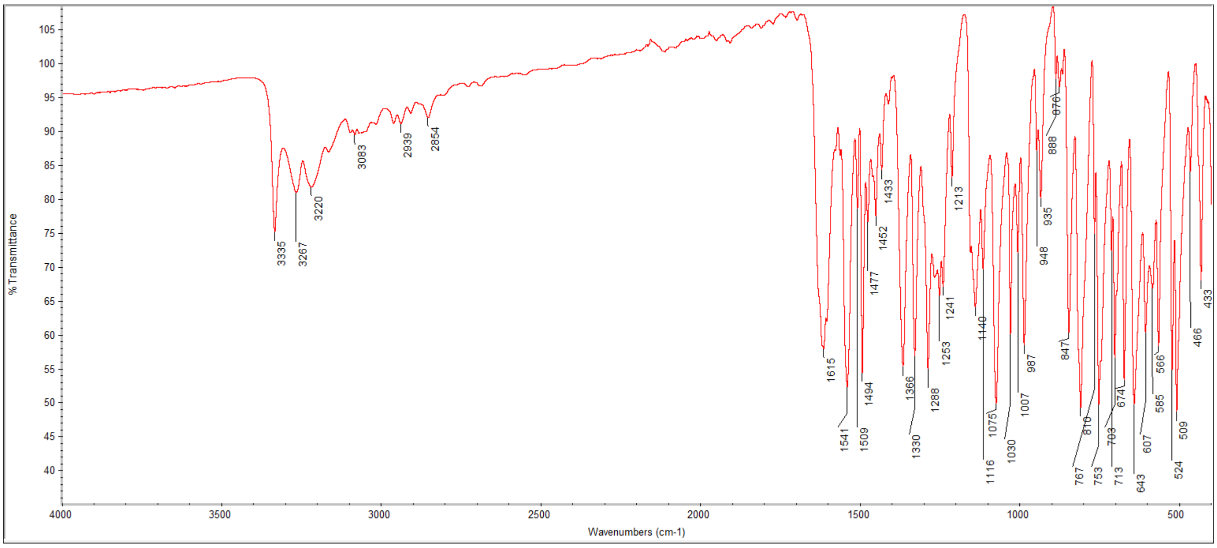

2,6-dichloro-*N*-(3,4-dihydroquinazolin-2-yl)benzene-1-sulfonamide PR 22

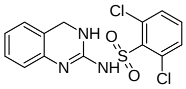

UPLC-MS:

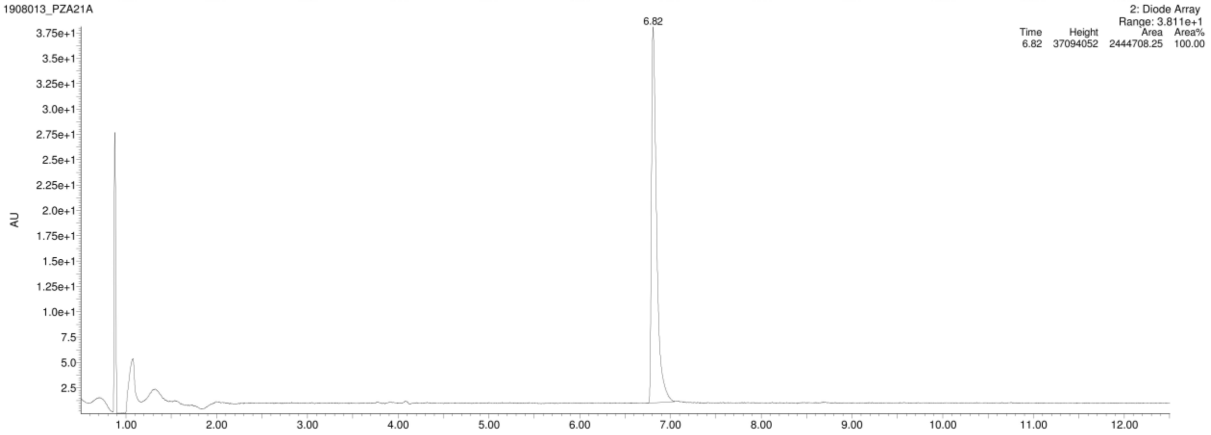

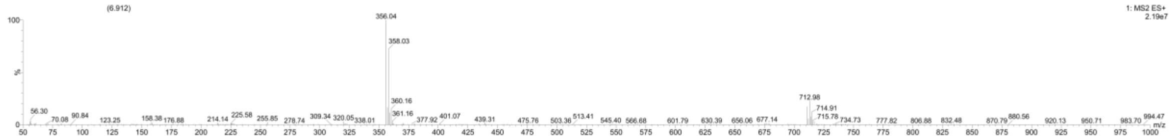

^{1}H NMR:

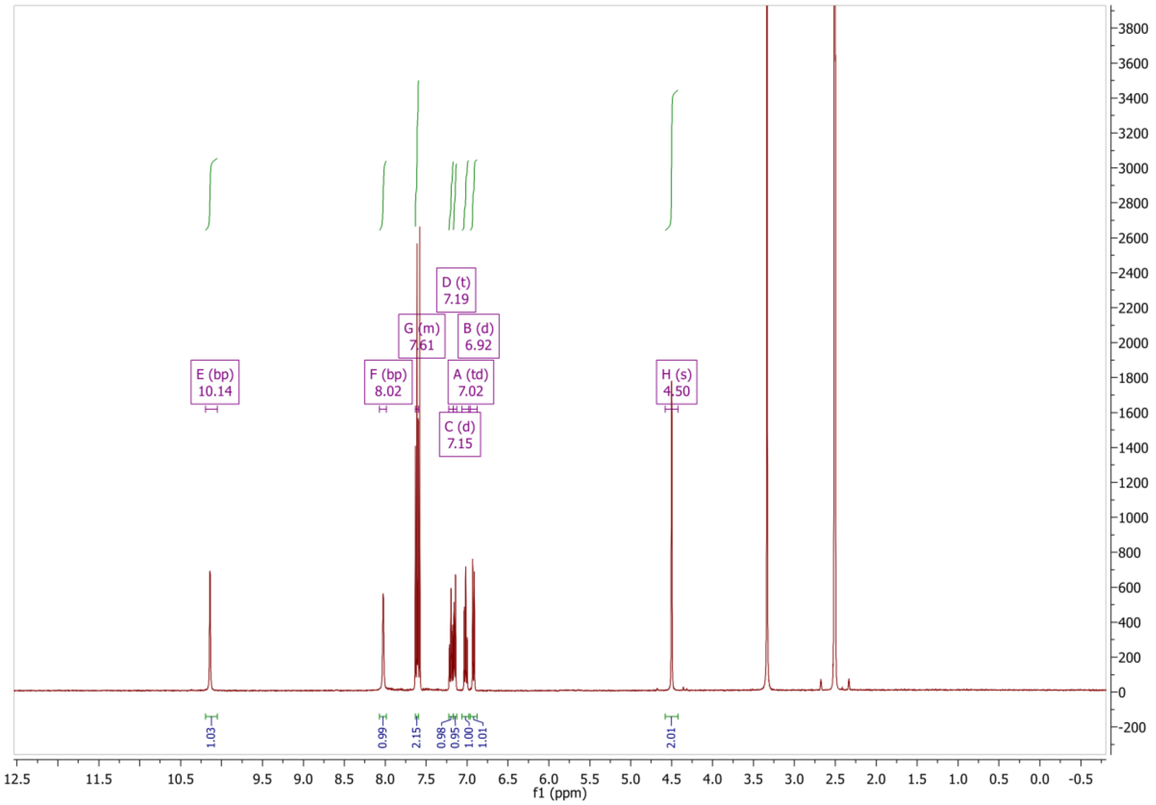

^{13}C NMR:

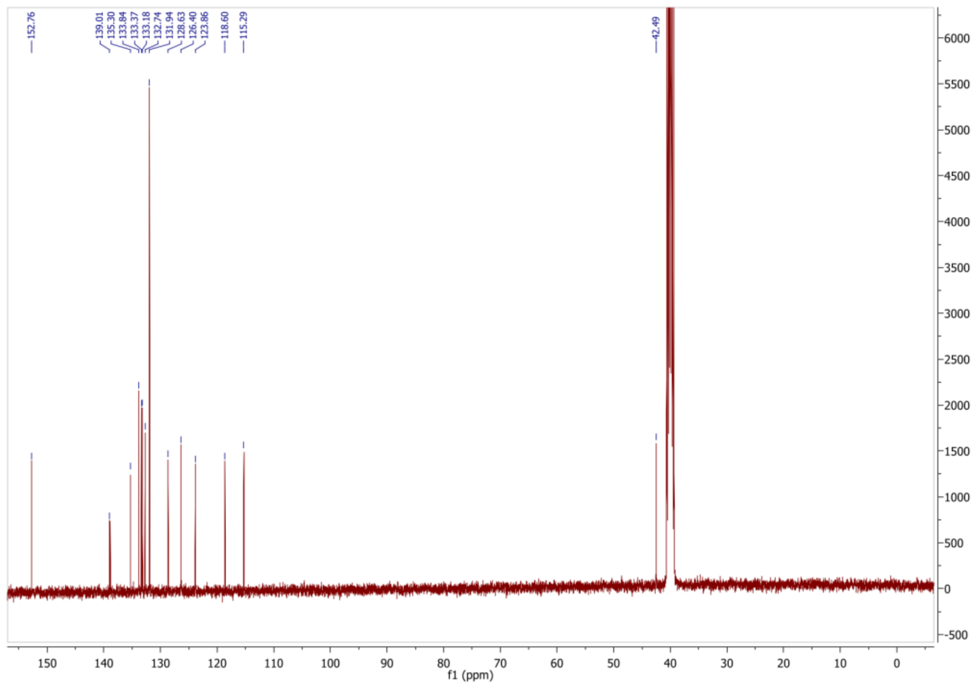

FT-IR:

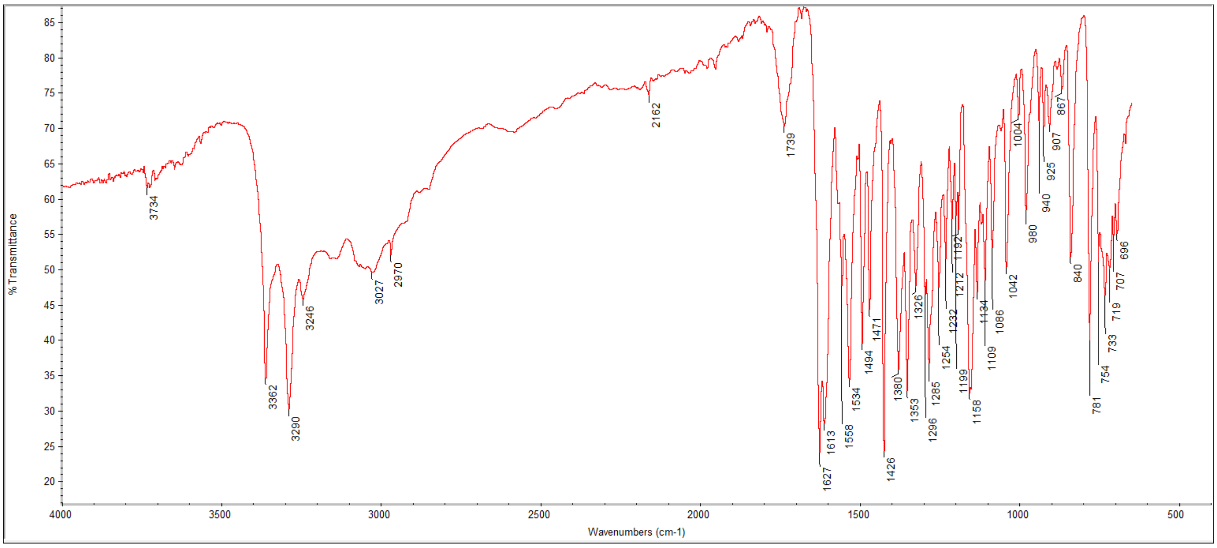

3,5-dichloro-*N*-(3,4-dihydroquinazolin-2-yl)benzene-1-sulfonamide PR 23

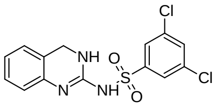

UPLC-MS:

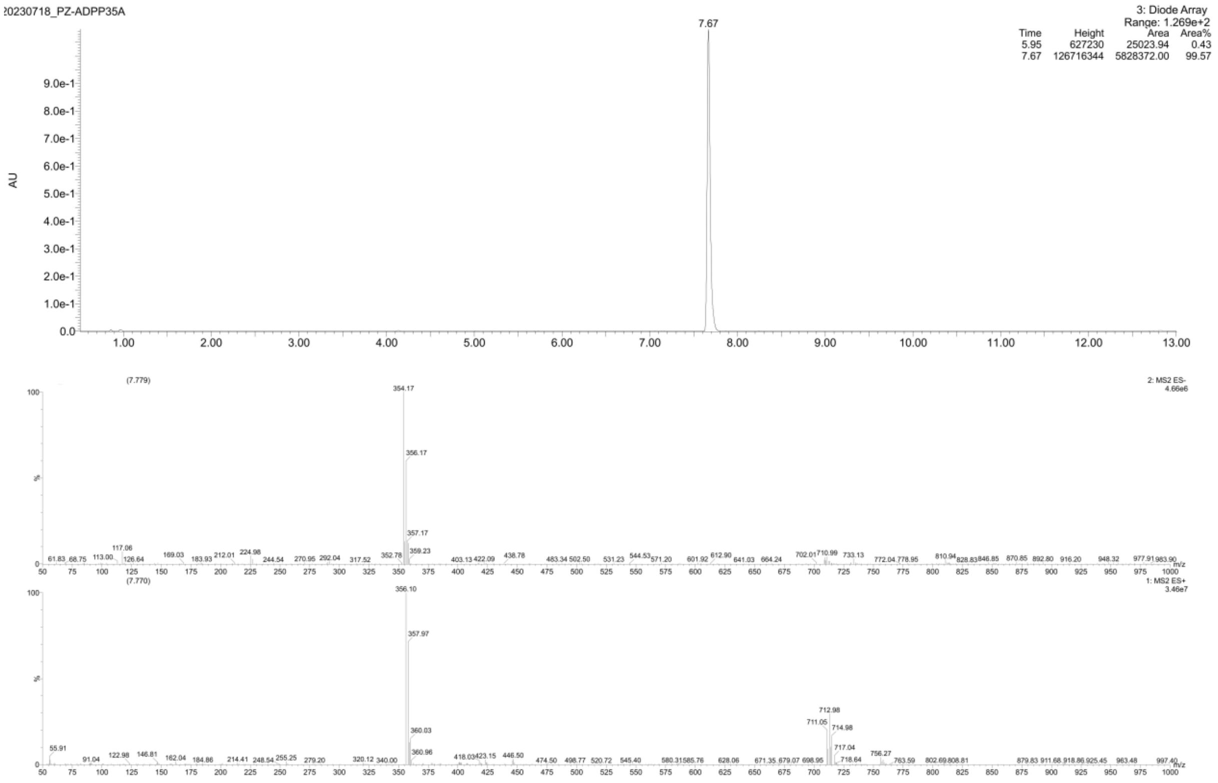

^{1}H NMR:

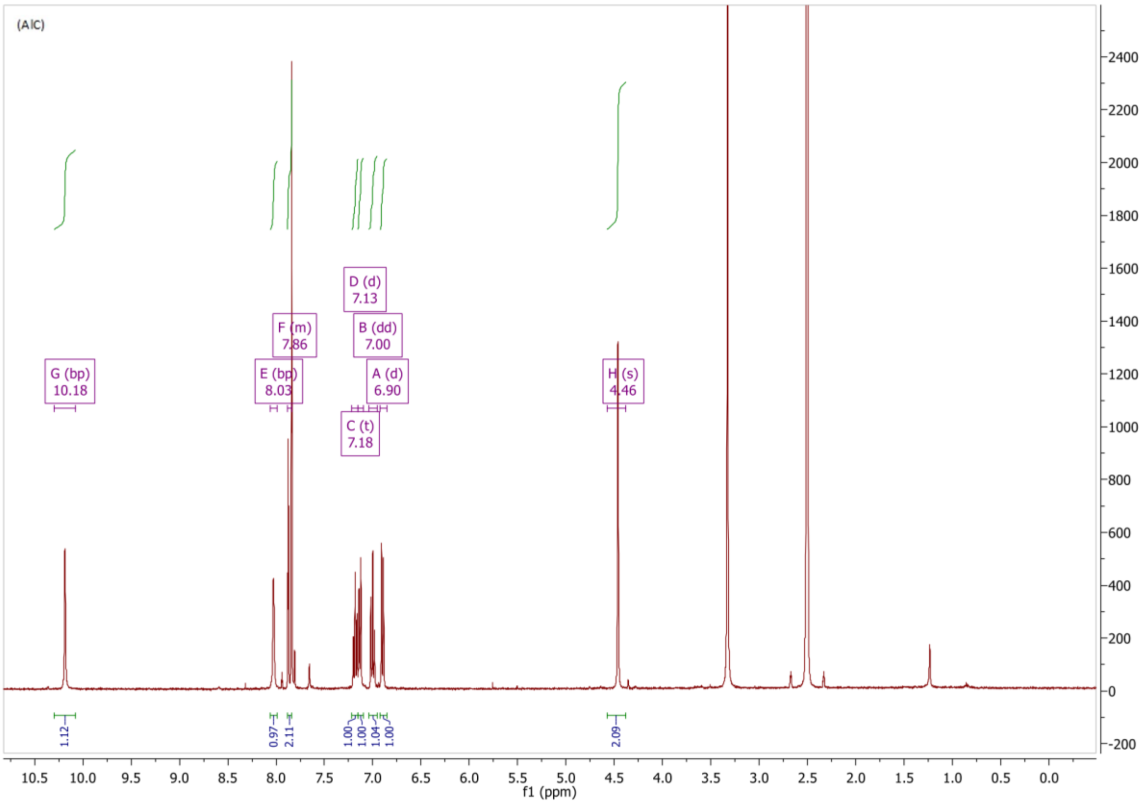

^{13}C NMR:

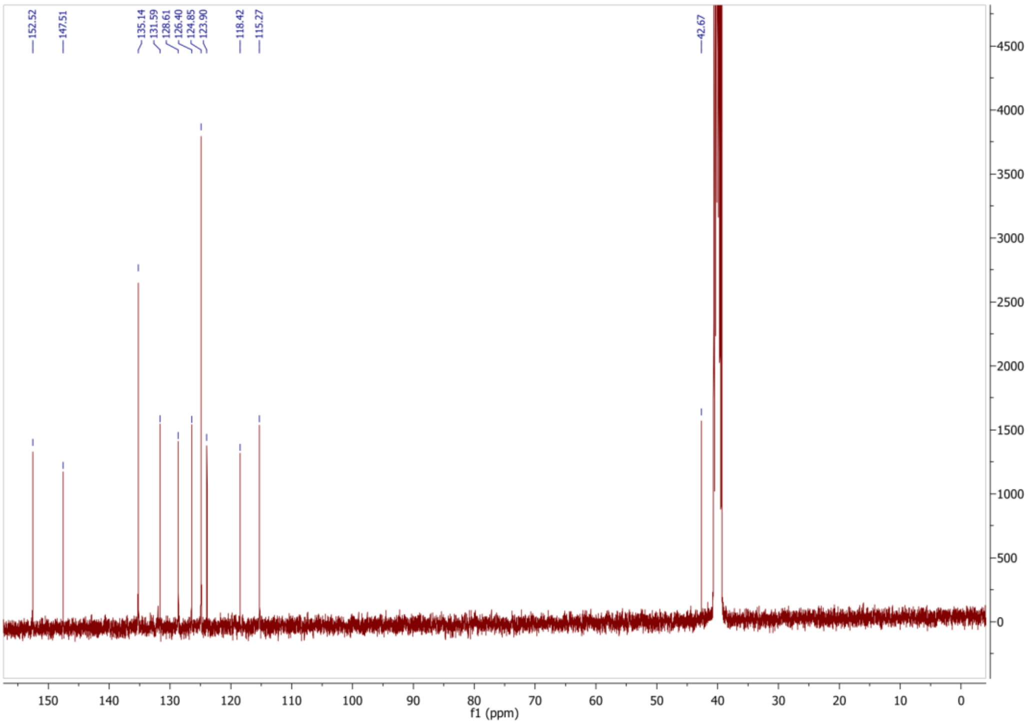

FT-IR:

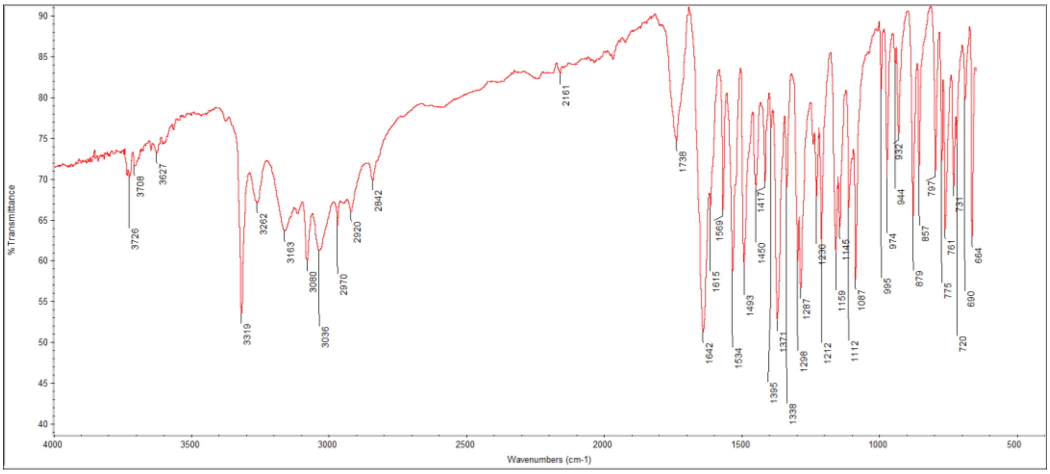

N-(3,4-dihydroquinazolin-2-yl)-3-methoxybenzene-1-sulfonamide PR 24

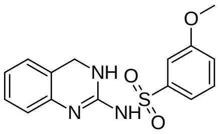

UPLC-MS:

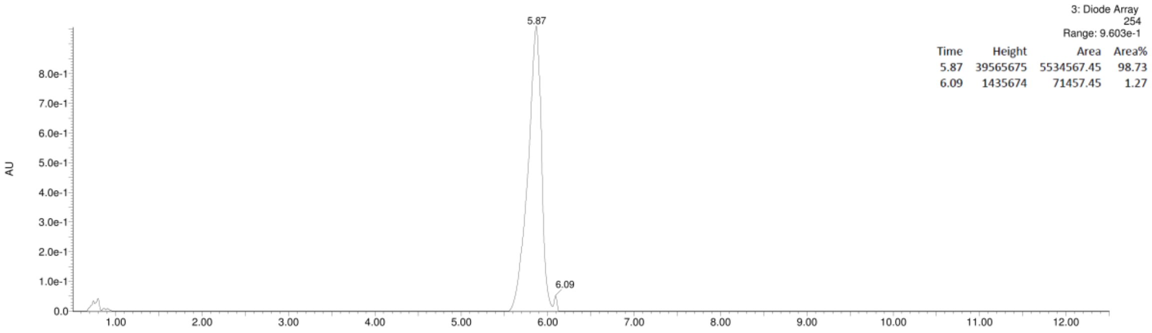

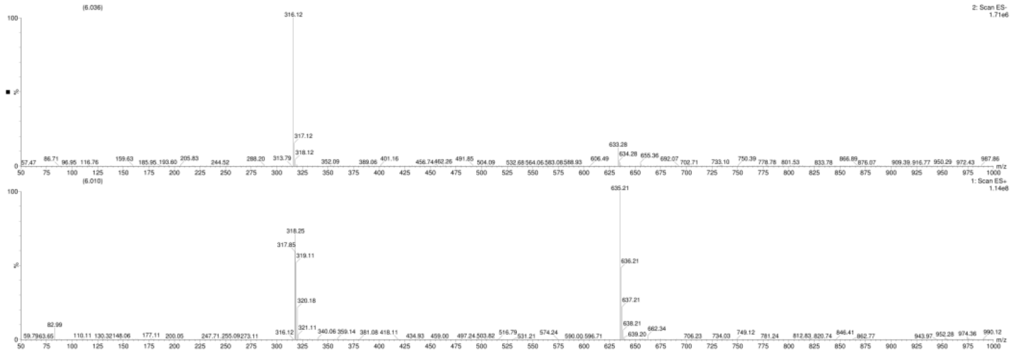

^{1}H NMR:

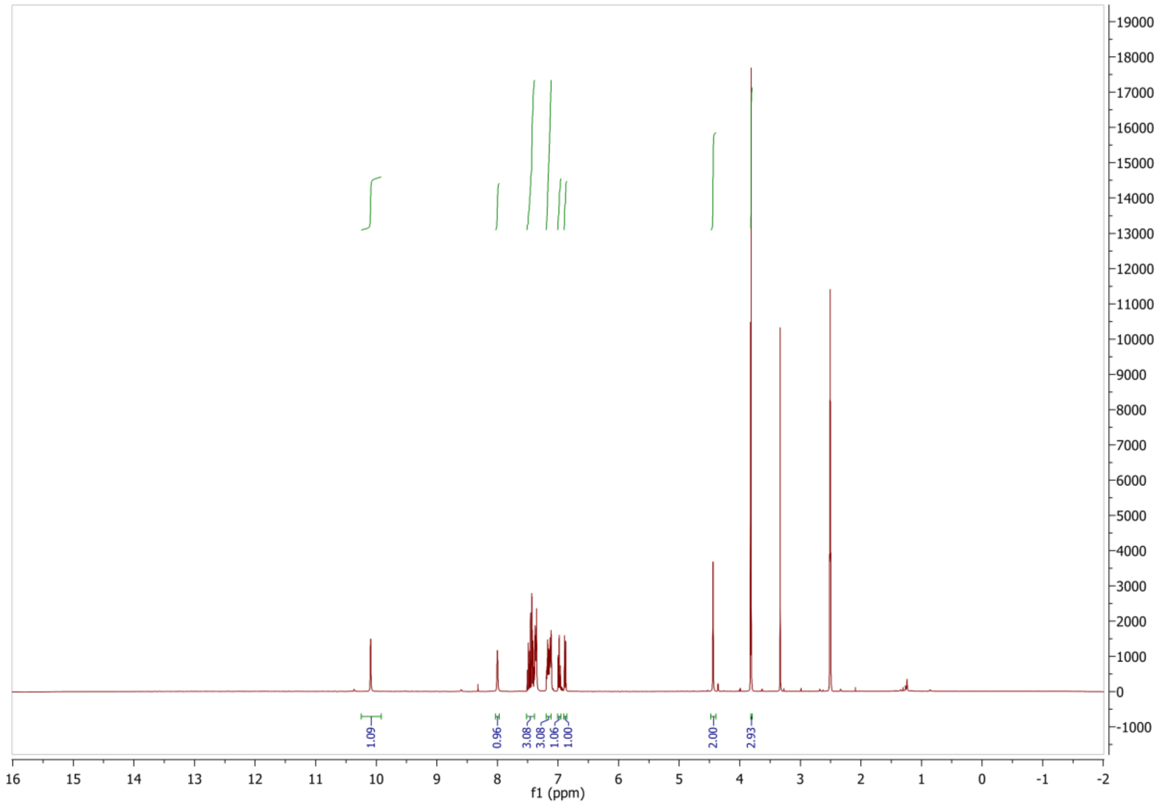

^{13}C NMR:

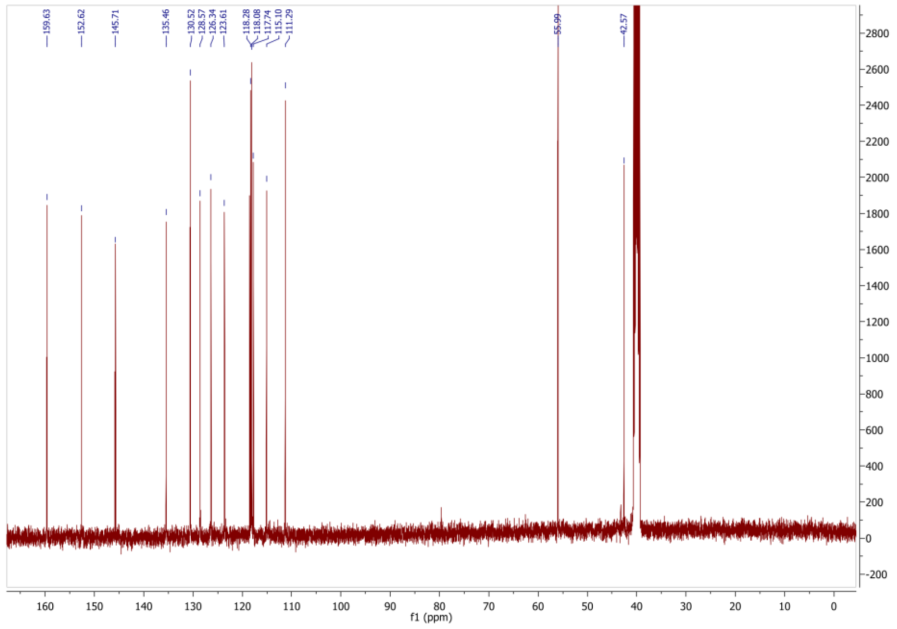

FT-IR:

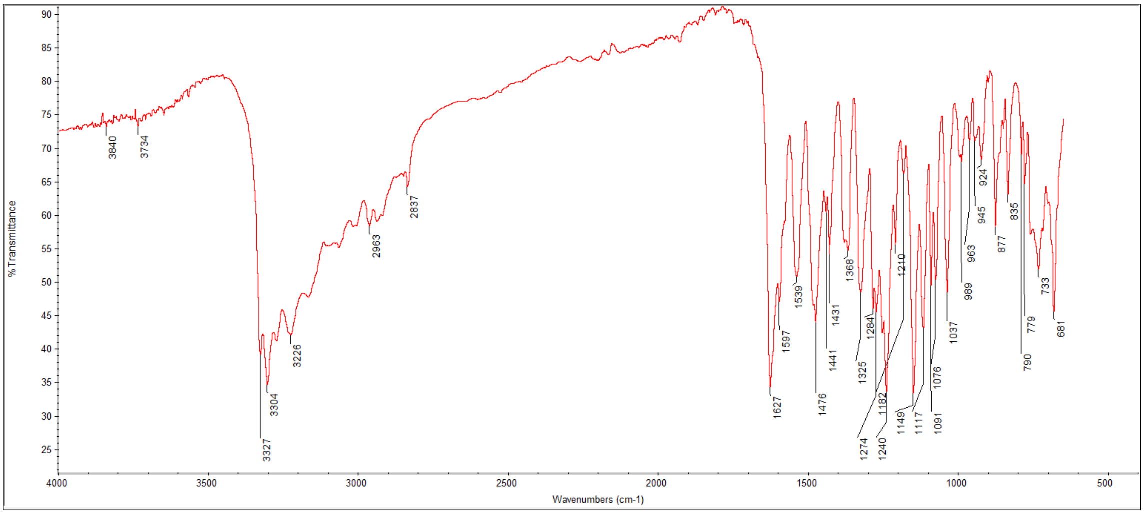

5-chloro-*N*-(3,4-dihydroquinazolin-2-yl)-2-methoxybenzene-1-sulfonamide PR 25

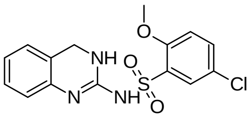

UPLC-MS:

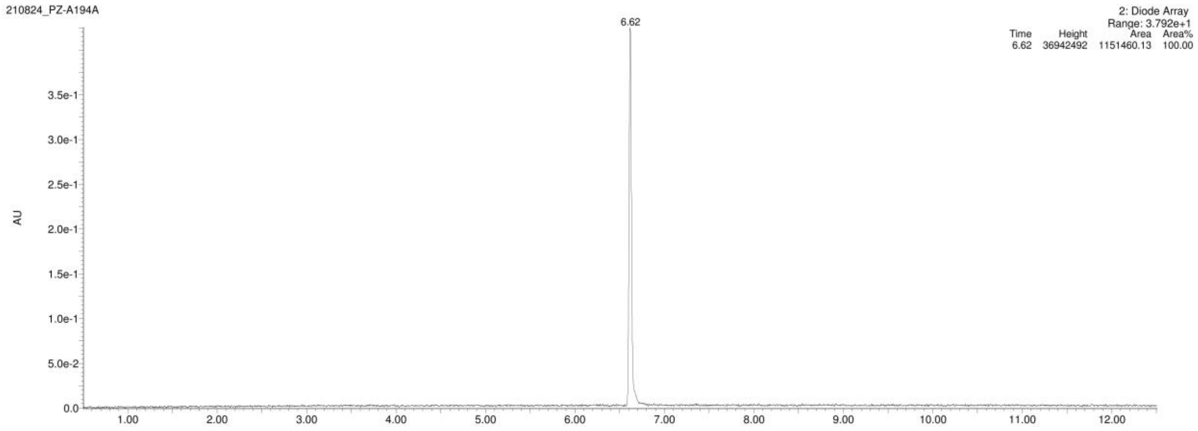

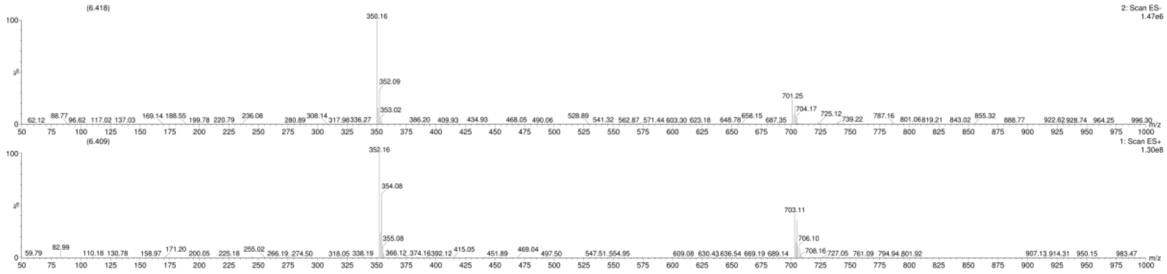

^{1}H NMR:

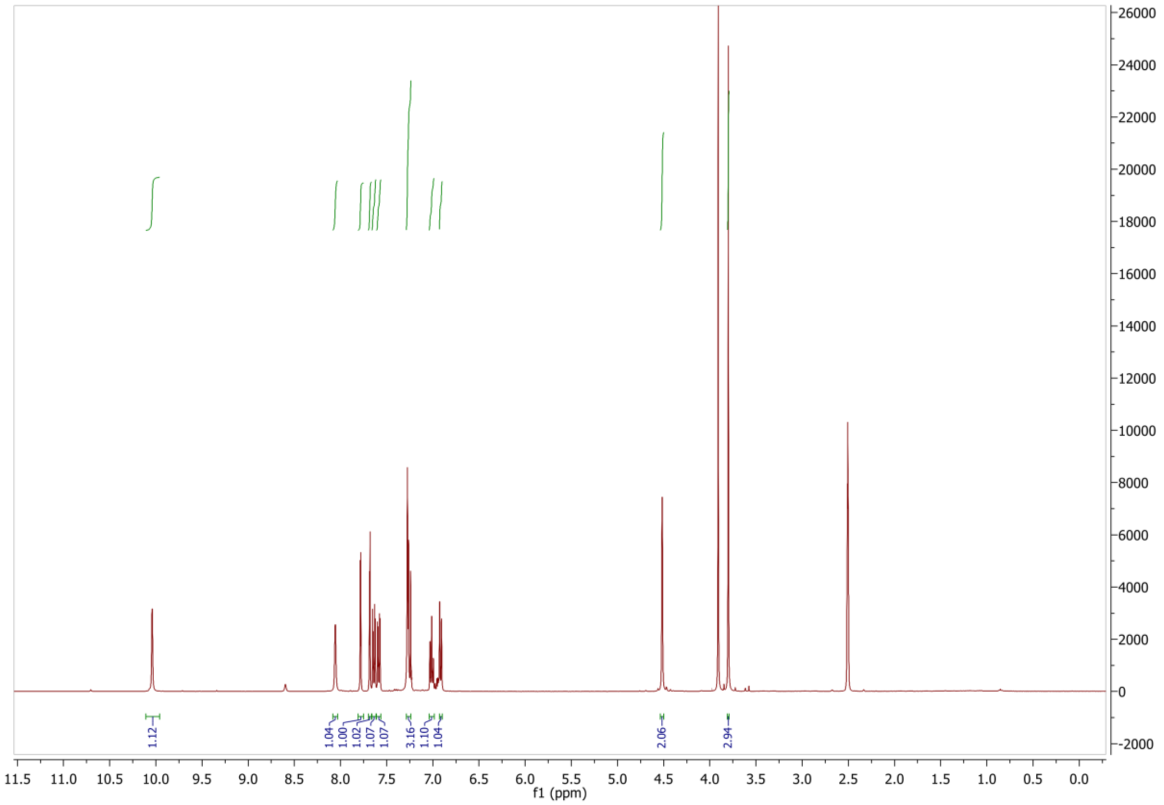

^{13}C NMR:

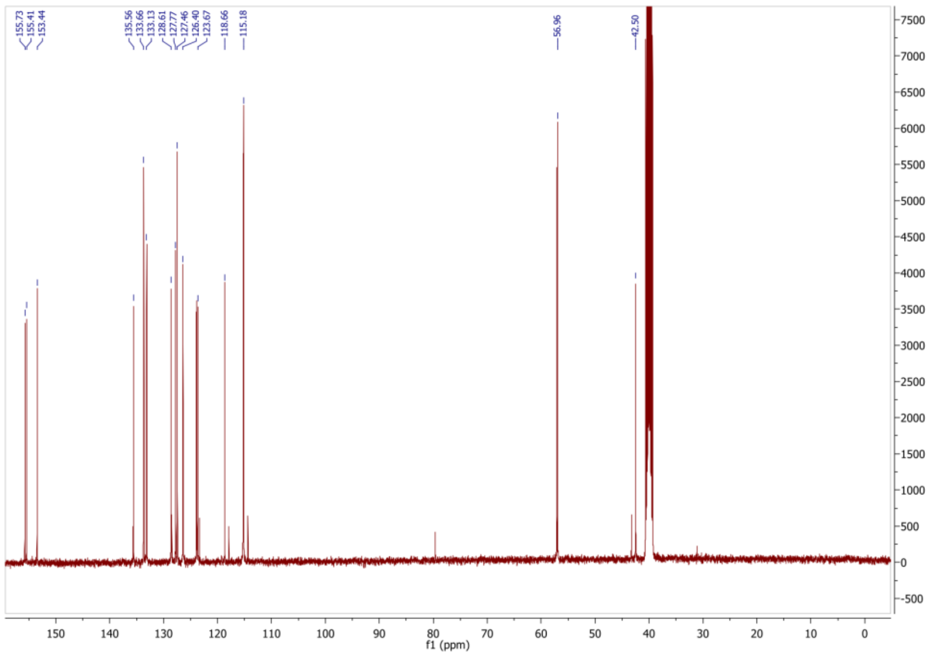

FT-IR:

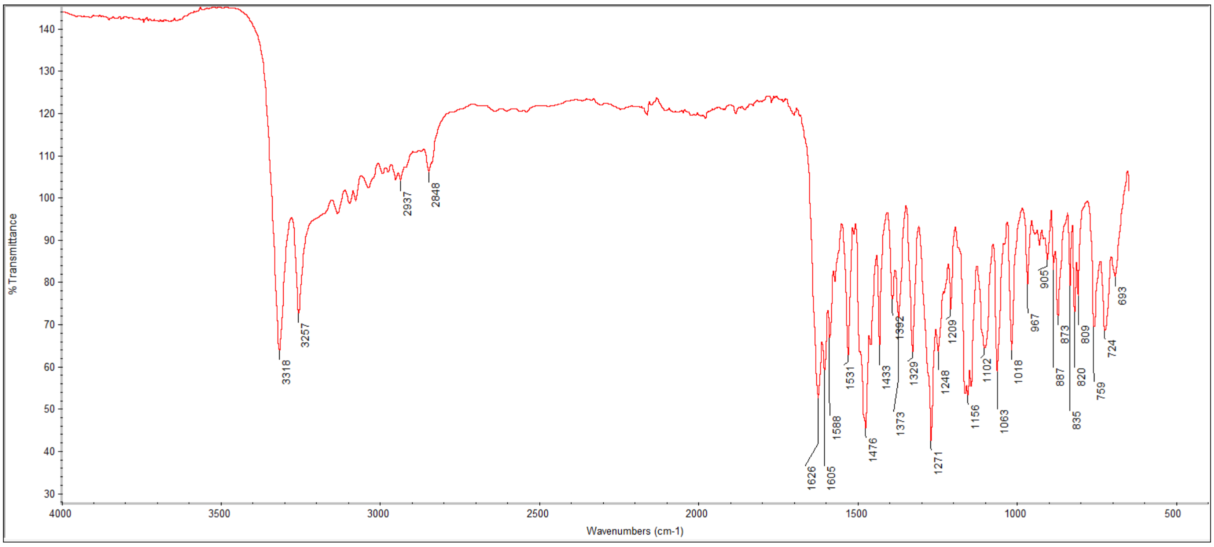

5-chloro-*N*-(3,4-dihydroquinazolin-2-yl)naphthalene-2-sulfonamide PR 30

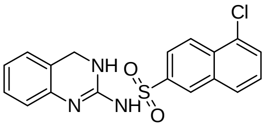

UPLC-MS:

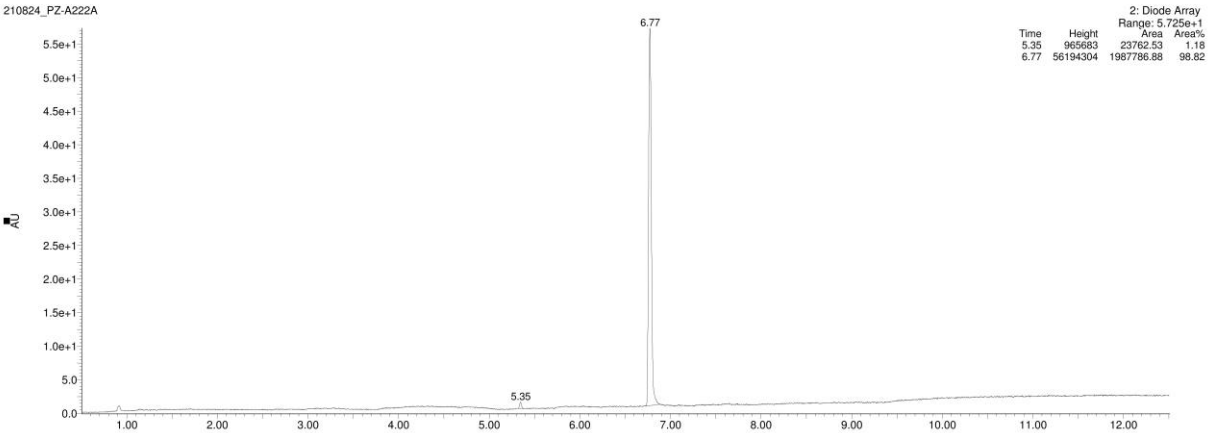

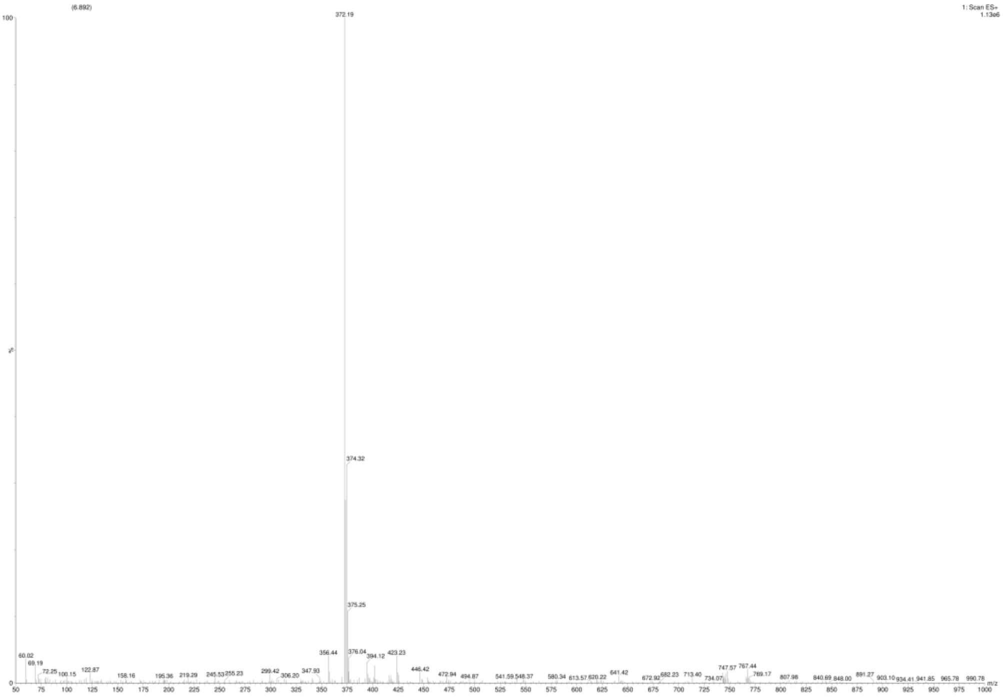
1: Scan ES+
1.13e6
372.19
374.32
375.25
m/z

^{1}H NMR:

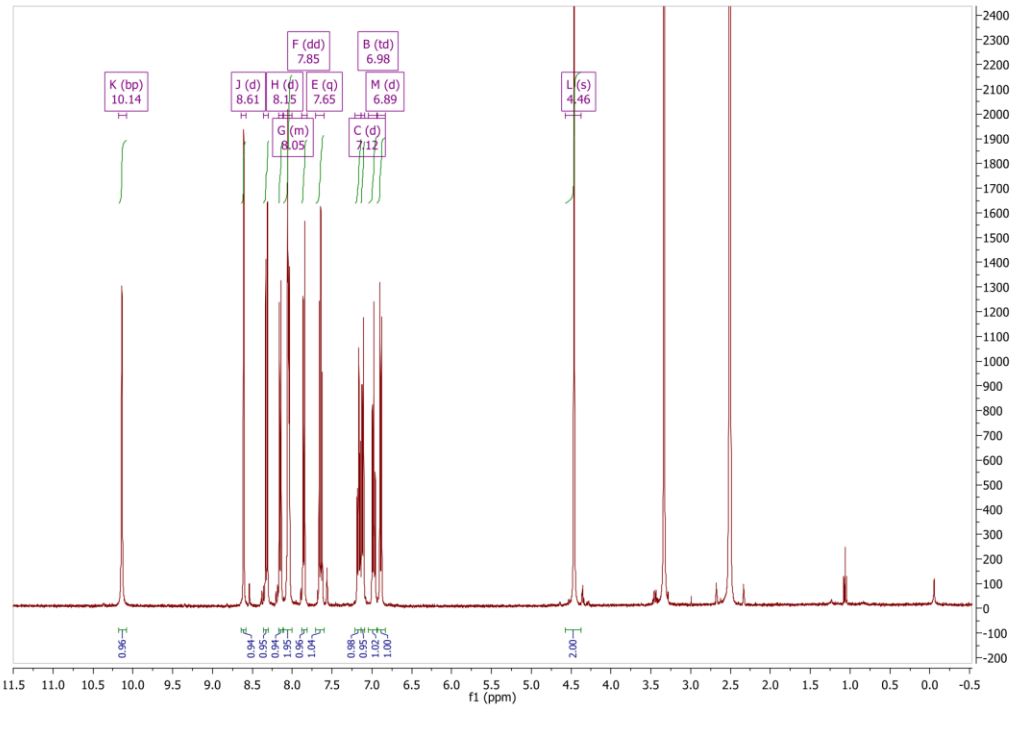
K (bp)
10.14
J (d)
8.61
H (d)
8.15
F (dd)
7.85
E (q)
7.65
G (m)
8.05
B (td)
6.98
M (d)
6.89
C (d)
7.12
L (s)
4.46
0.96
0.94
0.95
0.94
1.95
0.96
1.04
0.98
0.95
1.02
1.00
2.00
f1 (ppm)

^{13}C NMR:

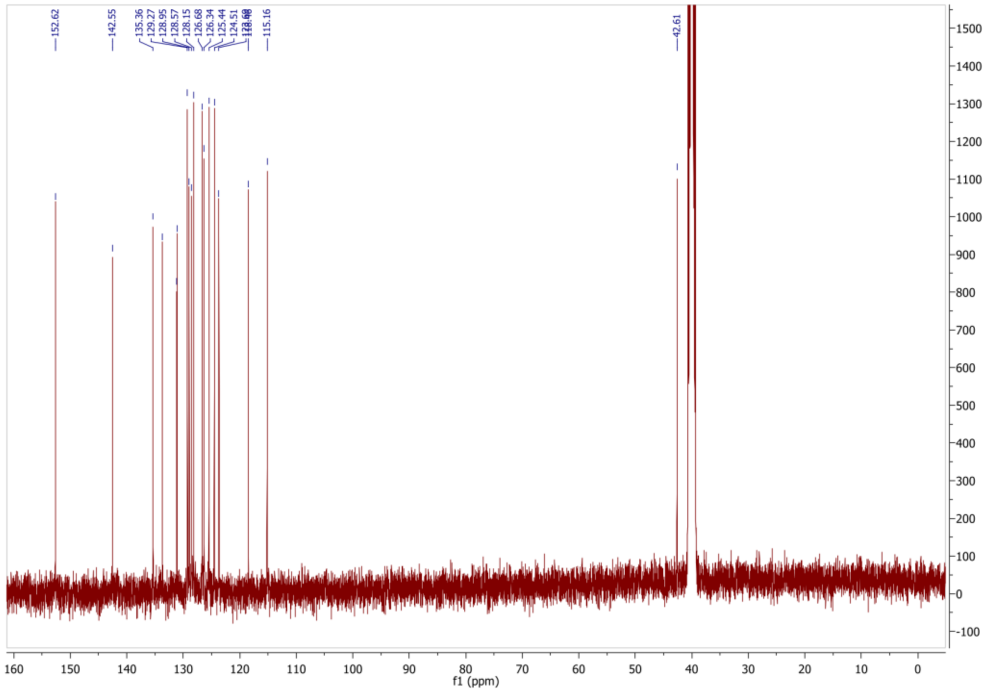

FT-IR:

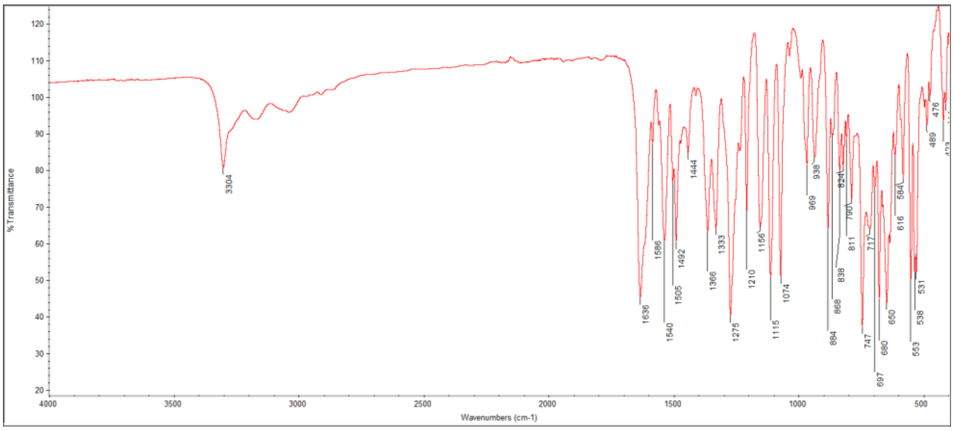

N-(5-chloro-3,4-dihydroquinazolin-2-yl)naphthalene-2-sulfonamide PR 31

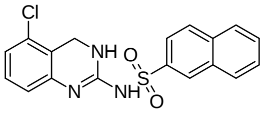

UPLC-MS:

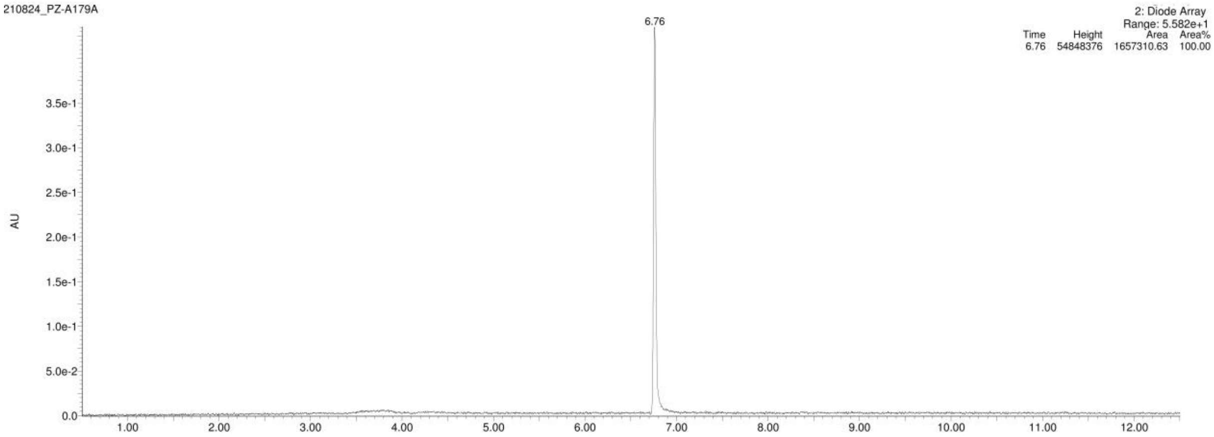
210824_PZ-A179A
6.76
2: Diode Array
Range: 5.582e+1
Time Height Area Area%
6.76 54848376 1657310.63 100.00
AU
3.5e-1
3.0e-1
2.5e-1
2.0e-1
1.5e-1
1.0e-1
5.0e-2
0.0
1.00 2.00 3.00 4.00 5.00 6.00 7.00 8.00 9.00 10.00 11.00 12.00

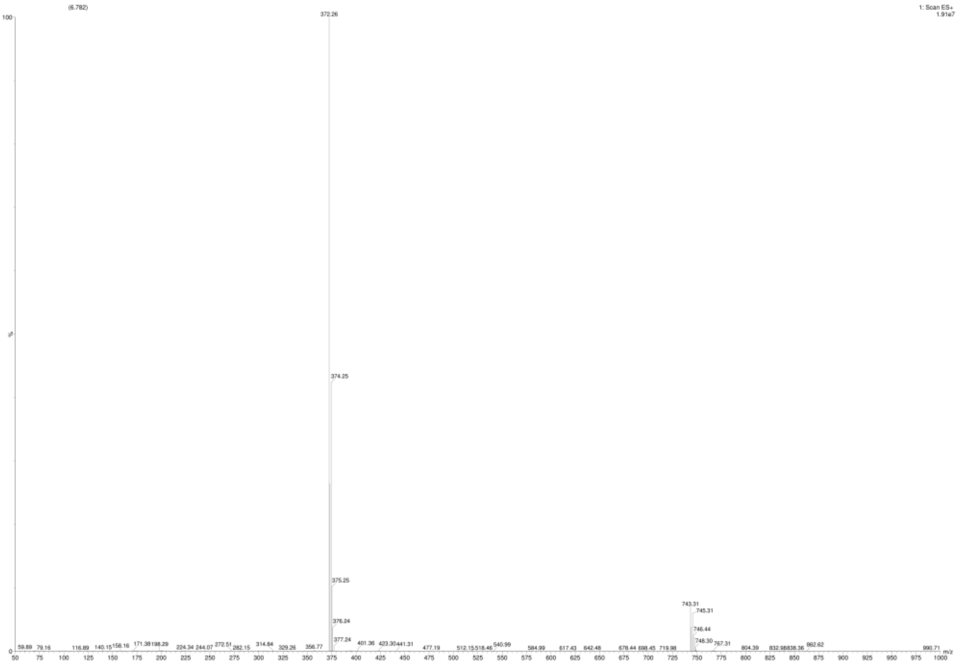
(6.782)
372.26
1: Scan ES+
1.91e7
100
%
374.25
375.25
376.24
377.24
743.31
745.31
746.44
748.30
0
m/z

^{1}H NMR:

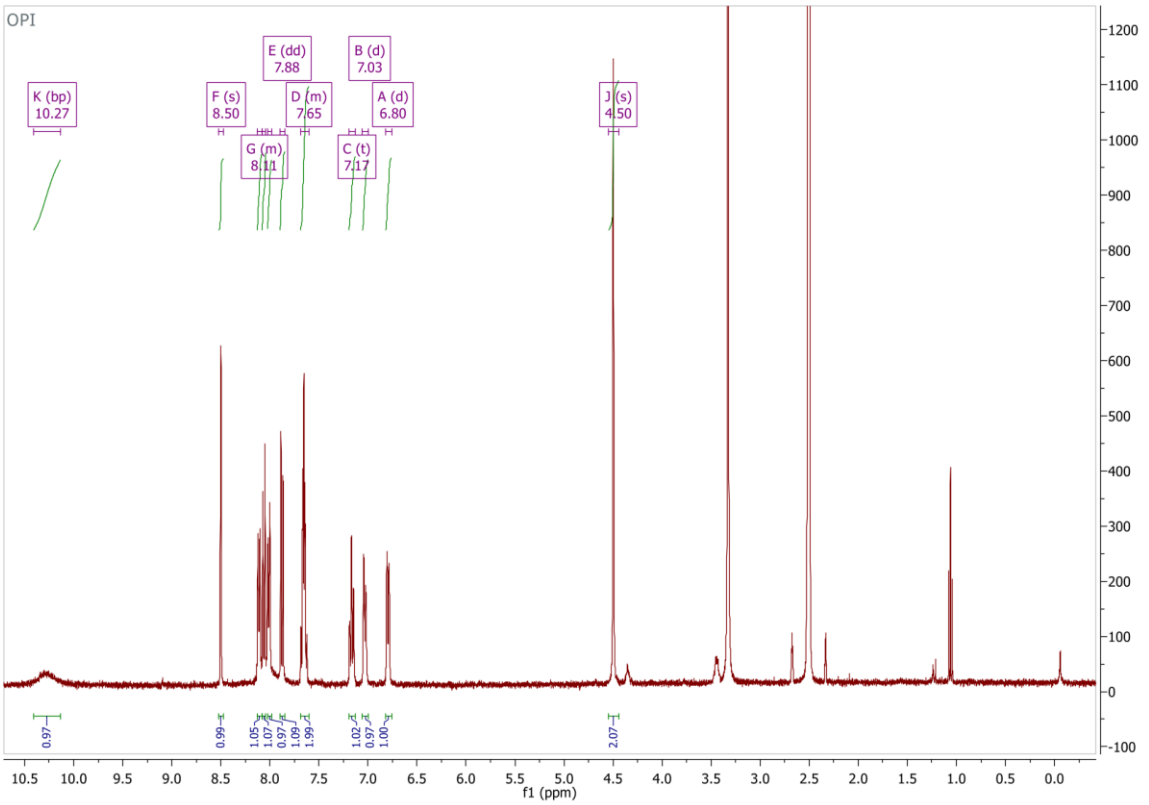

^{13}C NMR:

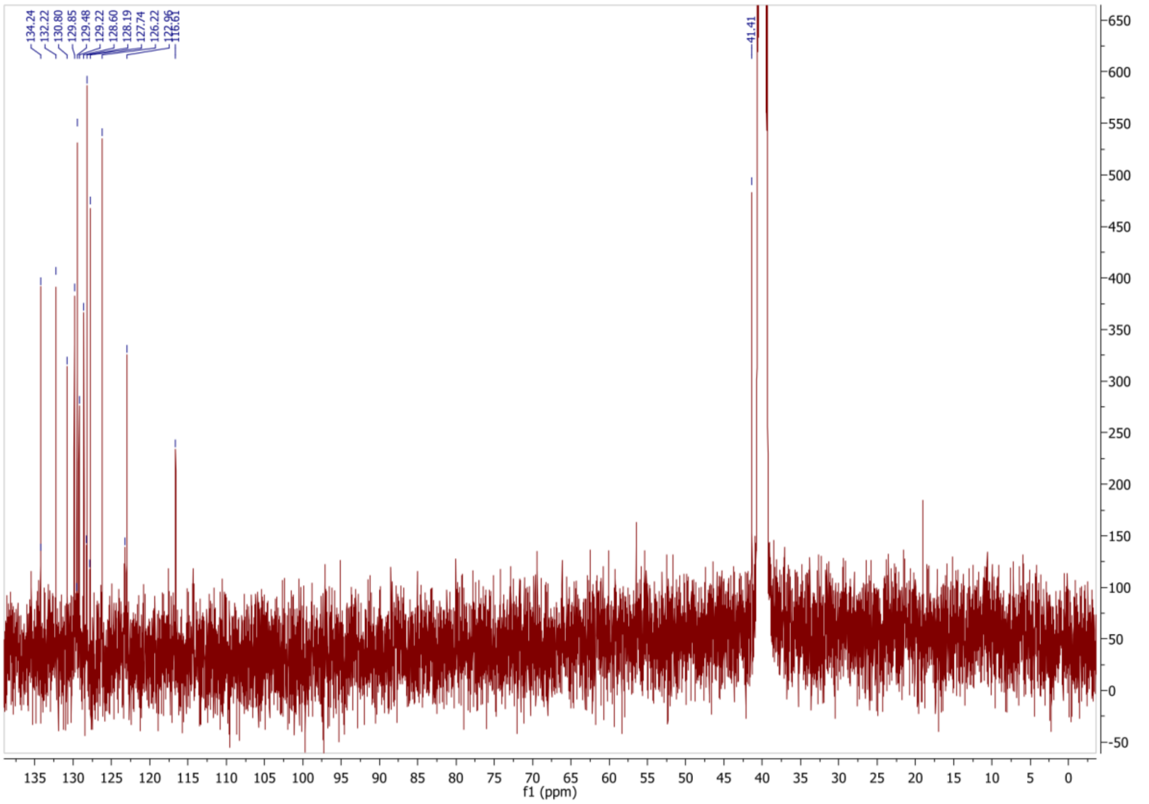

FT-IR:

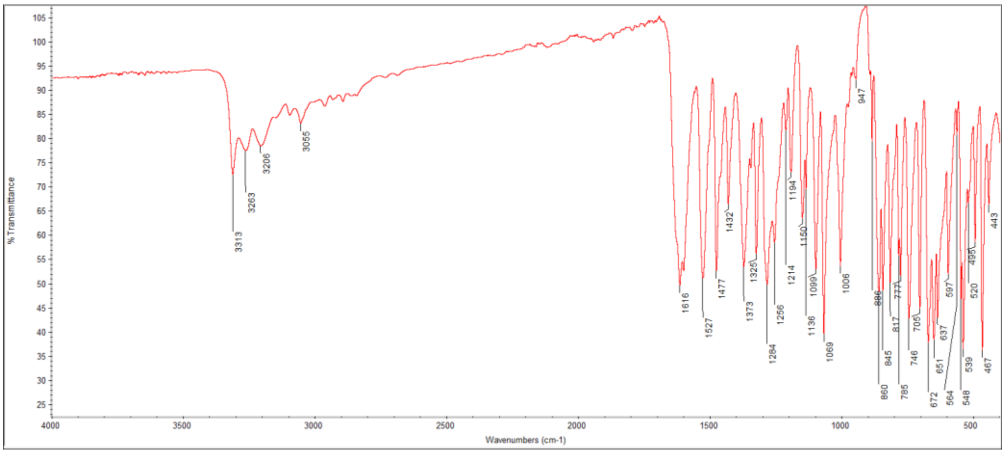

N-(7-chloro-3,4-dihydroquinazolin-2-yl)naphthalene-2-sulfonamide PR 33

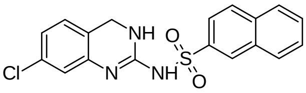

UPLC-MS:

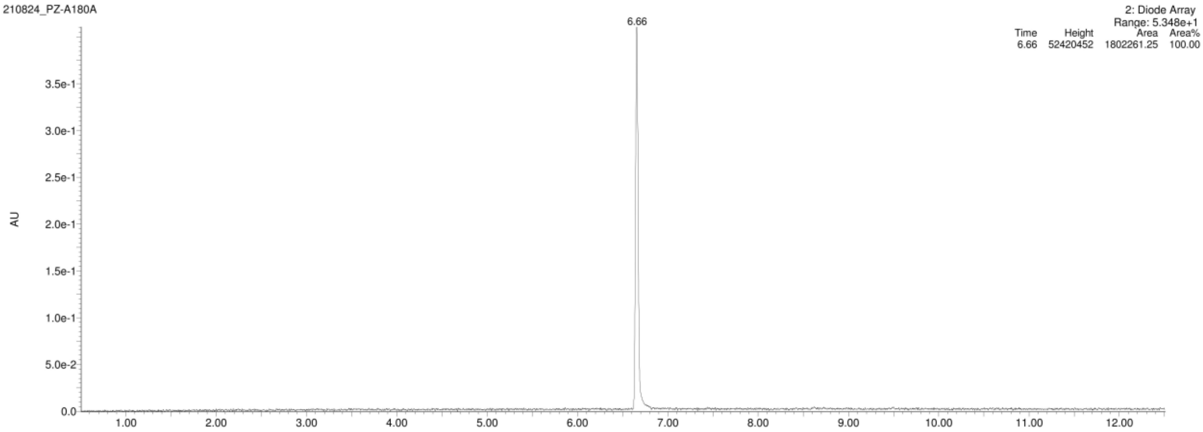

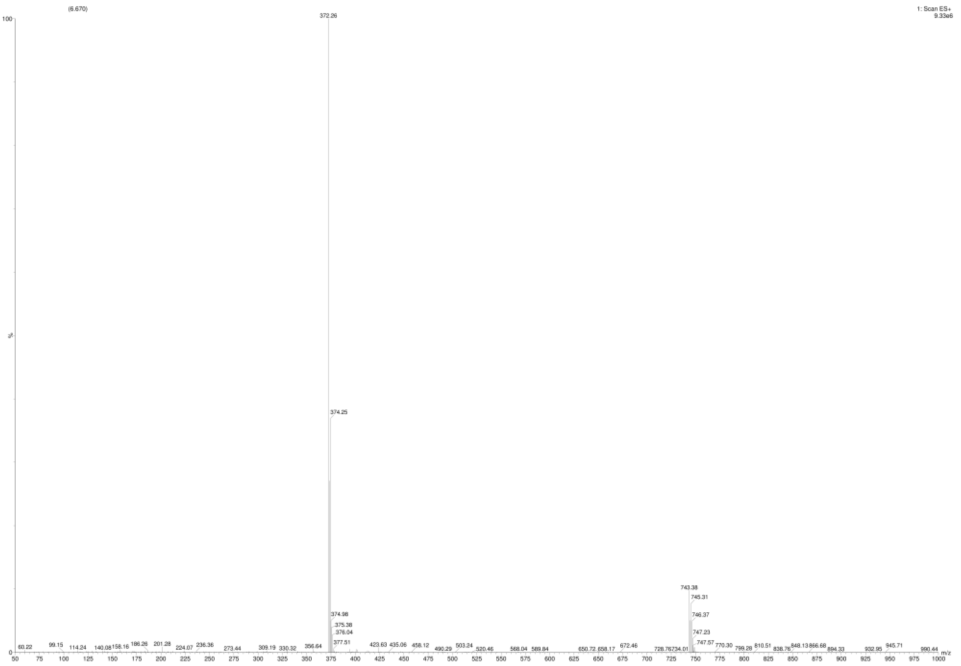

(6.670)
1: Scan ES+
9.33e6
372.26
374.25
743.38
745.31
m/z

^{1}H NMR:

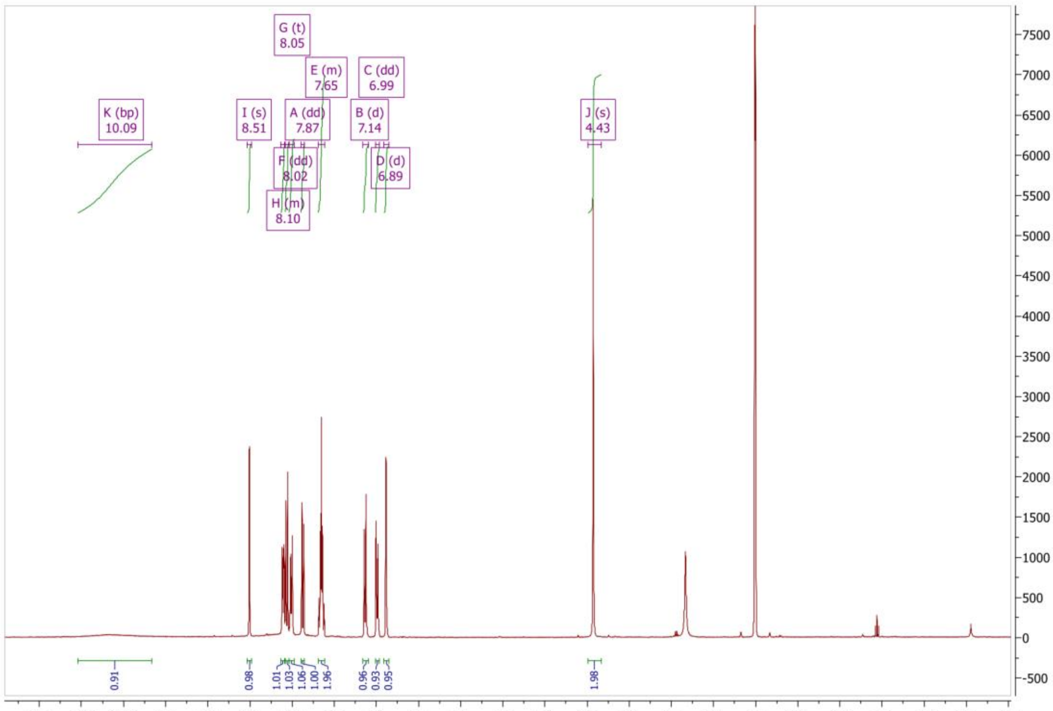

K (bp)
10.09
I (s)
8.51
A (dd)
7.87
G (t)
8.05
E (m)
7.65
C (dd)
6.99
B (d)
7.14
F (dd)
8.02
H (m)
8.10
D (d)
6.89
J (s)
4.43
f1 (ppm)

^{13}C NMR:

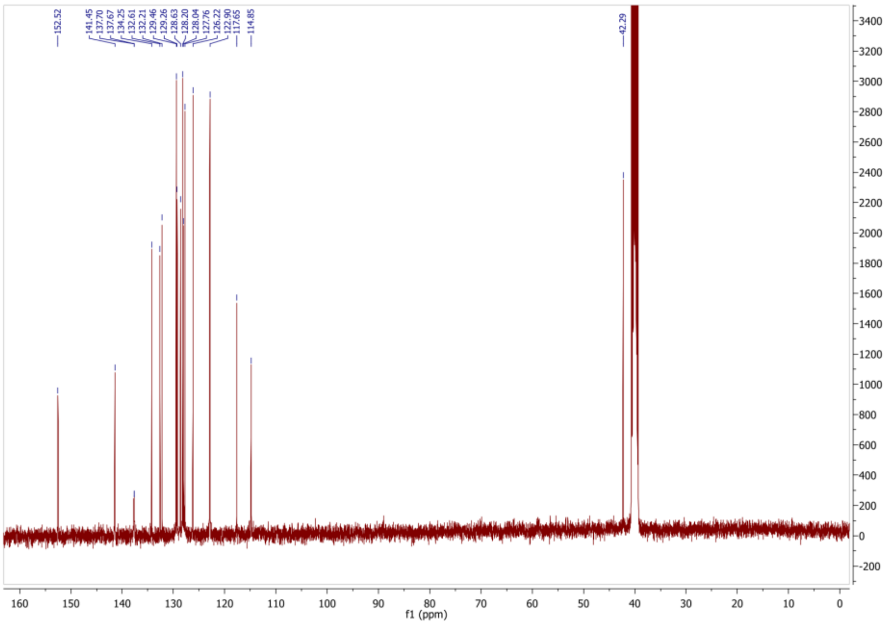

FT-IR:

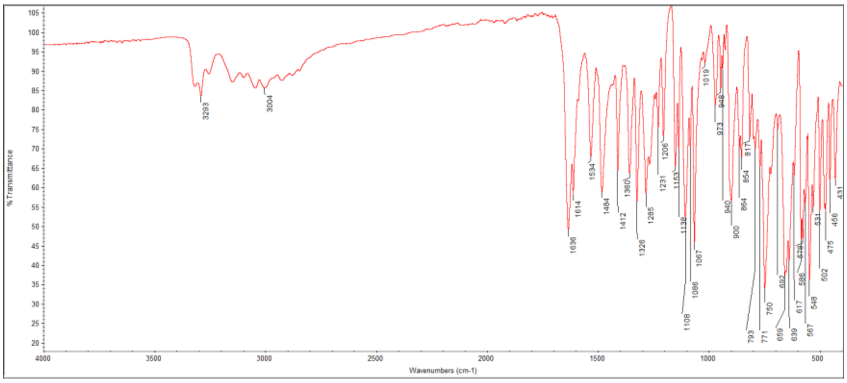

N-(7-chloro-4-methyl-3,4-dihydroquinazolin-2-yl)naphthalene-2-sulfonamide PR 37

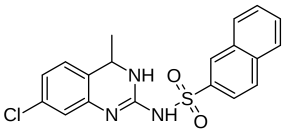

UPLC-MS:

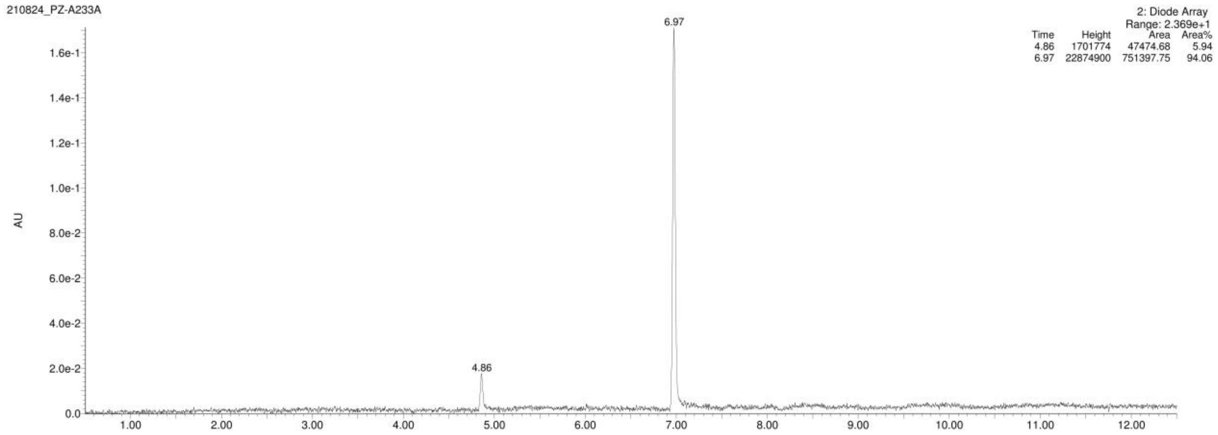

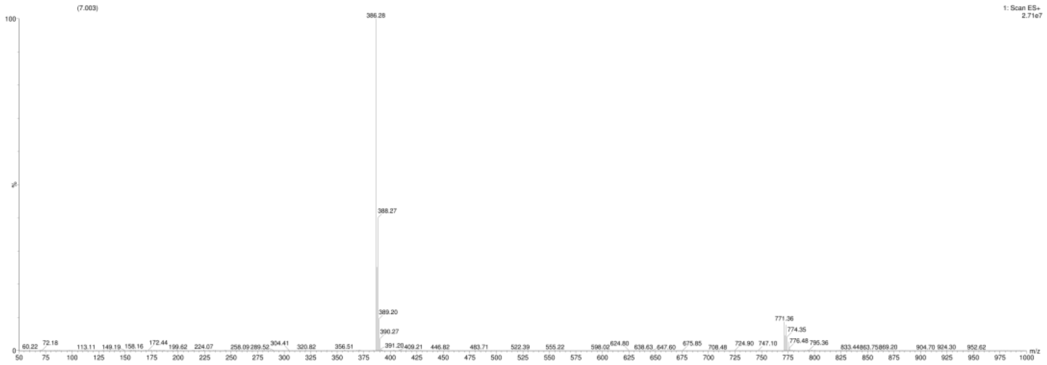

^{1}H NMR:

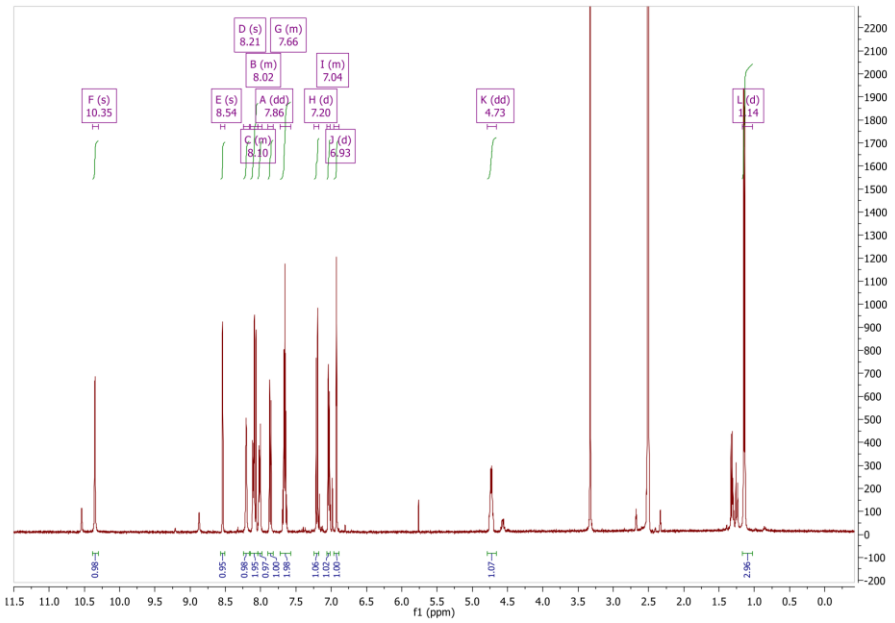

^{13}C NMR:

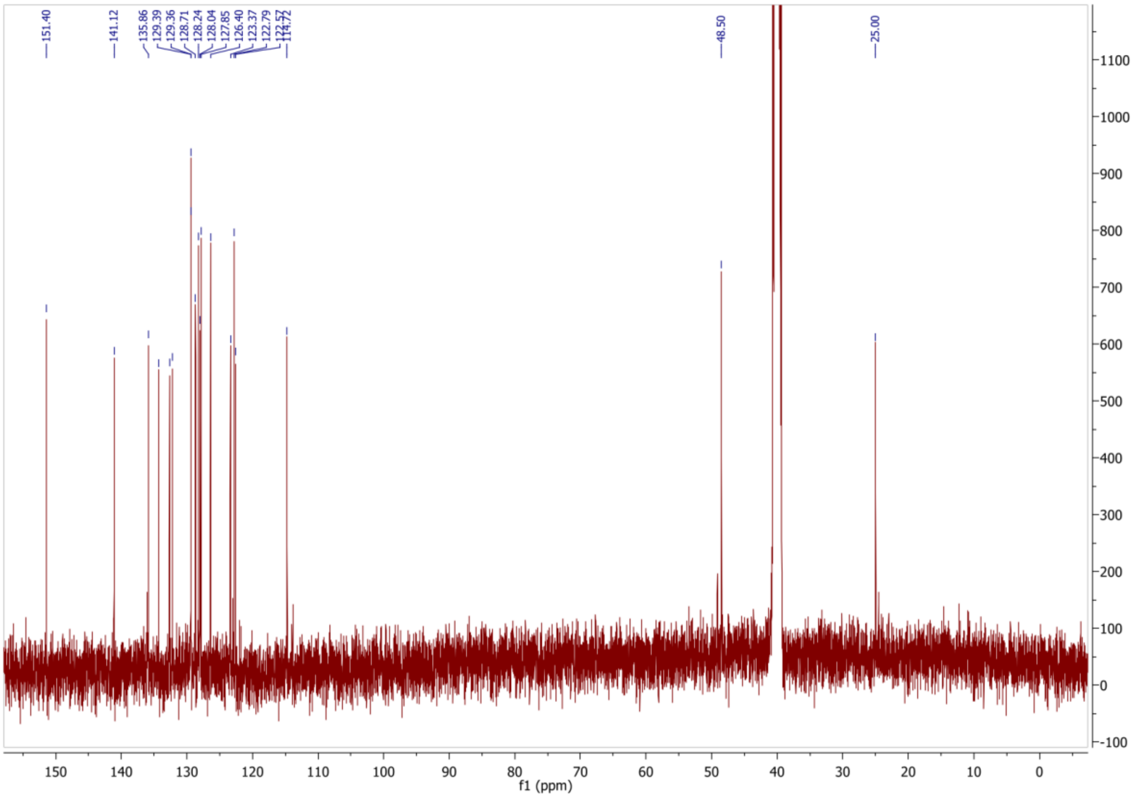

FT-IR:

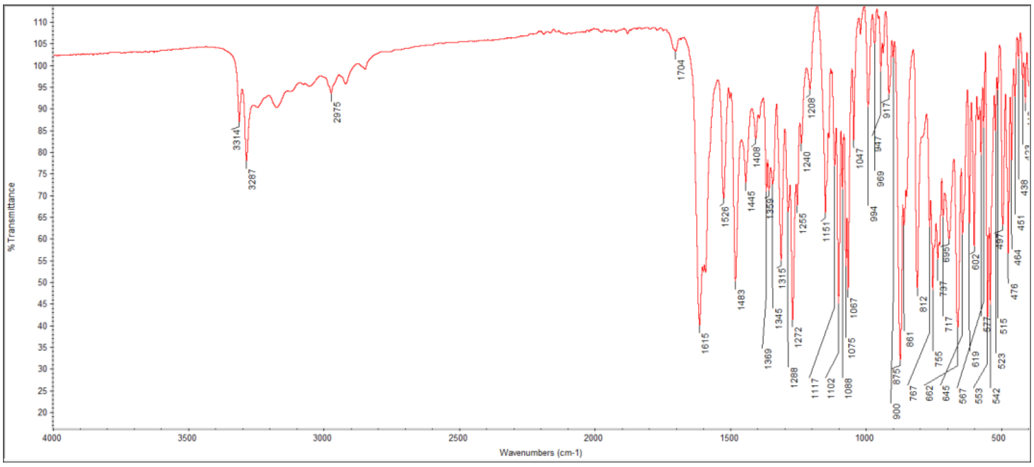

N-(4,5-dihydro-1*H*-1,3-benzodiazepin-2-yl)naphthalene-2-sulfonamide PR 38

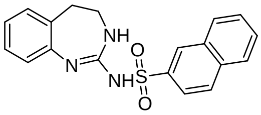

UPLC-MS:

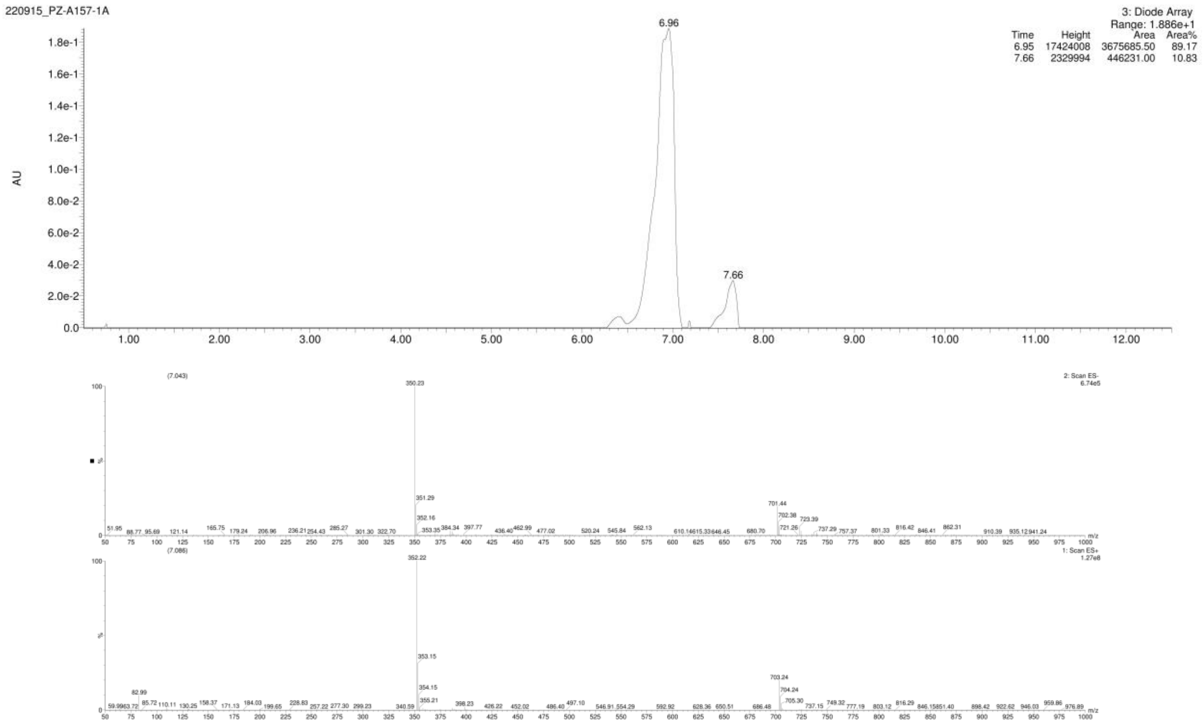

^{1}H NMR:

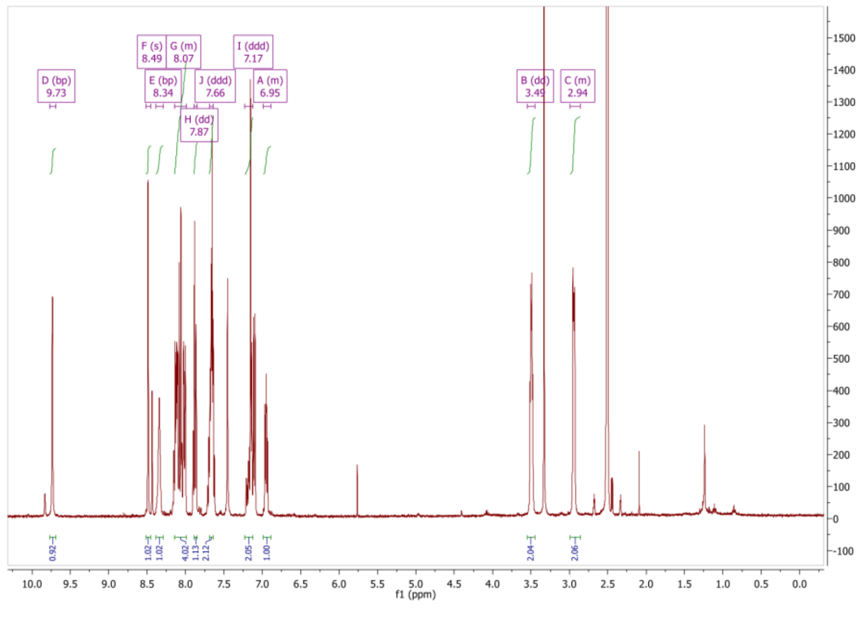

^{13}C NMR:

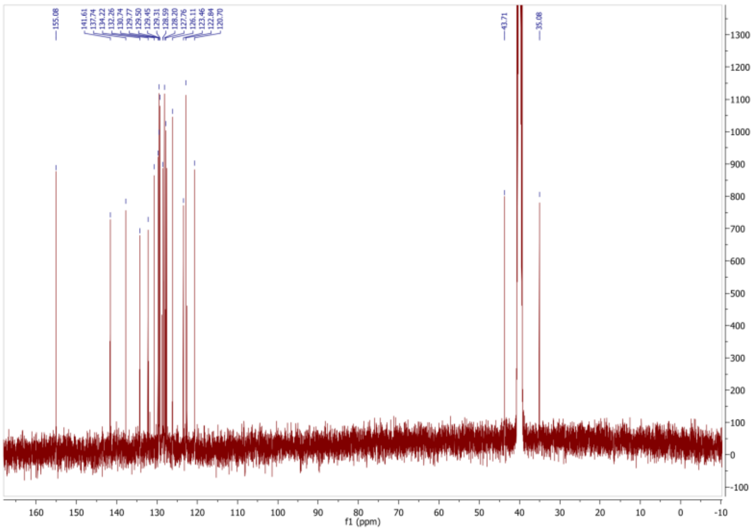

FT IR

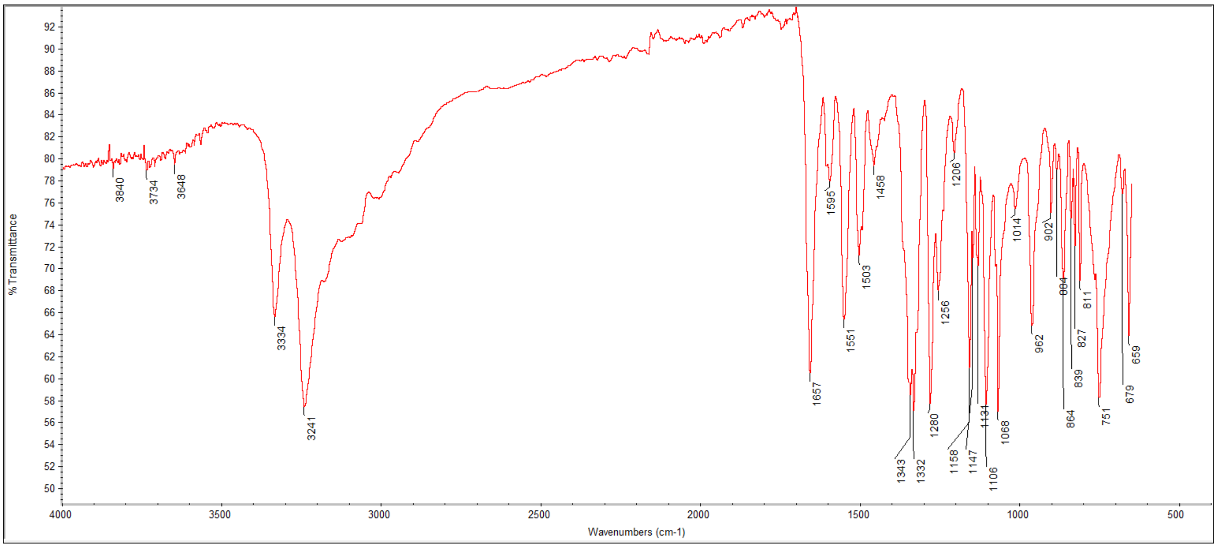

2-chloro-*N*-(3,4-dihydroquinazolin-2-yl)naphthalene-1-sulfonamide PR 41

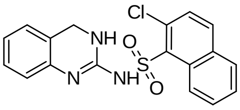

UPLC-MS:

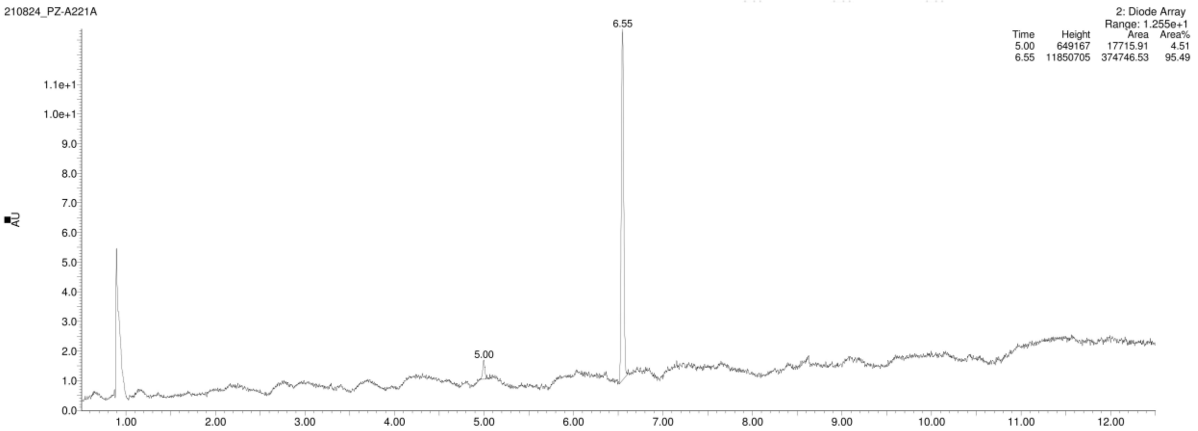

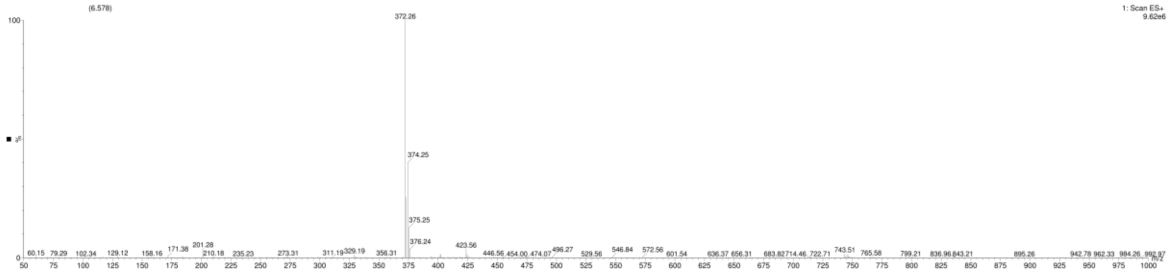

^{1}H NMR:

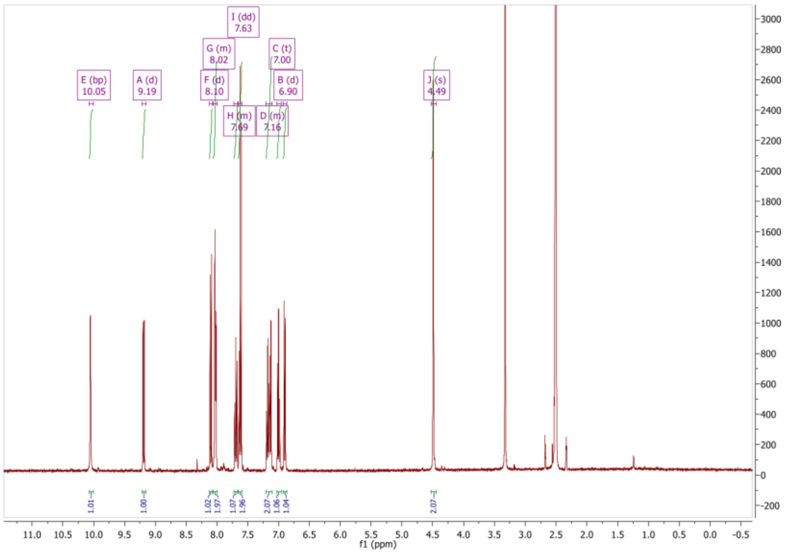

^{13}C NMR:

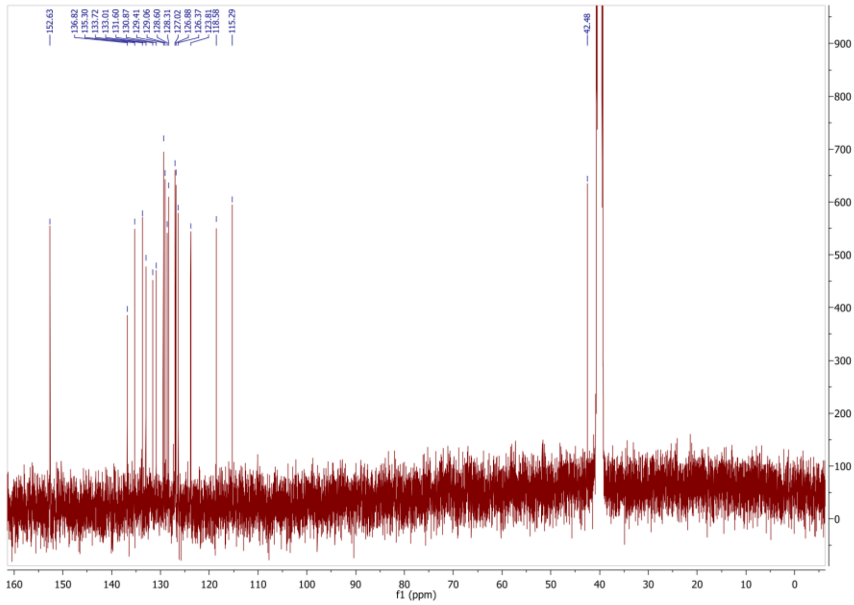

FT-IR:

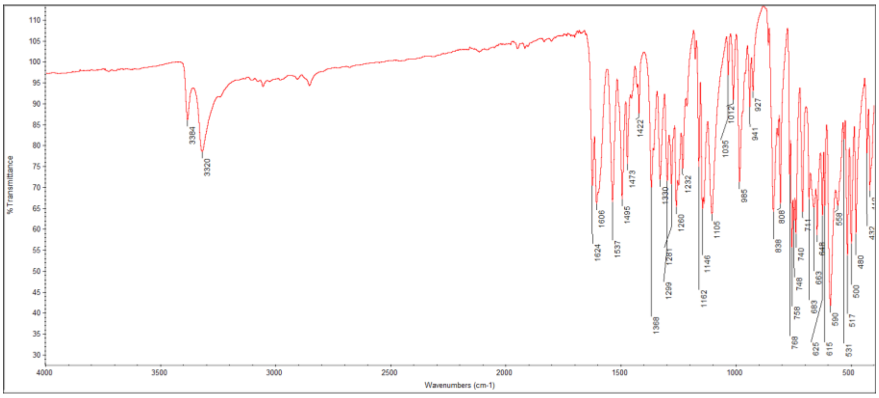

4-chloro-*N*-(3,4-dihydroquinazolin-2-yl)naphthalene-1-sulfonamide PR 42

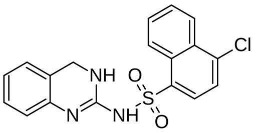

UPLC-MS:

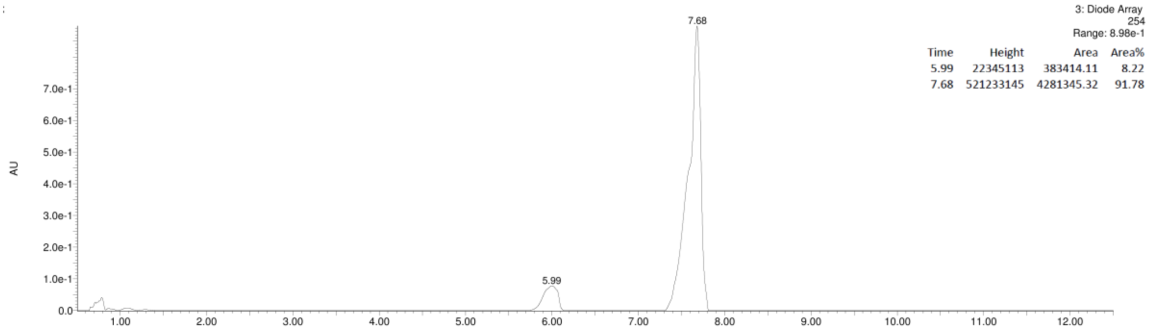

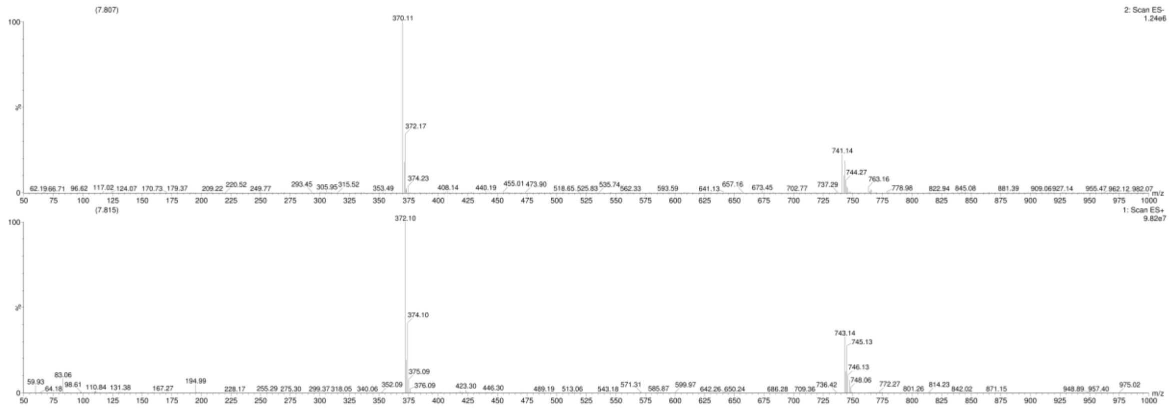

^{1}H NMR:

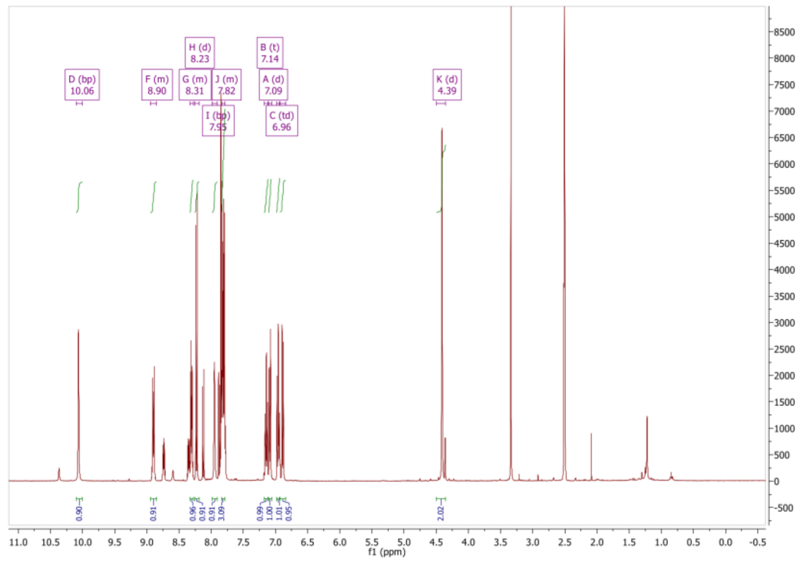

^{13}C NMR:

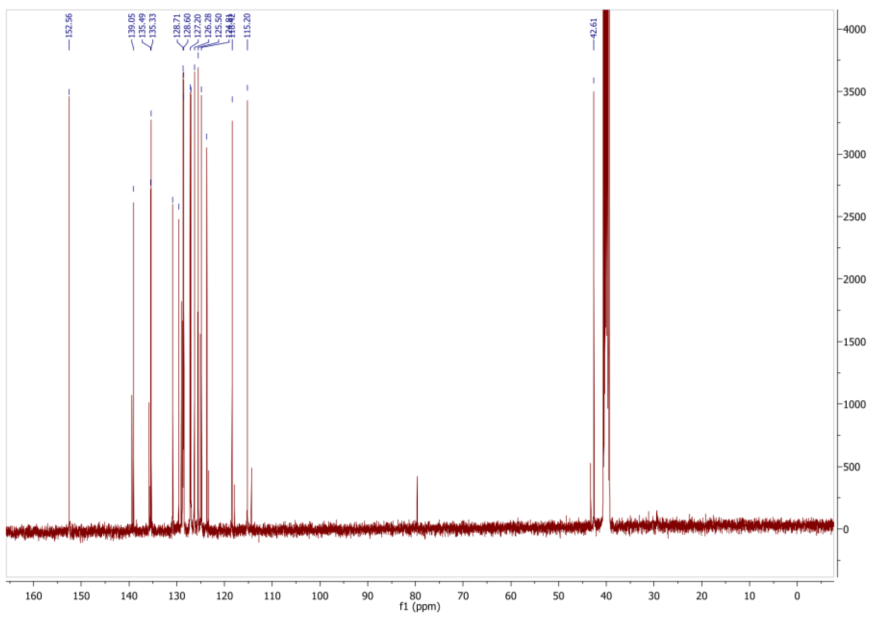

FT-IR:

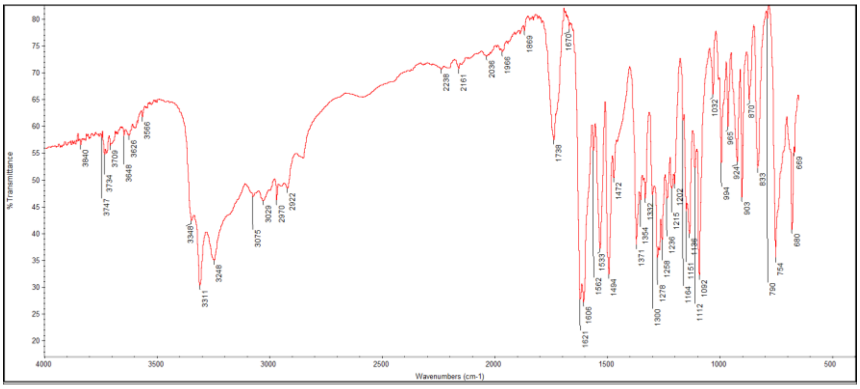

5-chloro-*N*-(3,4-dihydroquinazolin-2-yl)naphthalene-1-sulfonamide PR 43

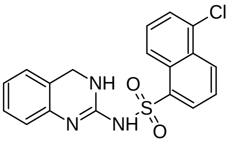

UPLC-MS:

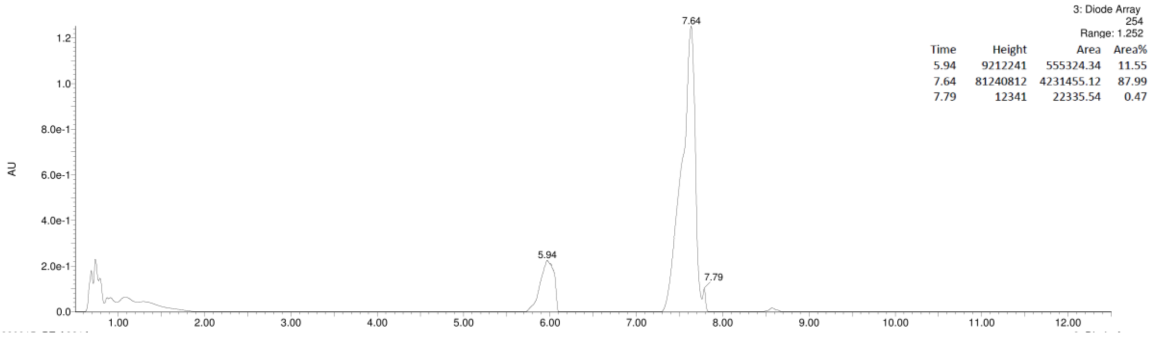

Time	Height	Area	Area%
5.94	9212241	555324.34	11.55
7.64	81240812	4231455.12	87.99
7.79	12341	22335.54	0.47

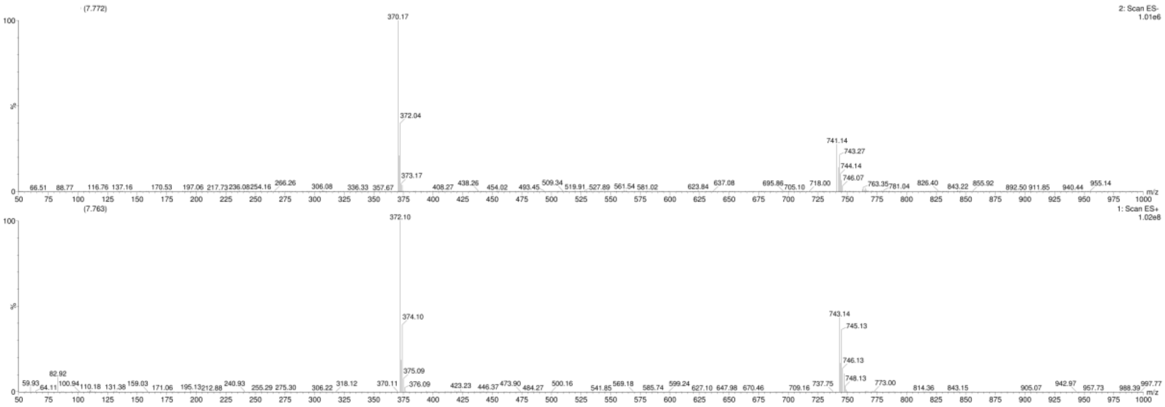

^{1}H NMR:

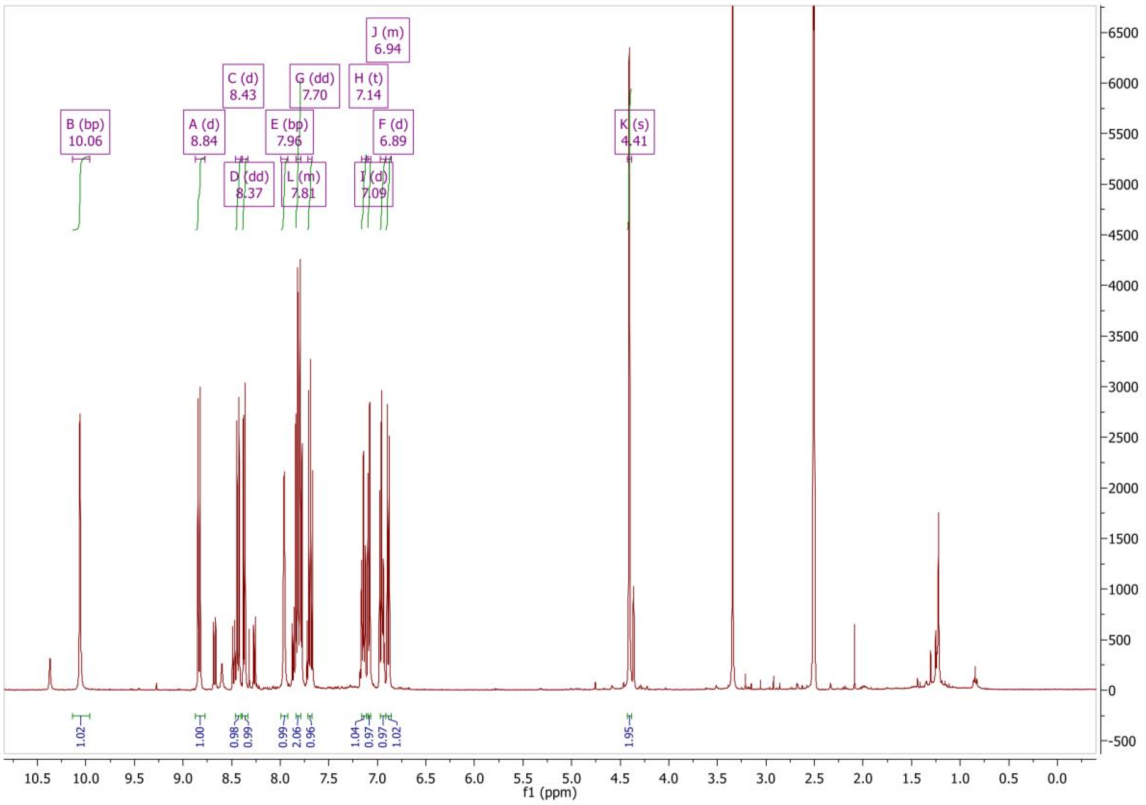

^{13}C NMR:

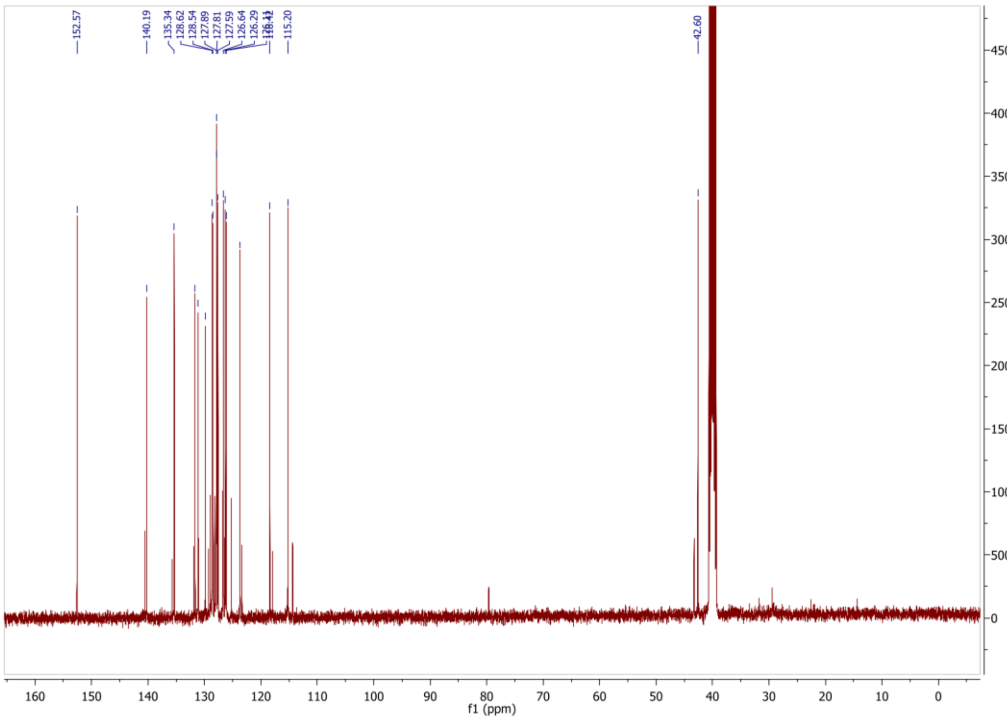

FT-IR:

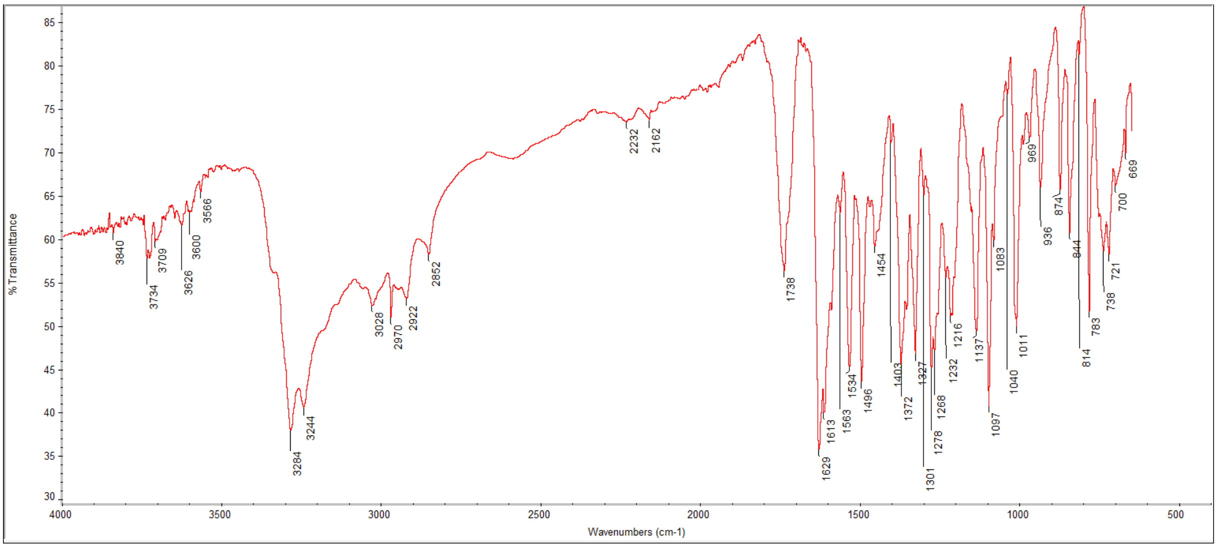

6-chloro-*N*-(3,4-dihydroquinazolin-2-yl)naphthalene-1-sulfonamide PR 44

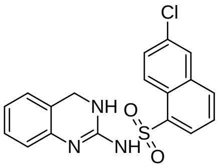

UPLC-MS:

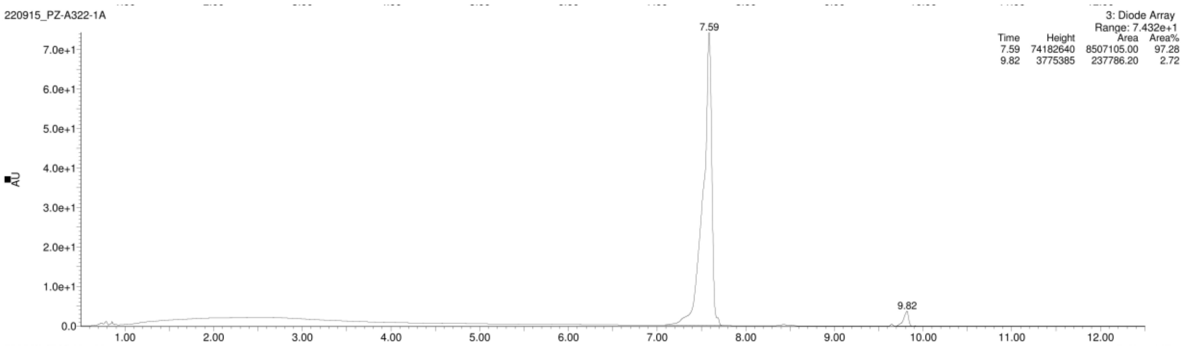

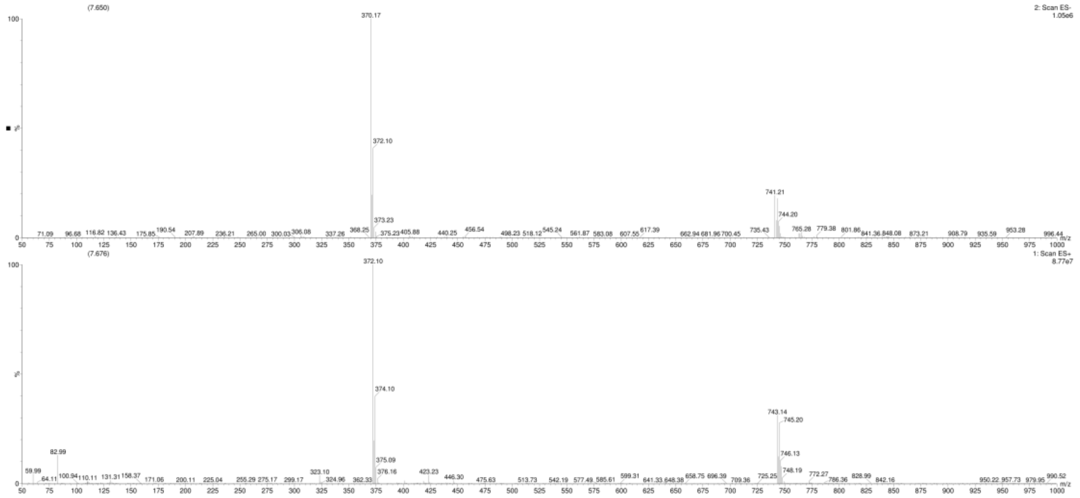
2: Scan ES-
1.05e6
370.17
372.10
373.23
741.21
744.20
1: Scan ES+
8.77e7
372.10
374.10
375.09
376.16
82.99
743.14
745.20
746.13
748.19
m/z

^{1}H NMR:

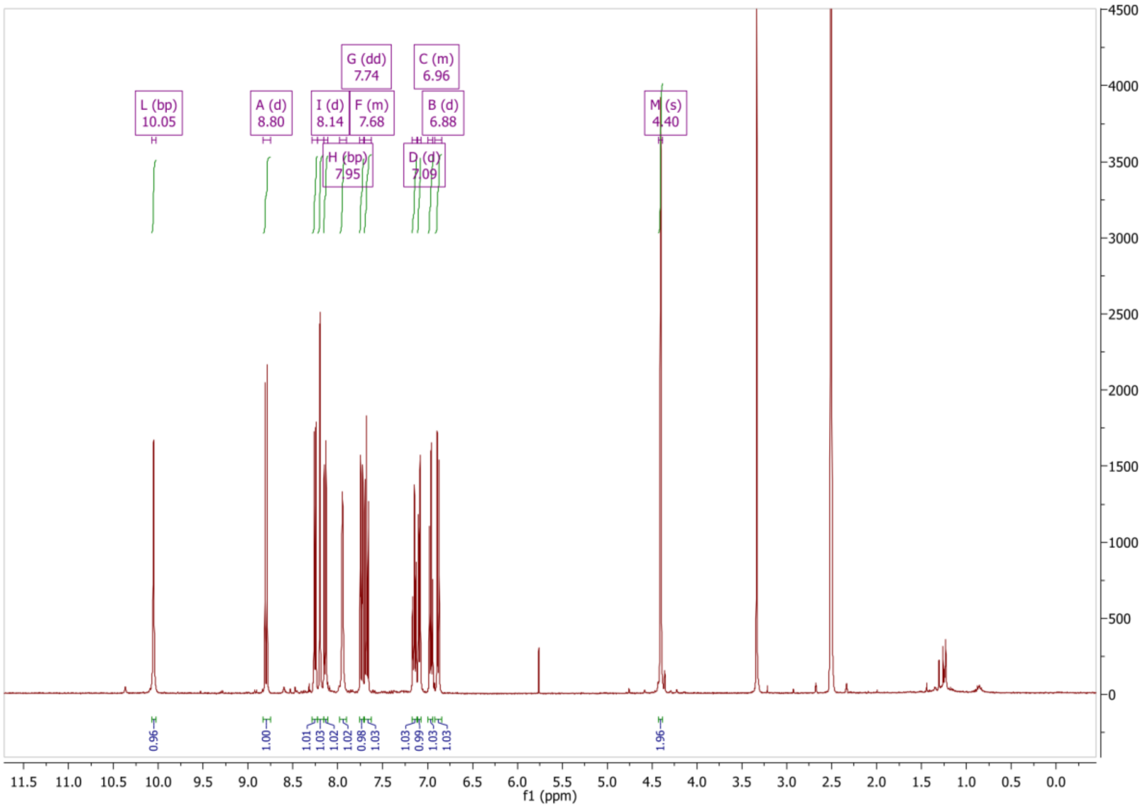
L (bp)
10.05
A (d)
8.80
I (d)
8.14
G (dd)
7.74
F (m)
7.68
H (bp)
7.95
C (m)
6.96
B (d)
6.88
D (d)
7.09
M (s)
4.40
0.96
1.00
1.01
1.03
1.02
1.02
0.98
1.03
1.03
0.99
1.03
1.03
1.96
11.5
11.0
10.5
10.0
9.5
9.0
8.5
8.0
7.5
7.0
6.5
6.0
5.5
5.0
4.5
4.0
3.5
3.0
2.5
2.0
1.5
1.0
0.5
0.0
f1 (ppm)
4500
4000
3500
3000
2500
2000
1500
1000
500
0

^{13}C NMR:

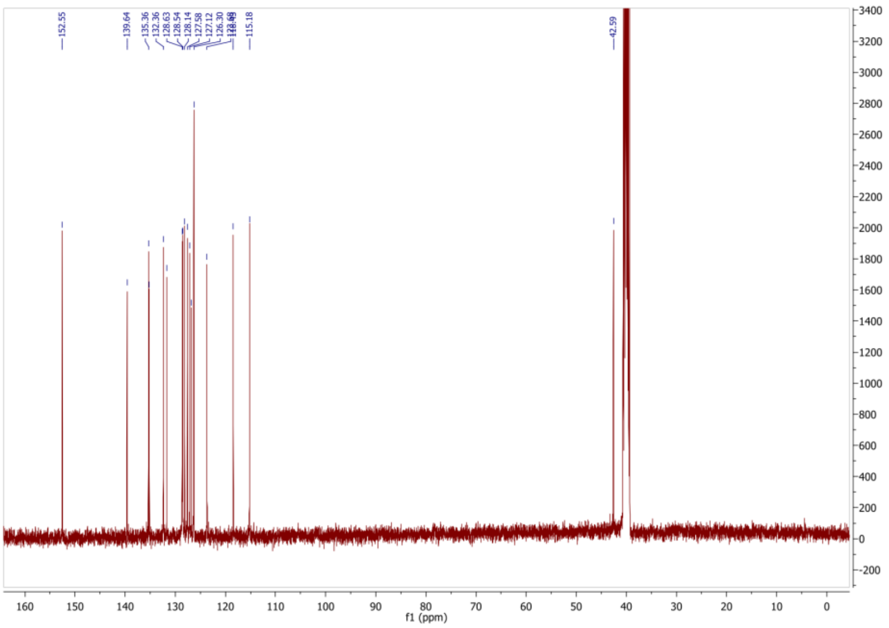

FT-IR:

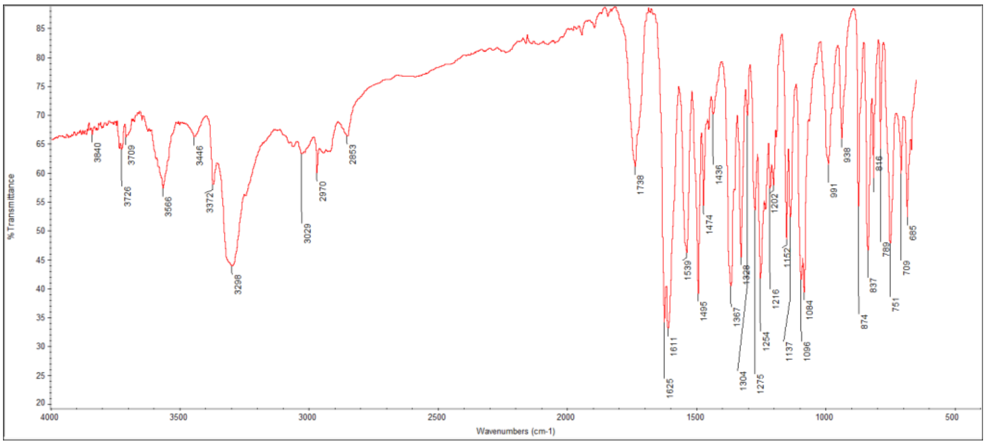

8-chloro-*N*-(3,4-dihydroquinazolin-2-yl)naphthalene-1-sulfonamide PR 45

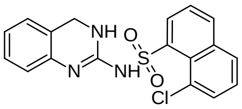

UPLC-MS:

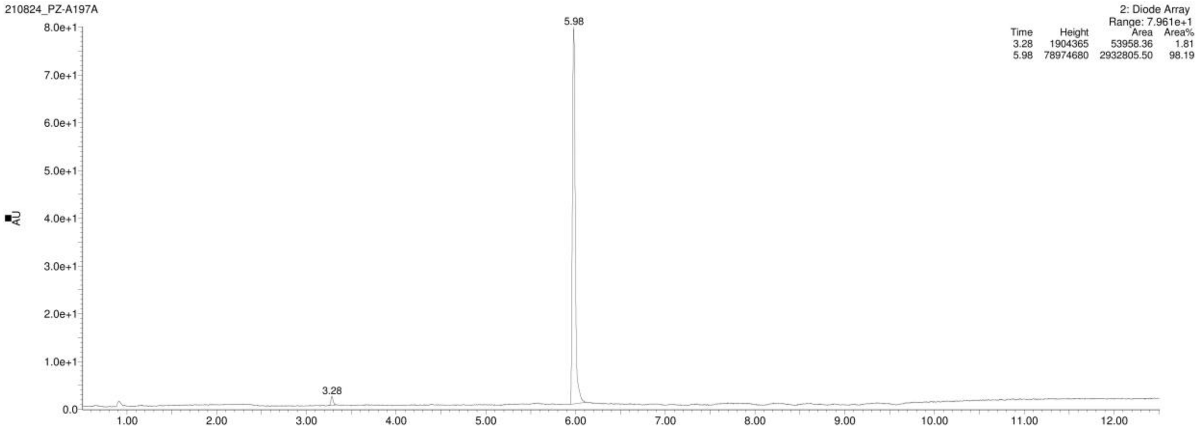

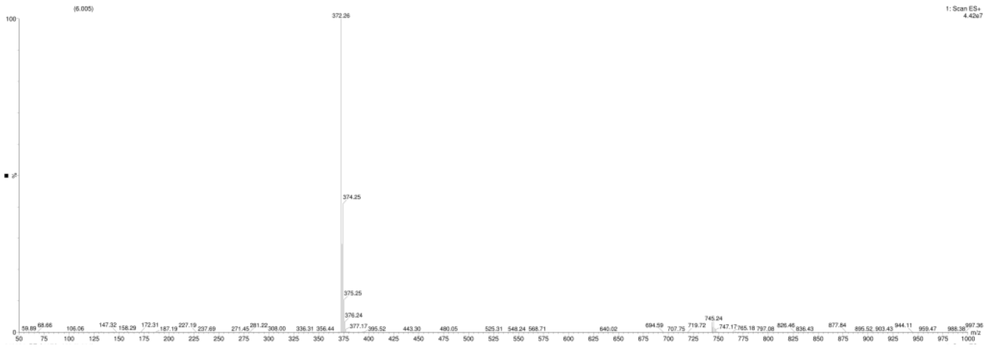

^{1}H NMR:

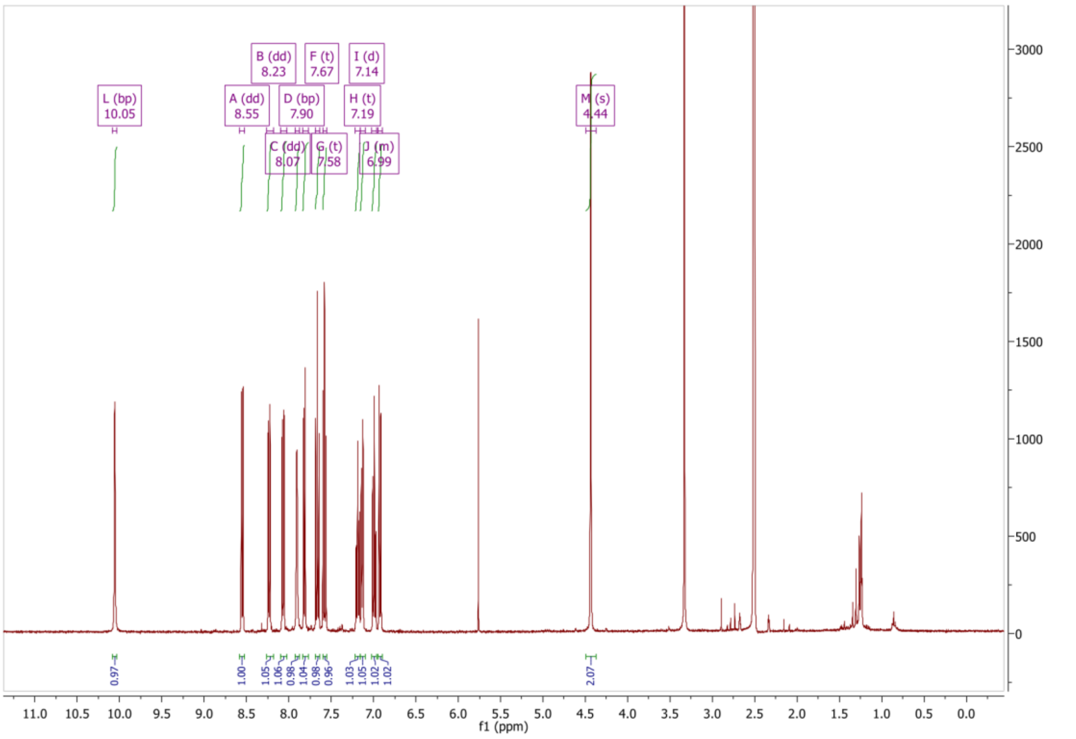

^{13}C NMR:

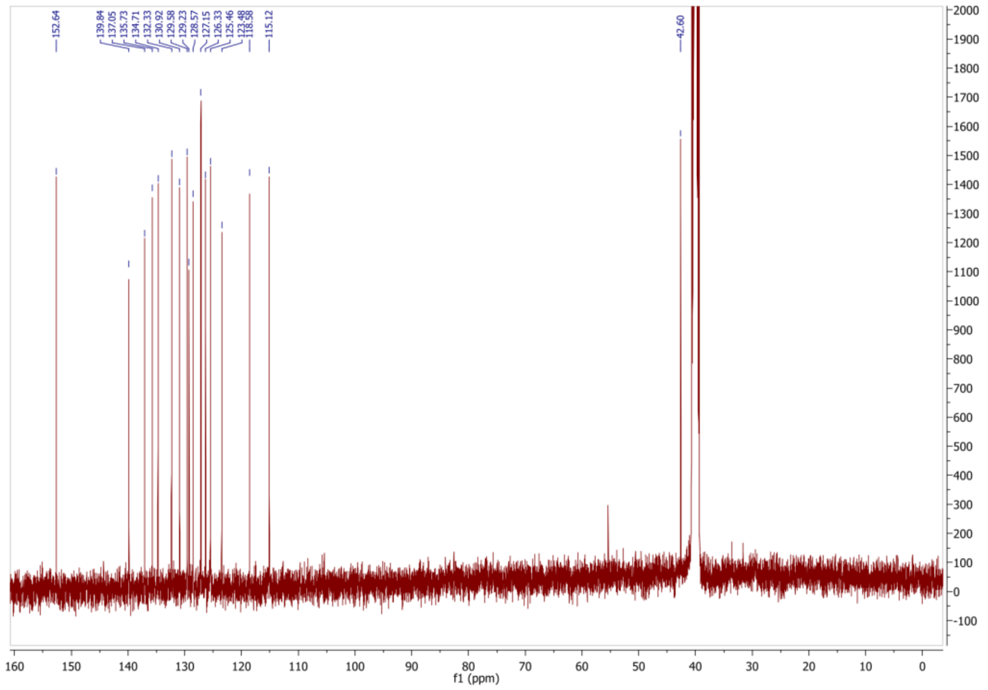

FT-IR:

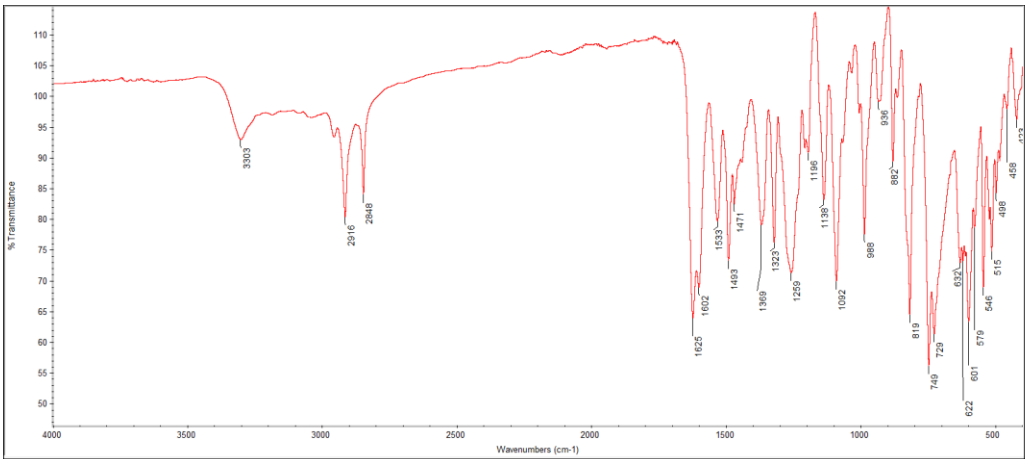

N-(5-chloro-3,4-dihydroquinazolin-2-yl)naphthalene-1-sulfonamide PR 46

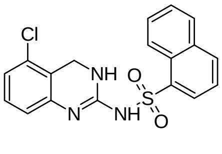

UPLC-MS:

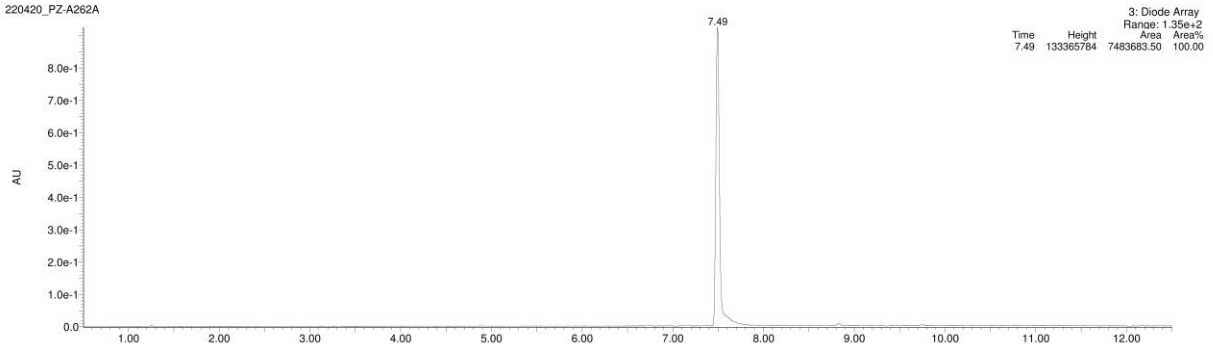

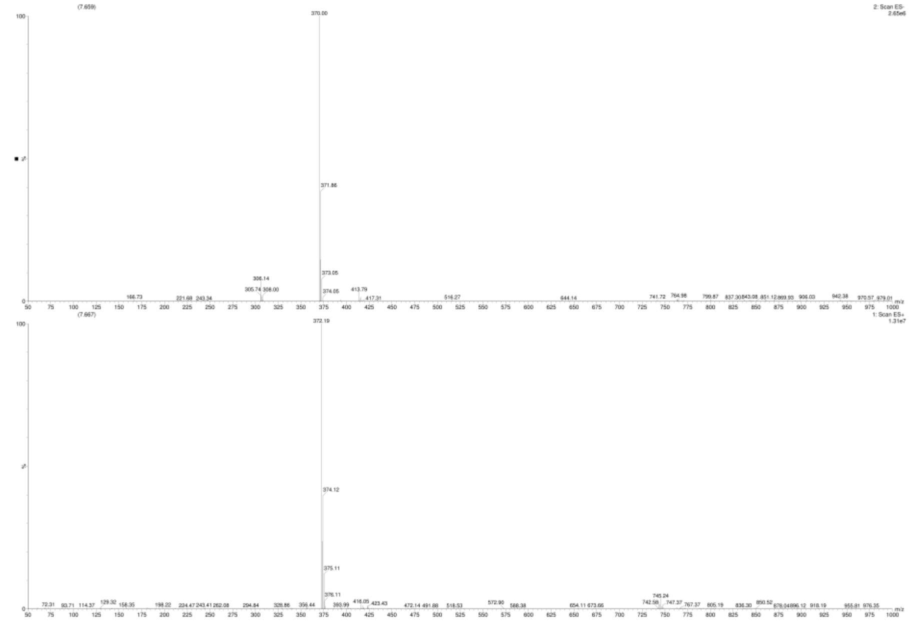

^{1}H NMR:

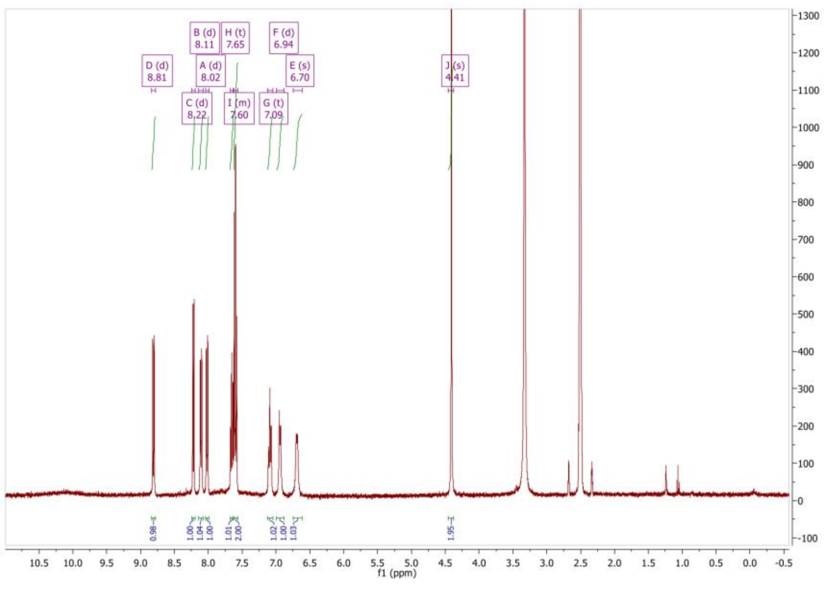

^{13}C NMR:

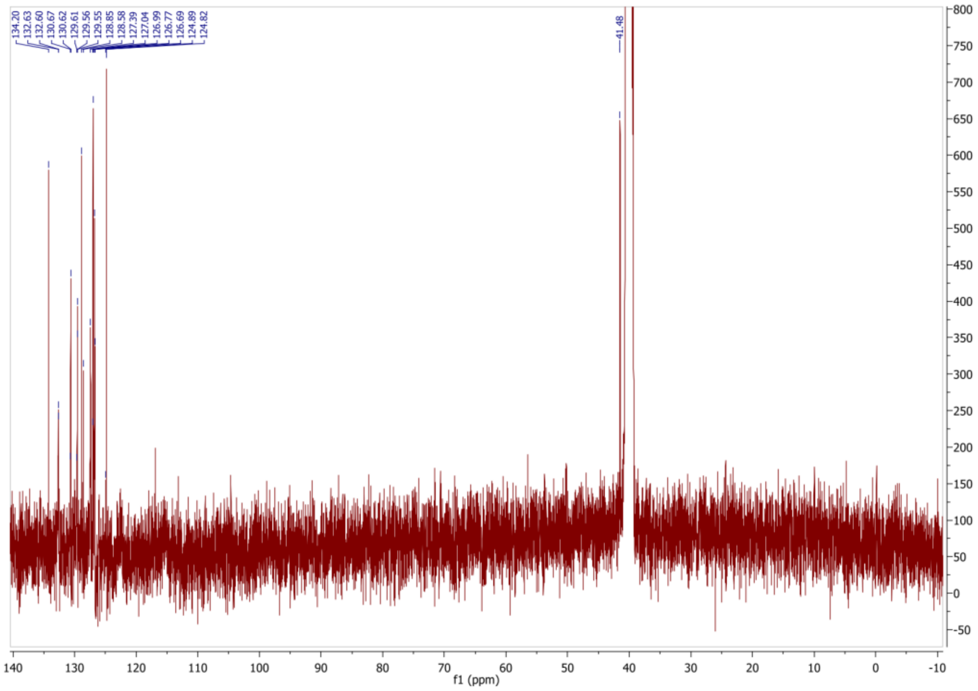

FT-IR:

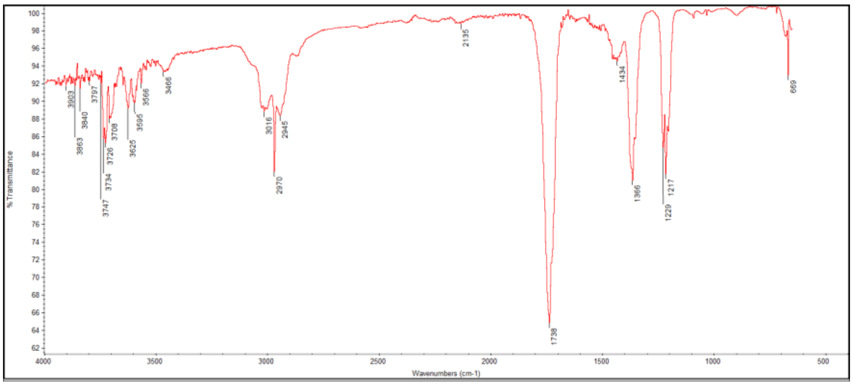

N-(6-chloro-3,4-dihydroquinazolin-2-yl)naphthalene-1-sulfonamide PR 47

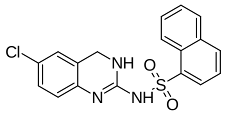

UPLC-MS:

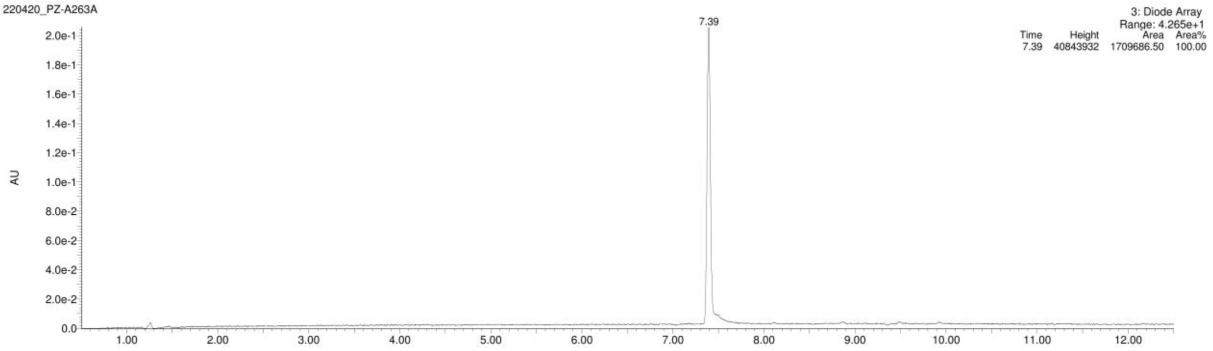

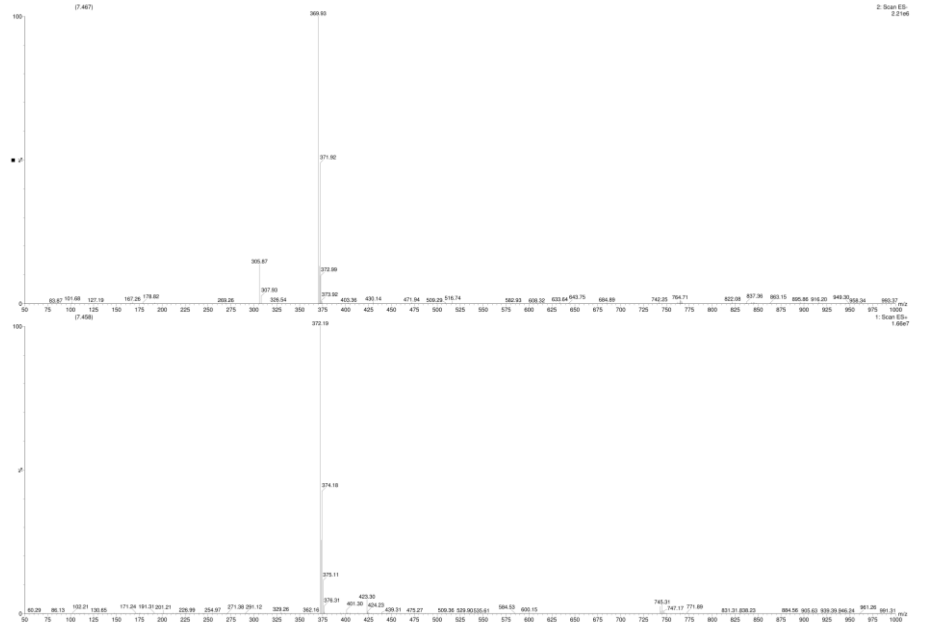

^{1}H NMR:

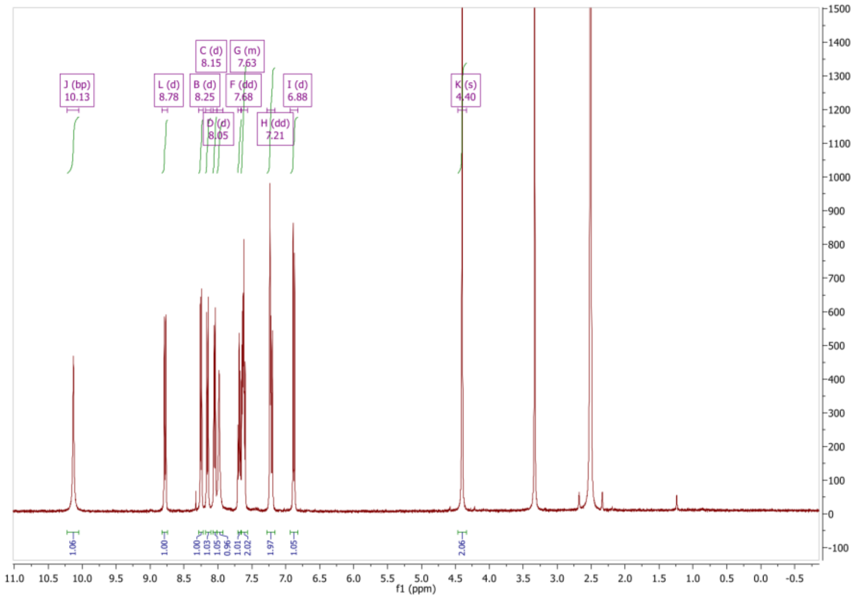

^{13}C NMR:

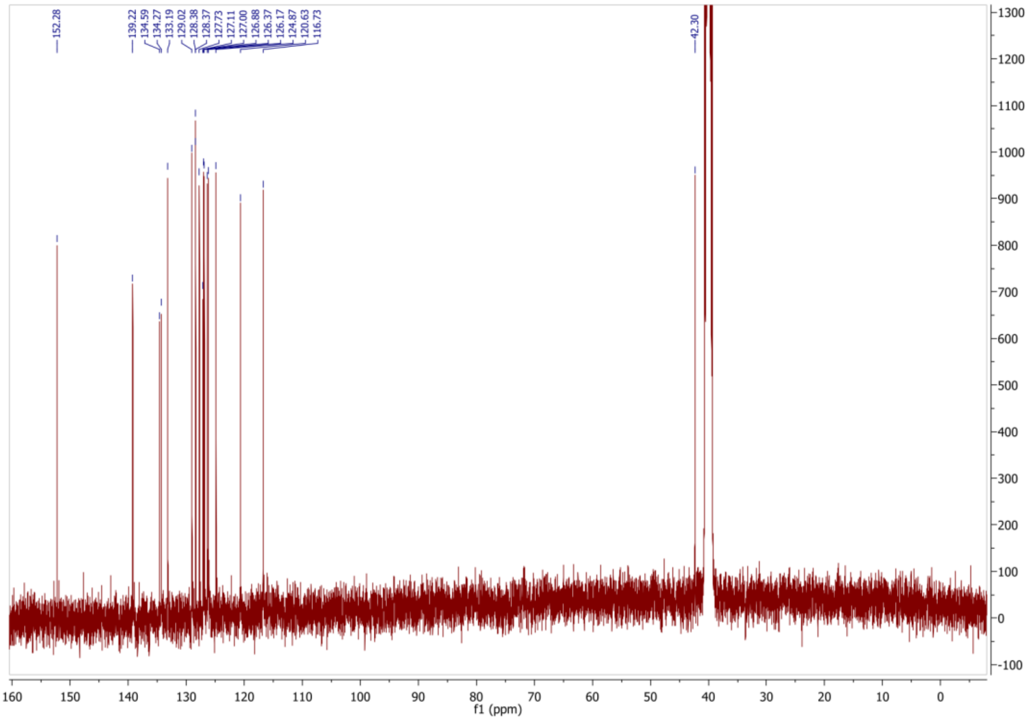

FT-IR:

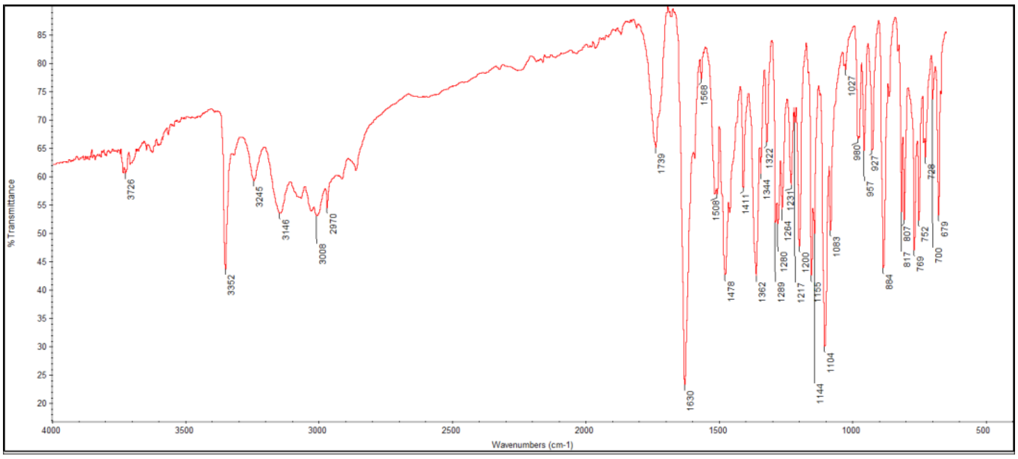

N-(6-chloro-3,4-dihydroquinazolin-2-yl)naphthalene-1-sulfonamide PR 48

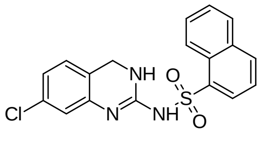

UPLC-MS:

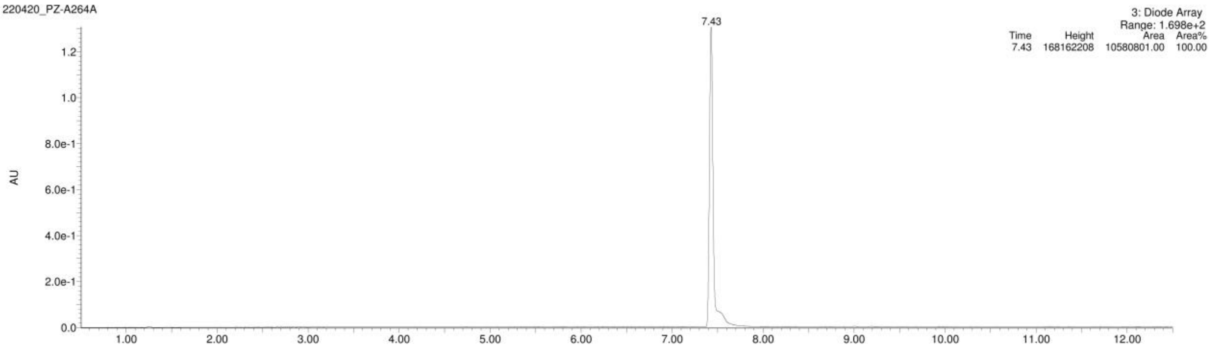

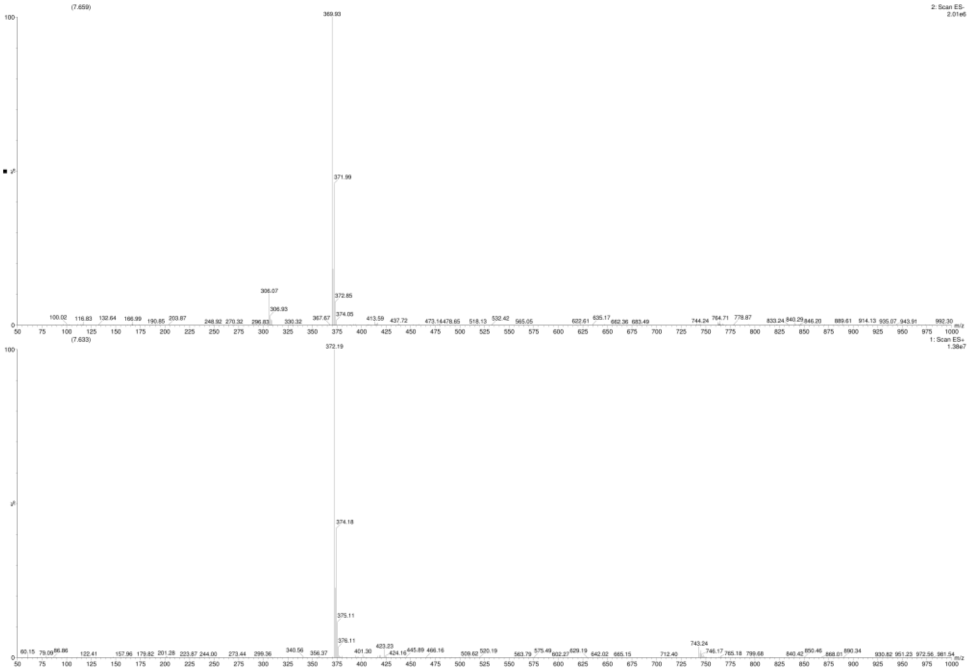

^{1}H NMR:

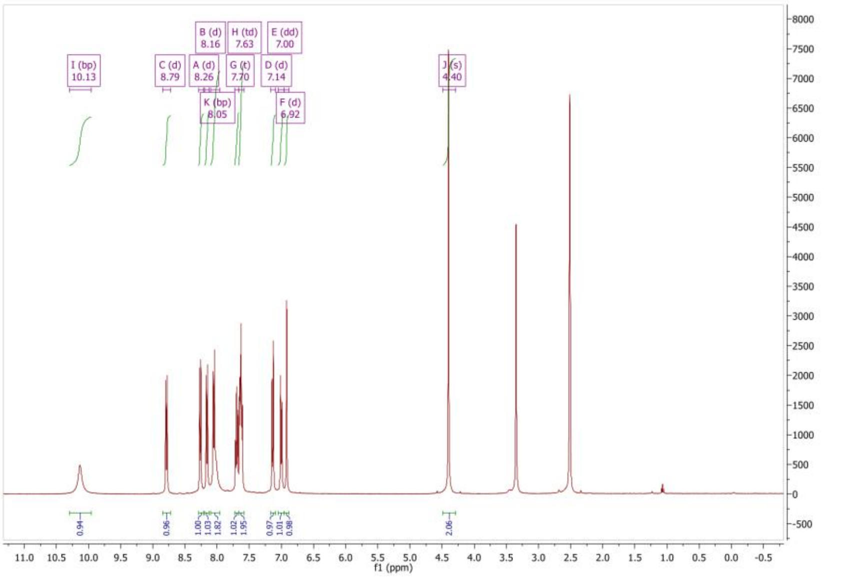

^{13}C NMR:

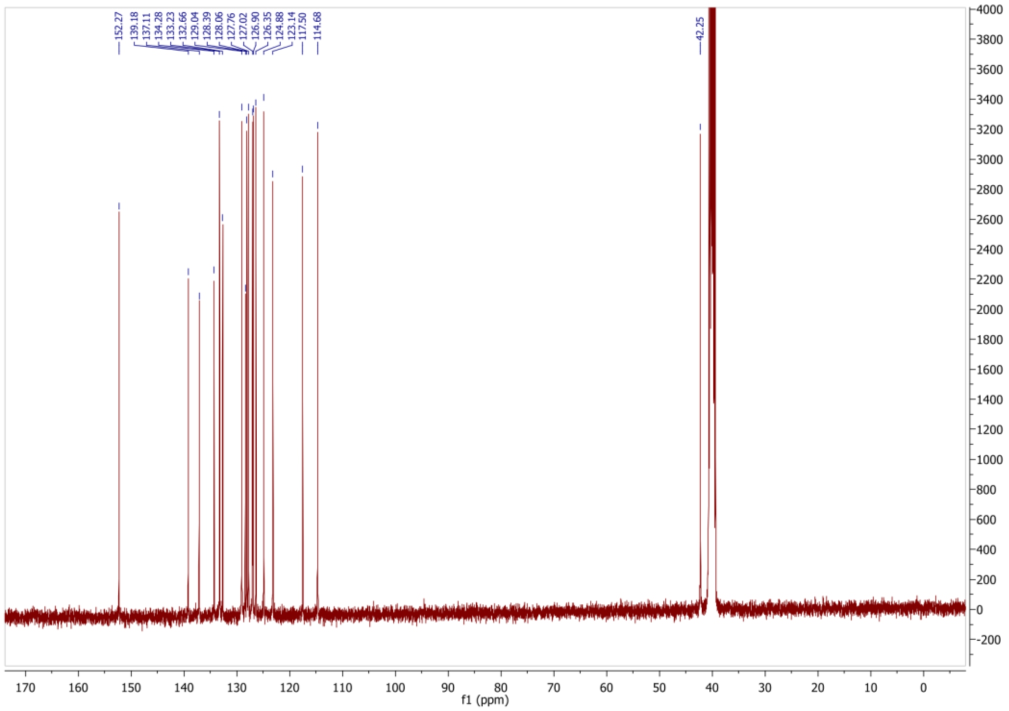

FT-IR:

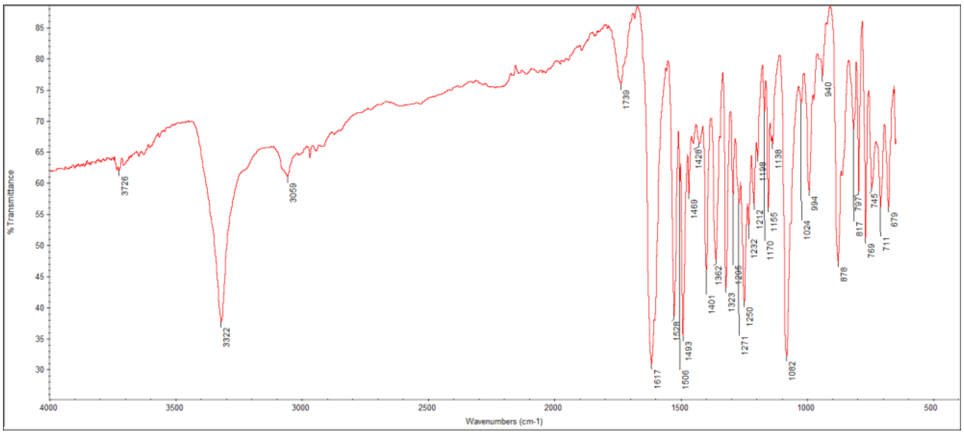

N-(8-chloro-3,4-dihydroquinazolin-2-yl)naphthalene-1-sulfonamide PR 49

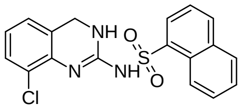

UPLC-MS:

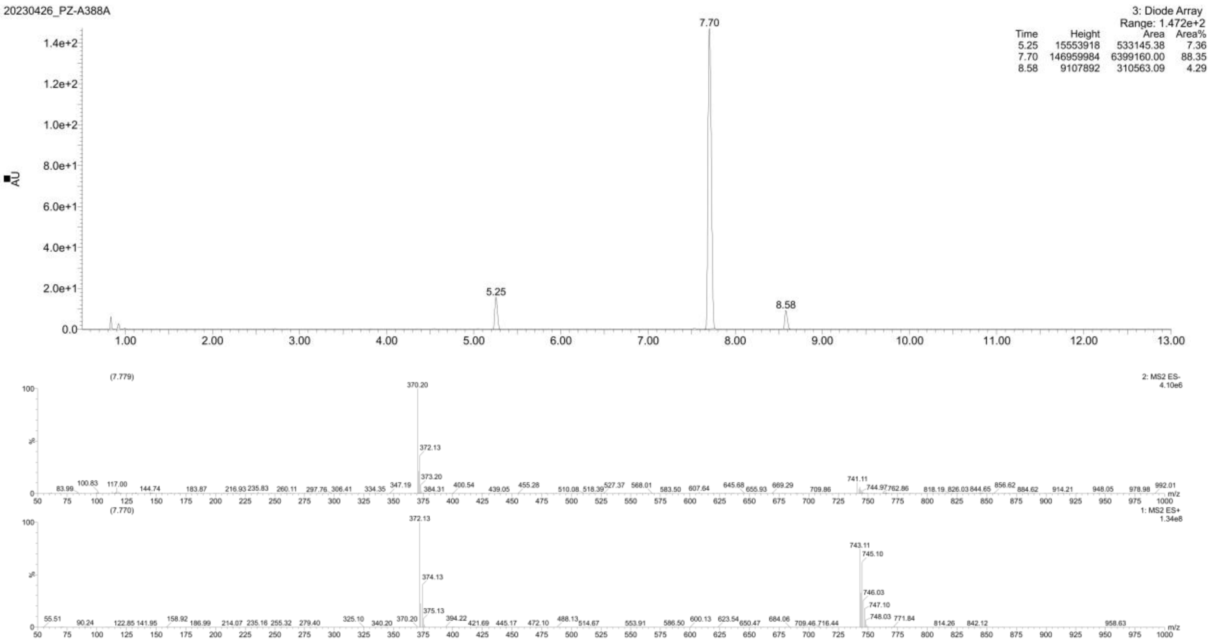

^{1}H NMR:

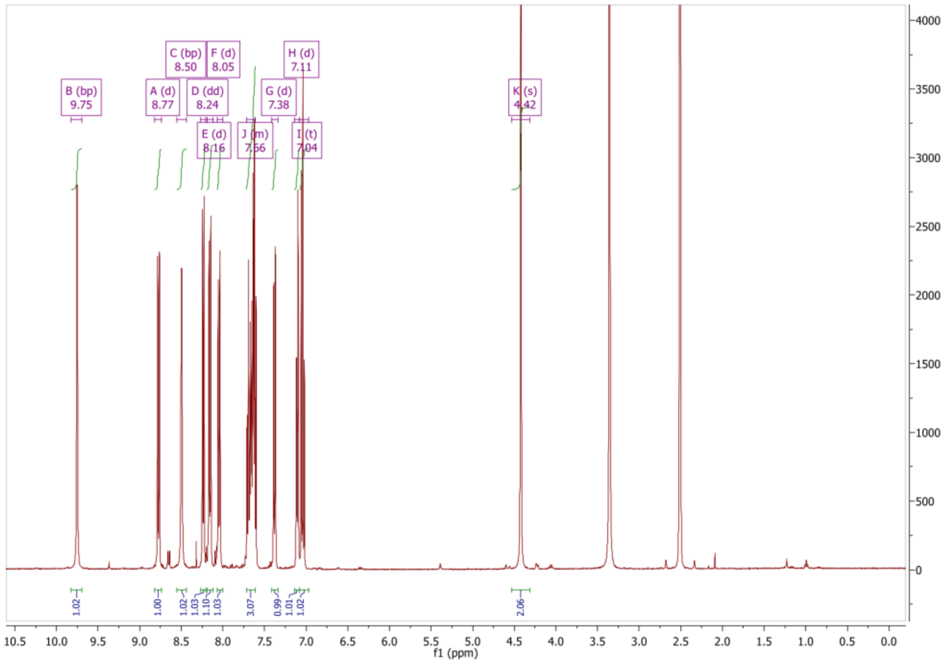

^{13}C NMR:

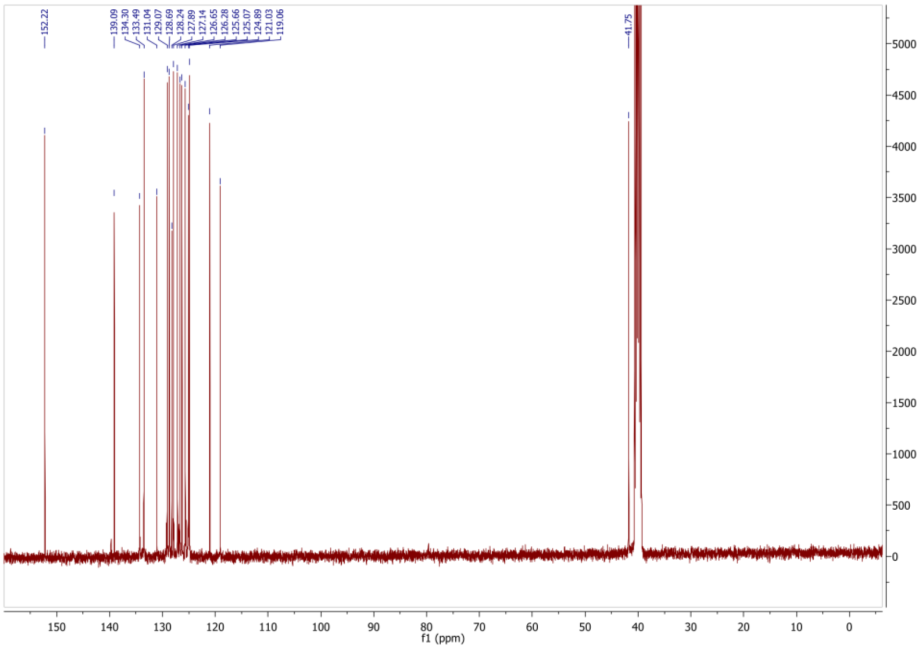

FT-IR:

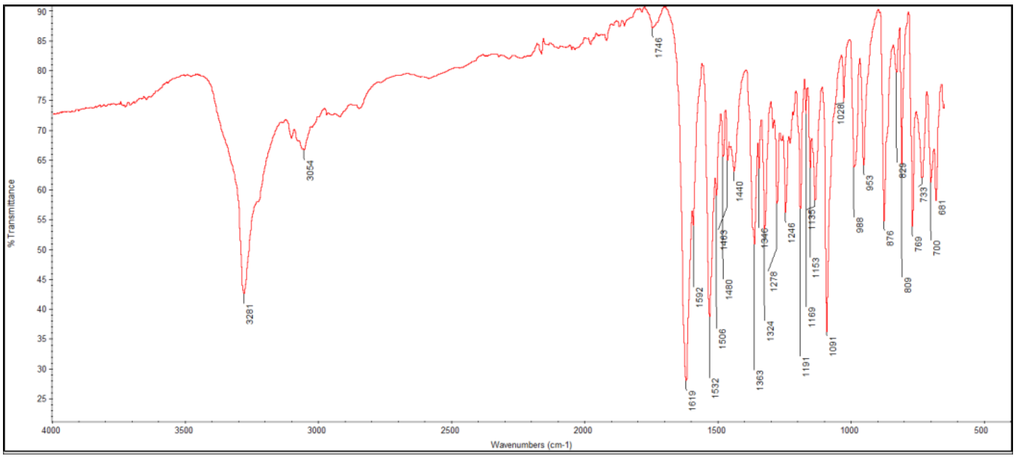

N-(5,6-dichloro-3,4-dihydroquinazolin-2-yl)naphthalene-1-sulfonamide PR 50

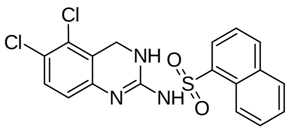

UPLC-MS:

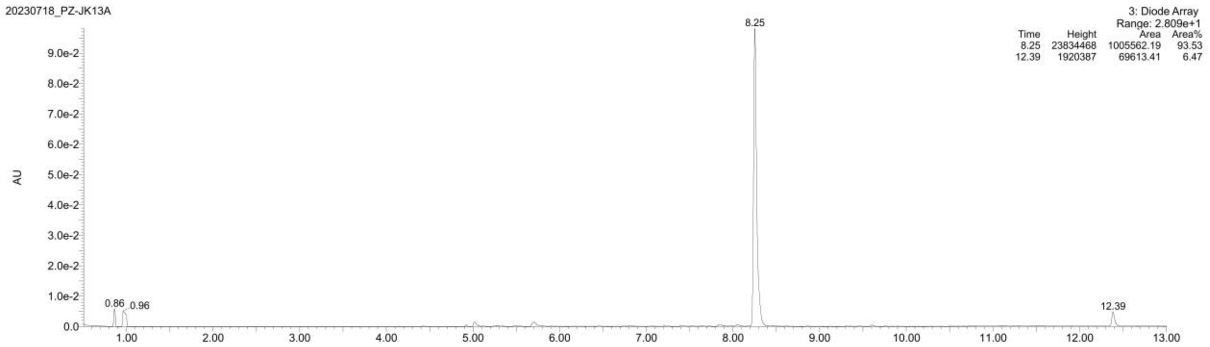

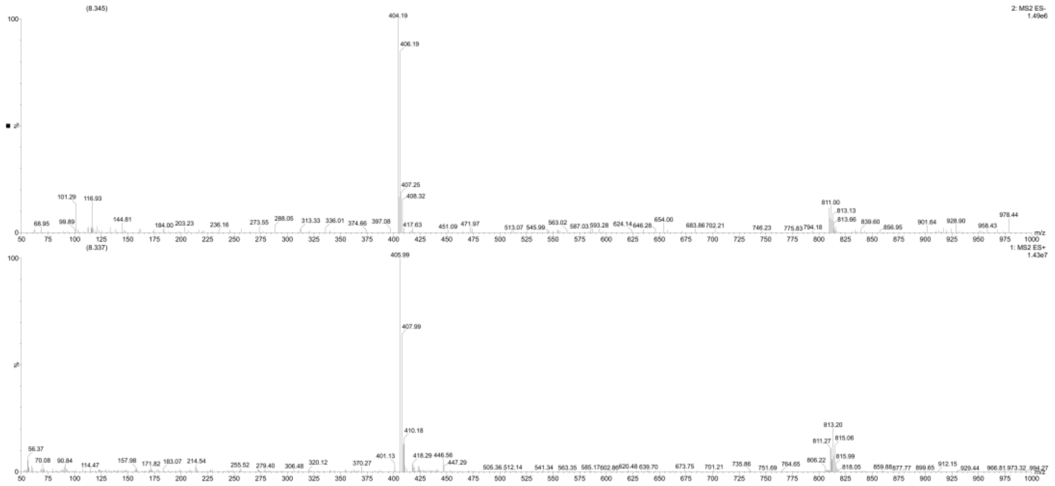

FT-IR:

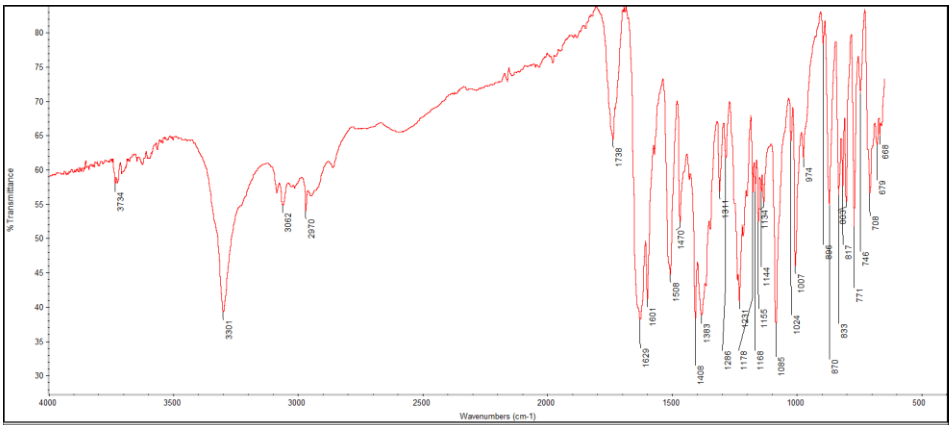

N-(6,8-dichloro-3,4-dihydroquinazolin-2-yl)naphthalene-1-sulfonamide PR 51

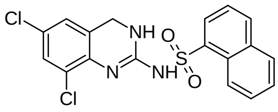

UPLC-MS:

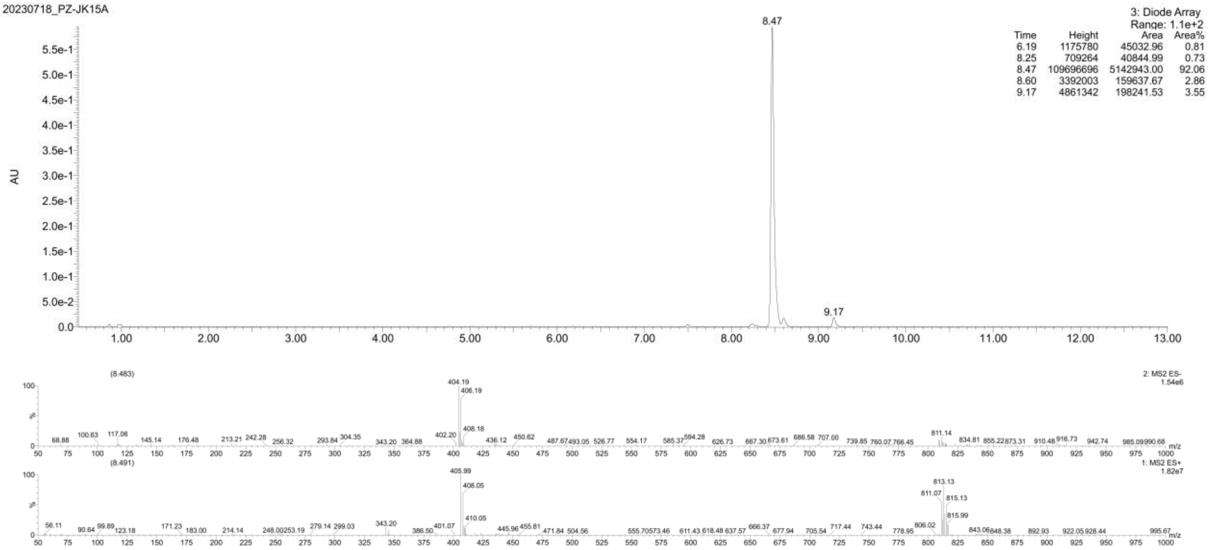

^{1}H NMR:

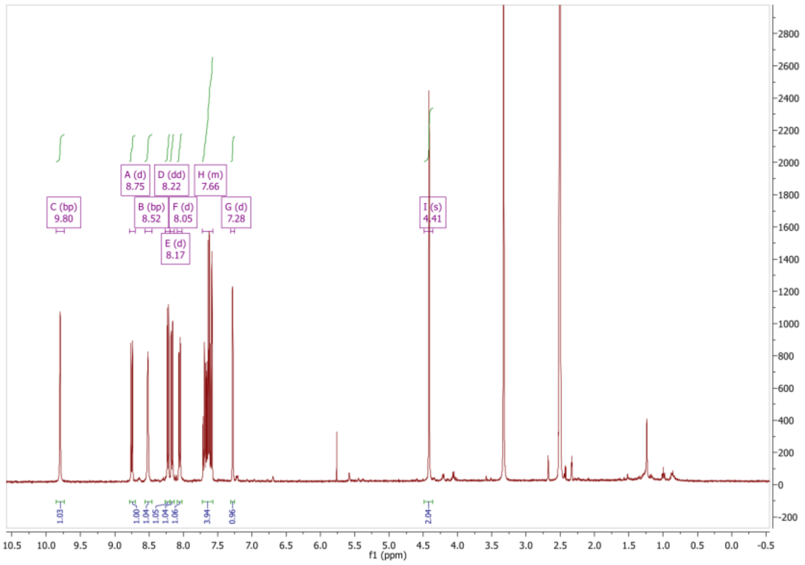

^{13}C NMR:

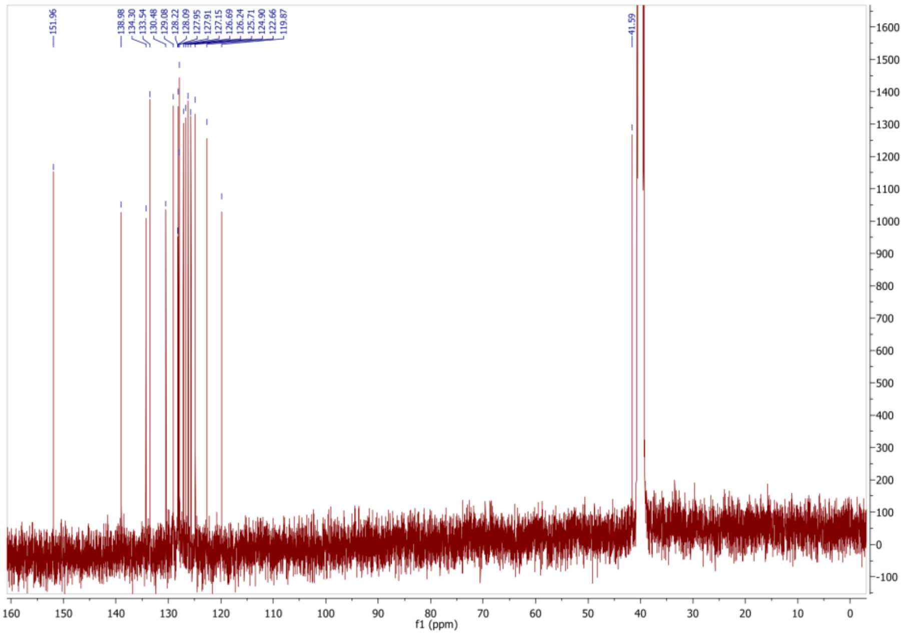

FT-IR:

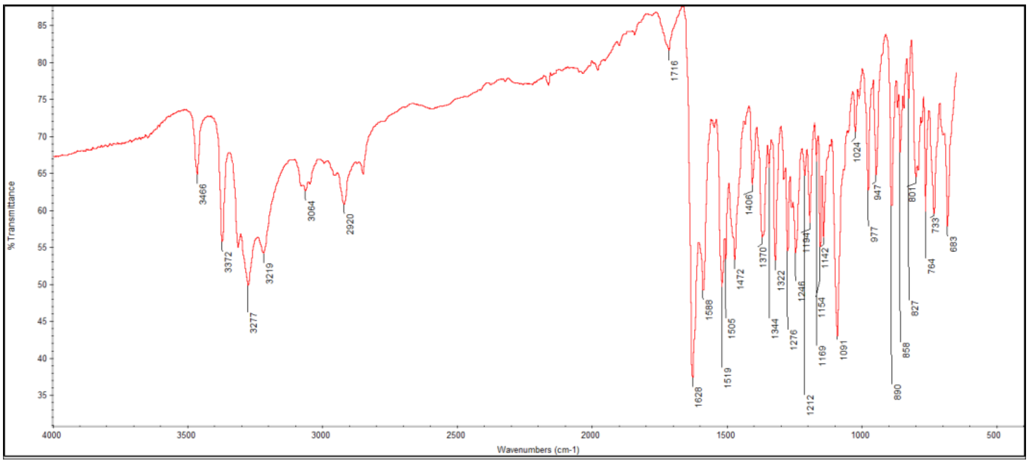

2-chloro-*N*-(5-chloro-3,4-dihydroquinazolin-2-yl)naphthalene-1-sulfonamide PR 52

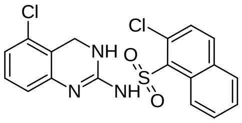

UPLC-MS:

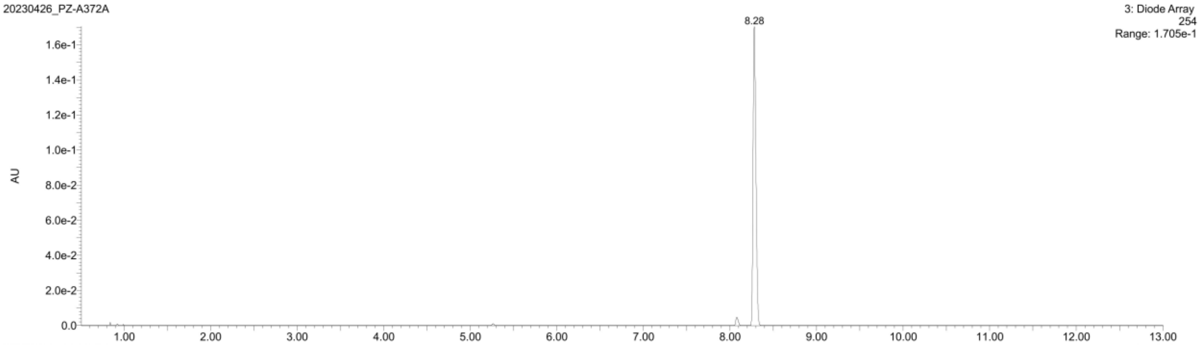

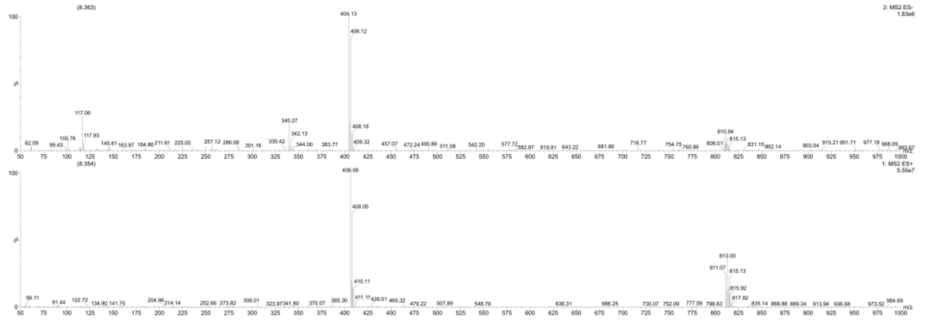

^{1}H NMR:

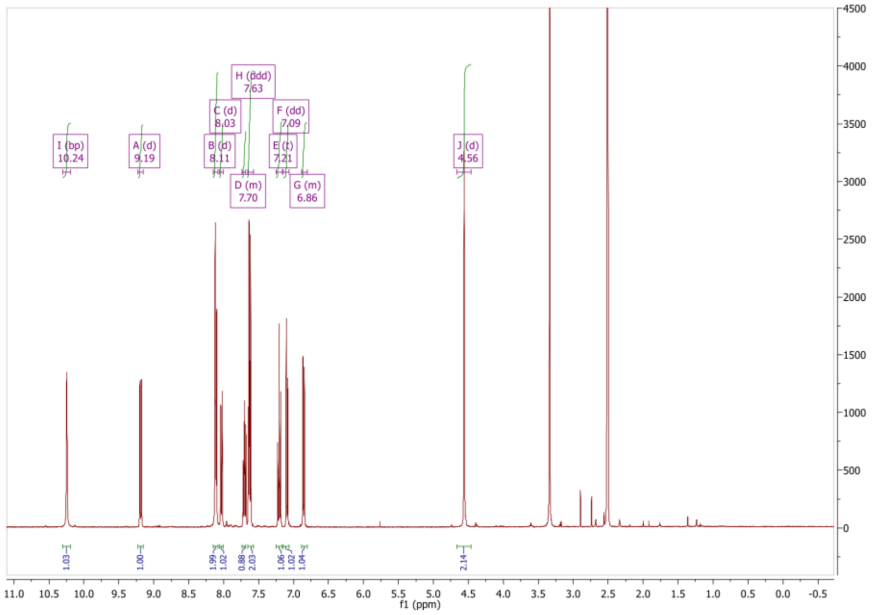

^{13}C NMR:

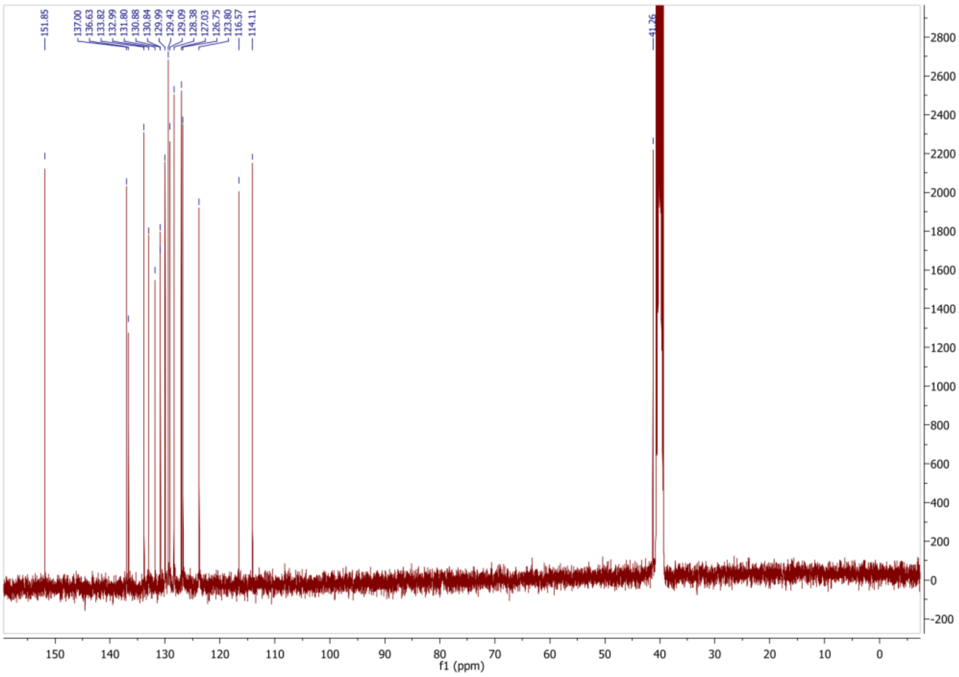

FT-IR:

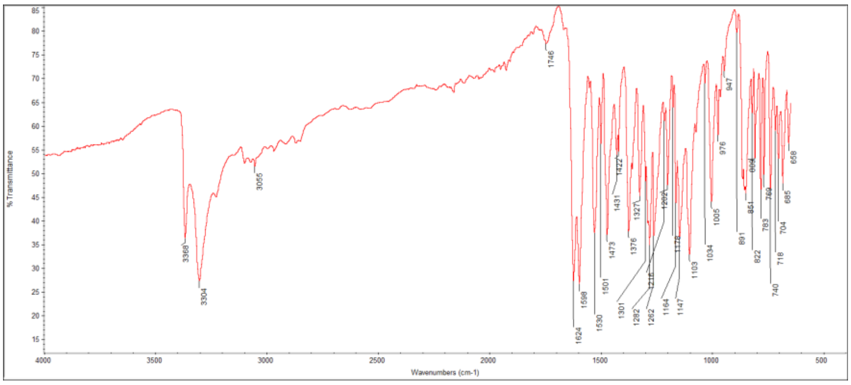

4-chloro-*N*-(5-chloro-3,4-dihydroquinazolin-2-yl)naphthalene-1-sulfonamide PR 53

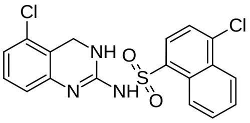

UPLC-MS:

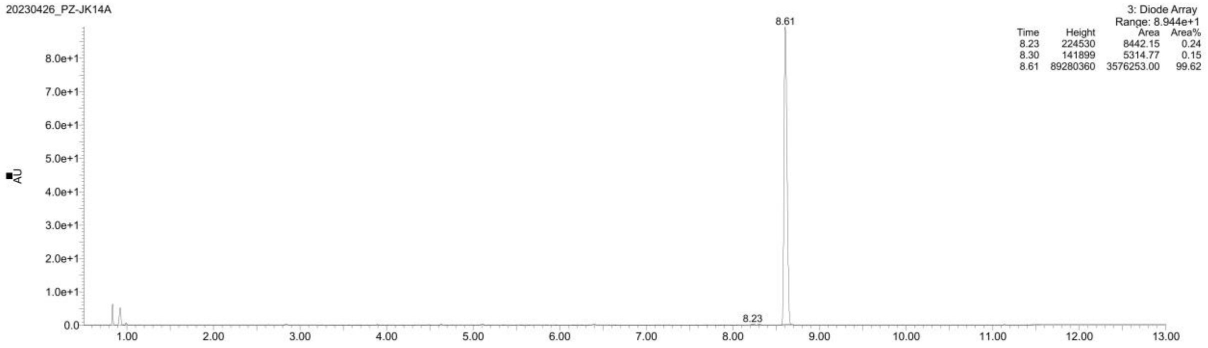

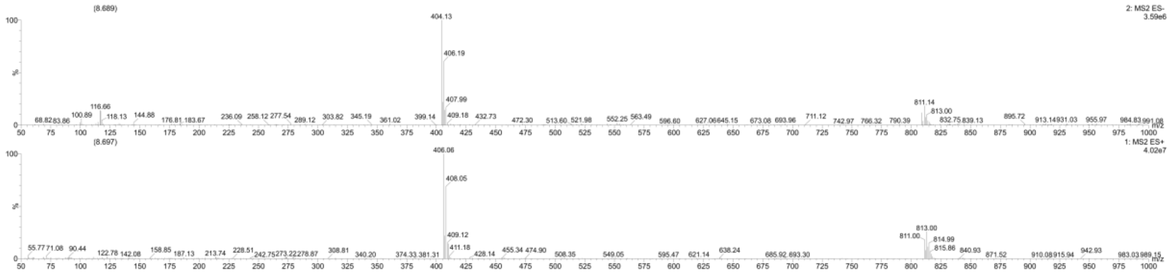

^{1}H NMR:

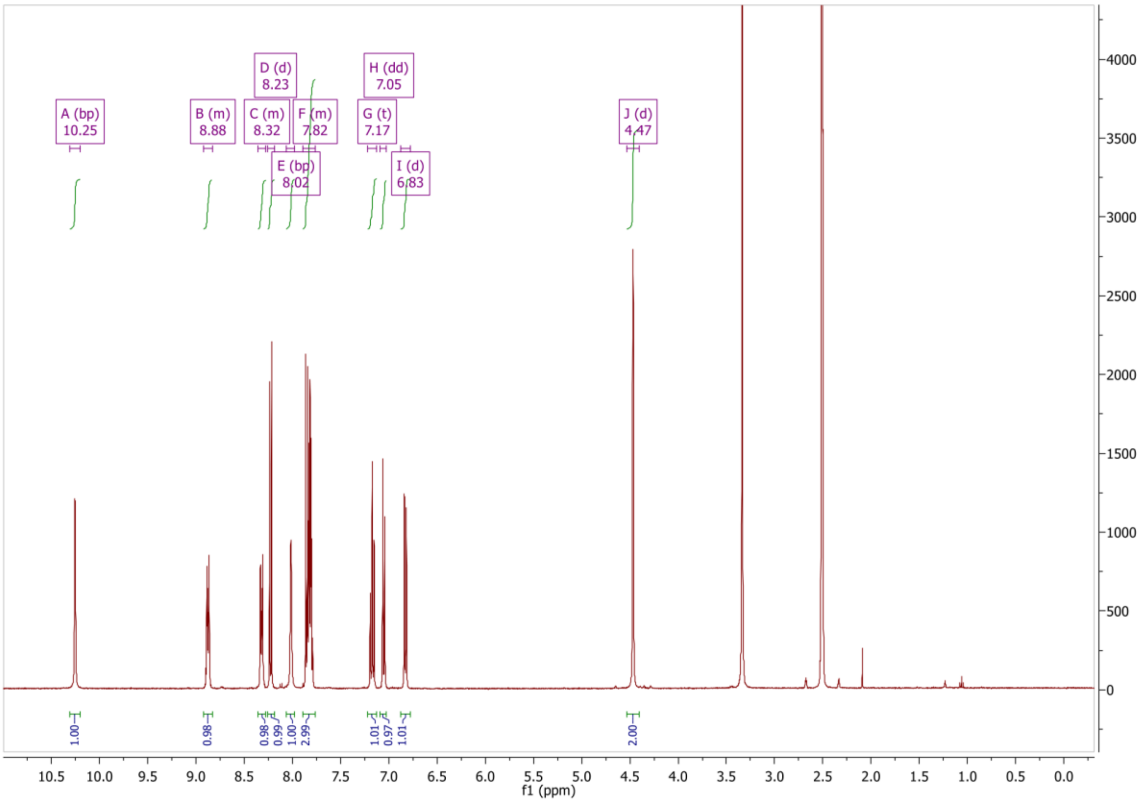
A (bp)
10.25
B (m)
8.88
C (m)
8.32
D (d)
8.23
E (bp)
8.02
F (m)
7.82
G (t)
7.17
H (dd)
7.05
I (d)
6.83
J (d)
4.47
1.00
0.98
0.98
0.99
1.00
2.99
1.01
0.97
1.01
2.00
f1 (ppm)

^{13}C NMR:

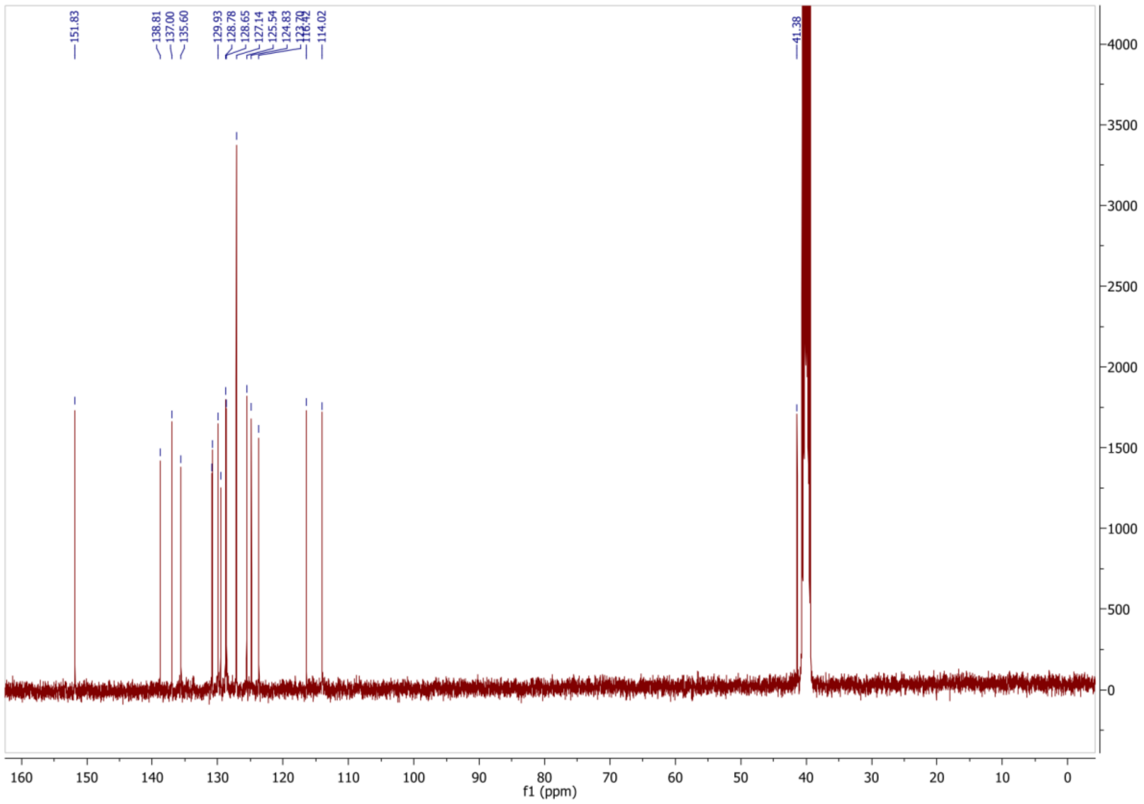

FT-IR:

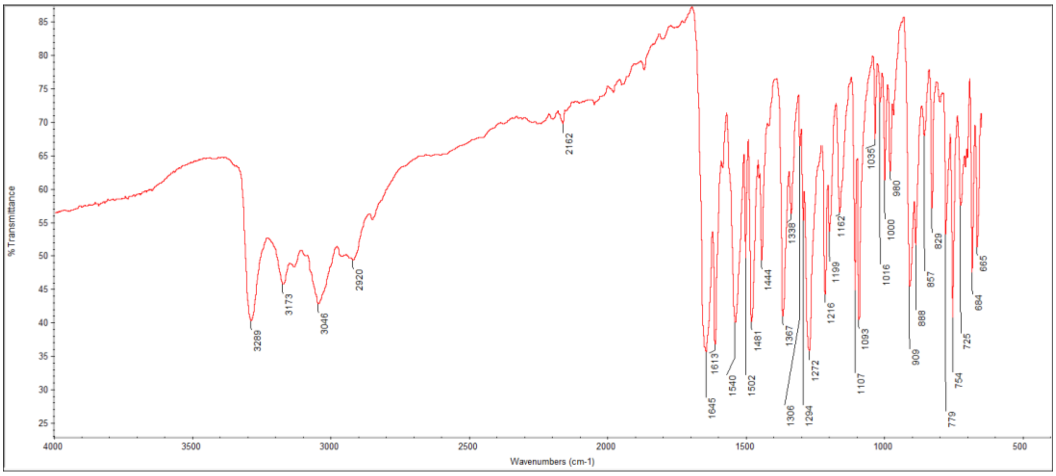

N-(5-fluoro-3,4-dihydroquinazolin-2-yl)naphthalene-1-sulfonamide PR 54

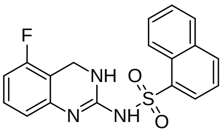

UPLC-MS:

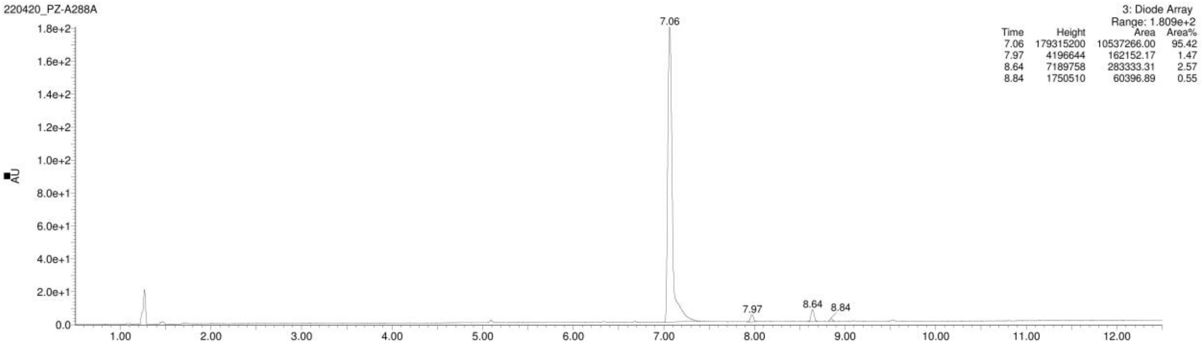

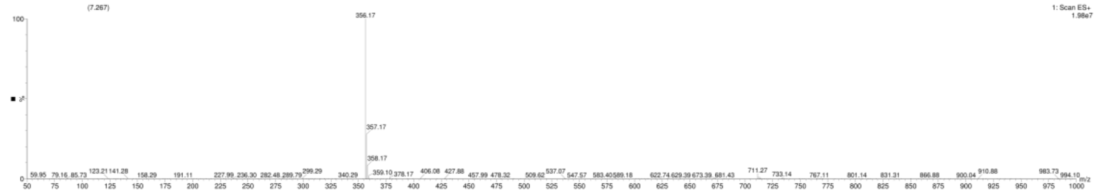

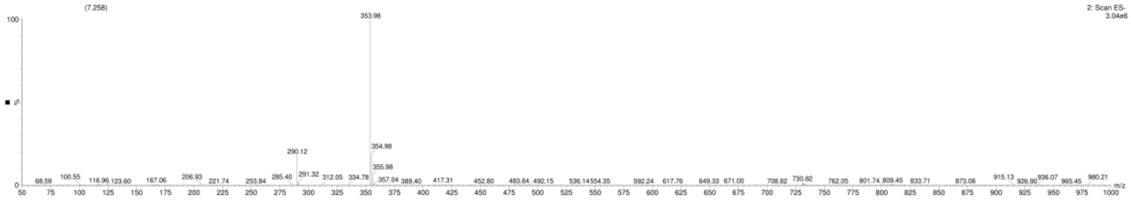

^{1}H NMR:

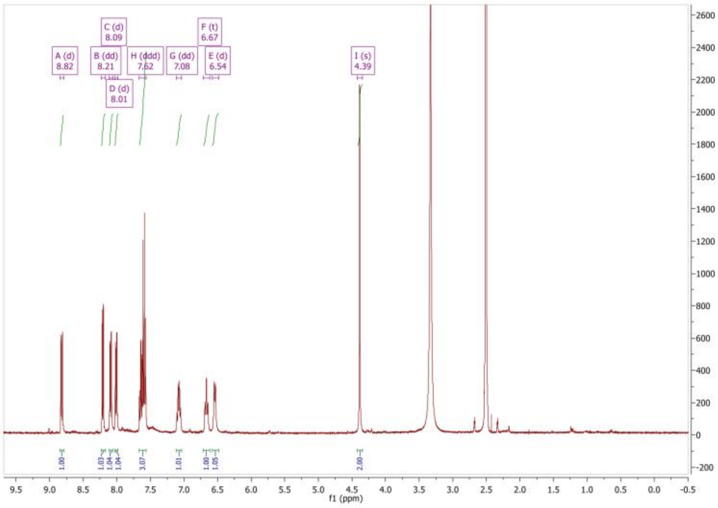

^{13}C NMR:

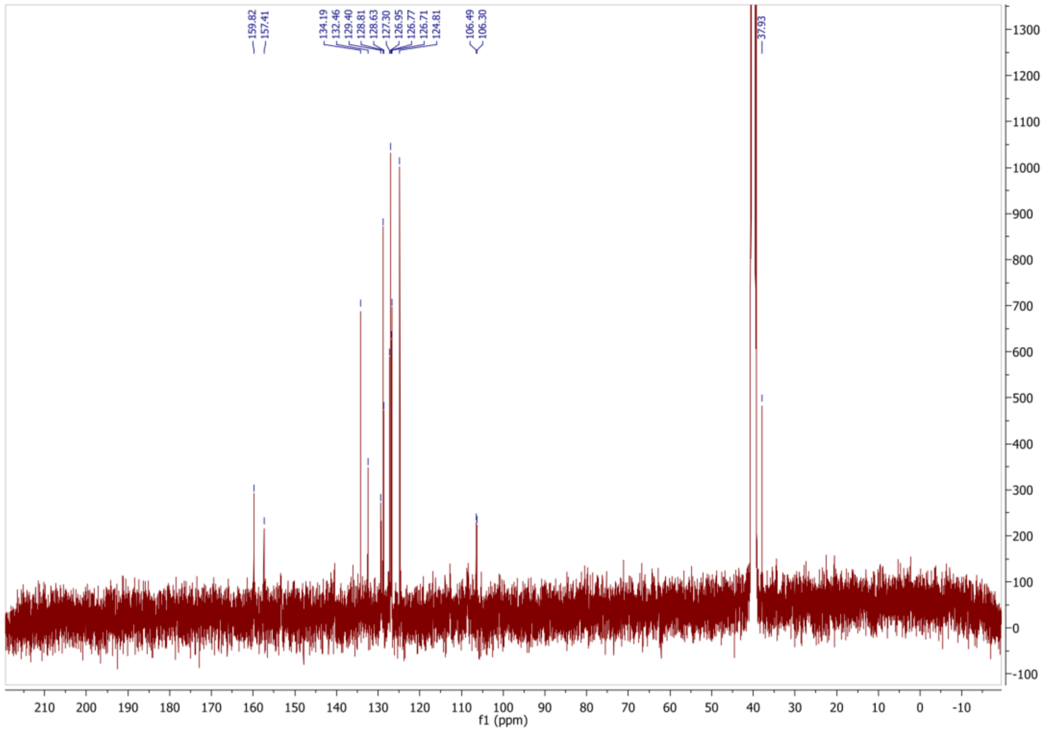

FT-IR:

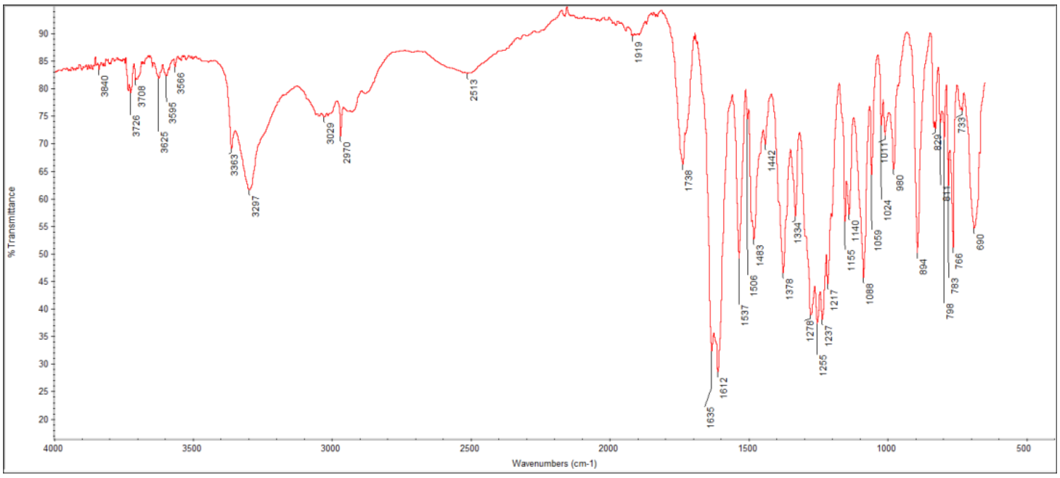

N-(6-fluoro-3,4-dihydroquinazolin-2-yl)naphthalene-1-sulfonamide PR 55

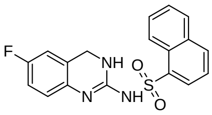

UPLC-MS:

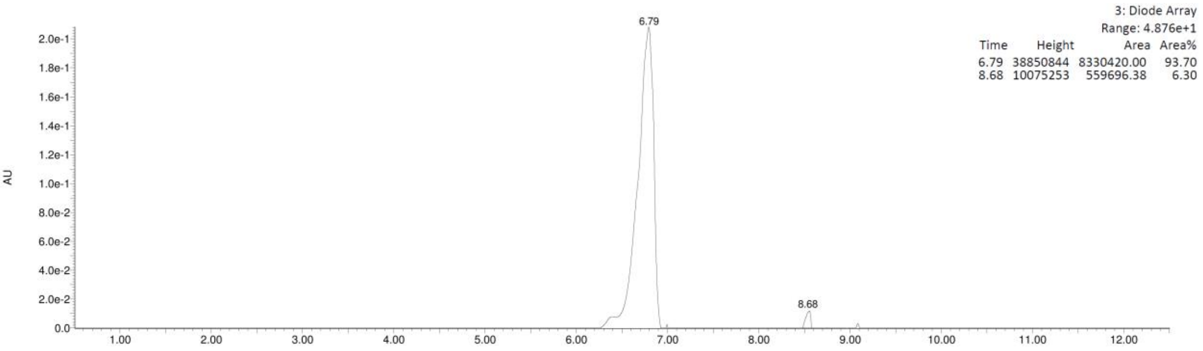
3: Diode Array
Range: 4.876e+1
Time Height Area Area%
6.79 38850844 8330420.00 93.70
8.68 10075253 559696.38 6.30
6.79
8.68
AU

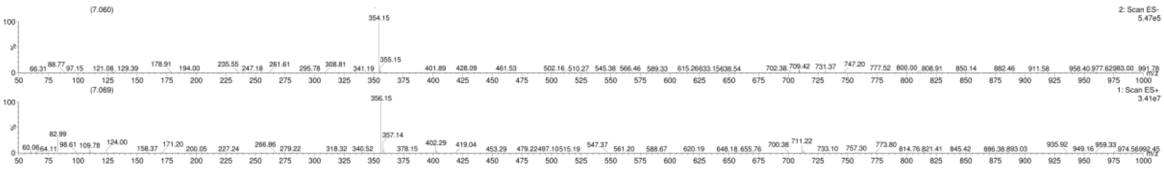
(7.060)
354.15
355.15
2: Scan ES-
5.47e5
(7.069)
356.15
357.14
1: Scan ES+
3.41e7
m/z

^{1}H NMR:

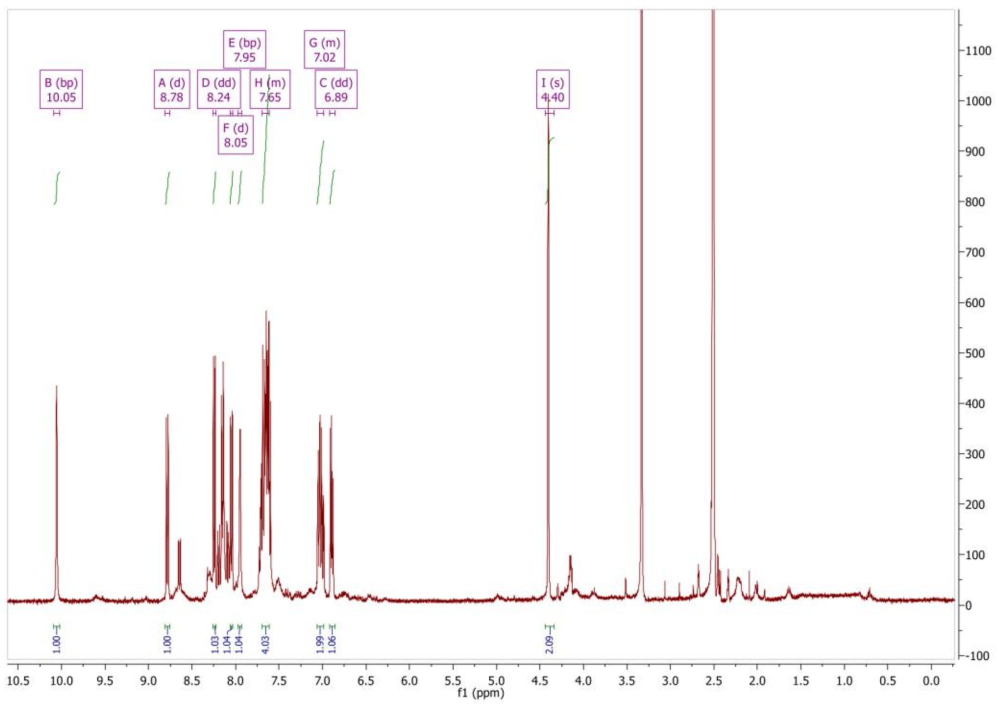
B (bp)
10.05
A (d)
8.78
D (dd)
8.24
E (bp)
7.95
F (d)
8.05
H (m)
7.65
G (m)
7.02
C (dd)
6.89
I (s)
4.40
1.00
1.00
1.03
1.04
1.04
4.03
1.99
1.06
2.09
f1 (ppm)

^{13}C NMR:

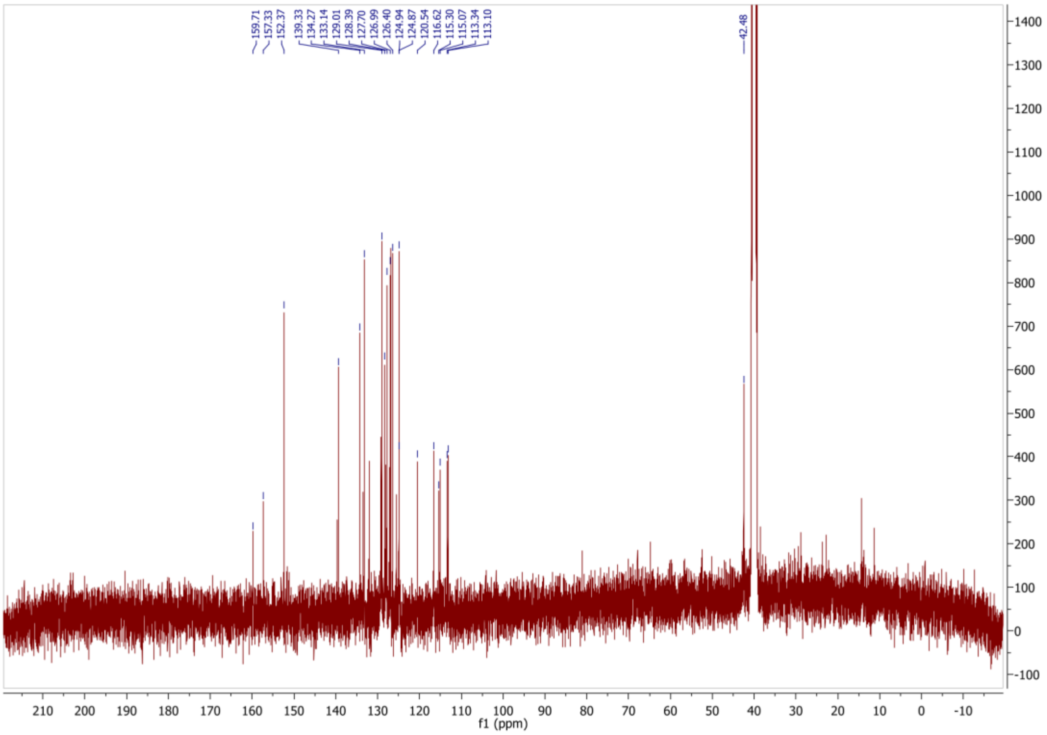

FT-IR:

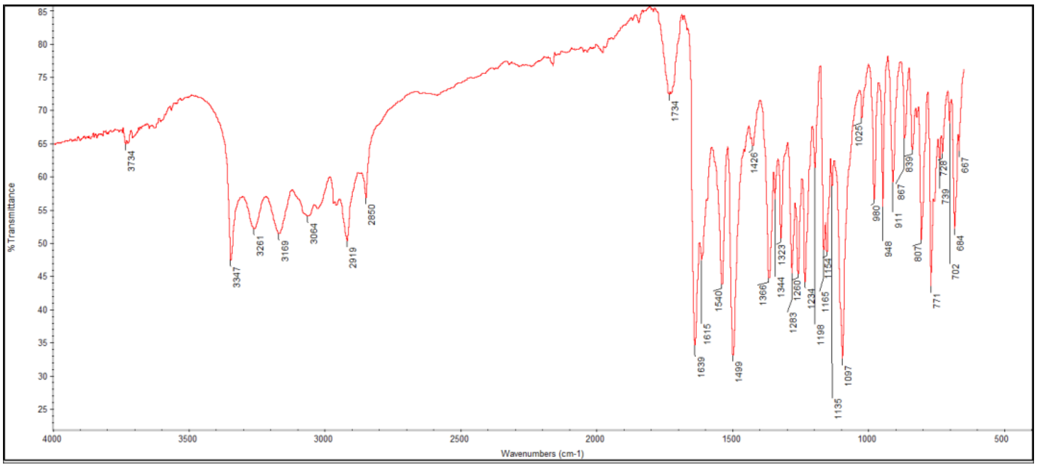

N-(7-fluoro-3,4-dihydroquinazolin-2-yl)naphthalene-1-sulfonamide PR 56

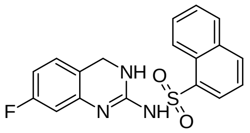

UPLC-MS:

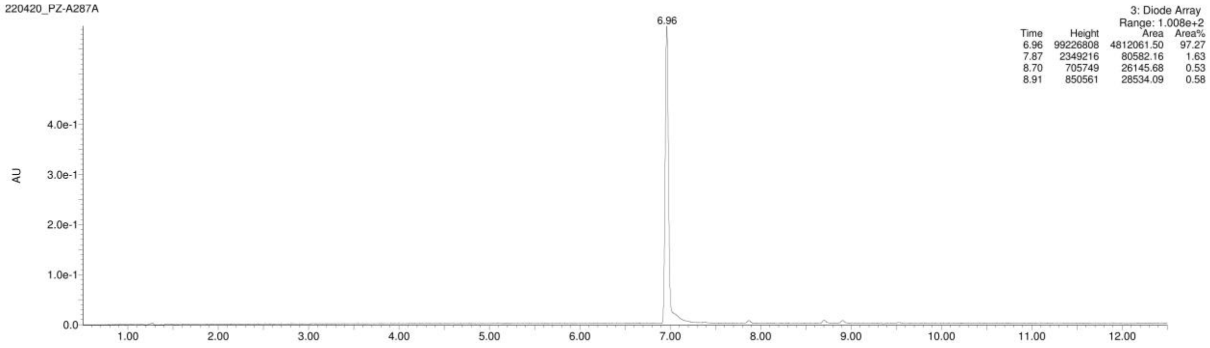

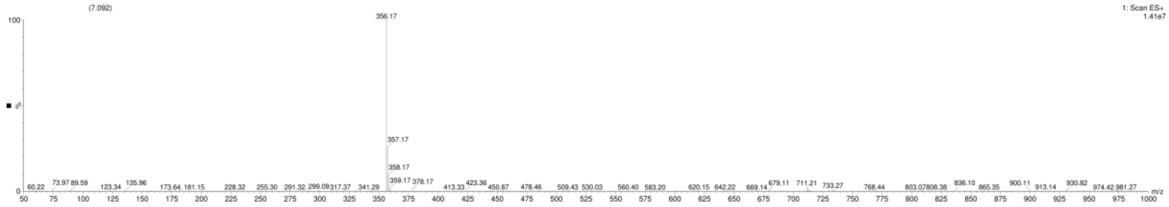

^{1}H NMR:

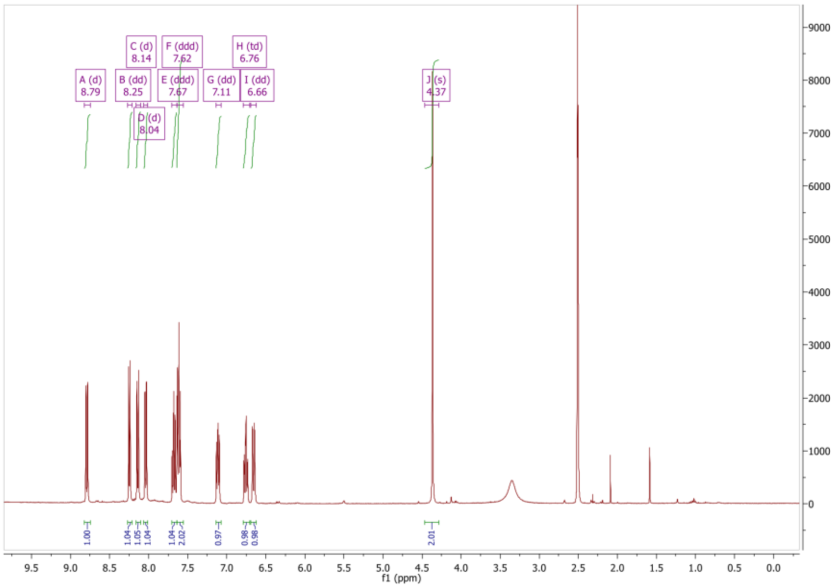

^{13}C NMR:

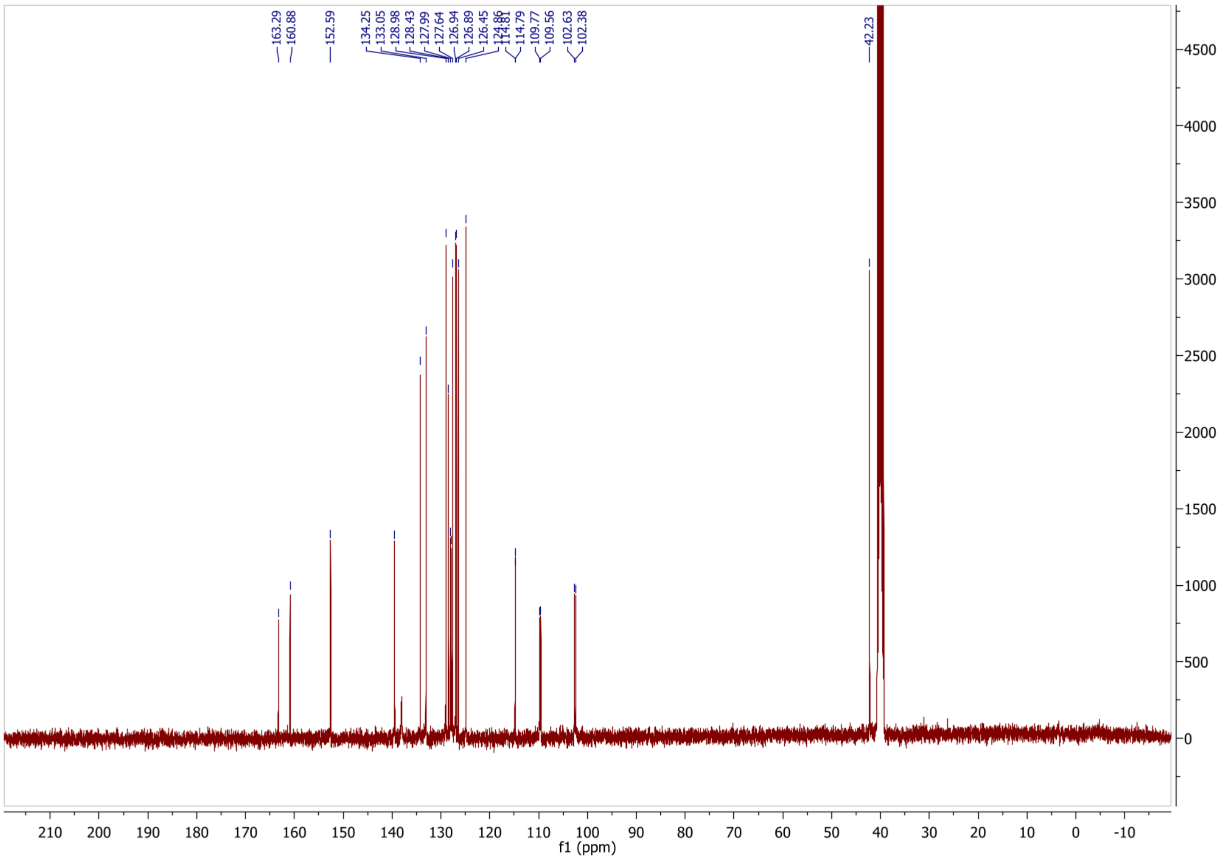

FT-IR:

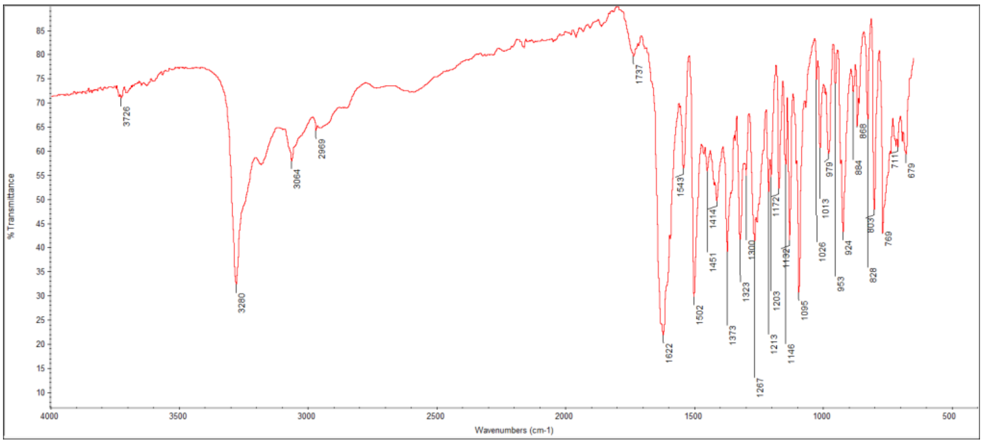

N-[6-(trifluoromethyl)-3,4-dihydroquinazolin-2-yl]naphthalene-1-sulfonamide PR 57

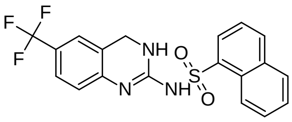

UPLC-MS:

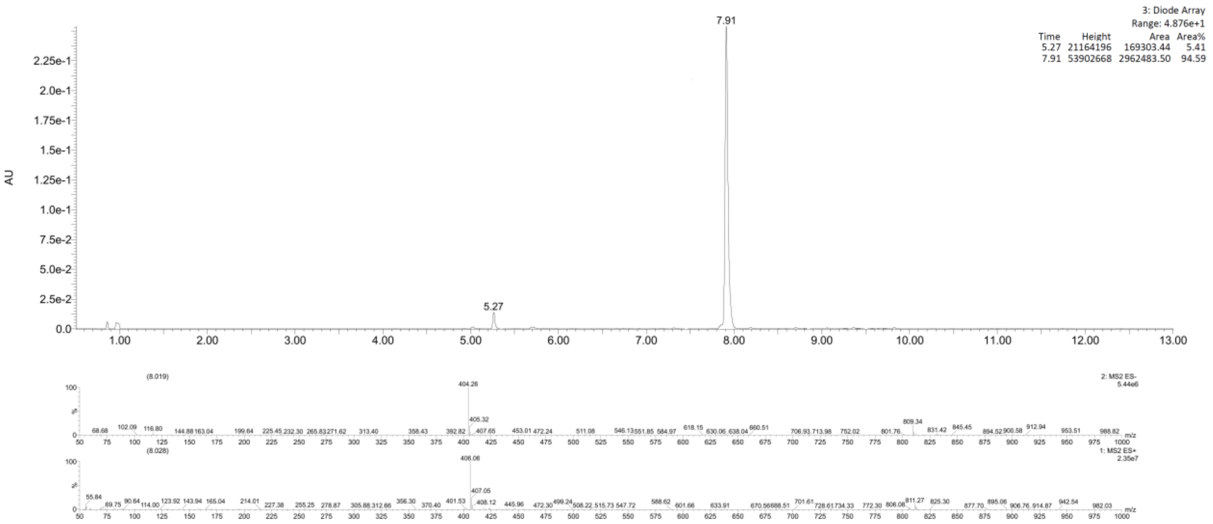
3: Diode Array
Range: 4.876e+1
Time Height Area Area%
5.27 21164196 169303.44 5.41
7.91 53902668 2962483.50 94.59
7.91
5.27
AU
2: MS2 ES-
5.44e6
1: MS2 ES+
2.35e7
404.26
406.06
m/z

^{1}H NMR:

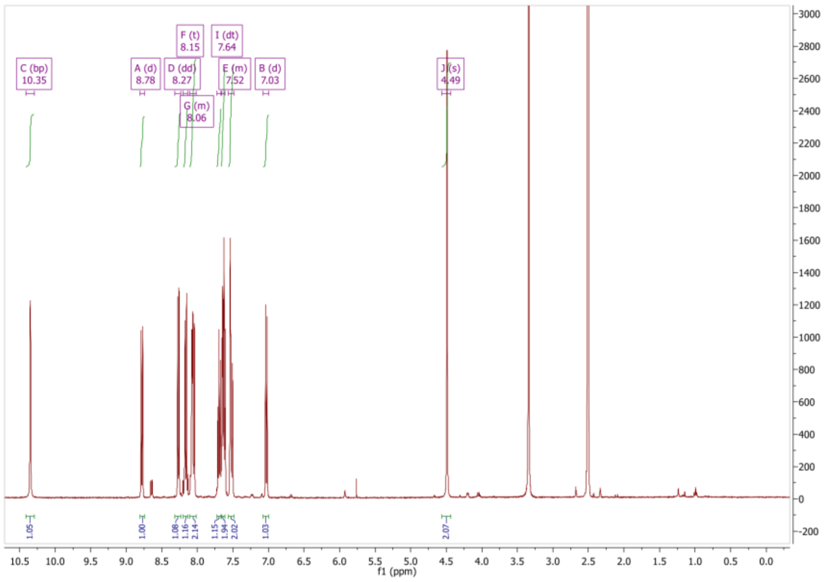
C (bp) 10.35
A (d) 8.78
D (dd) 8.27
F (t) 8.15
G (m) 8.06
I (dt) 7.64
E (m) 7.52
B (d) 7.03
J (s) 4.49
f1 (ppm)

^{13}C NMR:

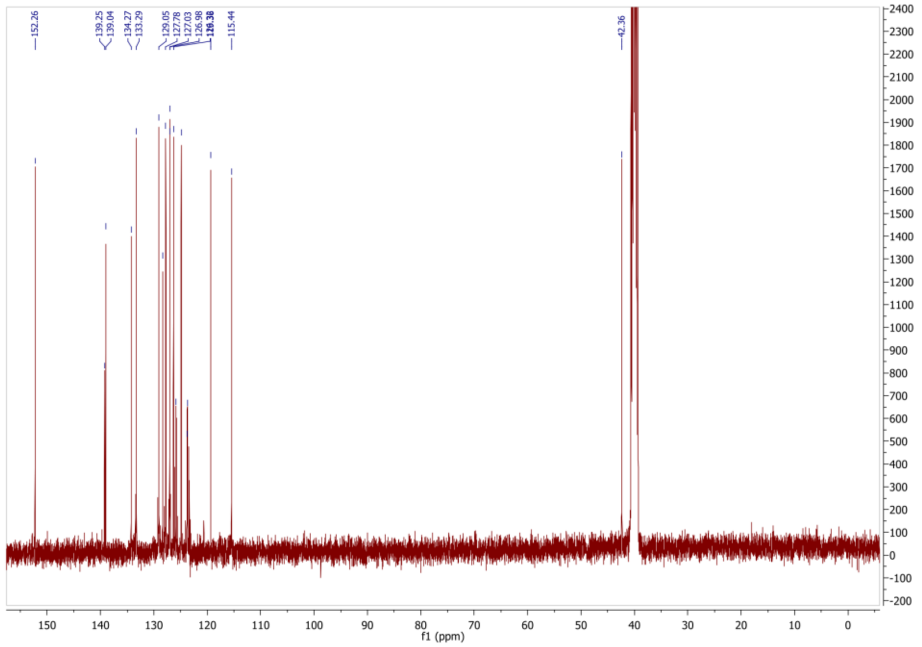

FT-IR:

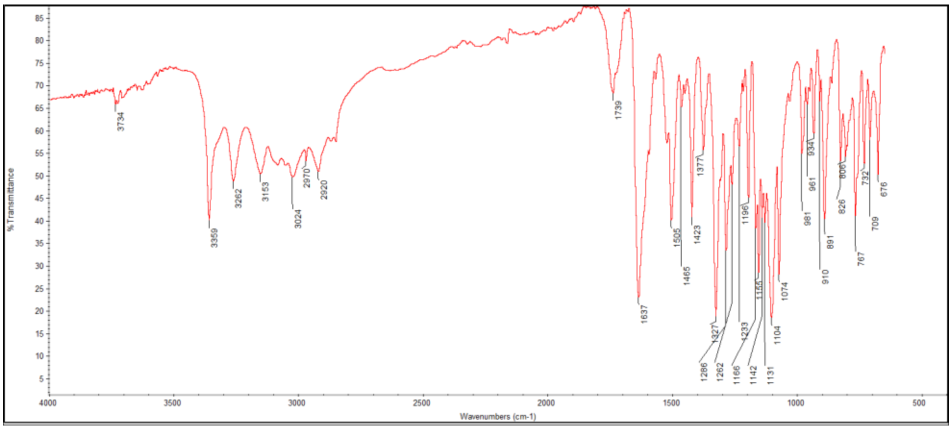

N-(5-methoxy-3,4-dihydroquinazolin-2-yl)naphthalene-1-sulfonamide PR 58

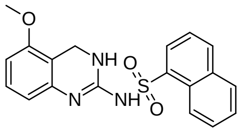

UPLC-MS:

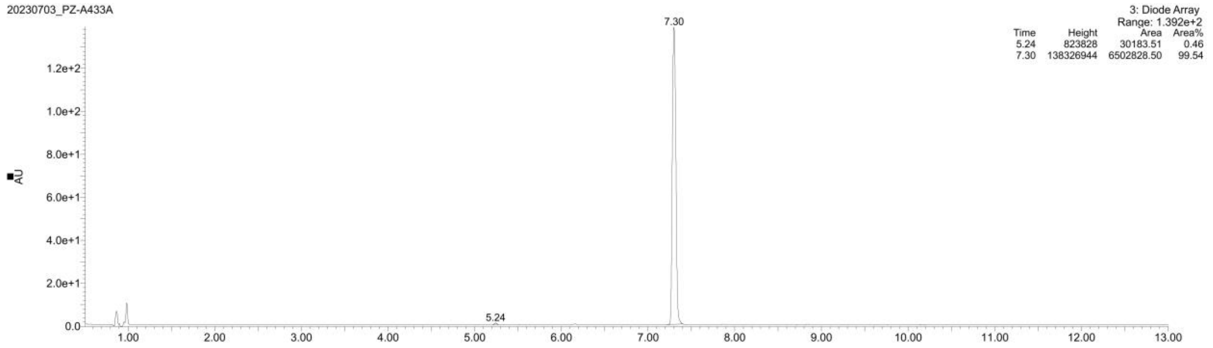
20230703_PZ-A433A
3: Diode Array
Range: 1.392e+2
Time Height Area Area%
5.24 823828 30183.51 0.46
7.30 138326944 6502828.50 99.54
7.30
5.24
AU

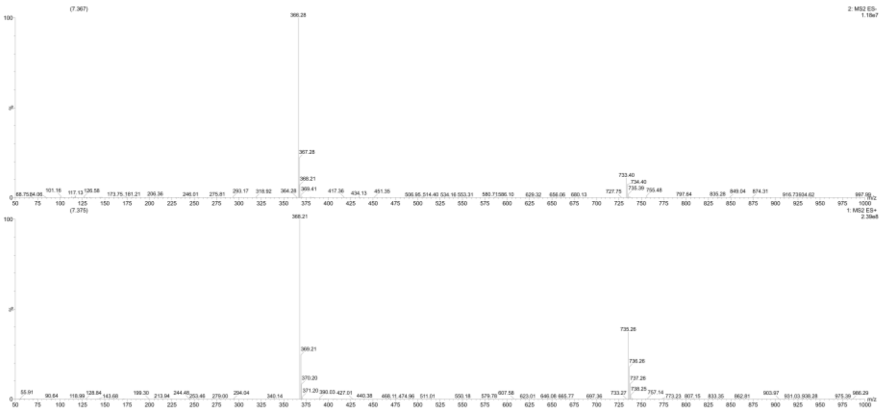
2: MS2 ES-
1: MS2 ES+
366.28
368.21
735.26

^{1}H NMR:

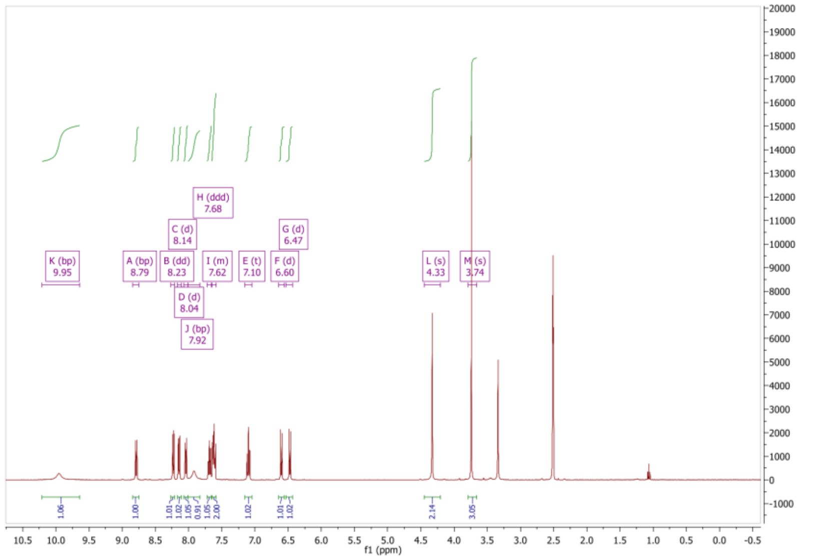
K (bp) 9.95
A (bp) 8.79
B (dd) 8.23
C (d) 8.14
D (d) 8.04
J (bp) 7.92
H (ddd) 7.68
I (m) 7.62
E (t) 7.10
F (d) 6.60
G (d) 6.47
L (s) 4.33
M (s) 3.74
f1 (ppm)

^{13}C NMR:

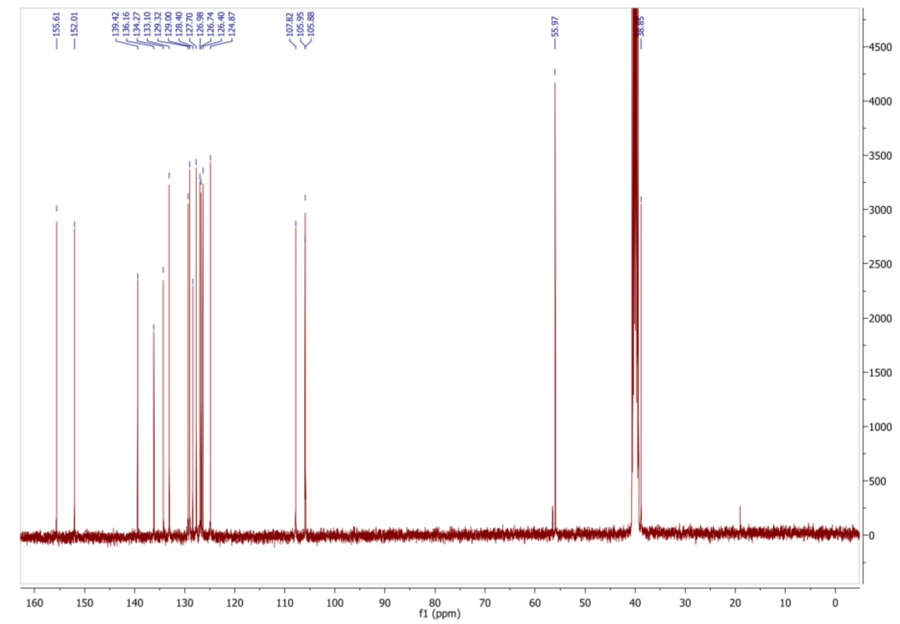

FT-IR:

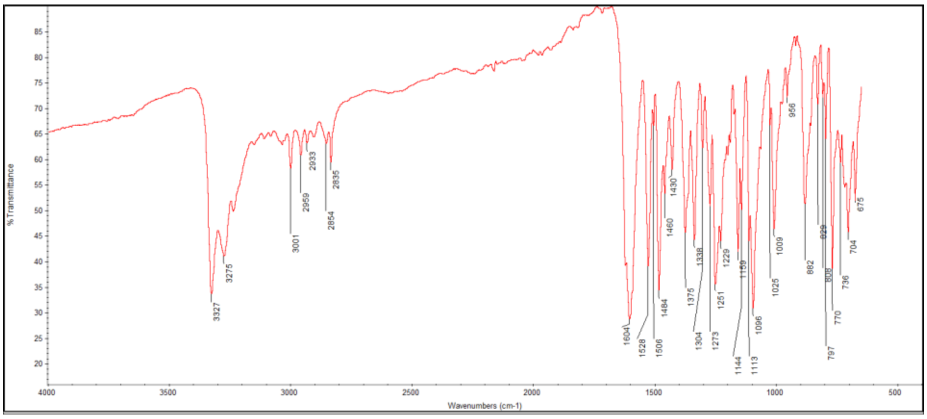

N-(6-methoxy-3,4-dihydroquinazolin-2-yl)naphthalene-1-sulfonamide PR 59

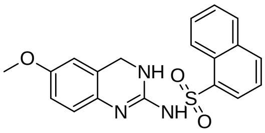

UPLC-MS:

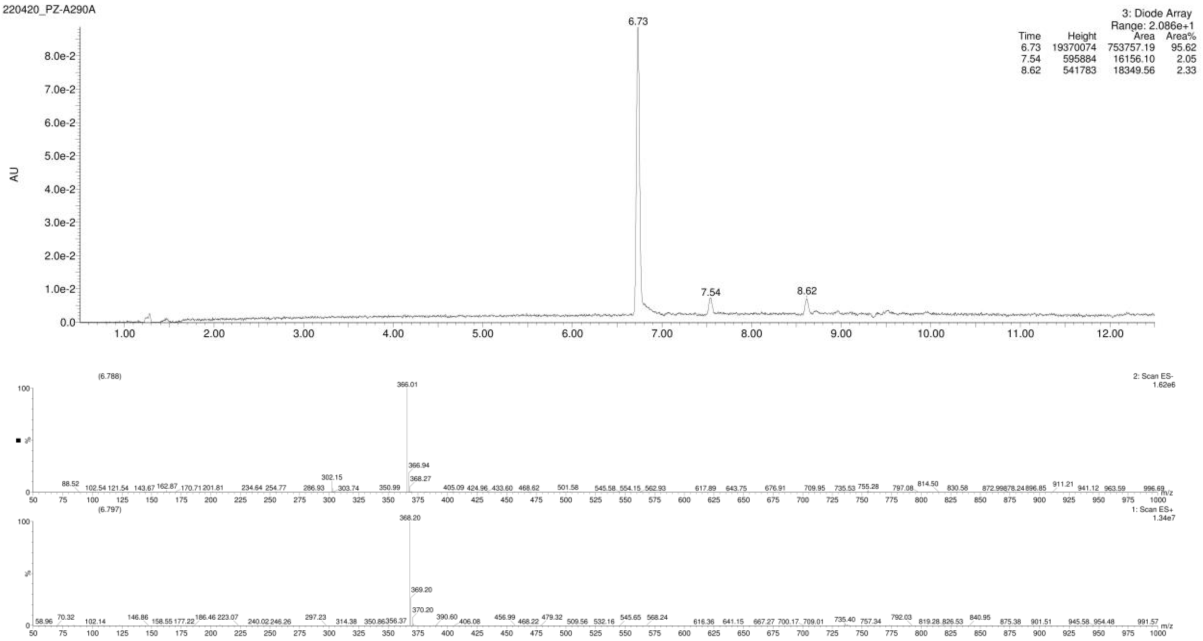

^{1}H NMR:

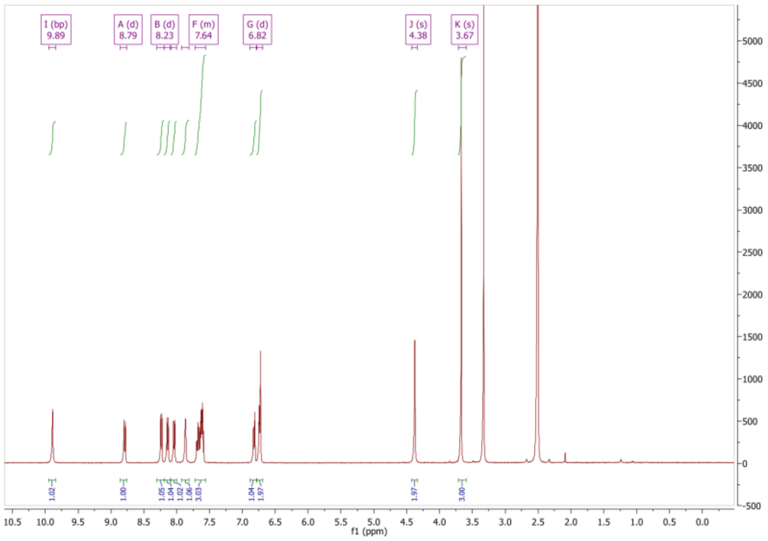

^{13}C NMR:

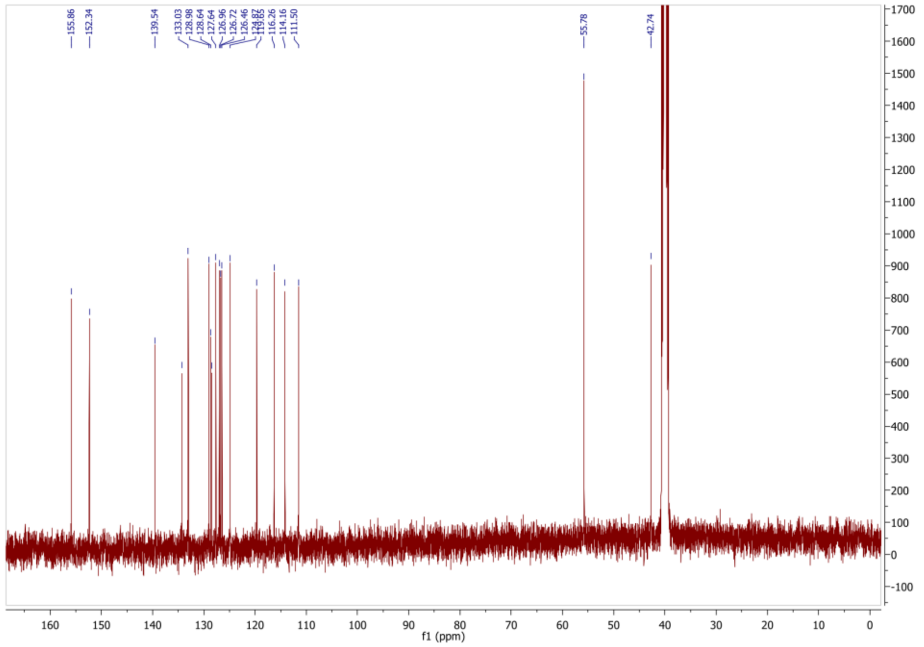

FT-IR:

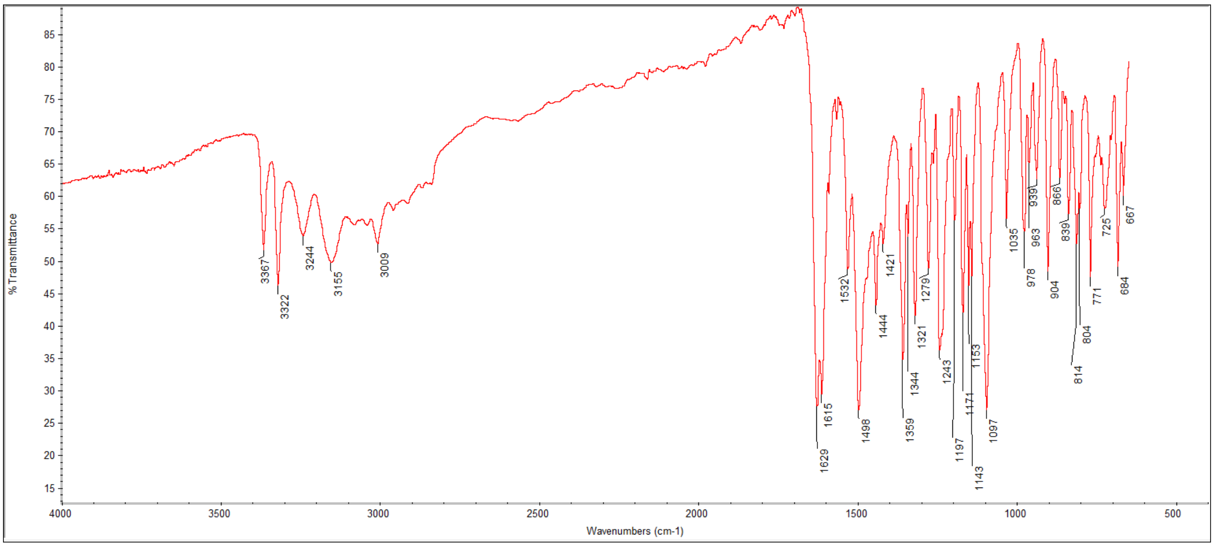

N-(7-methoxy-3,4-dihydroquinazolin-2-yl)naphthalene-1-sulfonamide PR 60

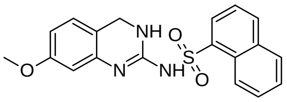

UPLC-MS:

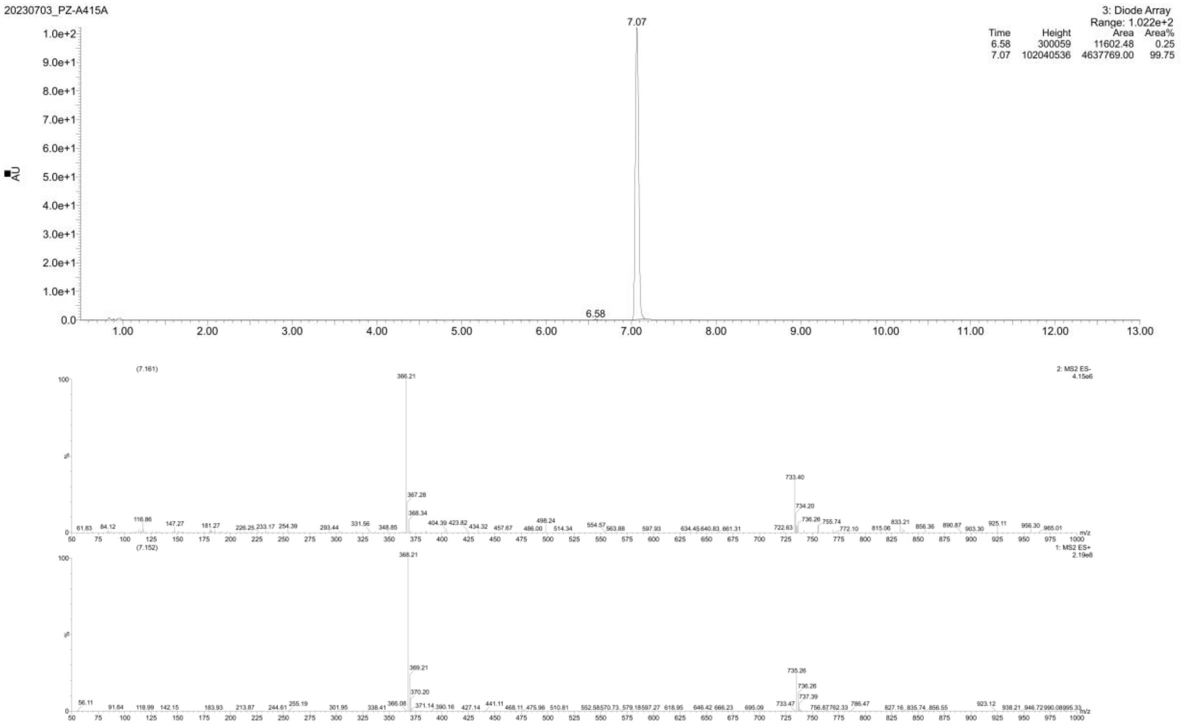

^{1}H NMR:

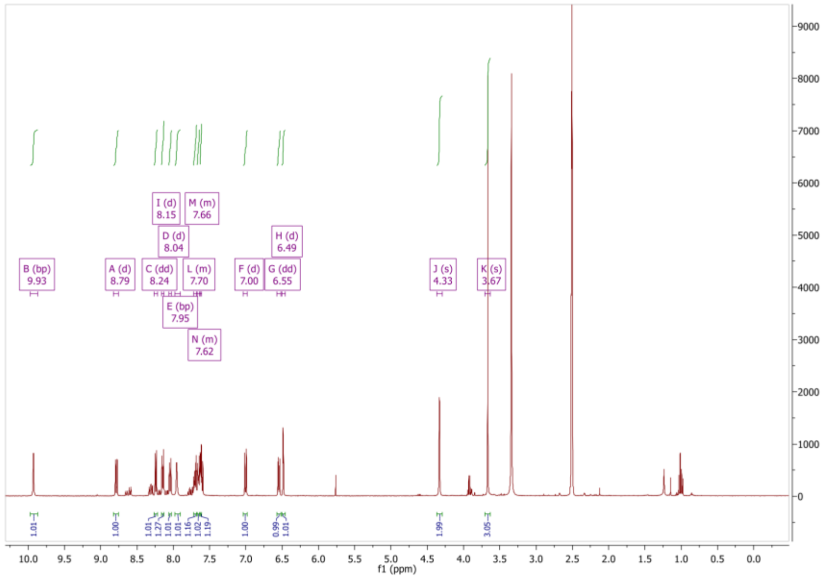

^{13}C NMR:

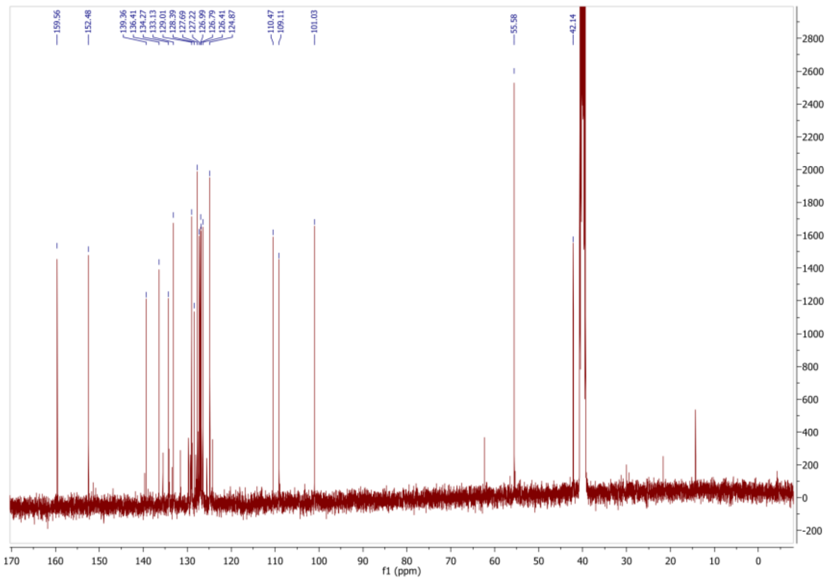

FT-IR:

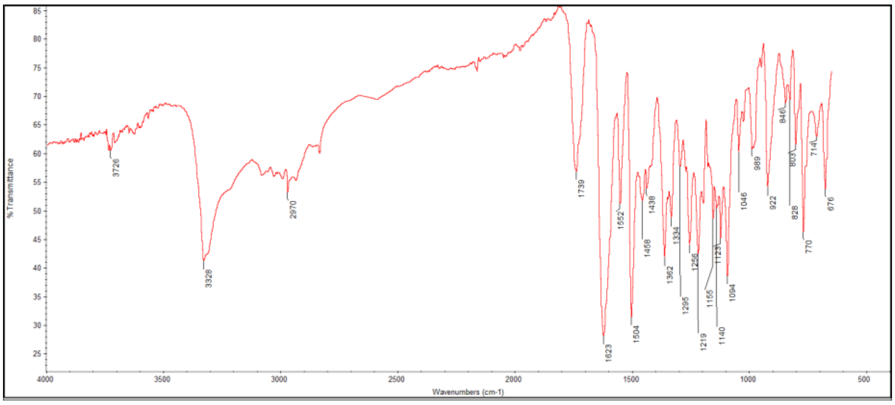

N-(5-methyl-3,4-dihydroquinazolin-2-yl)naphthalene-1-sulfonamide PR 61

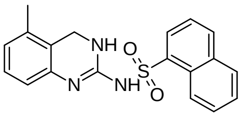

UPLC-MS:

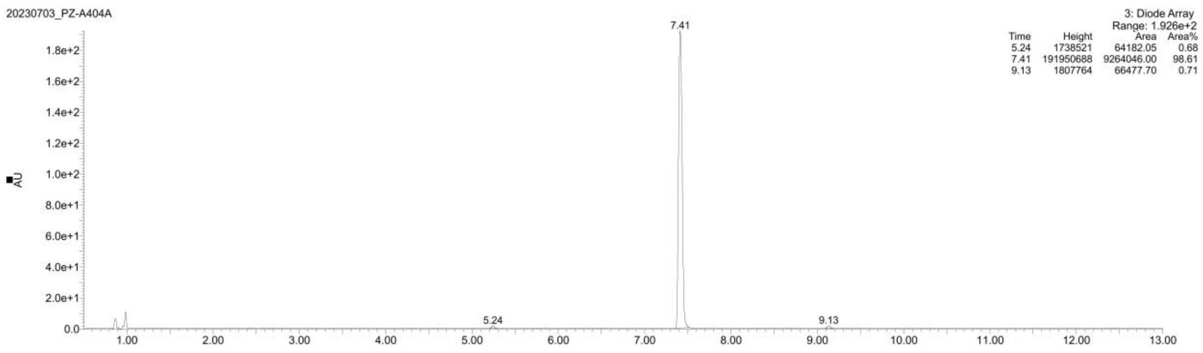

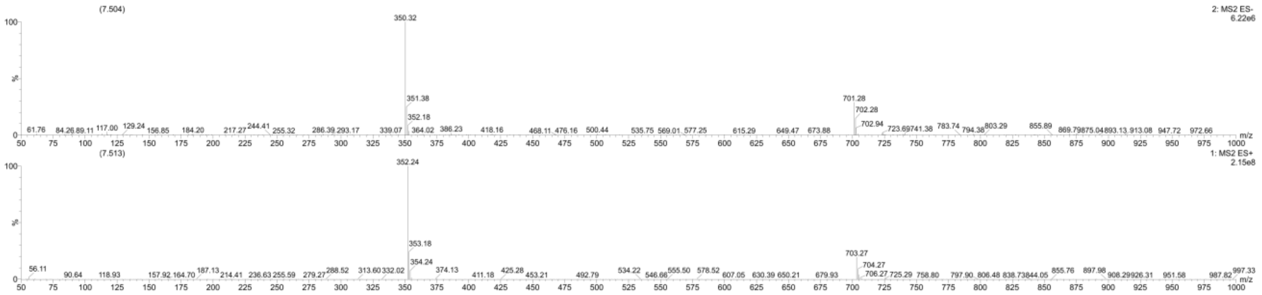

^{1}H NMR:

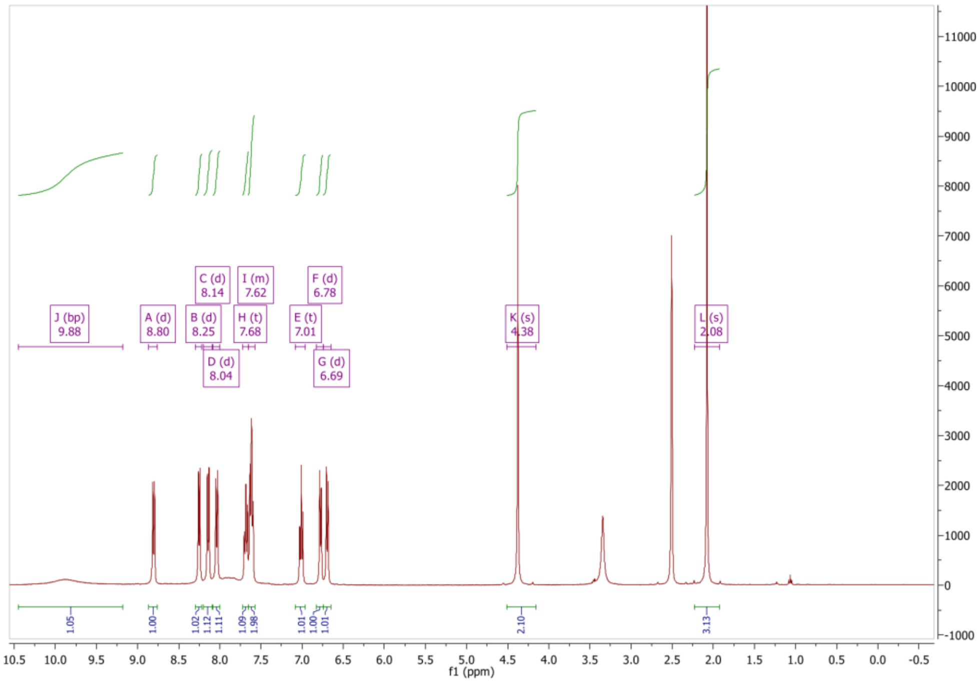

^{13}C NMR:

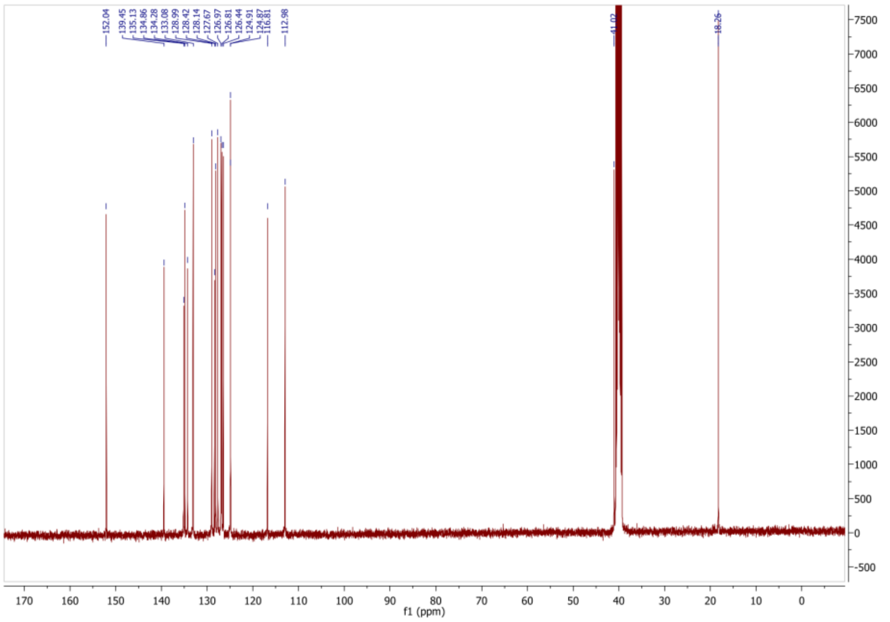

FT-IR:

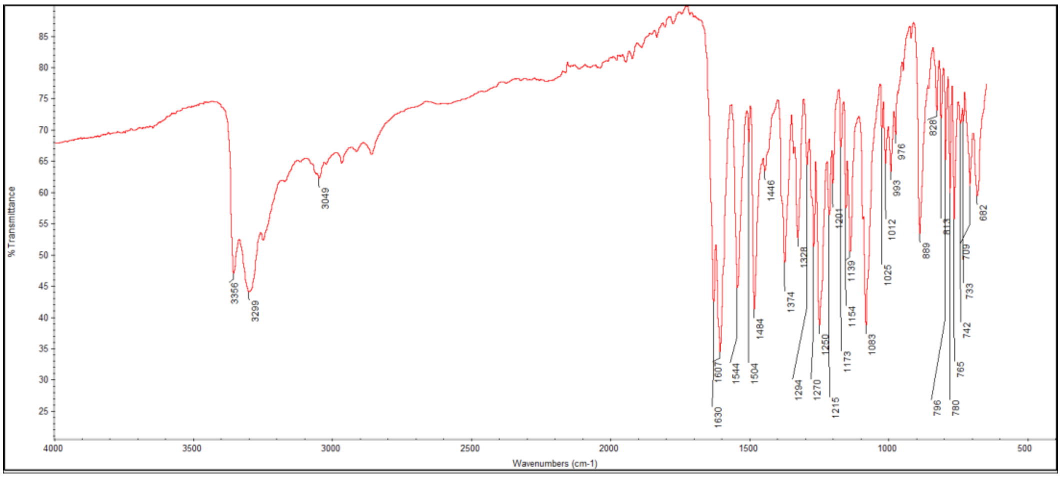

N-(7-methyl-3,4-dihydroquinazolin-2-yl)naphthalene-1-sulfonamide PR 62

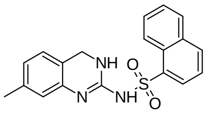

UPLC-MS:

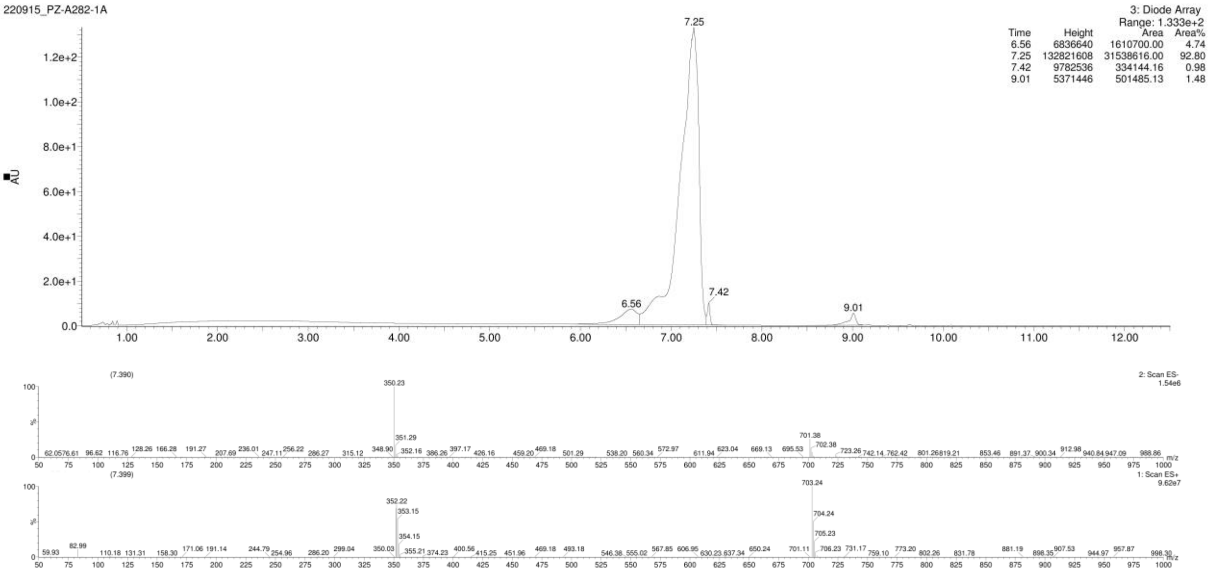

^{1}H NMR:

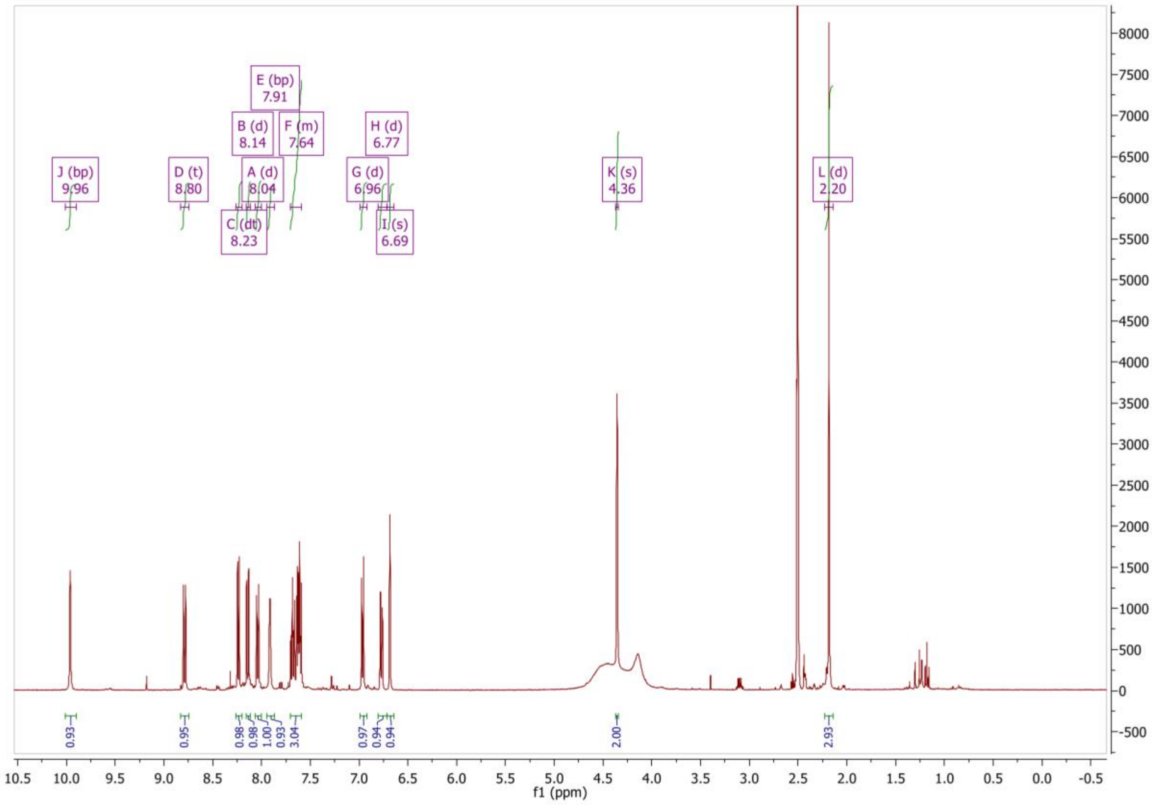

^{13}C NMR:

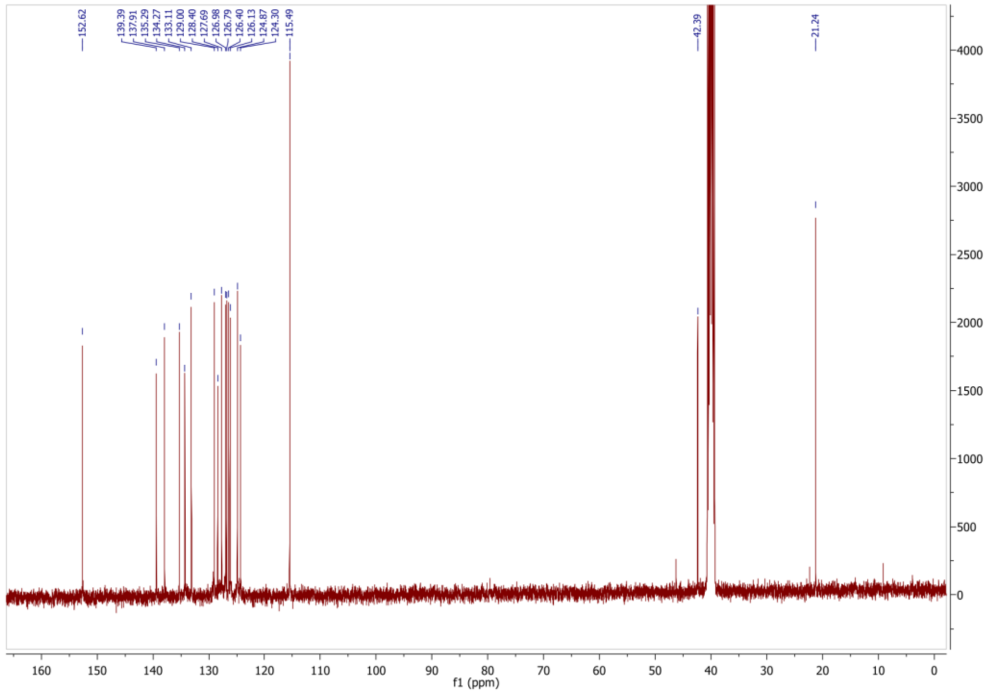

FT-IR:

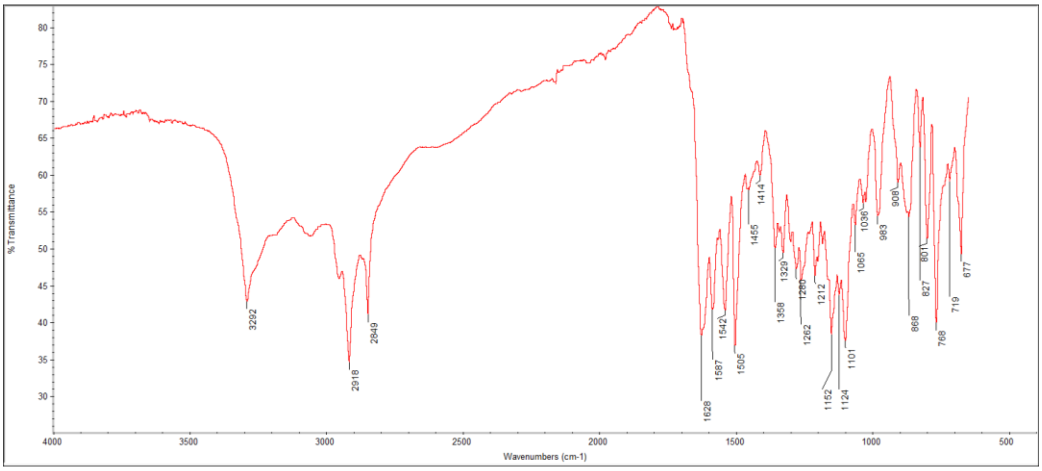

N-[6-(dimethylamino)-3,4-dihydroquinazolin-2-yl]naphthalene-1-sulfonamide PR 64

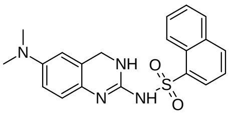

UPLC-MS:

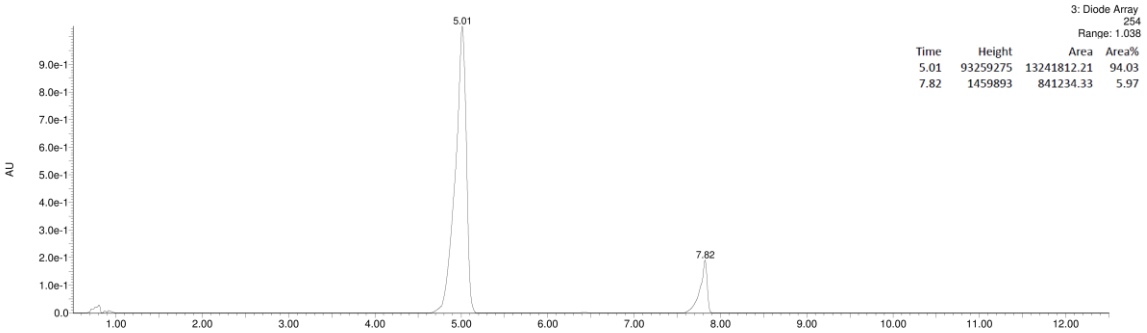

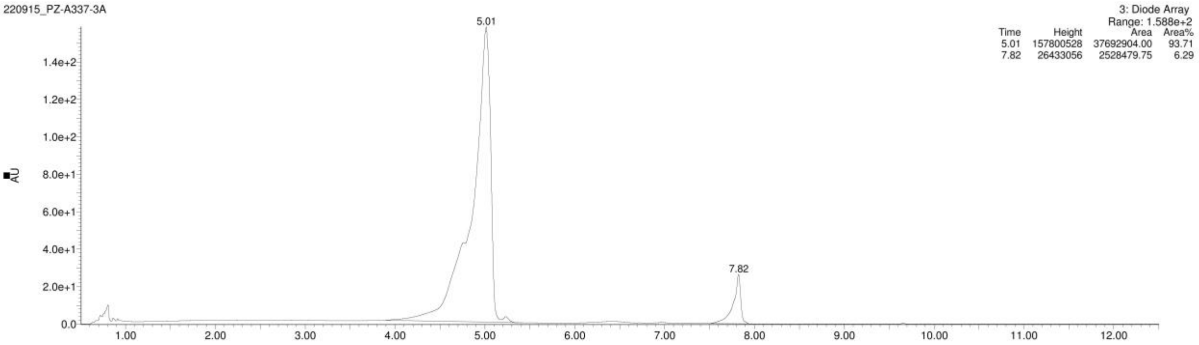

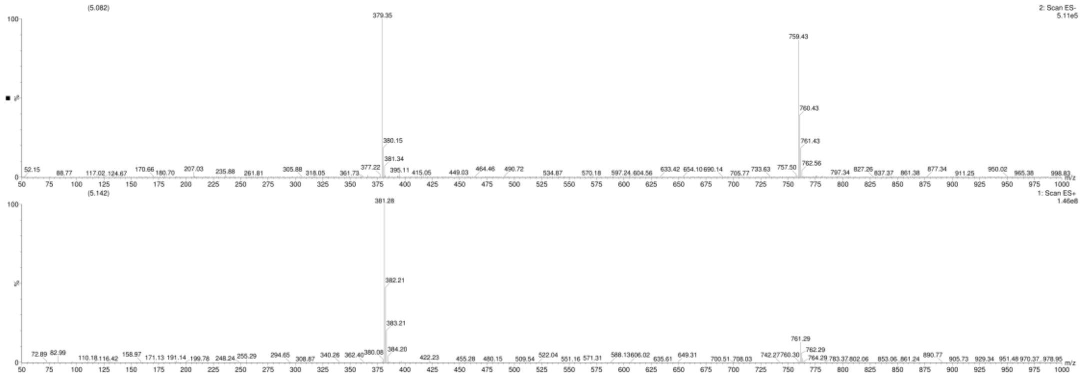

^{1}H NMR:

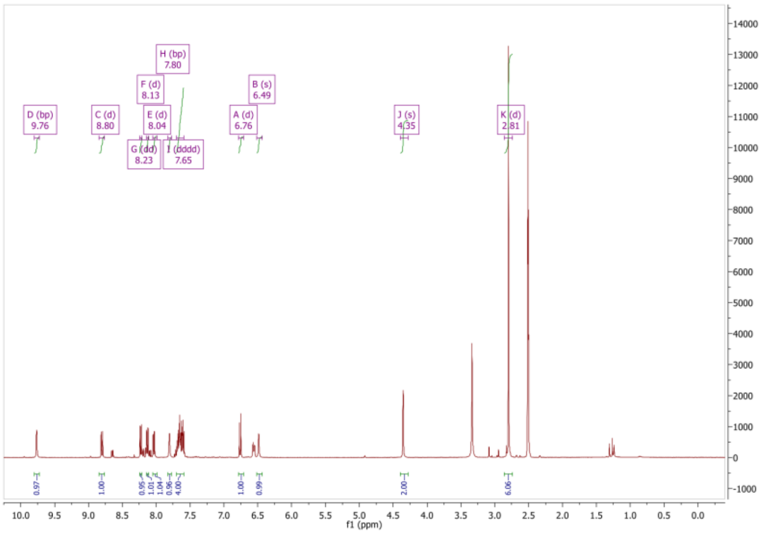

^{13}C NMR:

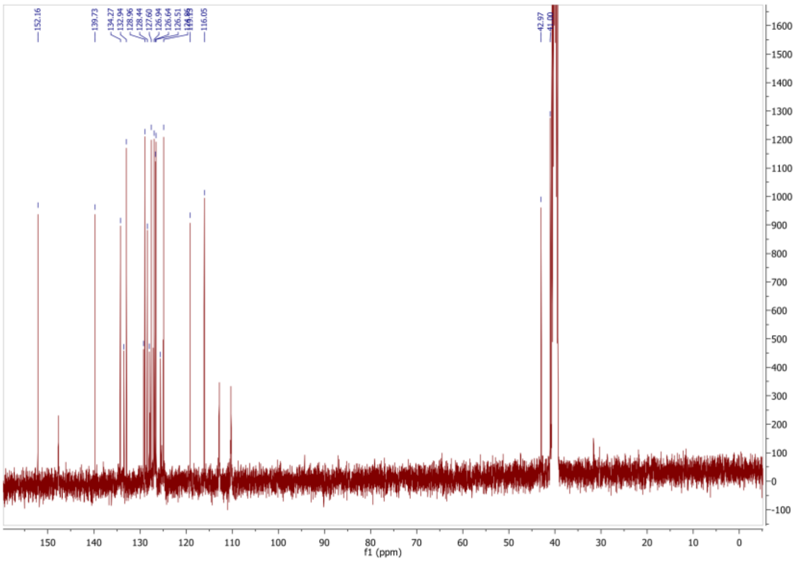

FT-IR:

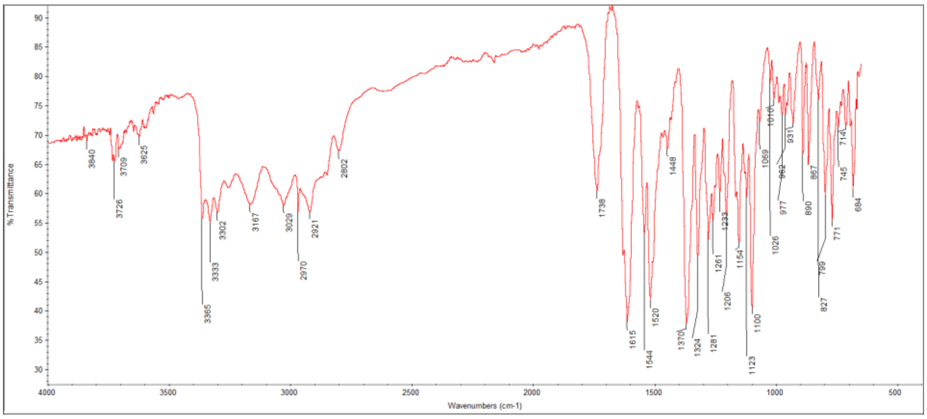

N-[6-(morpholin-4-yl)-3,4-dihydroquinazolin-2-yl]naphthalene-1-sulfonamide PR 65

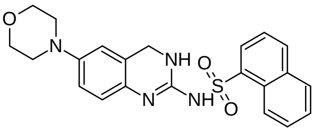

UPLC-MS:

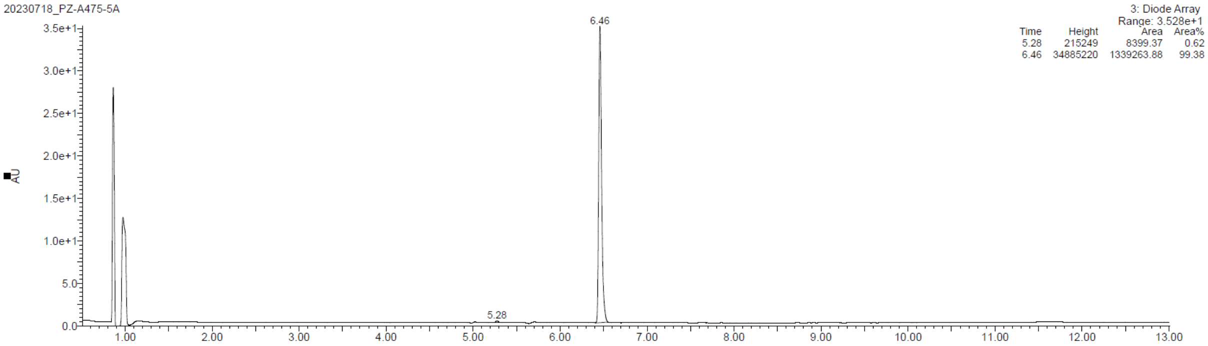

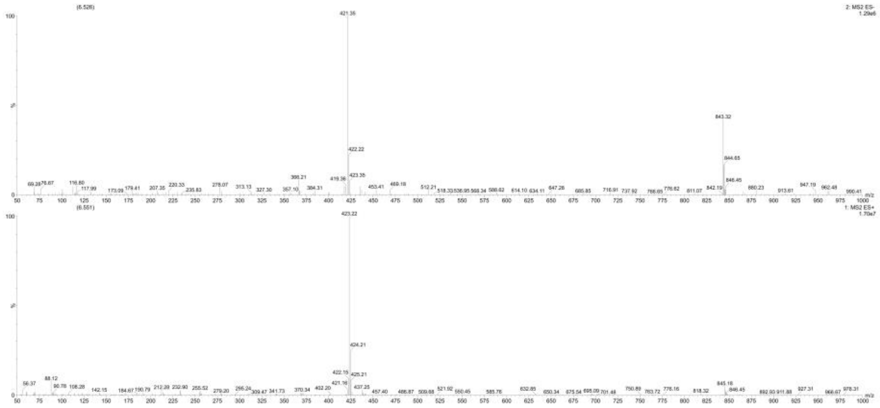

^{1}H NMR:

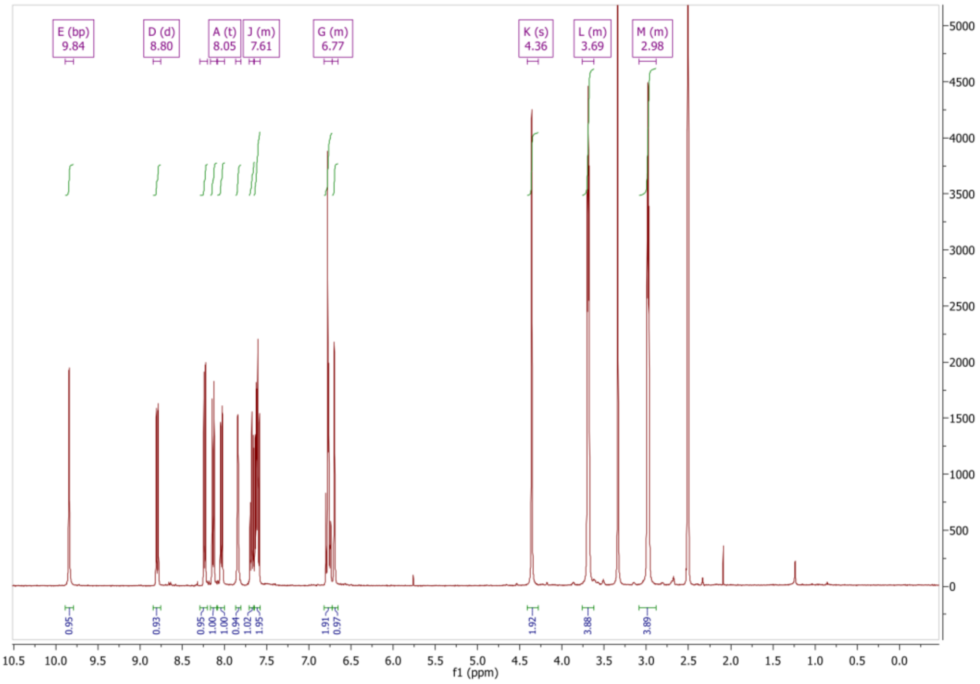

^{13}C NMR:

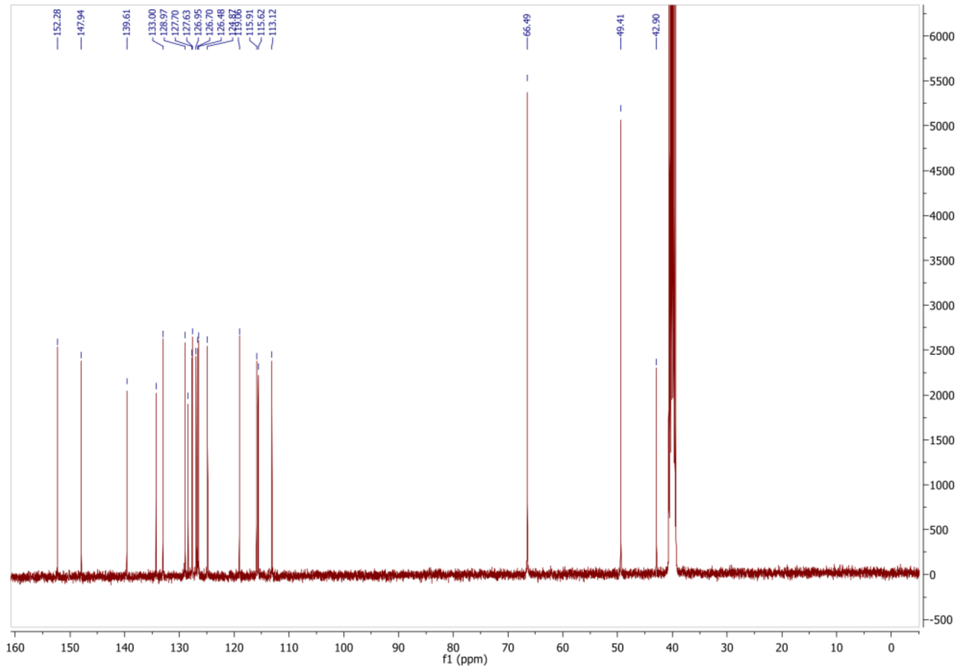

FT-IR:

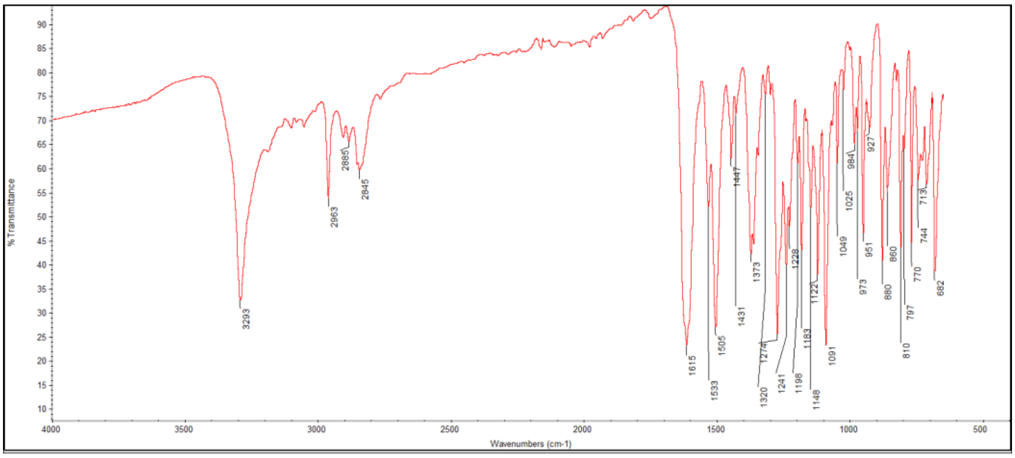

N-(4-methyl-3,4-dihydroquinazolin-2-yl)naphthalene-1-sulfonamide PR 67

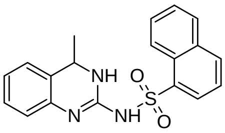

UPLC-MS:

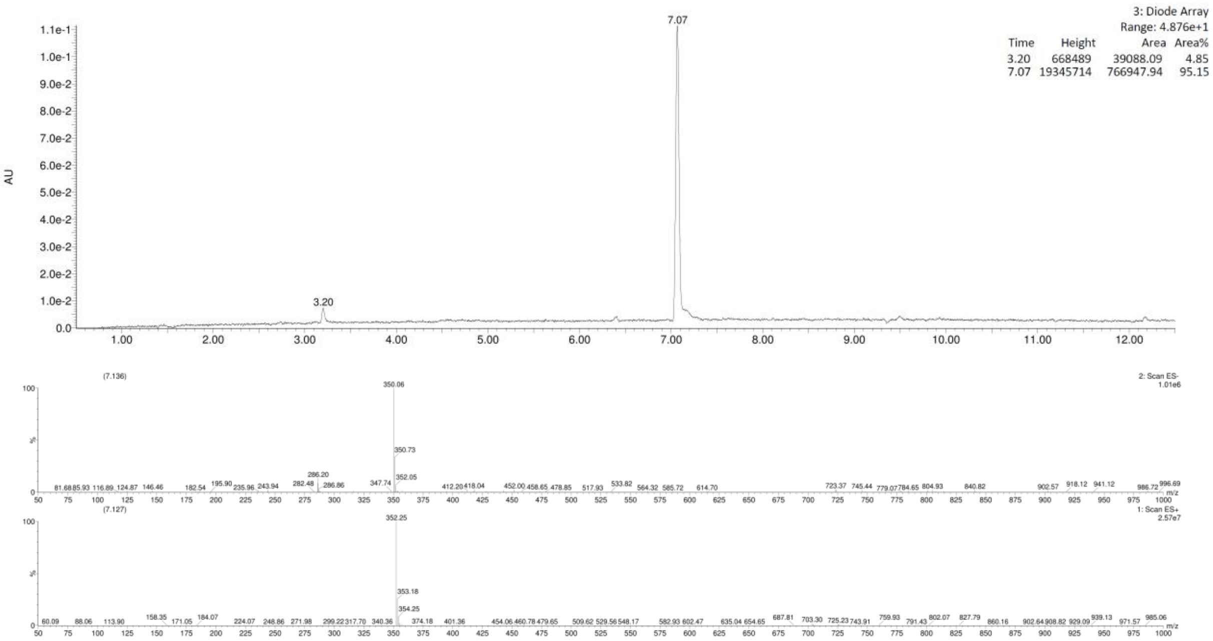

^{1}H NMR:

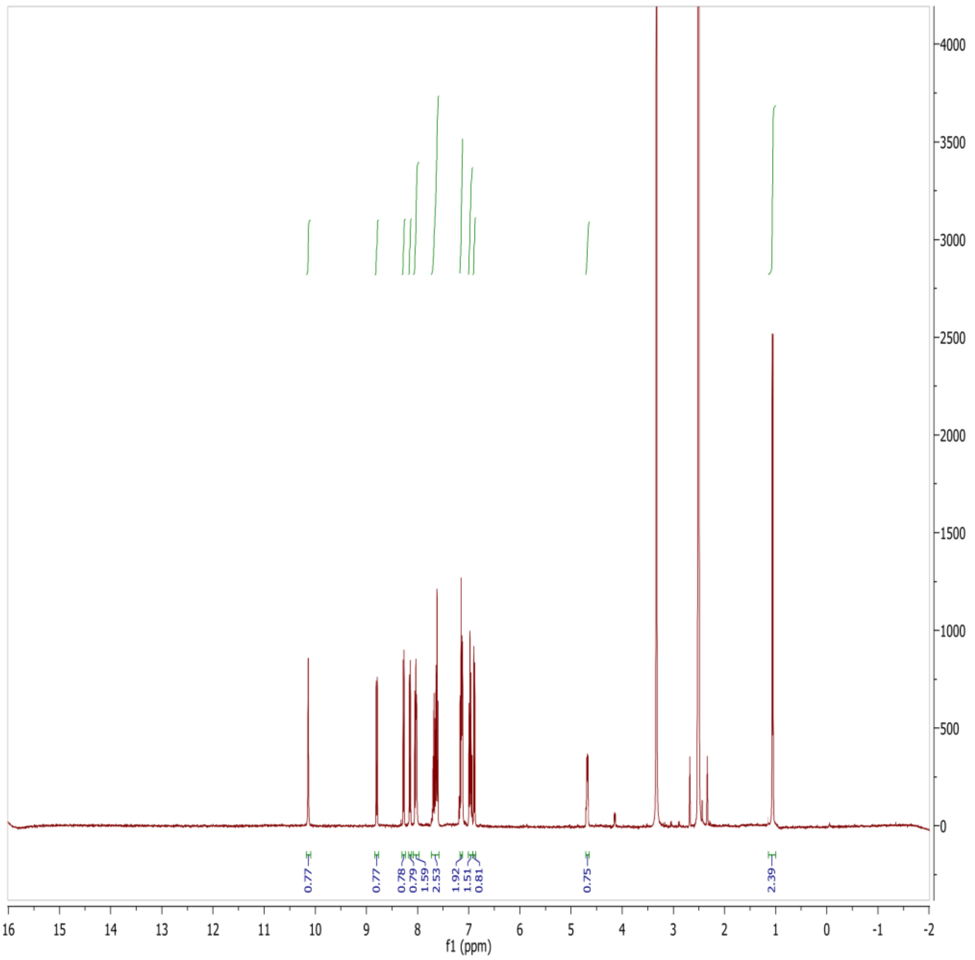

^{13}C NMR:

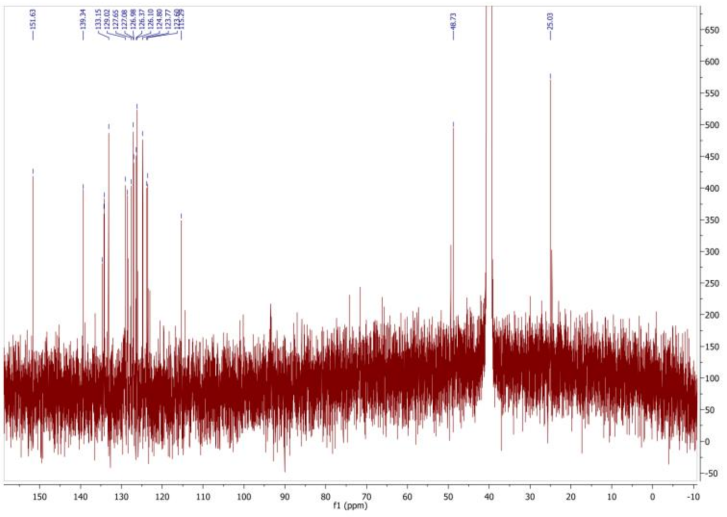

FT-IR:

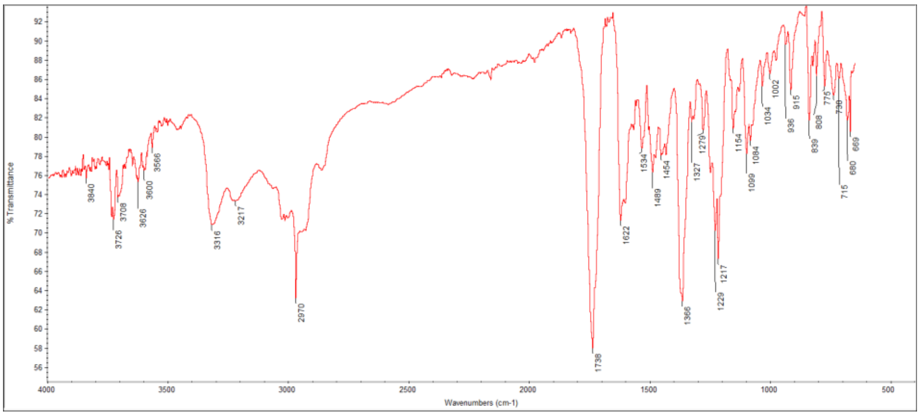

N-(5-chloro-4-methyl-3,4-dihydroquinazolin-2-yl)naphthalene-1-sulfonamide PR 68

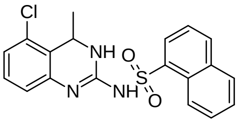

UPLC-MS:

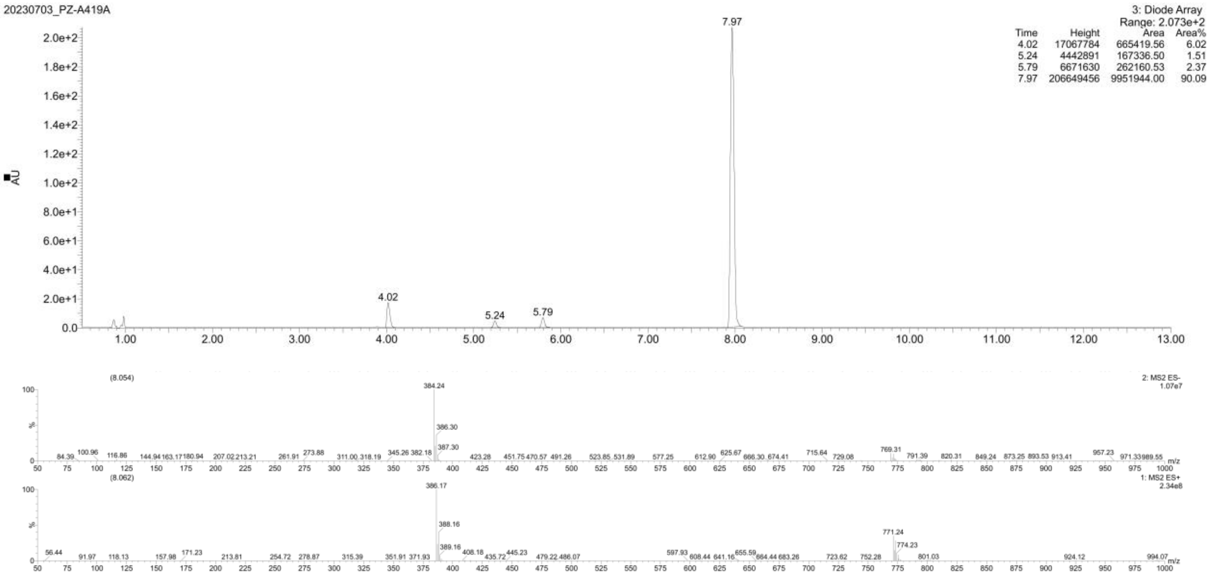

^{1}H NMR:

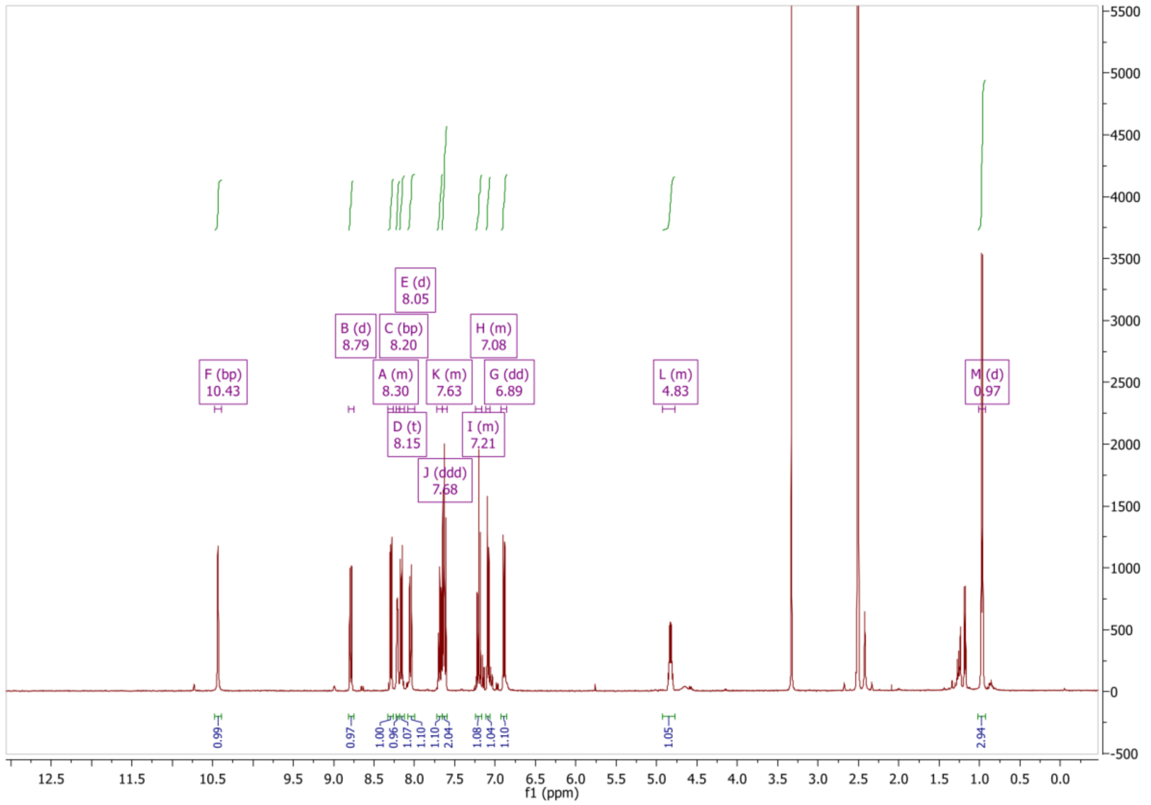

^{13}C NMR:

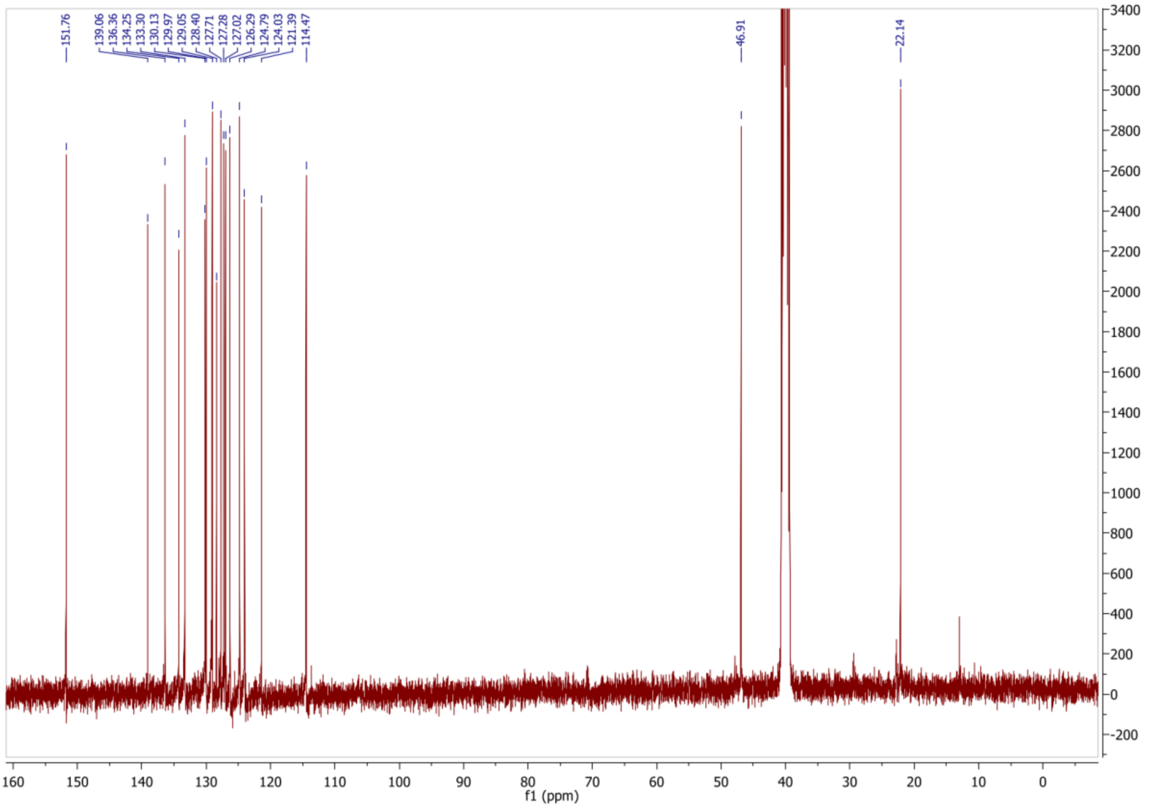

FT-IR:

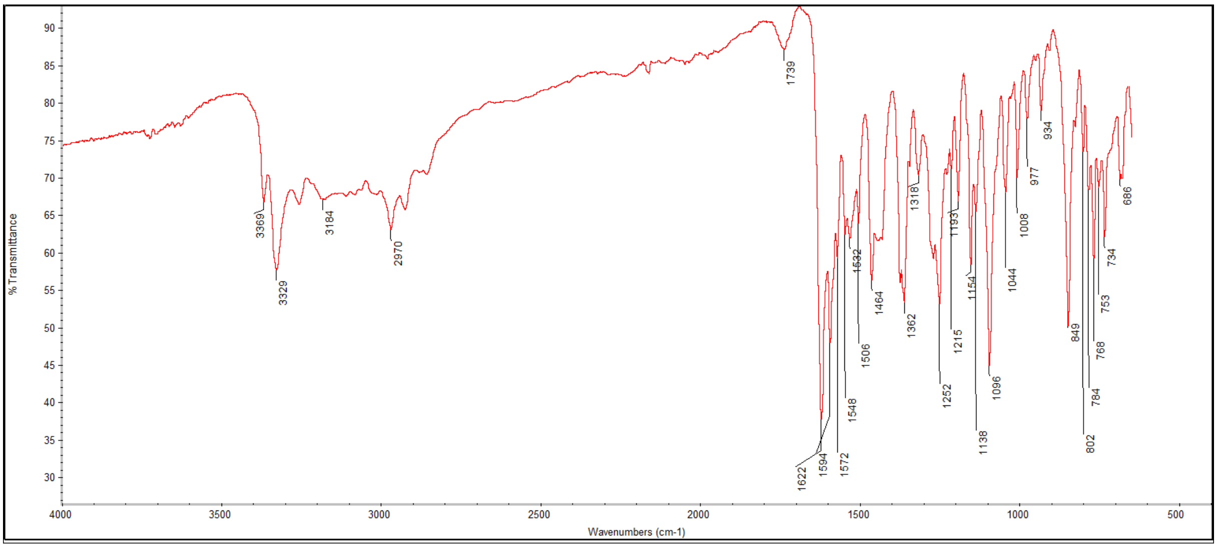

N-(6-chloro-4-methyl-3,4-dihydroquinazolin-2-yl)naphthalene-1-sulfonamide PR 69

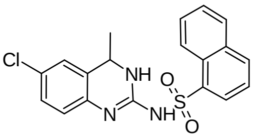

UPLC-MS:

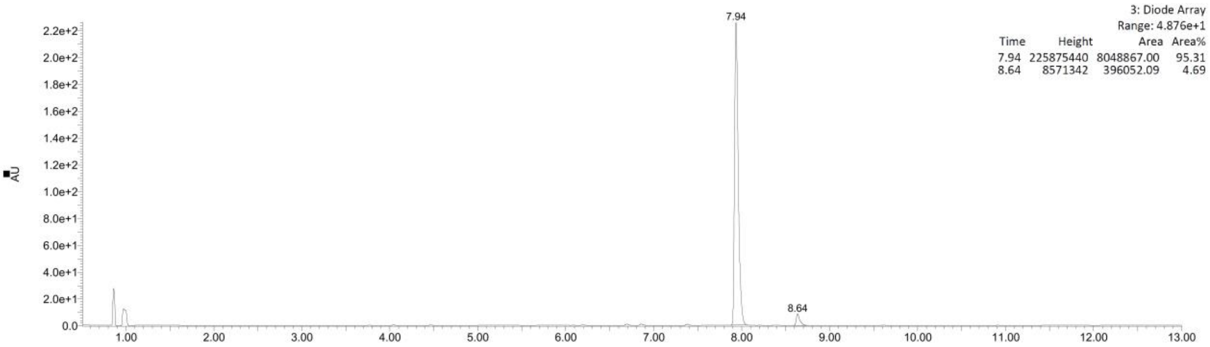

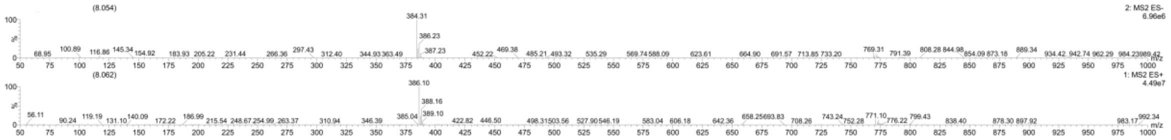

^{1}H NMR:

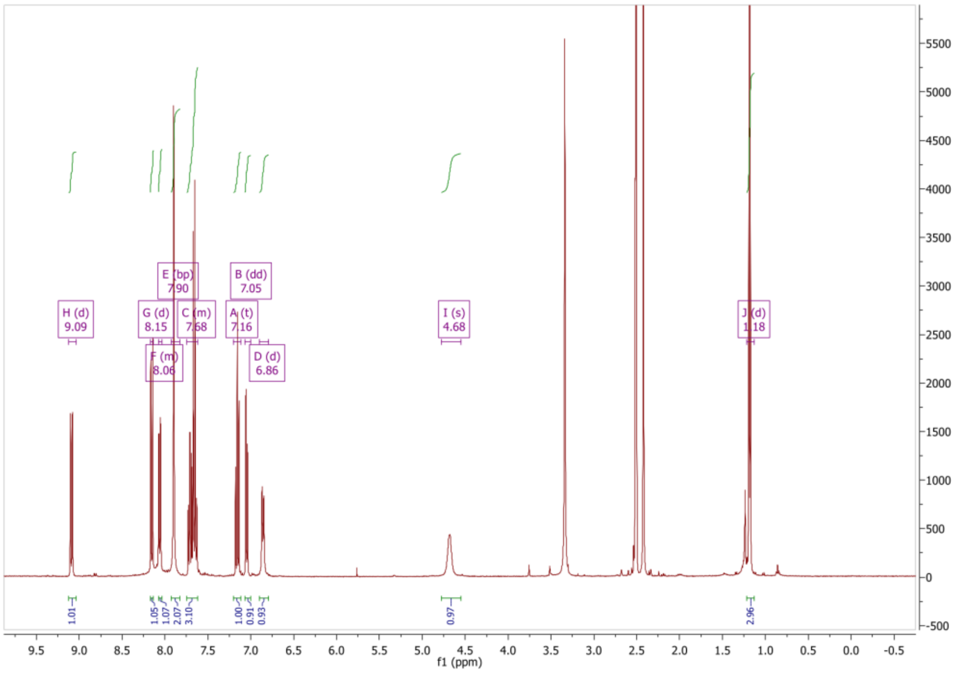

^{13}C NMR:

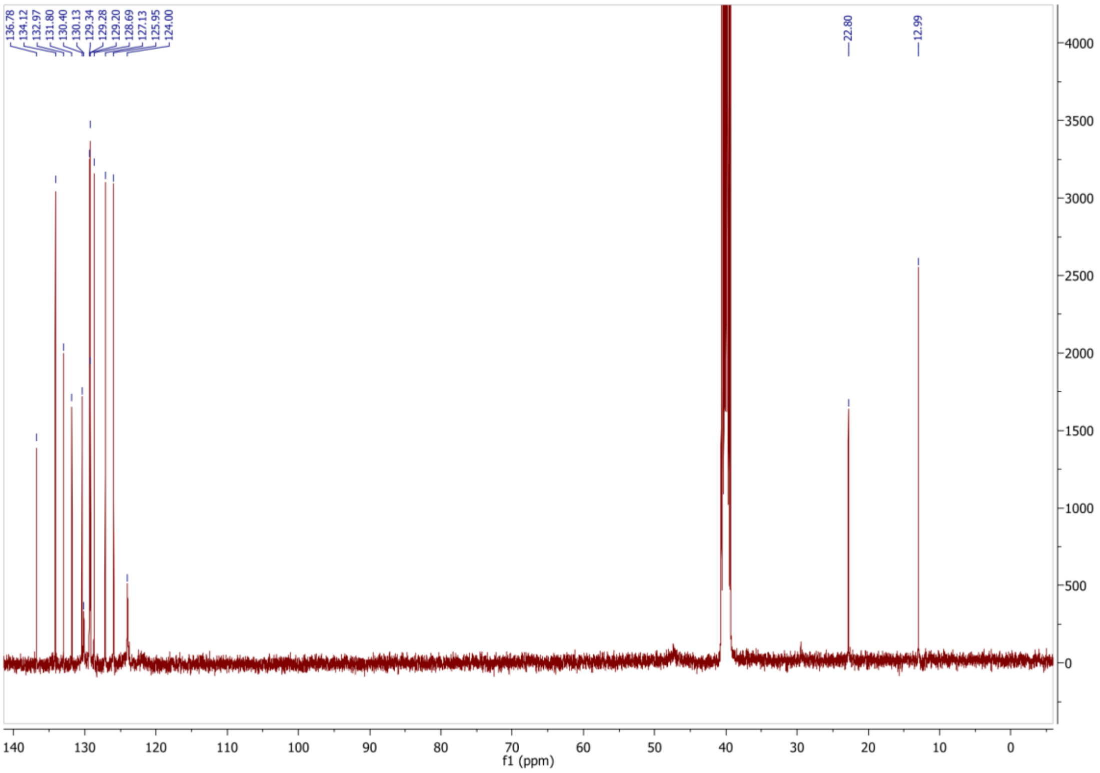

FT-IR:

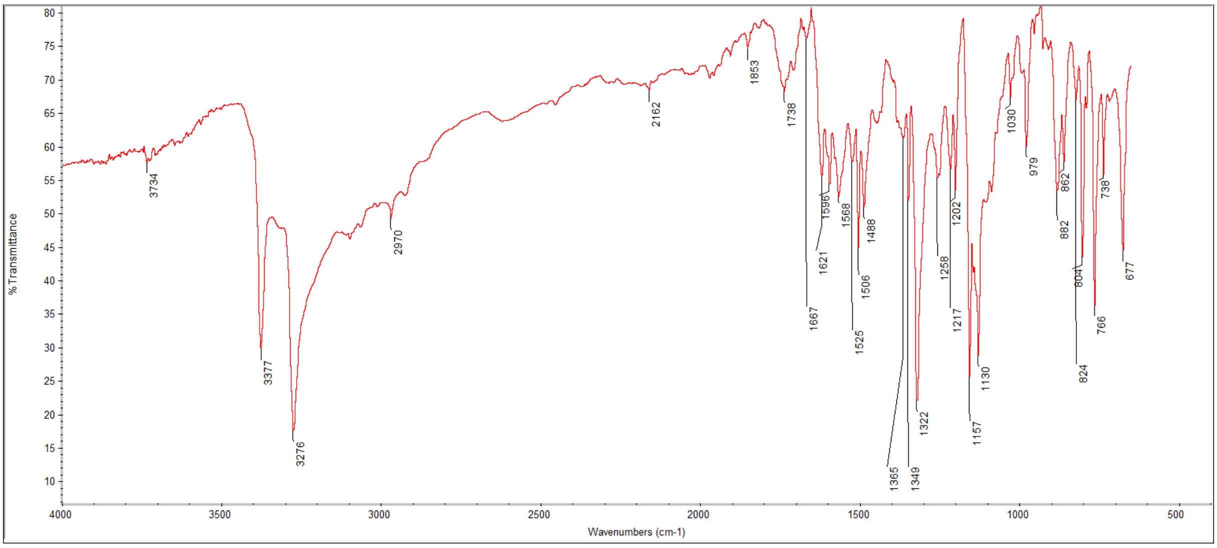

N-(7-chloro-4-methyl-3,4-dihydroquinazolin-2-yl)naphthalene-1-sulfonamide PR 70

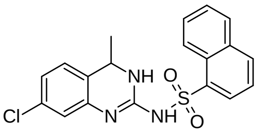

UPLC-MS:

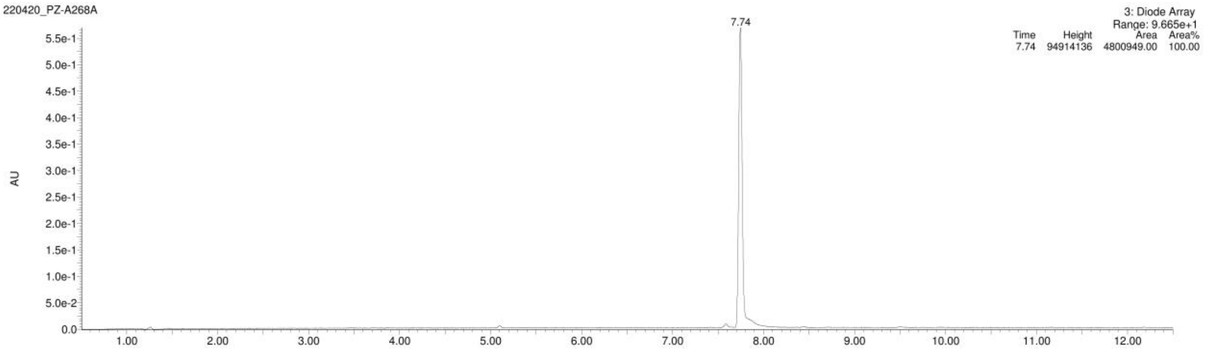

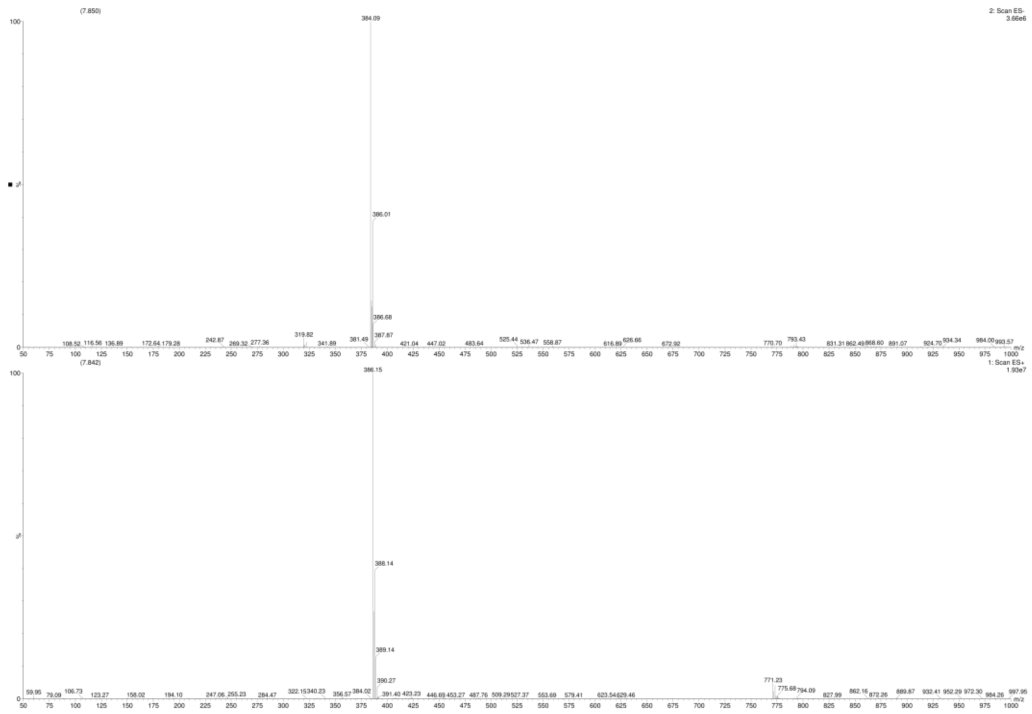

^{1}H NMR:

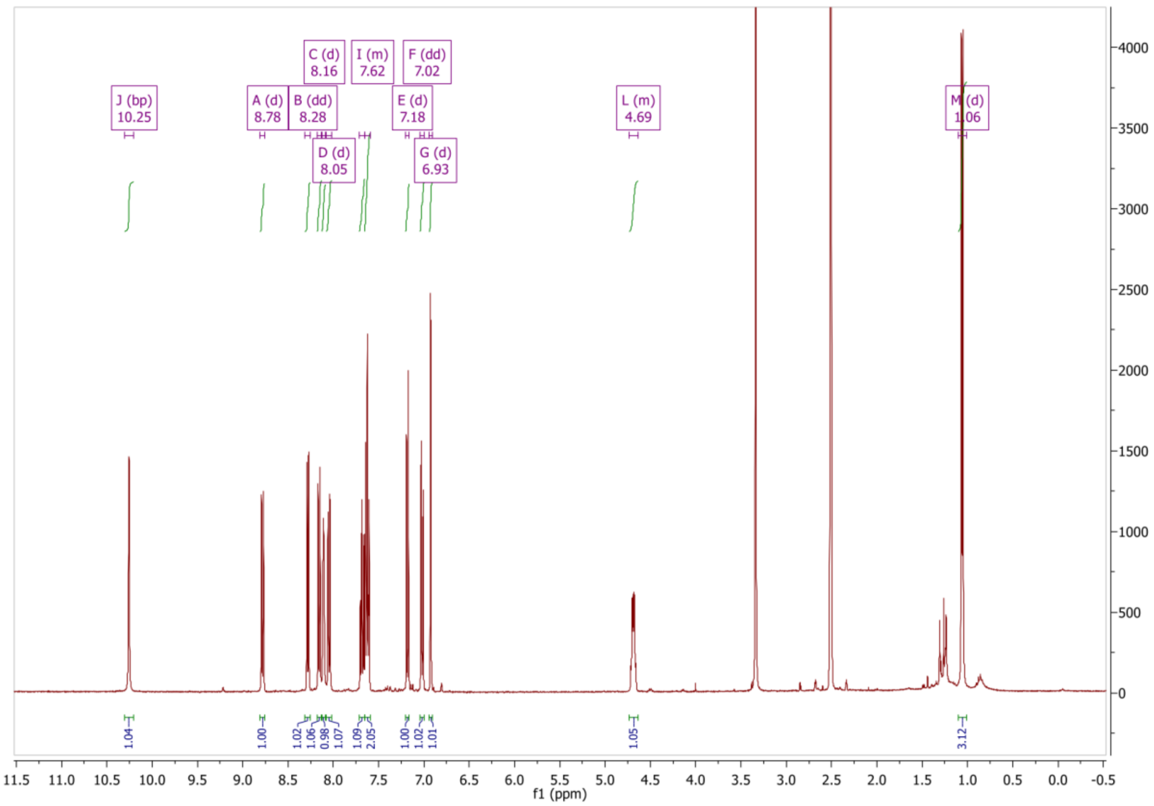

J (bp)
10.25
A (d)
8.78
B (dd)
8.28
C (d)
8.16
D (d)
8.05
I (m)
7.62
E (d)
7.18
F (dd)
7.02
G (d)
6.93
L (m)
4.69
M (d)
1.06
f1 (ppm)

^{13}C NMR:

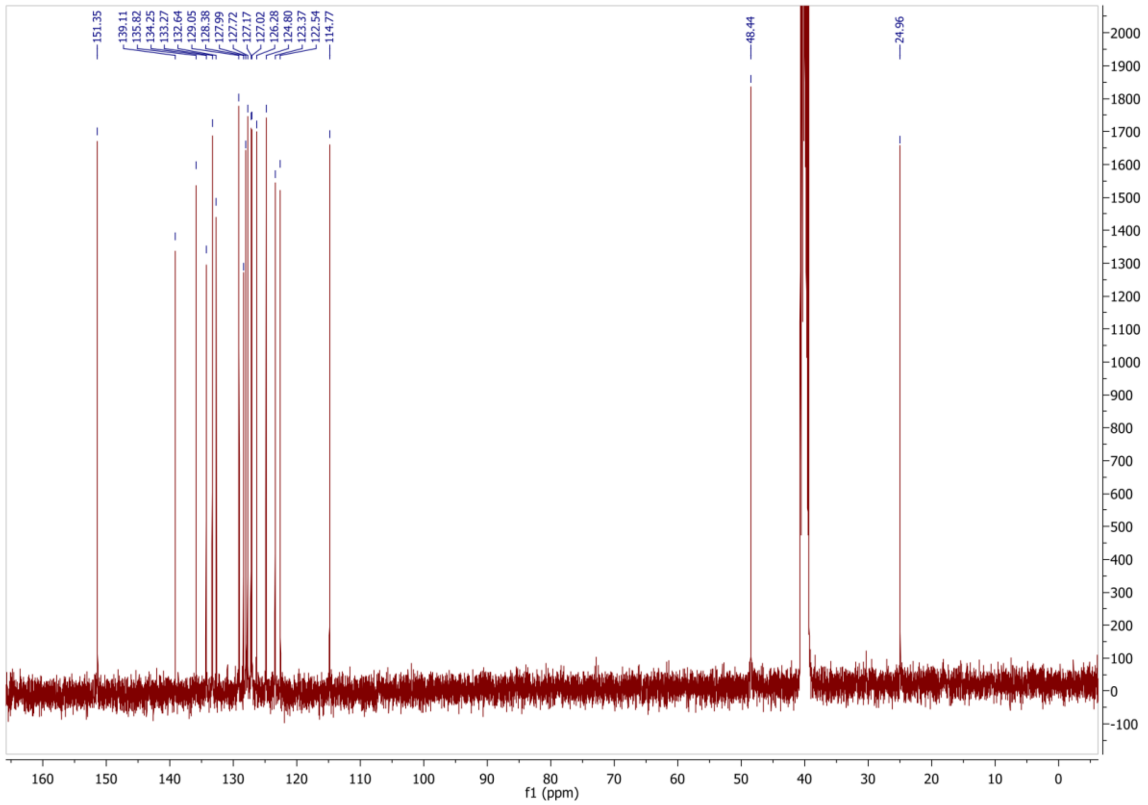

FT-IR:

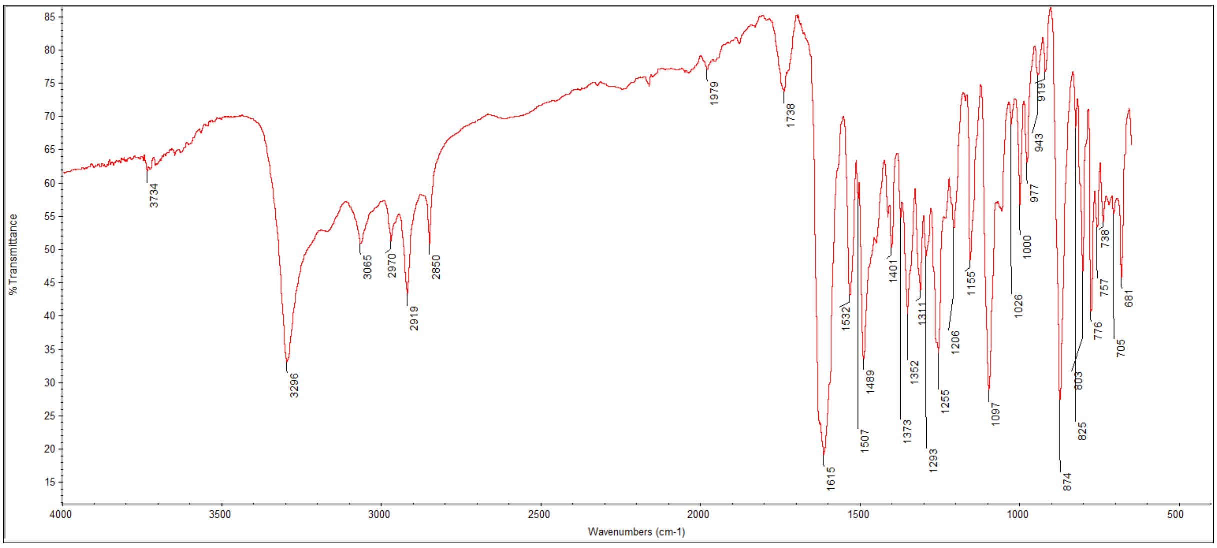

N-(8-chloro-4-methyl-3,4-dihydroquinazolin-2-yl)naphthalene-1-sulfonamide PR 71

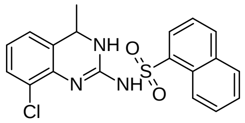

UPLC-MS:

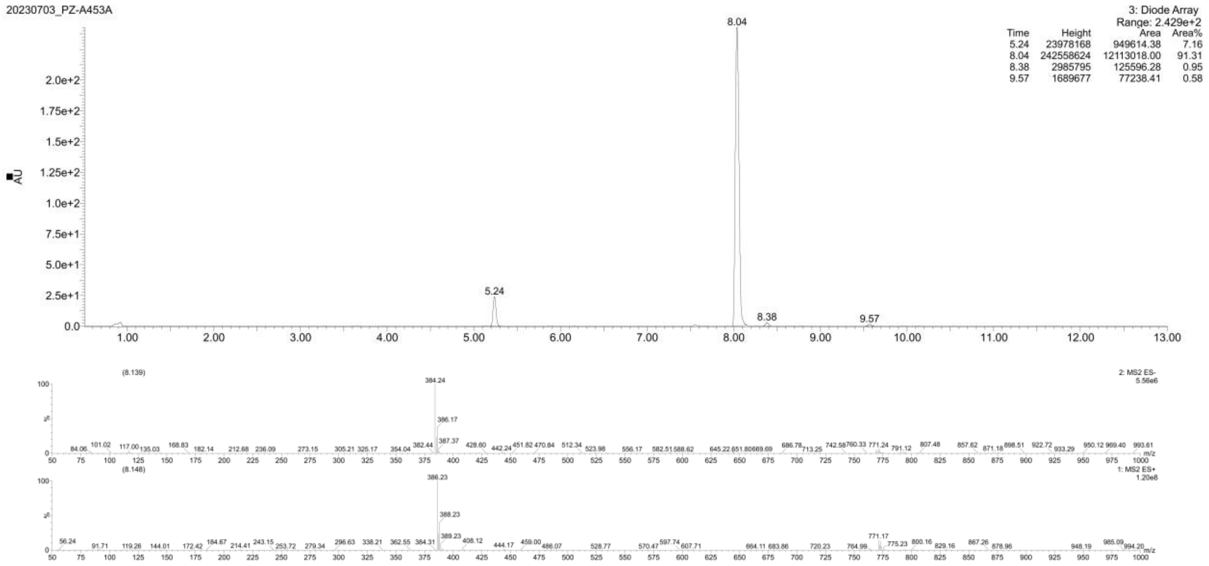
20230703_PZ-A453A
3: Diode Array
Range: 2.429e+2
Time Height Area Area%
5.24 23978168 949614.38 7.16
8.04 242558624 12113018.00 91.31
8.38 2985795 125596.28 0.95
9.57 1689677 77238.41 0.58
8.04
5.24
8.38
9.57
AU
2: MS2 ES-
5.56e6
(8.139)
384.24
386.17
1: MS2 ES+
1.20e8
(8.148)
386.23
388.23
m/z

^{1}H NMR:

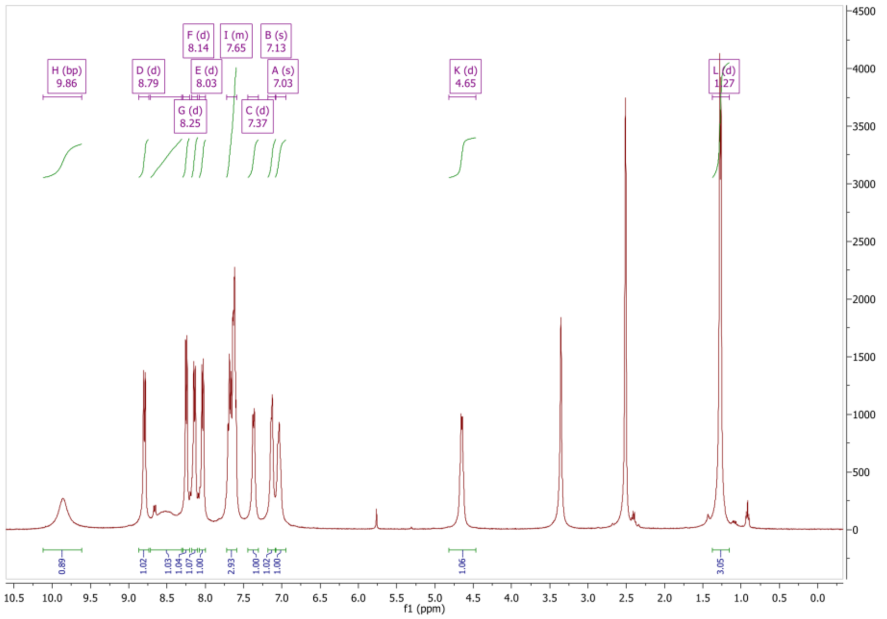
H (bp)
9.86
D (d)
8.79
F (d)
8.14
E (d)
8.03
G (d)
8.25
I (m)
7.65
C (d)
7.37
B (s)
7.13
A (s)
7.03
K (d)
4.65
L (d)
1.27
f1 (ppm)

^{13}C NMR:

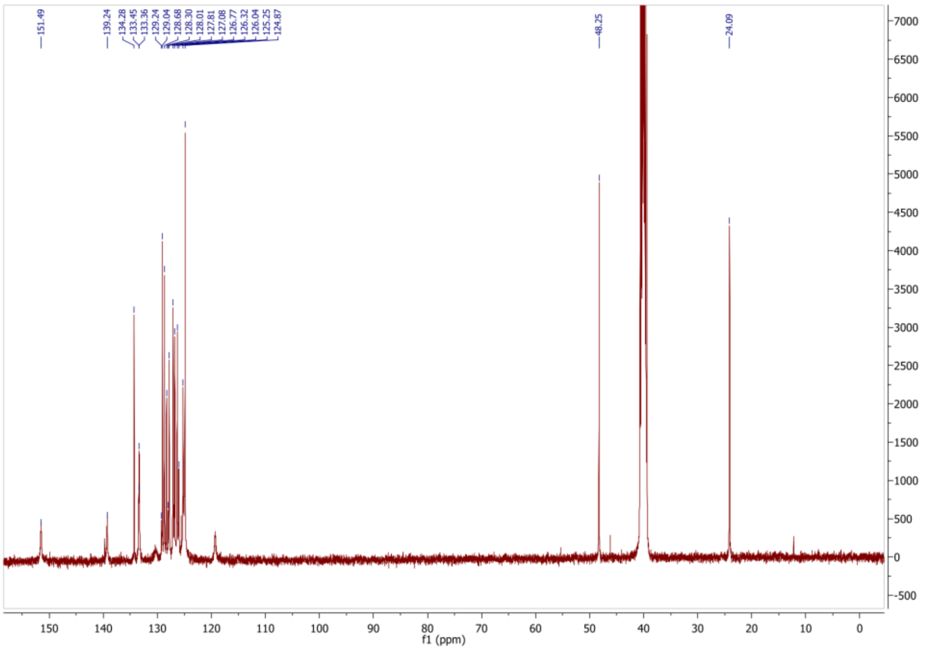

FT-IR:

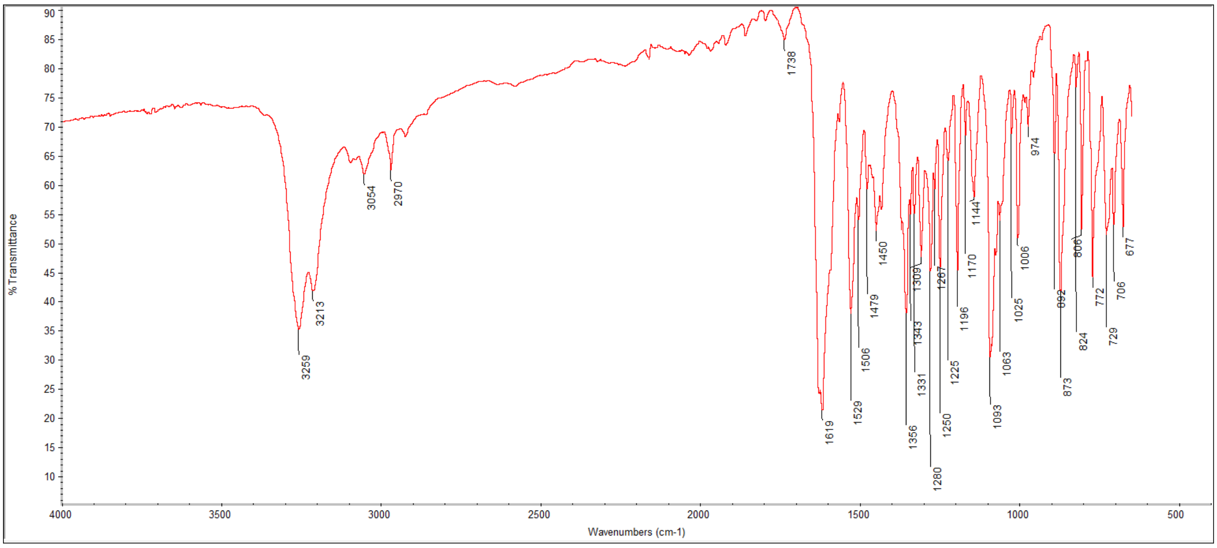

N-(6,8-dichloro-4-methyl-3,4-dihydroquinazolin-2-yl)naphthalene-1-sulfonamide PR72

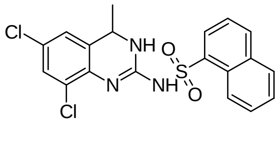

UPLC-MS:

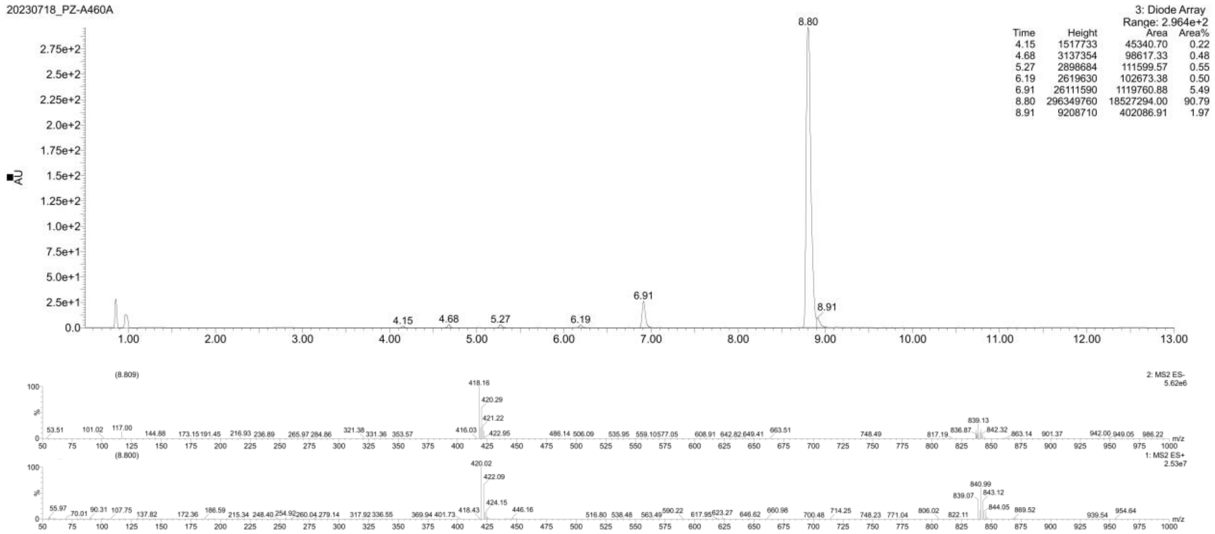

^{1}H NMR:

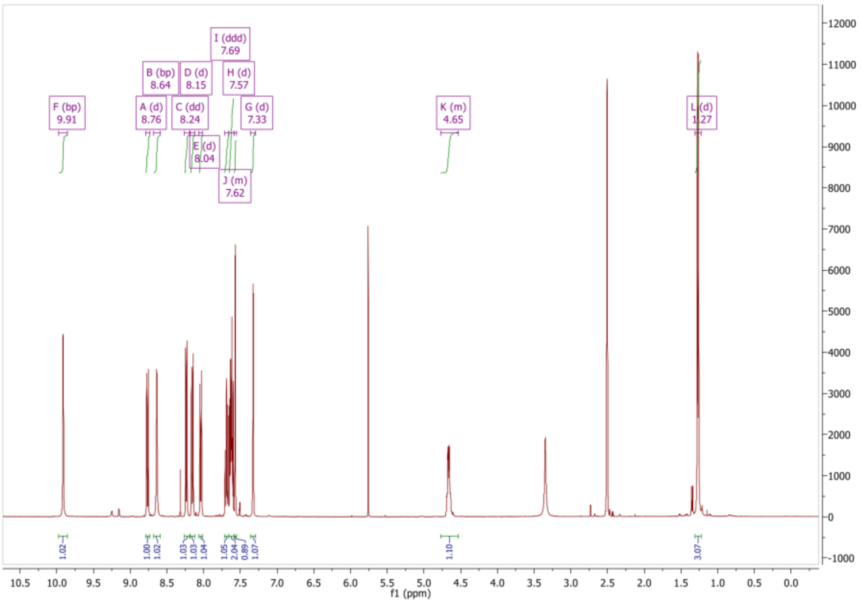

^{13}C NMR:

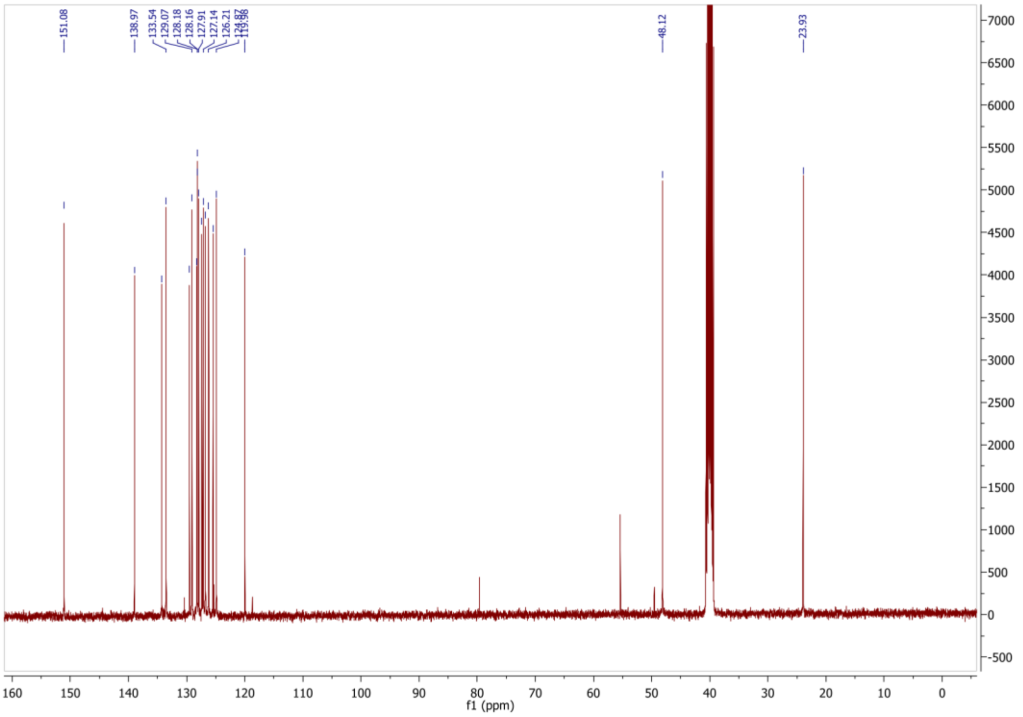

FT-IR:

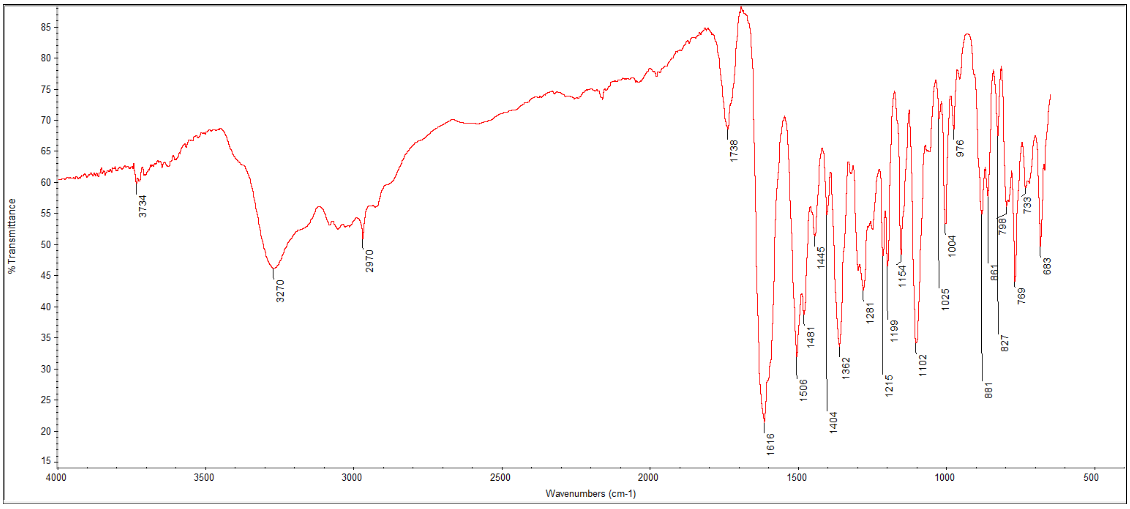

N-(5-fluoro-4-methyl-3,4-dihydroquinazolin-2-yl)naphthalene-1-sulfonamide PR 73

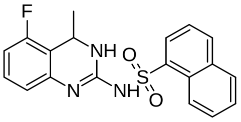

UPLC-MS:

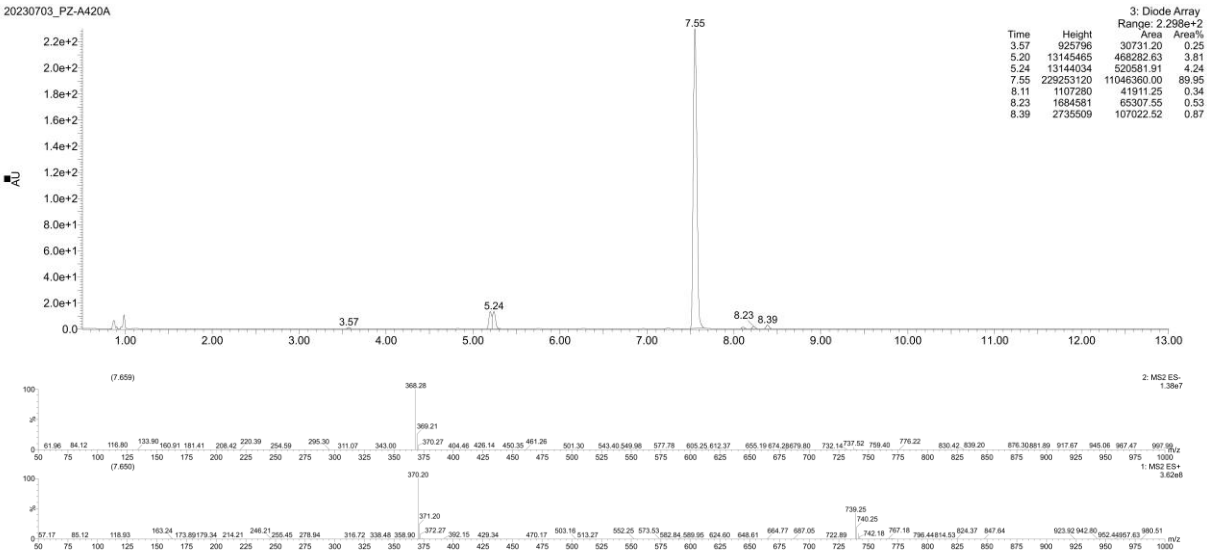

^{1}H NMR:

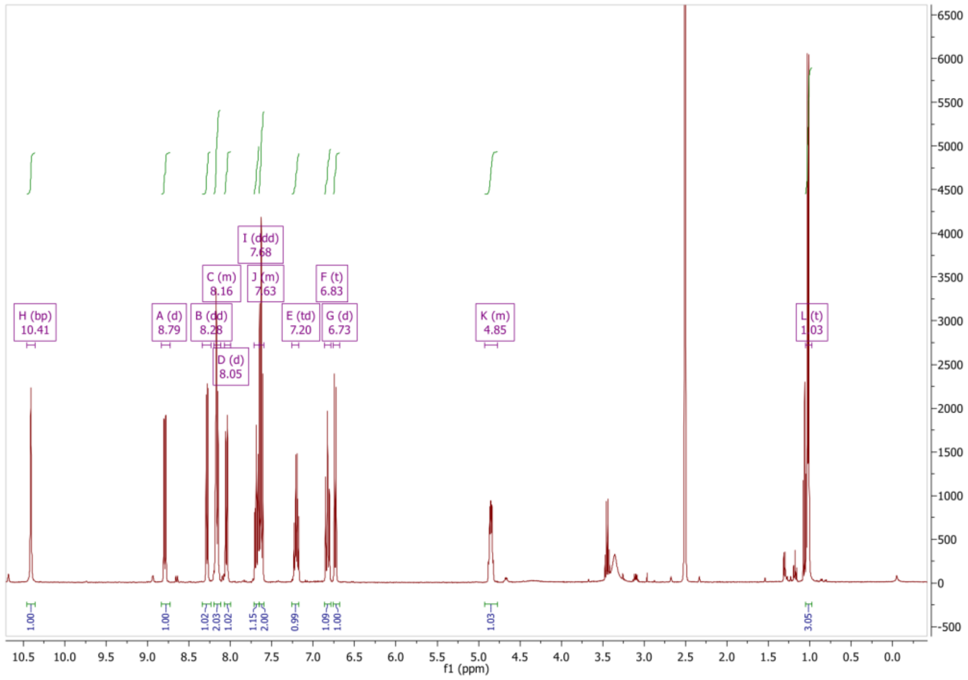

^{13}C NMR:

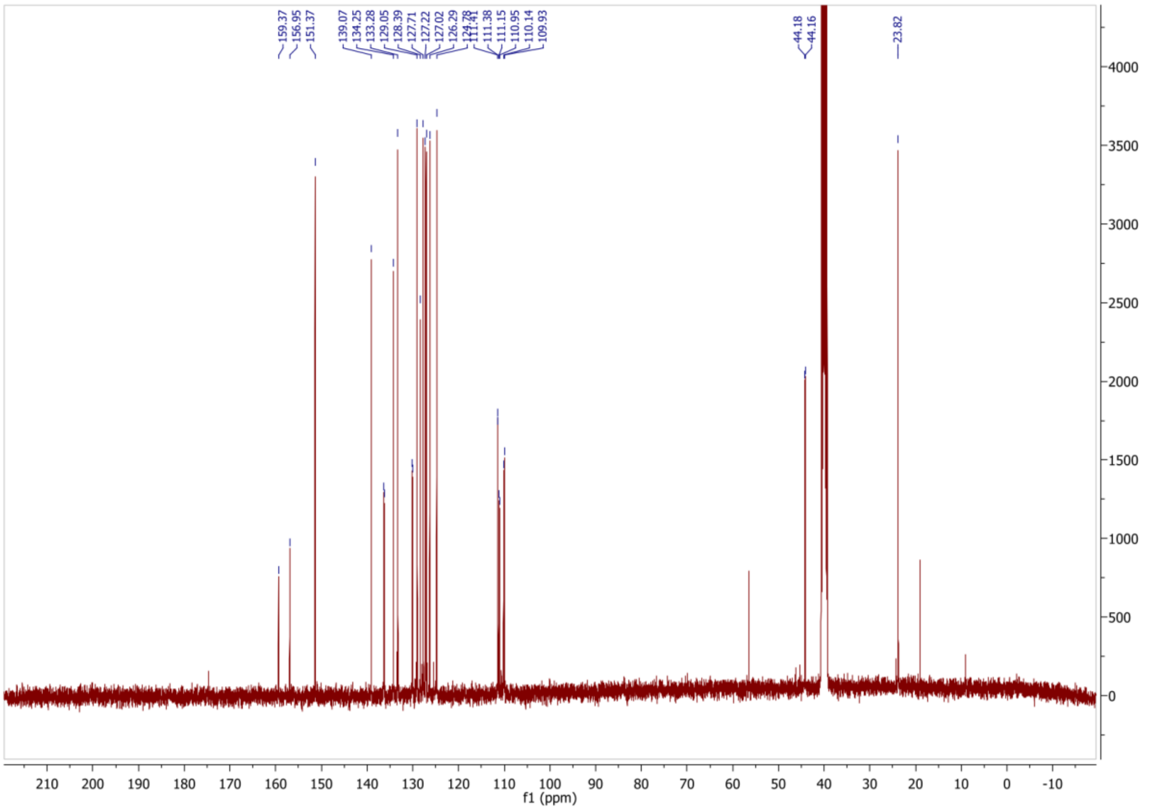

FT-IR:

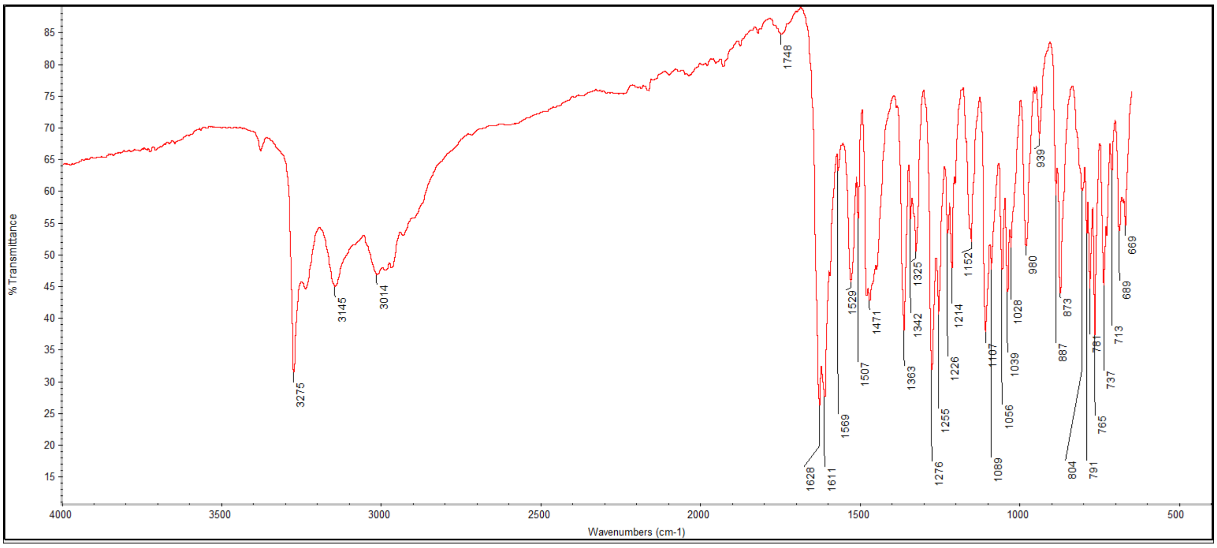

4-chloro-*N*-(6,8-dichloro-4-methyl-3,4-dihydroquinazolin-2-yl)naphthalene-1-sulfonamide PR 74

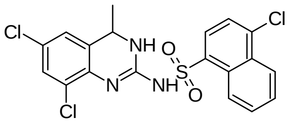

UPLC-MS:

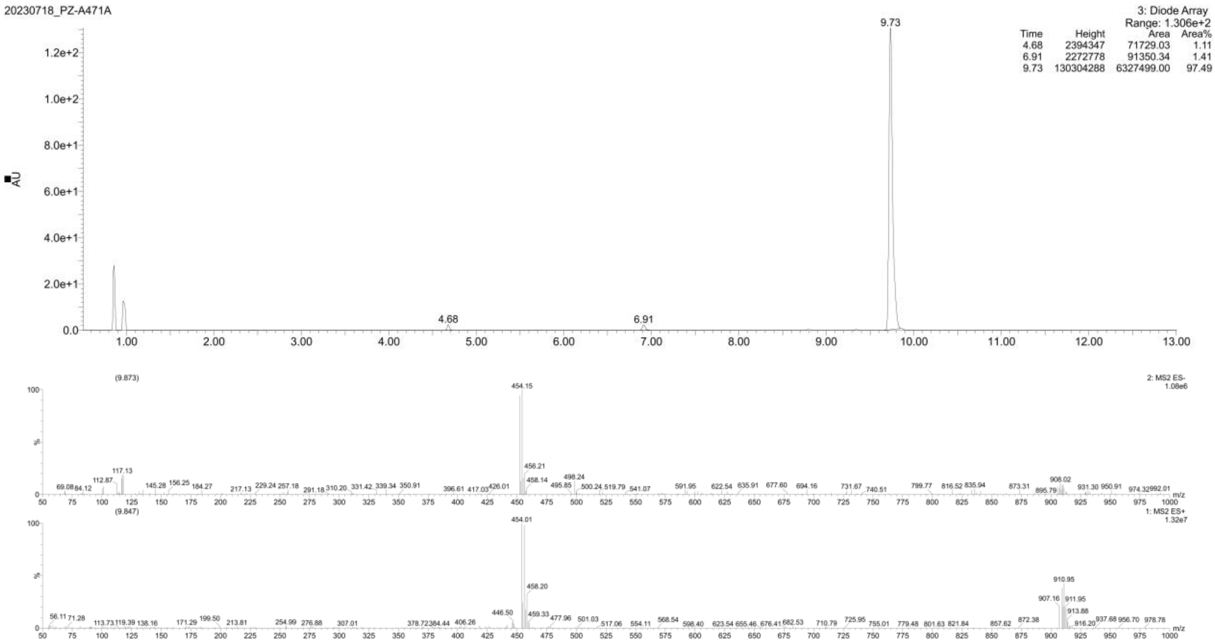

^{1}H NMR:

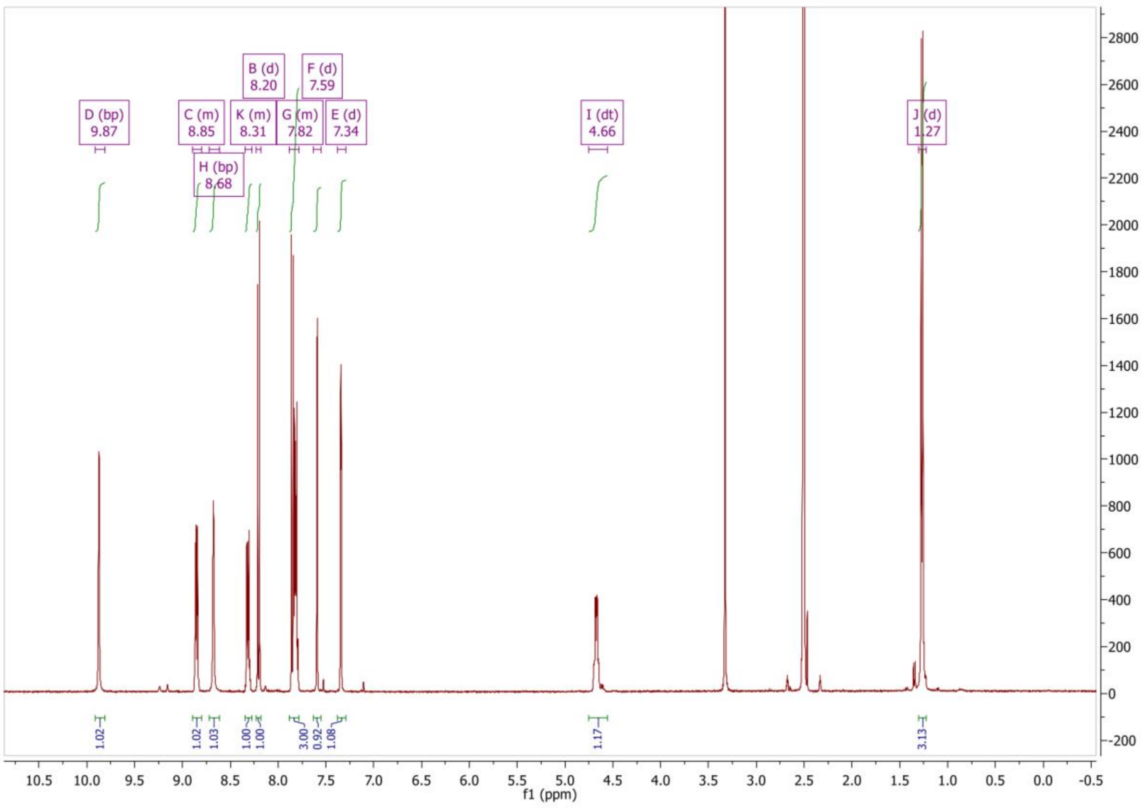

^{13}C NMR:

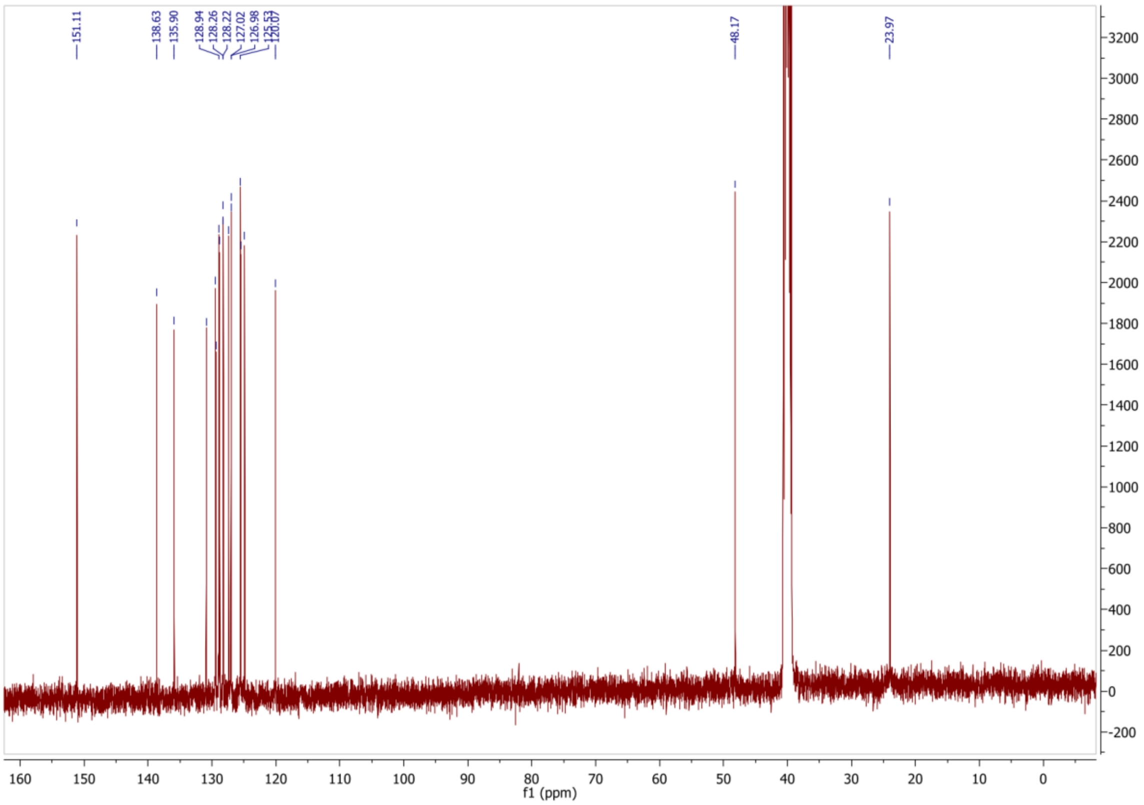

FT-IR:

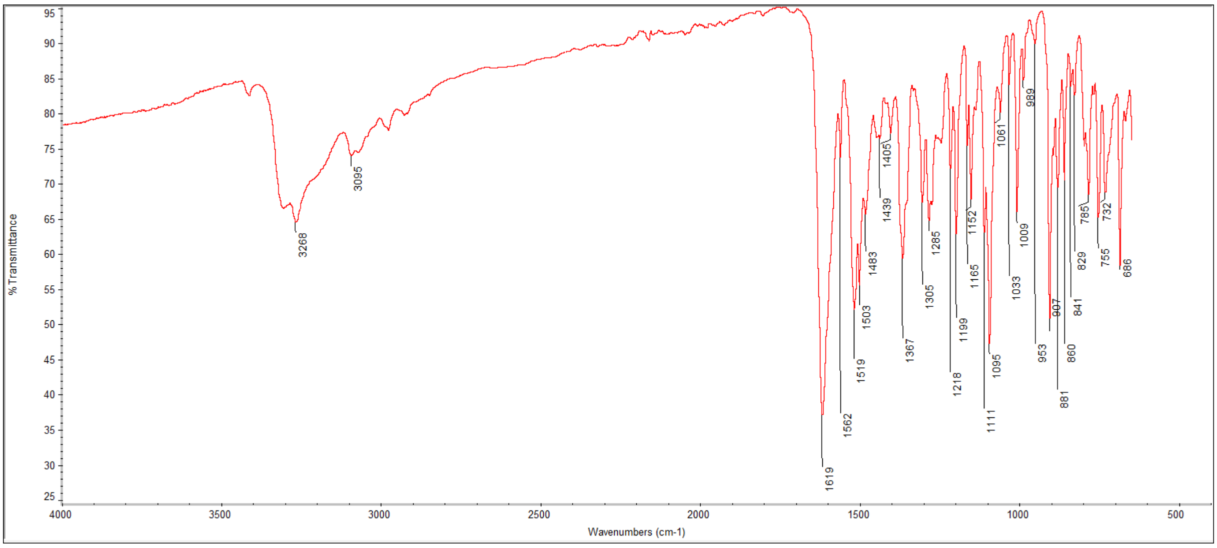

N-(6-bromo-4-methyl-3,4-dihydroquinazolin-2-yl)naphthalene-1-sulfonamide PR 75

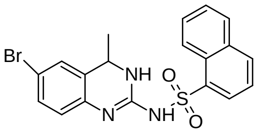

UPLC-MS:

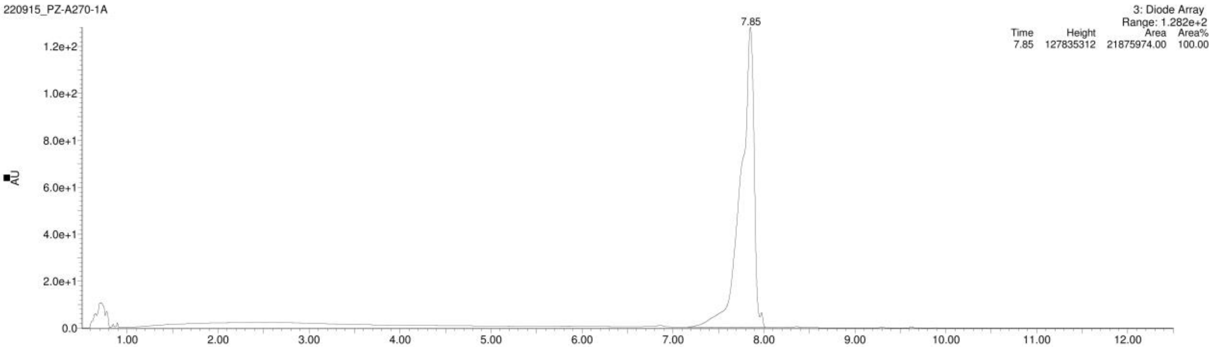

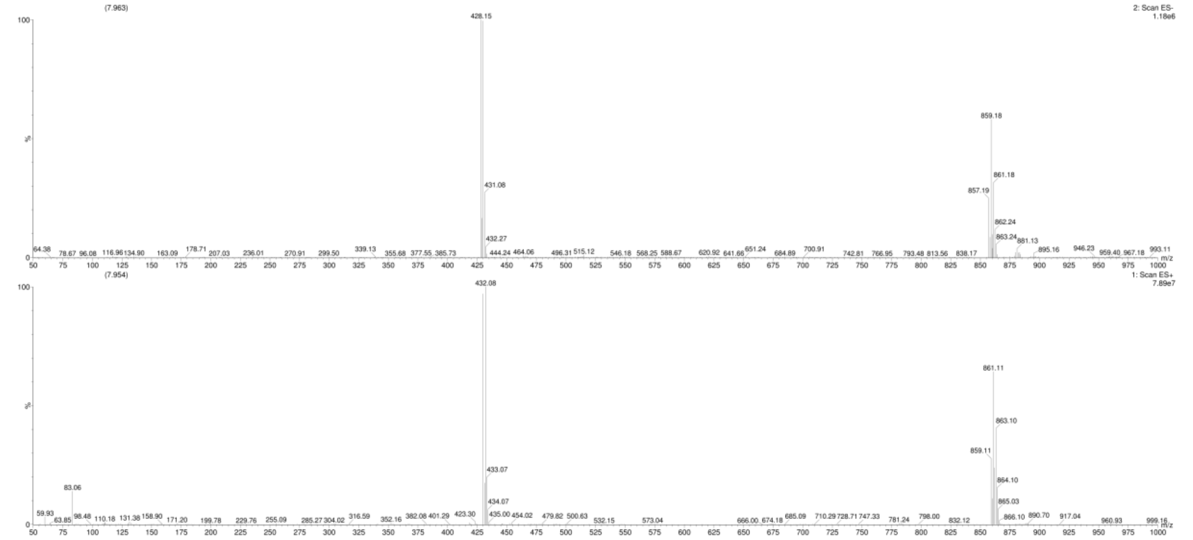

****

^{1}H NMR:

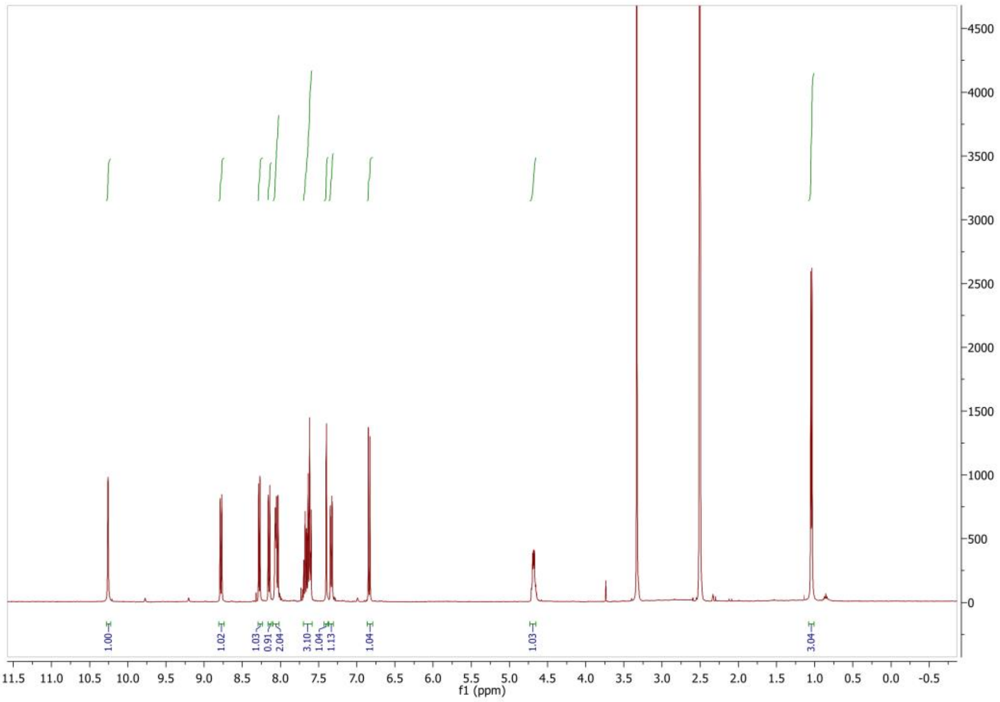

^{13}C NMR:

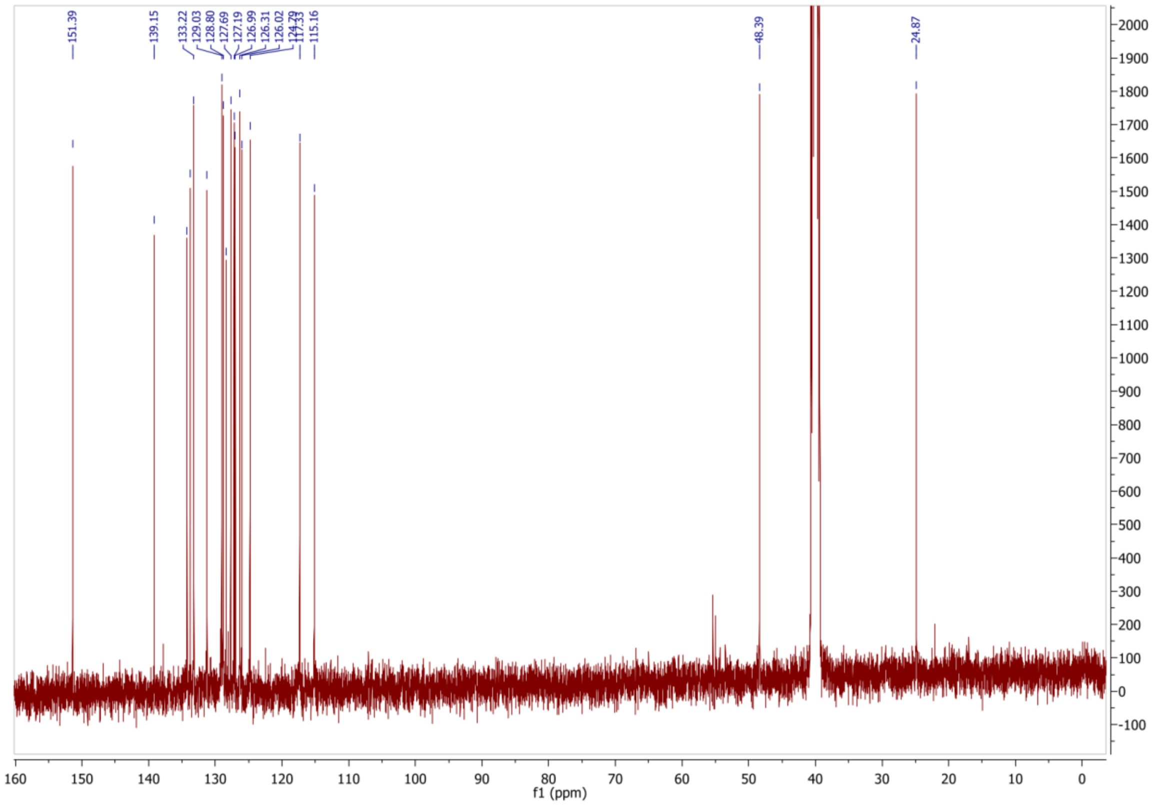

FT-IR:

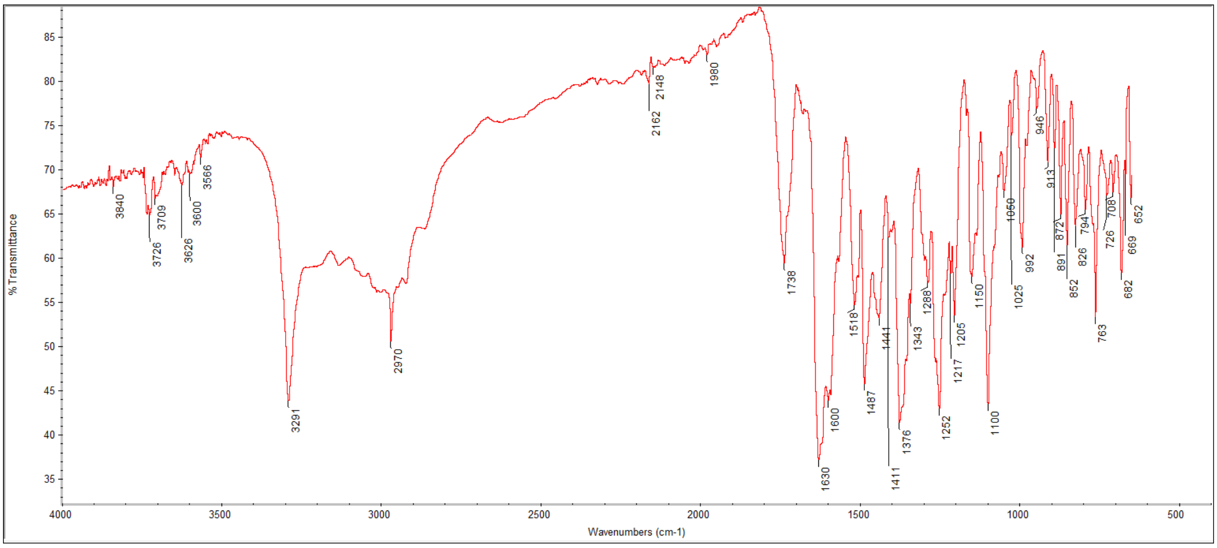

N-(3,4-dimethyl-3,4-dihydroquinazolin-2-yl)naphthalene-1-sulfonamide PR 76

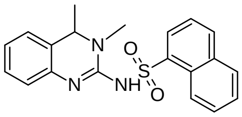

UPLC-MS:

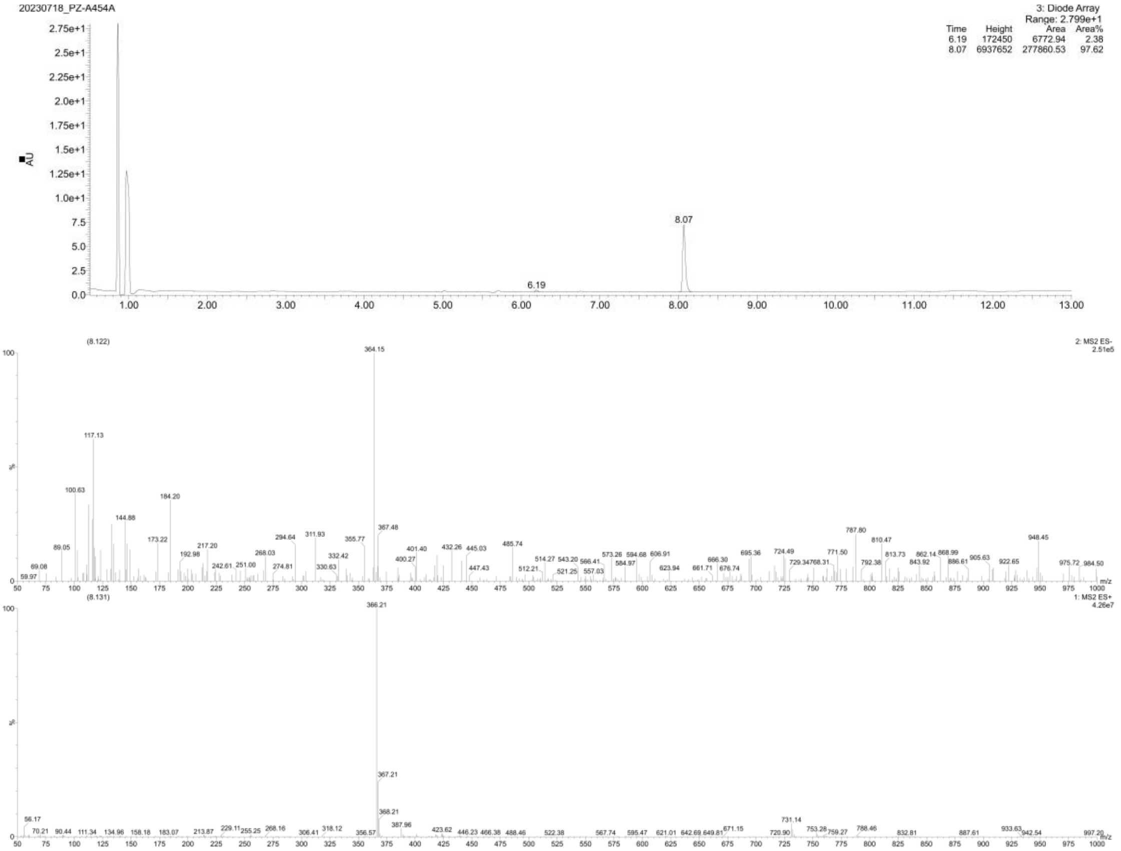

^{1}H NMR:

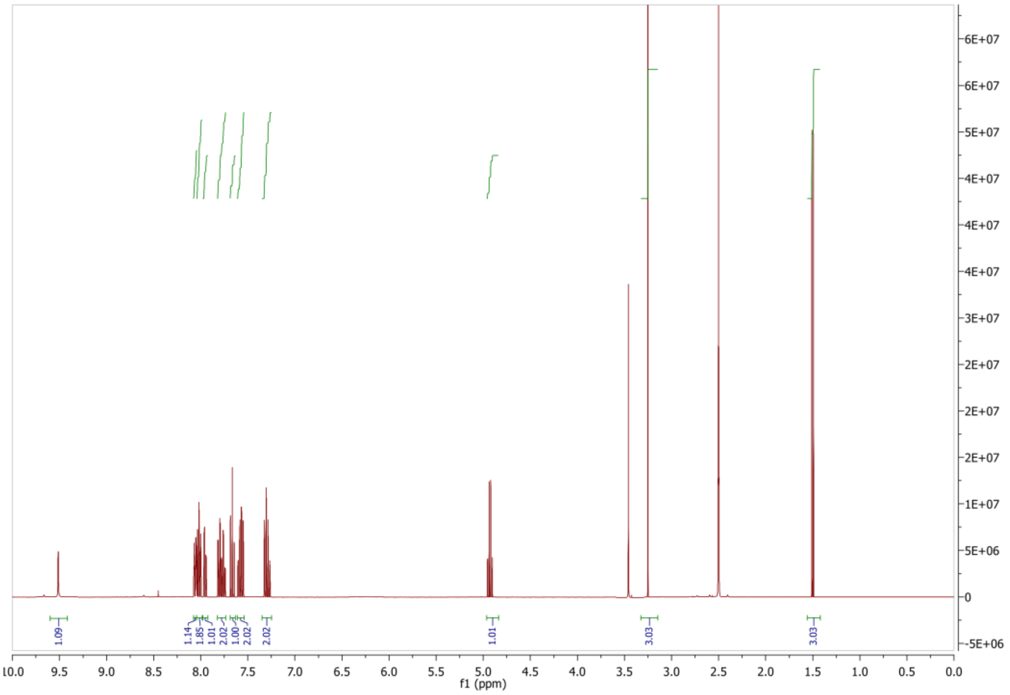

^{13}C NMR:

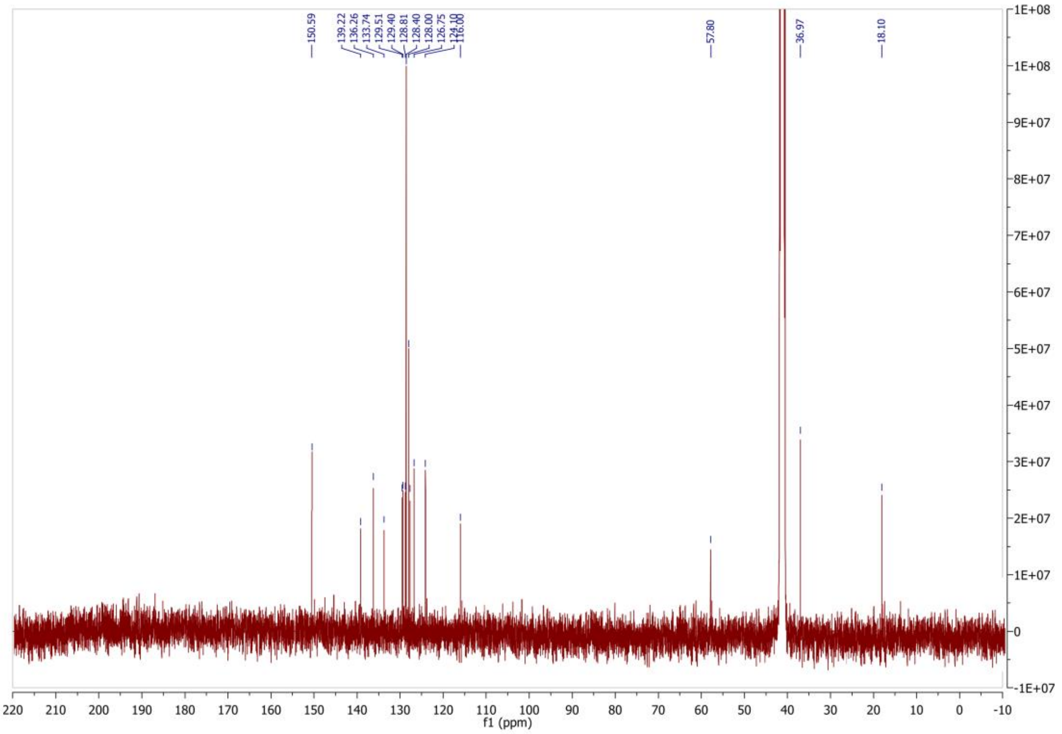

FT-IR:

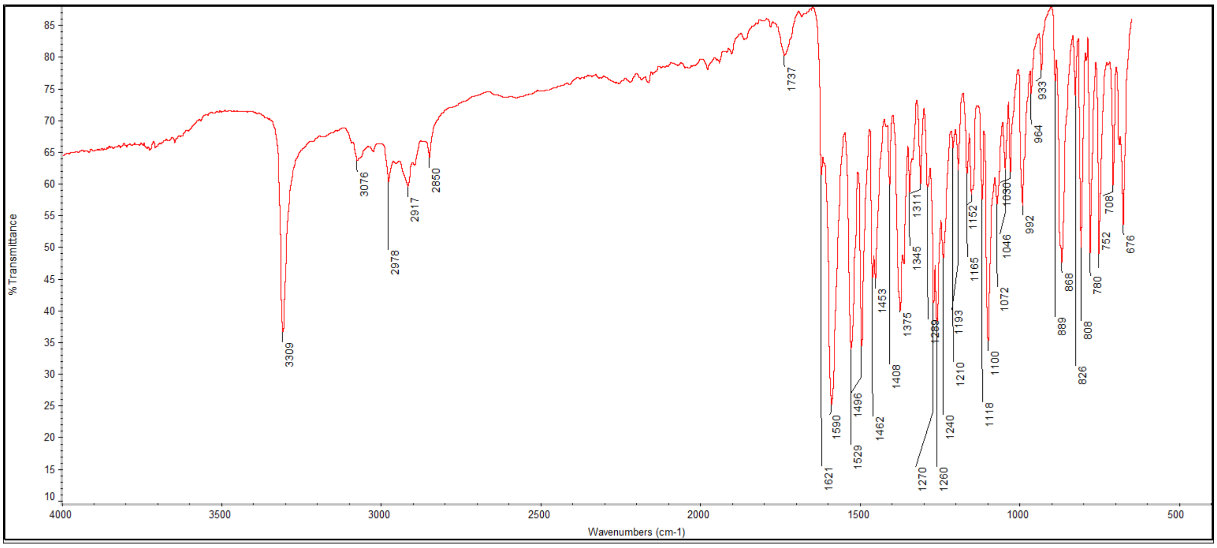

N-(4-methyl-4H-3,1-benzoxazin-2-yl)naphthalene-1-sulfonamide PR 77

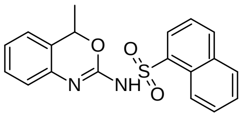

UPLC-MS:

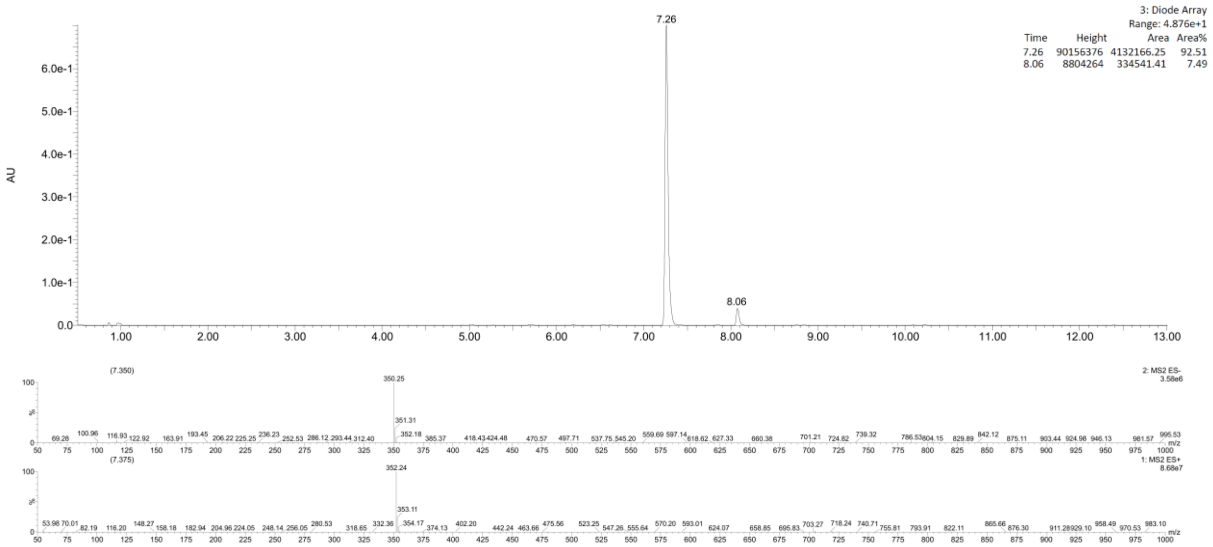

^{1}H NMR:

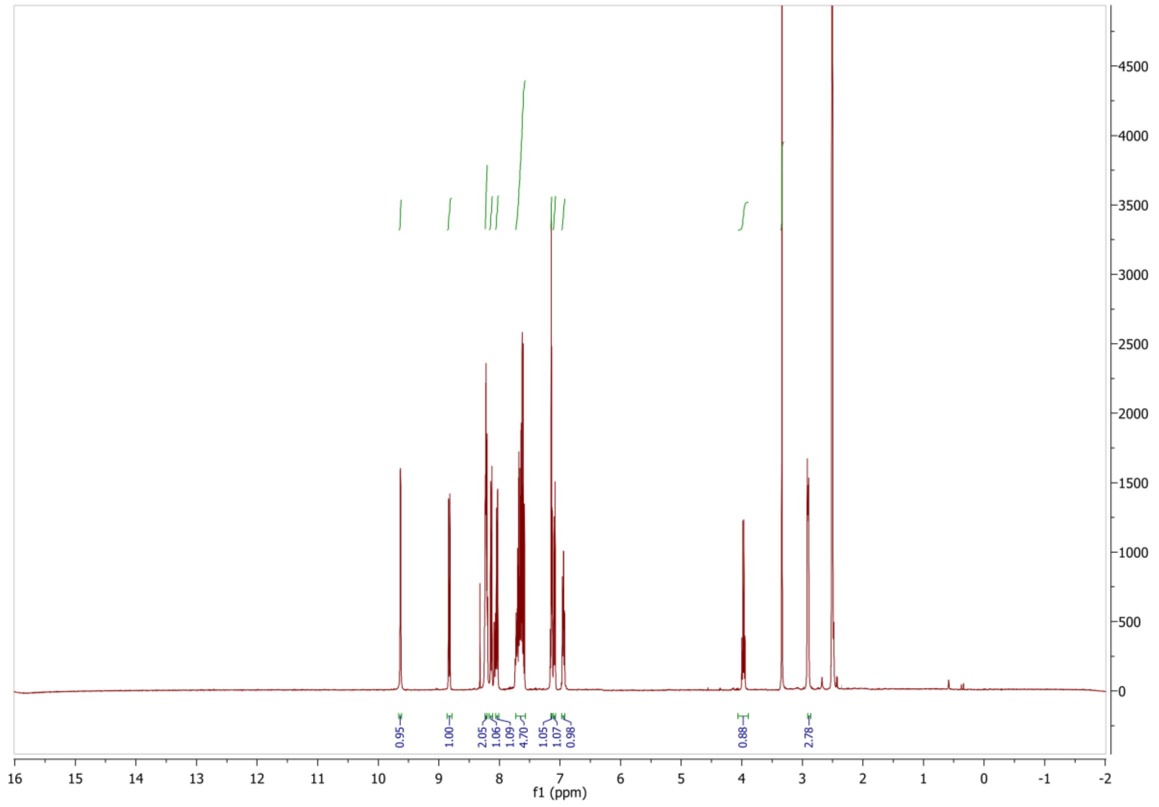

^{13}C NMR:

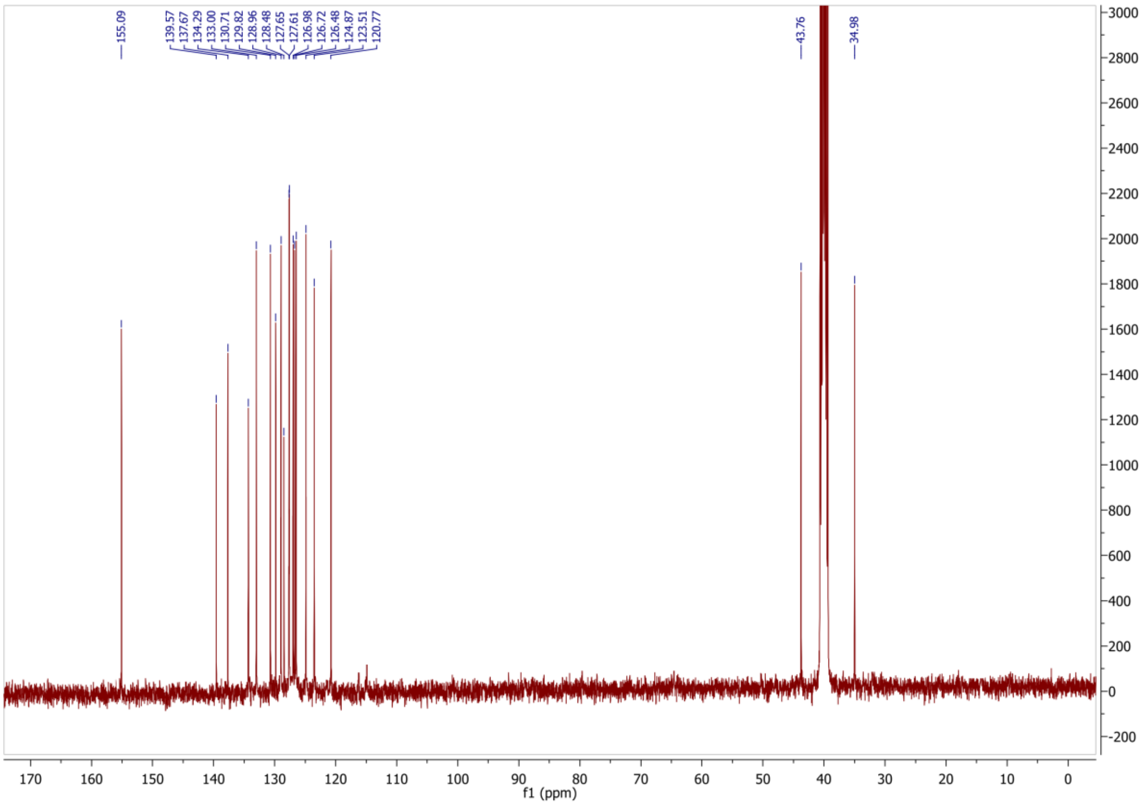

FT-IR:

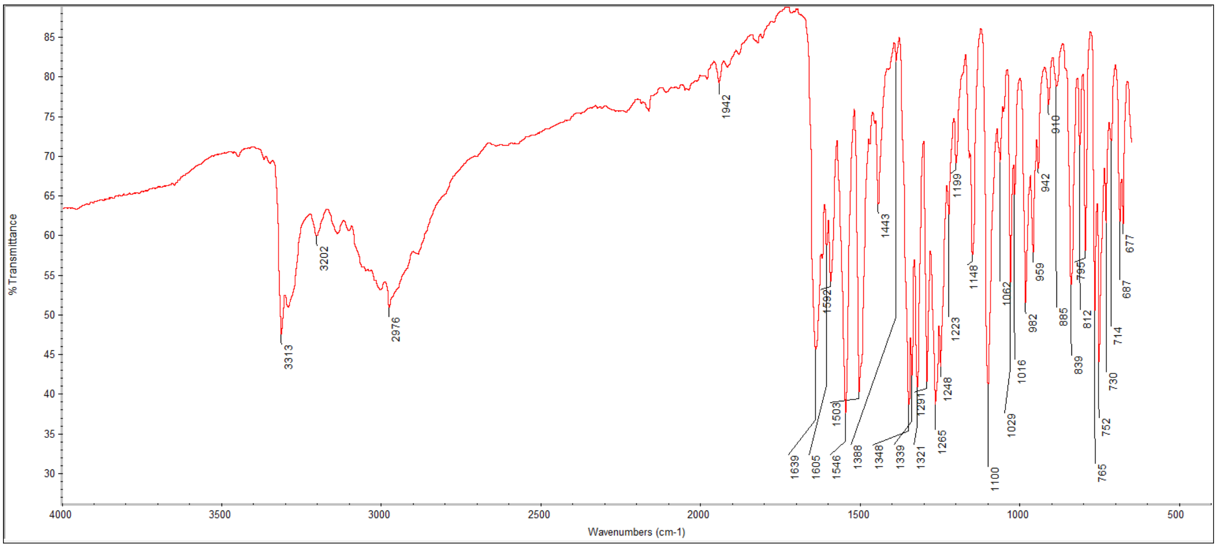

N-(4,5-dihydro-3H-1,3-benzodiazepin-2-yl)naphthalene-1-sulfonamide PR 78

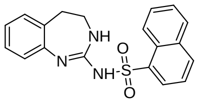

UPLC-MS:

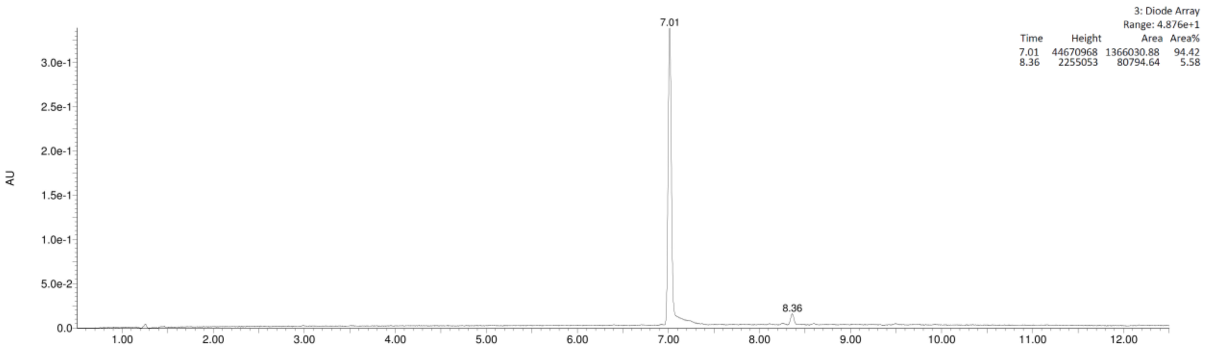

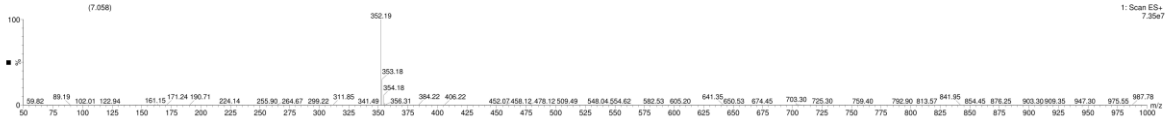

^{1}H NMR:

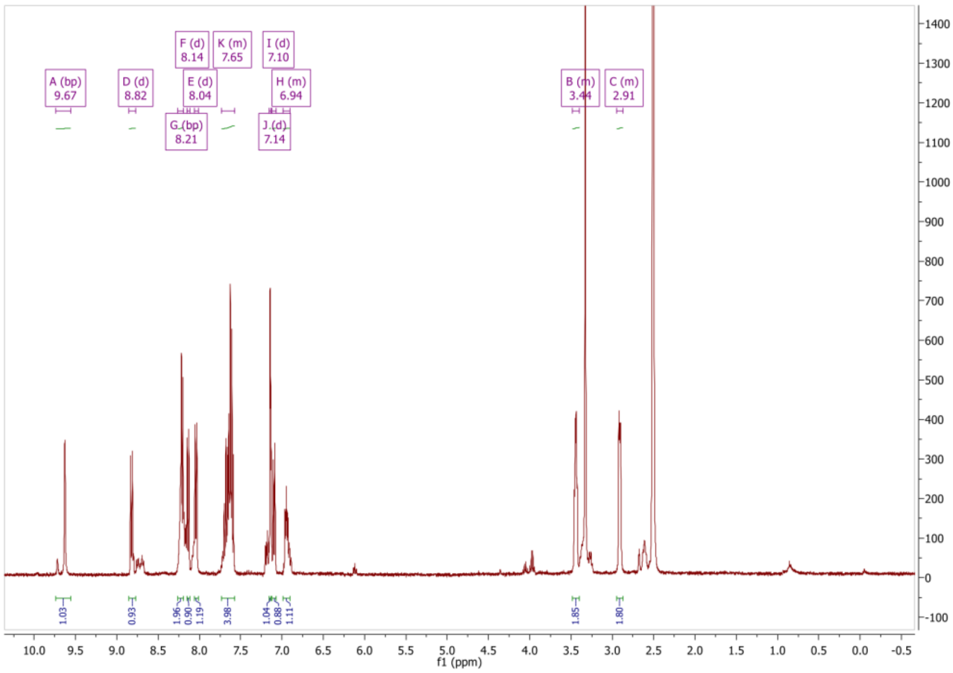

^{13}C NMR:

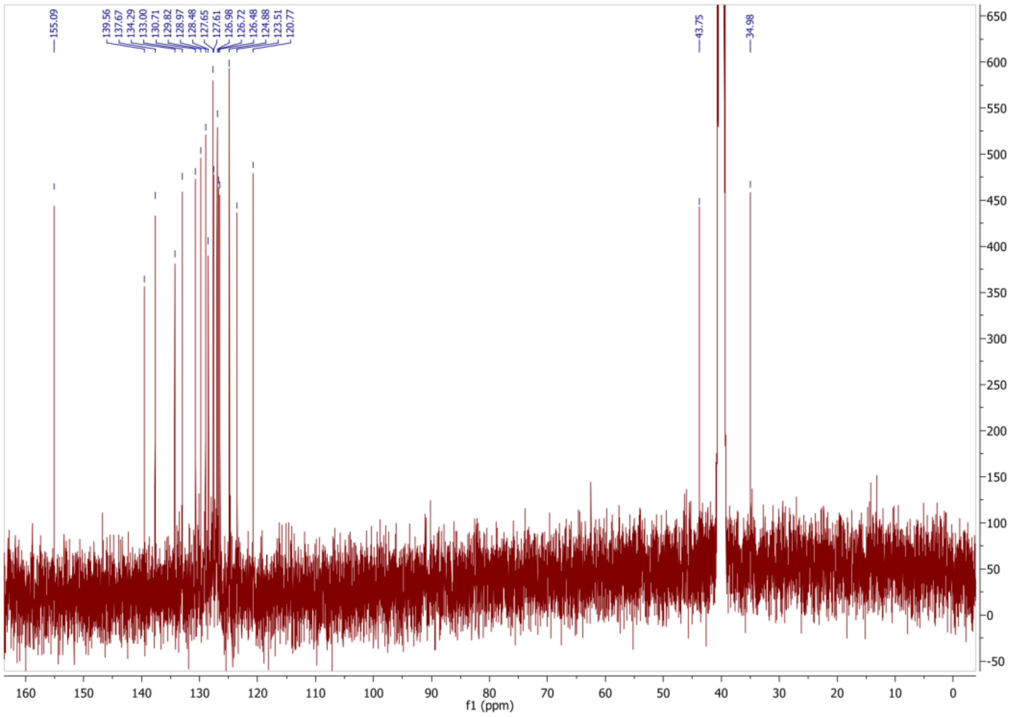
155.09
139.56
137.67
134.29
133.00
130.71
129.82
128.97
128.48
127.65
127.61
126.98
126.72
126.48
124.88
123.51
120.77
43.75
34.98
f1 (ppm)

FT-IR:

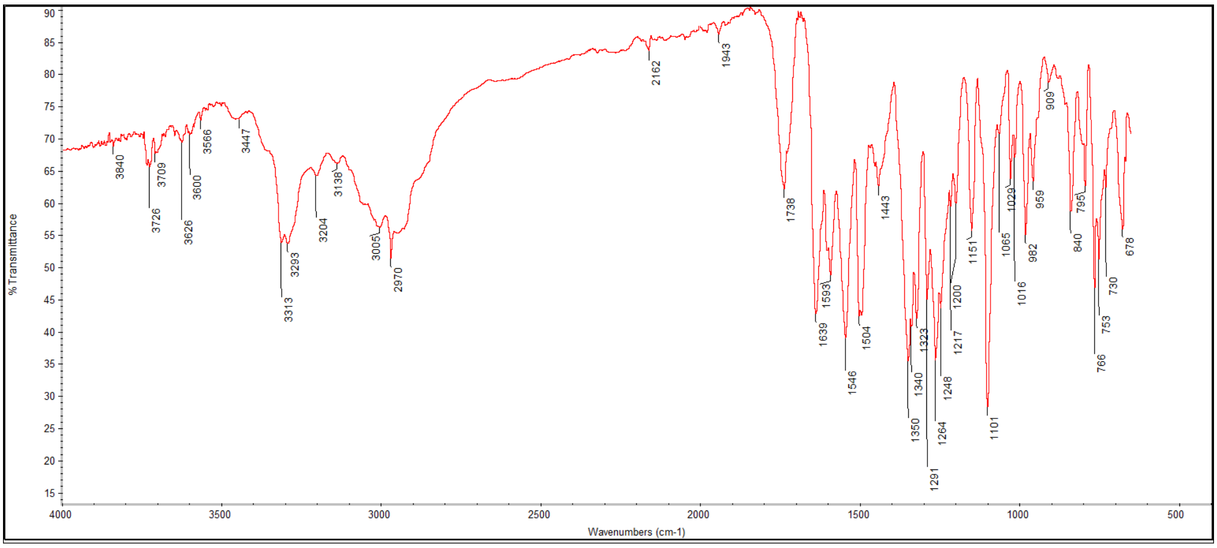
% Transmittance
Wavenumbers (cm-1)
3840
3726
3709
3626
3600
3566
3447
3313
3293
3204
3138
3005
2970
2162
1943
1738
1639
1593
1546
1504
1443
1350
1340
1323
1291
1264
1248
1217
1200
1151
1101
1065
1029
1016
982
959
909
840
795
766
753
730
678
