# Supplementary material for: Low-Basicity 5-HT6 Receptor Ligands from the Group of Cyclic Arylguanidine Derivatives and Their Antiproliferative Activity Evaluation
Source: Int J Mol Sci. 2024 Sep 24;25(19):10287. doi: 10.3390/ijms251910287 (PMC11477289; doi:10.3390/ijms251910287)
Supplement: Supplementary file 1 [file ijms-25-10287-s001.zip › SI_Table1.pdf]

		C18 at pH 2.6						C18 at pH 7.4						C18 at pH 10.5						IAM						HSA							
L.p.	Name	t1	t2	t3	tm	SD	CHI C18	t1	t2	t3	tm	SD	CHI C18	t1	t2	t3	tm	SD	CHI C18	t1	t2	t3	tm	SD	CHI IAM	t1	t2	t3	tm	SD	log(t)	logKHSA	%HSA
1	PR 68	3.86	3.95	3.95	3.92	0.0488	101.4	3.84	3.87	3.88	3.86	0.021	99.2	3.80	3.89	3.84	3.85	0.046	103.0	4.58	4.59	4.59	4.59	0.006	45.8	17.38	17.34	17.38	17.37	0.03	1.24	1.5	97.7
2	PR 73	3.71	3.77	3.70	3.73	0.0389	94.6	3.75	3.75	3.72	3.74	0.021	94.9	3.67	3.67	3.67	3.67	0.003	96.5	4.36	4.34	4.36	4.35	0.010	42.3	14.92	14.97	14.97	14.95	0.03	1.17	1.3	96.6
3	PR 71	3.96	3.97	3.89	3.94	0.0424	102.2	3.88	3.90	3.95	3.91	0.037	100.7	3.87	3.86	3.86	3.87	0.003	103.8	4.53	4.52	4.52	4.52	0.004	44.8	16.84	16.85	16.84	16.84	0.01	1.23	1.4	97.5
4	PR 77	3.94	3.96	3.95	3.95	0.0112	102.5	3.87	3.88	3.90	3.88	0.012	99.9	3.86	3.84	3.85	3.85	0.009	103.2	4.26	4.25	4.26	4.26	0.004	40.8	13.79	13.78	13.78	13.79	0.01	1.14	1.3	95.9
5	PR 9	3.20	3.15	3.18	3.18	0.0243	75.1	3.28	3.29	3.29	3.29	0.005	79.2	3.23	3.21	3.19	3.21	0.018	79.6	3.92	3.92	3.92	3.92	0.004	35.7	11.89	11.88	11.89	11.89	0.00	1.08	1.1	94.3
6	PR 41	3.75	3.76	3.77	3.76	0.0111	95.8	3.68	3.70	3.68	3.69	0.007	93.1	3.68	3.65	3.65	3.66	0.018	96.2	4.40	4.40	4.39	4.40	0.005	42.9	15.99	15.98	15.98	15.98	0.00	1.20	1.4	97.1
7	PR 17	3.70	3.74	3.70	3.71	0.0214	94.2	3.69	3.69	3.70	3.69	0.005	93.2	3.64	3.65	3.60	3.63	0.024	95.0	4.11	4.11	4.13	4.12	0.010	38.7	11.15	11.13		11.14	0.02	1.05	1.1	93.7
8	PR 11	3.27	3.29	3.29	3.28	0.0119	78.8	3.20	3.21	3.23	3.22	0.014	76.7	2.66	2.61	2.64	2.64	0.022	58.4	4.16	4.16	4.15	4.16	0.005	39.3	13.18	13.17	13.16	13.17	0.01	1.12	1.3	95.7
9	PR 47	3.80	3.81	3.73	3.78	0.0421	96.5	3.74	3.77	3.75	3.75	0.015	95.3	3.73	3.72	3.69	3.71	0.023	98.1	4.46	4.47	4.47	4.47	0.003	44.0	17.00	17.00	16.99	17.00	0.00	1.23	1.5	97.8
10	PR 54	3.64	3.64	3.58	3.62	0.0323	90.8	3.57	3.58	3.60	3.58	0.015	89.4	3.57	3.56	3.53	3.56	0.020	92.4	4.24	4.25	4.26	4.25	0.009	40.8	14.17	14.19	14.18	14.18	0.01	1.15	1.3	96.4
11	PR 19	3.42	3.42	3.41	3.42	0.0068	83.7	3.42	3.42	3.36	3.40	0.033	83.2	3.44	3.51	3.31	3.42	0.104	87.3	3.97	3.97	3.98	3.97	0.003	36.6	9.54	9.54	9.53	9.54	0.01	0.98	1.0	91.3
12	PR 42	3.91	3.91	3.83	3.88	0.0437	100.1	3.84	3.89	3.86	3.86	0.024	99.2	3.83	3.81	3.78	3.81	0.023	101.6	4.57	4.57	4.56	4.57	0.006	45.5	17.39	17.40	17.38	17.39	0.01	1.24	1.5	97.9
13	PR 44	3.85	3.86	3.78	3.83	0.0410	98.2	3.79	3.78	3.79	3.79	0.006	96.5	3.77	3.75	3.74	3.75	0.020	99.5	4.48	4.49	4.49	4.48	0.003	44.3	18.76	18.85	18.91	18.84	0.08	1.28	1.6	98.3
14	PR 75	3.96	3.94	4.01	3.97	0.0353	103.2	3.91	3.91	3.94	3.92	0.016	101.2	3.90	3.98	3.86	3.91	0.060	105.6	4.62	4.62	4.62	4.62	0.001	46.3	18.73	18.78	18.76	18.76	0.03	1.27	1.6	98.3
15	PR 58	3.66	3.70	3.67	3.68	0.0182	92.8	3.61	3.63	3.63	3.62	0.009	90.8	3.60	3.86	3.56	3.67	0.160	96.6	4.29	4.30	4.30	4.30	0.004	41.4	15.39	15.65	15.83	15.62	0.22	1.19	1.4	97.3
16	PR 61	3.70	3.71	3.65	3.68	0.0329	93.1	3.64	3.65	3.66	3.65	0.007	91.8	3.63	3.60	3.58	3.61	0.027	94.2	4.29	4.29	4.29	4.29	0.003	41.4	15.49	15.50	15.71	15.57	0.12	1.19	1.4	97.3
17	PR 53	4.22	4.22	4.14	4.19	0.0468	111.2	4.14	4.15	4.16	4.15	0.012	109.2	4.10	4.12	4.08	4.10	0.018	112.5	4.97	4.96	4.97	4.97	0.005	51.6	24.74	24.53	24.75	24.67	0.13	1.39	1.8	99.5
18	PR 51	4.13	4.14	4.06	4.11	0.0446	108.2	4.05	4.07	4.08	4.07	0.013	106.2	4.06	4.04	4.01	4.04	0.027	110.1	4.76	4.75	4.75	4.75	0.003	48.4	21.86	21.55	21.78	21.73	0.16	1.34	1.7	99.0
19	PR 66	3.34	3.36	3.37	3.36	0.0110	81.4	3.29	3.30	3.31	3.30	0.011	79.6	3.27	3.24	3.21	3.24	0.029	80.7	3.94	3.93	3.93	3.94	0.003	36.0	10.15	10.06	10.16	10.12	0.06	1.01	1.0	92.7
20	PR 74	4.65	4.58	4.59	4.60	0.0401	125.7	4.56	4.58	4.58	4.57	0.011	123.9	4.52	4.54	4.51	4.52	0.016	128.1	5.22	5.22	5.23	5.23	0.002	55.5	29.02	28.95	28.95	28.97	0.04	1.46	2.0	99.9
21	PR8	3.63	3.56	3.58	3.59	0.0370	89.8	3.64	3.59	3.60	3.61	0.029	90.3	3.56	3.52	3.48	3.52	0.044	91.0	4.21	4.22	4.22	4.22	0.005	40.3								
22	PR109	3.52	3.44	3.43	3.46	0.0477	85.3	3.46	3.45	3.47	3.46	0.009	85.1	3.47	3.42	3.38	3.42	0.044	87.4	4.14	4.14	4.15	4.14	0.005	39.1								
23	PR 30	3.87	3.89	3.80	3.85	0.0461	99.1	3.81	3.82	3.83	3.82	0.010	97.7	3.81	3.78	3.74	3.77	0.034	100.4	4.54	4.54	4.54	4.54	0.003	45.2								
24	PR 21	3.74	3.74	3.67	3.72	0.0436	94.2	3.65	3.67	3.68	3.67	0.017	92.3	3.67	3.63	3.60	3.63	0.031	95.2	4.34	4.33	4.33	4.33	0.003	42.0								
25	PR 14	3.19	3.18	3.18	3.19	0.0070	75.4	3.17	3.18	3.15	3.17	0.015	75.0	3.17	3.08	3.06	3.10	0.056	75.7	3.63	3.63	3.63	3.63	0.001	31.3								
26	PR 31	3.87	3.87	3.86	3.87	0.0049	99.7	3.80	3.82	3.82	3.81	0.010	97.4	3.81	3.77	3.76	3.78	0.030	100.6	4.68	4.55	4.55	4.60	0.072	46.0								
27	PR 33	3.84	3.85	3.80	3.83	0.0269	98.3	3.78	3.80	3.77	3.78	0.019	96.4	3.78	3.75	3.75	3.76	0.018	99.9	4.51	4.53	4.53	4.52	0.011	44.9								
28	PR 37	3.95	3.88	3.89	3.91	0.0398	101.0	3.88	3.89	3.90	3.89	0.009	100.1	3.90	3.86	3.74	3.83	0.084	102.6	4.60	4.61	4.62	4.61	0.010	46.1								
29	PR 48	3.83	3.83	3.78	3.81	0.0292	97.6	3.77	3.80	3.84	3.80	0.038	97.1	3.77	3.73	3.46	3.65	0.165	95.9	4.49	4.51	4.51	4.50	0.009	44.6								
30	PR 56	3.63	3.62	3.62	3.62	0.0085	91.0	3.58	3.56	3.58	3.57	0.008	89.0	3.56	3.64	3.71	3.64	0.073	95.4	4.23	4.24	4.23	4.23	0.007	40.5								
31	PR 59	3.49	3.43	3.44	3.45	0.0294	84.9	3.45	3.46	3.46	3.46	0.010	85.0	3.47	3.41	3.48	3.45	0.038	88.4	4.08	4.09	4.09	4.09	0.006	38.2								
32	PR 62	3.74	3.66	3.67	3.69	0.0442	93.2	3.68	3.68	3.69	3.68	0.009	93.0	3.69	3.69	3.39	3.59	0.174	93.7	4.30	4.30	4.31	4.31	0.007	41.6								
33	PR 70	3.94	3.86	3.86	3.88	0.0460	100.2	3.87	3.88	3.89	3.88	0.014	99.7	3.88	3.84	3.65	3.79	0.120	101.0	4.58	4.58	4.59	4.58	0.003	45.8								
34	PR 18	3.26	3.19	3.20	3.21	0.0403	76.4	3.19	3.20	3.22	3.20	0.017	76.3	3.19	3.15	3.87	3.40	0.403	86.6	3.77	3.78	3.77	3.77	0.002	33.5								
35	PR 24	3.13	3.17	3.14	3.15	0.0202	74.1	3.18	3.14	3.14	3.15	0.021	74.5	3.11	3.08	3.11	3.10	0.018	75.5	3.63	3.63	3.64	3.63	0.005	31.4								
36	PR 25	3.34	3.39	3.45	3.39	0.0505	82.8	3.33	3.34	3.36	3.34	0.014	81.0	3.29	3.30	3.32	3.30	0.018	83.0	3.89	3.89	3.89	3.89	0.003	35.3								
37	PR 60	3.56	3.57	3.50	3.55	0.0379	88.2	3.50	3.51	3.52	3.51	0.011	87.0	3.51	3.48	3.52	3.50	0.023	90.3	4.15	4.15	4.15	4.15	0.002	39.2								
38	PR 23	3.79	3.72	3.72	3.74	0.0437	95.2	3.72	3.73	3.74	3.73	0.007	94.6	3.69	3.69	3.73	3.70	0.023	97.7	4.37	4.38	4.37	4.37	0.003	42.6								
39	PR 50	4.06	4.07	4.03	4.05	0.0225	106.2	3.99	4.00	4.01	4.00	0.012	103.9	4.01	3.96	4.02	4.00	0.031	108.6	4.80	4.80	4.79	4.80	0.006	49.0								
40	PR 72	4.28	4.27	4.28	4.28	0.0056	114.1	4.23	4.21	4.20	4.21	0.014	111.3	4.23	4.18	4.23	4.22	0.029	116.7	4.82	4.81	4.81	4.81	0.003	49.3								
41	Venlafaxine	2.76	2.77	2.77	2.76	0.0100	53.5	3.83	3.84	3.83	3.83	0.000	82.2	4.81	4.82	4.82	4.82	0.010	92.9	3.70	3.71	3.71	3.71	0.010	29.9	1.50	1.69	1.69	1.63	0.21	0.11	-0.55	22.4
42	Mirtazapine	2.43	2.44	2.44	2.44	0.0100	46.7	4.41	4.39	4.39	4.39	0.010	101.2	4.27	4.26	4.28	4.27	0.010	79.0	4.08	4.07	4.54	4.23	0.270	39.3	4.77	4.68	4.66	4.70	0.67	0.06	0.51	76.9
43	Amitriptyline	3.36	3.36	3.37	3.36	0.0000	65.9	4.73	4.71	4.70	4.71	0.010	112.0	5.74	5.73	5.75	5.74	0.010	116.3	4.65	4.65	4.66	4.65	0.010	47.0	6.79	6.85	6.72	6.79	0.83	0.06	0.87	88.9
44	Desipramine	3.27	3.27	3.27	3.27	0.0000	64.0	4.36	4.33	4.34	4.34	0.010	99.5	5.23	5.25	5.27	5.25	0.020	103.9	4.54	4.54	4.54	4.54	0.000	44.9	5.90	5.95	6.05	5.97	0.78	0.08	0.74	85.5
45	Mianserin	3.05	3.04	3.05	3.05	0.0100	59.4	4.62	4.60	4.61	4.61	0.010	108.6	5.04	5.05	5.05	5.05	0.000	98.7	4.50	4.50	4.50	4.50	0.000	44.2	7.41	7.38	7.38	7.39	0.87	0.02	0.95	90.9
	**Calibration SET**																																
	Theophylline	1.20	1.22	1.21	1.21	0.0072	17.9	1.59	1.58	1.59	1.59	0.005		1.21	1.26	1.27	1.25	1.26															
	Benzimidazole	1.66	1.61	1.60	1.62	0.0327	6.3	1.92	1.91	1.93	1.92	0.008		1.79	1.83	1.85	1.82	1.83															
	Colichicine	2.60	2.58	2.58	2.58	0.0121	43.9	2.59	2.59	2.59	2.59	0.000		2.49	2.53	2.60	2.54	2.56															
	Acetophenone	2.67	2.66	2.66	2.66	0.0056	64.1	2.66	2.66	2.66	2.66	0.003		2.54	2.61	2.61	2.59	2.60															
	Indole	2.98	2.98	2.99	2.98	0.0064	72.1	3.01	3.00	3.01	3.01	0.006		2.89	2.88	2.87	2.88	2.88															
	Propiophenone	3.18	3.08	3.08	3.11	0.0575	77.4	3.08	3.09	3.08	3.08	0.009		2.97	3.02	3.02	3.00	3.01															
	Butyrophenone	3.59	3.51	3.51	3.53	0.0457	87.3	3.54	3.51	3.53	3.53	0.017		3.40	3.45	3.42	3.42	3.43															
	Valerophenone	3.94	3.85	3.86	3.88	0.0471	96.4	3.88	3.85	3.87	3.87	0.016		3.75	3.79	3.79	3.78	3.79															
	Paracetamol																		Paracetamol	1.88	1.88	1.88	1.88	0.00	2.9								
	Acetanilidine																		Acetanilidine	2.31	2.31	2.31	2.31	0.00	11.5								
	Acetophenone																		Acetophenone	2.61	2.61	2.60	2.61	0.00	17.2								
	Propiohenone																		Propiohenone	3.20	3.20	3.19	3.19	0.01	25.9								
	Butyrophenone																		Butyrophenone	3.66	3.66	3.65	3.65	0.01	32								
	Valerophenone																		Valerophenone	4.04	4.05	4.03	4.04	0.01	37.3								
	Hexanophenone																		Hexanophenone	4.37	4.37	4.36	4.36	0.01	41.8								
	Heptanophenone																		Heptanophenone	4.63	4.63	4.62	4.63	0.01	45.7								
	Octanophenone																		Octanophenone	4.86	4.87	4.83	4.85	0.02	49.4								
	Paracetamol																		paracetamol							1.16	1.15	1.15	1.15	0.01	0.06		
	Nizatidine																		nizatidine							1.38	1.36	1.40	1.38	0.02	0.14		
	Trimetoprim																		trimetoprim							2.18	2.18	2.18	2.18	0.00	0.34		
	Carbamazepine																		carbamazepine							4.06	4.07	4.09	4.07	0.02	0.61		
	Propranolol																		propranolol							10.62	10.66	10.62	10.63	0.02	1.03		
	Nicardipine																		nicardipine							11.74	11.78	11.87	11.80	0.07	1.07		
	Warfarin																		warfarin							16.35	16.41	16.46	16.41	0.06	1.22		
	Diclofenac																		diclofenac							23.07	23.03	23.21	23.10	0.10	1.36		
	Indometacin																		indometacin							23.65	23.79	24.22	23.89	0.30	1.38		
